# Pulse oximetry adoption and oxygen orders at paediatric admission over 7 years in Kenya: a multihospital retrospective cohort study

**DOI:** 10.1136/bmjopen-2021-050995

**Published:** 2021-09-07

**Authors:** Timothy Tuti, Jalemba Aluvaala, Samuel Akech, Ambrose Agweyu, Grace Irimu, Mike English, The Clinical Information Network Author Group, Lynda Isaaka

**Affiliations:** 1 Health Services Unit, KEMRI-Wellcome Trust Research Programme, Nairobi, Kenya; 2 Department of Paediatrics and Child Health, University of Nairobi, Nairobi, Kenya; 3 Nuffield Department of Medicine and Department of Paediatrics, University of Oxford, Oxford, UK

**Keywords:** quality in health care, paediatrics, audit, epidemiology, protocols & guidelines, tropical medicine

## Abstract

**Objectives:**

To characterise adoption and explore specific clinical and patient factors that might influence pulse oximetry and oxygen use in low-income and middle-income countries (LMICs) over time; to highlight useful considerations for entities working on programmes to improve access to pulse oximetry and oxygen.

**Design:**

A multihospital retrospective cohort study.

**Settings:**

All admissions (n=132 737) to paediatric wards of 18 purposely selected public hospitals in Kenya that joined a Clinical Information Network (CIN) between March 2014 and December 2020.

**Outcomes:**

Pulse oximetry use and oxygen prescription on admission; we performed growth-curve modelling to investigate the association of patient factors with study outcomes over time while adjusting for hospital factors.

**Results:**

Overall, pulse oximetry was used in 48.8% (64 722/132 737) of all admission cases. Use rose on average with each month of participation in the CIN (OR: 1.11, 95% CI 1.05 to 1.18) but patterns of adoption were highly variable across hospitals suggesting important factors at hospital level influence use of pulse oximetry. Of those with pulse oximetry measurement, 7% (4510/64 722) had hypoxaemia (SpO_2_ <90%). Across the same period, 8.6% (11 428/132 737) had oxygen prescribed but in 87%, pulse oximetry was either not done or the hypoxaemia threshold (SpO_2_ <90%) was not met. Lower chest-wall indrawing and other respiratory symptoms were associated with pulse oximetry use at admission and were also associated with oxygen prescription in the absence of pulse oximetry or hypoxaemia.

**Conclusion:**

The adoption of pulse oximetry recommended in international guidelines for assessing children with severe illness has been slow and erratic, reflecting system and organisational weaknesses. Most oxygen orders at admission seem driven by clinical and situational factors other than the presence of hypoxaemia. Programmes aiming to implement pulse oximetry and oxygen systems will likely need a long-term vision to promote adoption, guideline development and adherence and continuously examine impact.

**Strengths and limitations of this study**

- The data presented are a rare example of long-term tracking of hospital practice in a routine low-income and middle-income country (LMIC) setting presenting a useful illustration of the process of pulse oximetry adoption in LMIC hospitals that has broader implications for thinking on technology adoption.
- We employ a complex modelling framework to explore patient-level and hospital-level factors influencing outcomes of interest at admission (pulse oximetry done, and oxygen prescribed) over time that accommodate and account for between hospital variability, within hospital non-linear trends in the outcomes over time and entry into the clinical network at different time points.
- We have no contemporaneous data from Kenyan settings that do not belong to the Clinical Information Network for comparison with most other studies exploring the use of pulse oximetry essentially being cross sectional.

## Introduction

### Background

Compared with clinical signs (eg, central cyanosis, indrawing), pulse oximetry is felt to be easy to use and posited to be at least 20% more accurate in identifying children at risk of hypoxaemia (defined as a blood oxygen saturation of <90%).^1 2^ As pulse oximetry is an inexpensive tool, it is now recommended for guiding the assessment of illness severity and treatment for respiratory illnesses, including increasing emphasis on its role for screening children with respiratory illness in primary care in low-income and middle-income country (LMIC).^1 2^ As hypoxaemia may be found in all severe childhood illnesses such as sepsis, meningitis, severe acute malnutrition and malaria, its use is widely recommended as a screening tool in all paediatric admissions as a fifth vital sign. The advent of COVID-19 has further invigorated programmatic efforts to scale up pulse oximetry together with improved oxygen delivery systems.^3–9^

Despite being recommended for many years, there is little long-term data on the adoption of pulse oximetry in LMIC, even from hospitals. Data on use during the COVID-19 pandemic are also lacking from such settings as are large studies from routine settings on whether pulse oximeter readings influence the use of oxygen. We and others have described that adoption is undermined by system factors such as inadequate supply and repair of oximeters and if healthcare workers have insufficient training on when, how, and why to use them and interpret their results.^9 10^ This compounds wider and similar problems in managing oxygen systems themselves.^11^ Our earlier work used data from seven Kenyan hospitals between September 2013 and February 2016. Here, we update and extend these analyses to include data from paediatric admissions in a growing network of Kenyan hospitals before and after prolonged national health workers strikes in 2017, including data obtained during the COVID-19 pandemic.^12^ The objective of this study was to characterise adoption and explore specific patient, clinical and hospital factors that might influence pulse oximetry and oxygen use and highlight useful considerations for entities working on programmes to improve access to pulse oximetry and oxygen.^13–15^

## Methods

### Ethics and reporting

The reporting of this observational study follows the Strengthening the Reporting of Observational Studies in Epidemiology statement.^16^ The Scientific and Ethics Review Unit of the Kenya Medical Research Institute (KEMRI) approved the collection of the deidentified data that provides the basis for this study as part of the Clinical Information Network (CIN). The CIN is run in partnership with the Ministry of Health and participating hospitals with aims to improve the quality of routine paediatric hospital data for use in improvement activities, observational and interventional research.^17 18^ Individual consent for access to deidentified patient data was not required.

### Patient and public involvement

No patients were involved in the design, conduct, reporting or dissemination plans of our research except through the KEMRI ethical review process where they have representatives.

### Study design and setting

From a broad context perspective, in many LMICs including Kenya, hospital management and monitoring systems are weak, with major human and material resource constraints. These challenges affect hospitals’ delivery of inpatient maternal, surgical and adult medical care as well as paediatric and neonatal care. Consequently, there is very limited organisational and resource slack to mobilise for any new purpose. Interventions such as oximetry and oxygen seeking to achieve large scale change must therefore either consider how to mobilise new resources or consider what is achievable with limited resources.^19 20^ We have described the broader context of the Kenyan healthcare system with reference to the paediatric burden of disease in great detail elsewhere.^19 20^

We report a retrospective cohort study of 18 public hospitals in Kenya largely providing first-referral level care predominantly admitting patients that present directly to the facility, and purposefully selected to be of at least moderate size and representative of different malaria transmission zones. Hospitals joined the CIN at different calendar time points between 2013 and 2017. Few of their patients may be formally referred from primacy care facilities but there are few functional referral mechanisms such as ambulance systems.^21^ Pulse oximetry has generally not been available outside hospitals in the public sector.^22^ Pneumonia is the major killer of children across the country except in settings where malaria is highly endemic (9/18 hospitals in this study).^21^

The hospitals receive 3 monthly clinical audit and feedback reports on the quality of care they provide for common conditions.^23^ Paediatric team leaders (paediatricians and nurses) met face to face once or twice annually until 2019 (before the pandemic) to discuss these reports and how to improve clinical care. From 2018, participation in more specific research studies was also discussed in meetings with hospitals. This resulted in two studies being initiated in subsets of the CIN hospitals which might have influenced the adoption of pulse oximetry. Hospital participation in these studies was not mutually exclusive: a hospital could be part of one, both or none of the research studies. Not all hospitals recruited in the same study were recruited at the same time. Summary details of these studies are provided in table 1 with greater detail on which hospitals participated in the studies provided in online supplemental tables 1 and 2. The 18 hospitals included in the study had a median of 1 pulse oximeter(s) per paediatric ward (IQR: 1–3) (online supplemental table 1).

10.1136/bmjopen-2021-050995.supp1 Supplementary data

**Table 1:** Description of studies undertaken in Clinical Information Network hospitals

Study name	Study site selection rationale and period	Goal	Extra resources provided to hospitals (H) by projects*	Extra notes
RTS, S/AS01 trial: (ClinicalTrials.gov Identifier: NCT03806465)	Study start: December 2018 Site selection: by MoH/WHO, selecting high volume public hospitals in regions getting the vaccine.	The evaluation of the feasibility, safety and impact of the RTS, S/AS01 malaria vaccine in paediatric cases in Kenya	One extra healthcare worker provided per paediatric ward. Extra pulse oximetry equipment provided per ward: One item: H4	Clinician role: promote compliance with guidelines for meningitis and malaria including complete assessment and investigations including the performance of lumbar punctures, and consenting participants for storage of cerebrospinal fluid storage
SEARCH trial (ClinicalTrials.gov Identifier: NCT04041791)	Started in two phases: March 2019 Hospitals: H3, H9, H12, H13, H16 June 2019 Hospitals: H1, H2, H6, H10, H11 Site selection: from the expected size of pneumonia population, the geographical balance of hospitals and support from local hospital teams	Pragmatic factorial individually randomised controlled trial to compare alternative antibiotics and alternative modes of fluid therapy for children with severe pneumonia	One extra healthcare worker provided per participating hospital. Extra pulse oximetry equipment provided to the hospital: Two items: H3, H6, H9, H10, H11, H12, H13, H16 One item: H1, H2	Clinician role: promote compliance with the study protocol, including completing patient assessment, investigations and diagnoses. Ensure appropriate administration of study interventions. Recruitment of study participants was suspended on the 8 April 2020 due to COVID-19 pandemic. Adult inpatient surveillance in addition to paediatric surveillance began on the 4 May 2020.

H15 got one pulse oximeter in June 2020 even though it was not part of any study.

*We were not able to track and record introduction of pulse oximeters procured directly by hospitals or other sources, or those personally owned by healthcare workers, nor ability to repair existing pulse oximeters when required.

MoH, Ministry of Health.

Recording of pulse oximetry values has been included as part of a structured paediatric admission record (PAR)^24^ used since 2013. Hospitals joining the CIN agree to provide the PAR themselves and promote its use—the purpose of the PAR is to prompt admitting clinicians to fully assess children and rapidly document their findings.^24^ Emergency Triage Assessment and Treatment plus Admission Care (ETAT+) training^25^ has been used in Kenya since 2008 and was adapted in 2013 so that pulse oximetry was a recommended part of the assessment in all sick children and especially those with danger signs. Many of the junior and senior medical staff in CIN hospitals would have received ETAT+training (eg, as an undergraduate or postgraduate course or as in-service training).^25^ Additionally, the Basic Paediatric Protocols that are widely disseminated have specifically referred to the use of pulse oximetry—if available—for all pneumonia cases since 2016.^26^ The promotion of PAR use by hospitals, adaption and scale up of ETAT+training, and dissemination of the basic paediatric protocols to clinicians nationally are posited to have a system-wide effect on the adoption of pulse oximetry use.

### Study participants

All medical admissions to paediatric wards (ages 0–13 years), but not those admitted to specific newborn units, to the selected hospitals between 1 February 2014, or the date at which a hospital joined CIN, and 31 December 2020 were eligible for inclusion in this study. We excluded children whose admission or discharge dates were missing or improbable (eg, discharge date is earlier than admission date), and those whose admission fell within 2017, a period of prolonged health worker strikes that resulted in major disruption to healthcare delivery.^12^ The expectation was that all would have pulse oximetry performed at admission given they were sick enough to warrant inpatient care but among these we also report the proportion of children with danger signs comprising any level of altered consciousness or with recorded signs of respiratory distress especially deserving of pulse oximetry.^10 26 27^

### Data sources and management

Methods of collection and cleaning of data in the CIN are reported in detail elsewhere.^28^ Clinical data for paediatric admissions to the hospitals within the CIN are captured through PAR forms^27^ that are approved by the Ministry of Health. The PAR prompts the clinician with a checklist of fields including patient biodata, clinical assessment, admission and discharge diagnoses, treatments and to record outcome (survival or death). The CIN supports one data clerk in each hospital to extract data from paper medical records, nursing charts, treatment charts and available laboratory reports each day after children’s discharge into the primary data collection tool developed in Research Electronic Data Capture. Automated error checking happens at the point of entry by daily review, every week centrally and both are complemented by regular external data quality assurance reviews.^28^ A minimal dataset—which is unsuitable for these pulse oximetry analyses—is collected for (1) all admissions with a burn or a surgical diagnosis to the paediatric ward(s), (2) admissions during major holiday breaks, (3) admissions when the data clerk was on leave and (4) on a random selection of records in hospitals where the workload is very high. This process is explained in detail elsewhere.^28 29^

### Descriptive analysis

To characterise adoption of pulse oximetry at admission over time, we use pooled data from the period February 2014 to November 2018 (excluding 2017) from 13 network hospitals. From December 2018 data were available from 17 hospitals after 5 joined the CIN linked to new studies, and one (H7) exited. To explore patterns in more detail, we employed two approaches: (1) for the period from December 2018 to December 2020, we plot data pooled for hospital subgroups depending on which research studies they were taking part in as these might influence pulse oximetry use; (2) we plot adoption within each hospital for the number of months it was part of the network, again excluding 2017 where relevant. This latter approach was used to reveal the extent of variability in adoption at the hospital level.

For the individual patient population, we tabulated and summarised categorical data as proportions, with continuous variables reported as medians (IQR) if they were not normally distributed. From the included paediatric admission patient population data, we report the prevalence of important clinical features such as respiratory distress symptoms (eg, central cyanosis, indrawing, grunting and difficulty breathing), circulatory symptoms (eg, slow capillary refill, less than three seconds), level of consciousness, and whether the patient was being readmitted or referred to the hospital. We also report the prevalence of summary clinical features such as having any sign of respiratory distress and any danger sign and the proportion for whom oxygen was ordered as a treatment at admission.

### Statistical methods

To examine two binary outcomes, (1) pulse oximetry used and, (2) oxygen ordered at admission, we modelled individual patient data. Our key explanatory variable was time as part of (or exposure to) the CIN, computed as the number of months since joining the CIN for a specific hospital. This allowed associations with these outcomes to be explored in hospitals while accounting for clustering by both hospital and time in months since the hospital joined the CIN; the analysis of pulse oximetry use employs a much larger dataset to extend our earlier findings.^10^

Predictors included in our models were patients’ age and sex, and the record of presence or absence of respiratory signs and symptoms, circulatory symptoms, level of consciousness, whether the patient was being readmitted or referred to the hospital. At the hospital level, we included terms for their specific identity and malaria endemicity zone. Hospital participation in one of the four research study subgroups was included as a covariable at the patient level to take account of possible effects occurring only after a hospital joined specific studies.

We modelled whether oxygen was prescribed at admission with an interest in whether patients with a recorded pulse oximetry value (SpO_2_) <90% at admission would be started on oxygen therapy. Where the pulse oximetry value at admission was missing, based on findings from a previous CIN study, the assumption was that the clinician did not have the information.^10^ The analysis therefore explored (1) whether pulse oximetry values (SpO_2_ <90%) were associated with oxygen prescription and (2) in the absence of a pulse oximetry value <90%, which clinical signs are associated with the prescription of oxygen therapy.

We included the variables of interest in hierarchical multivariate logistic regression models, with a random intercept for each hospital and a random slope for the time within hospital. This model specification commonly referred to as a growth curve model, allowed the explanatory variable of time to have a different effect for each hospital. A *growth curve model* typically refers to statistical methods that allow, in our case, the estimation of interhospital variability in intrahospital patterns of change over time.^30^ Our approach to the growth-curve model fitting is within the multilevel modelling framework, with patients nested in hospitals nested in time points.^30^ Different ways of specifying the growth-curve model using a multilevel modelling framework are explained in detail elsewhere.^31^ Before embarking on the growth-curve modelling, we examined for each hospital whether the 2017 strike resulted in a significant discontinuity in pulse oximetry use to check if treating time as months in the CIN, a continuous variable, instead of as calendar time was justifiable given the strike caused a 12-month data gap.

Where there were missing patient-level data, we applied fully conditional specification multivariate imputation by chained equations^32^ to generate imputed datasets under the missing at random (MAR) assumption to allow us to analyse all eligible patients. Previous studies indicate that MAR is a reasonable approach for CIN data.^33^ We contrasted findings using imputation with the findings of the complete case analysis.

## Results

### Descriptive findings

**Figure 1:**
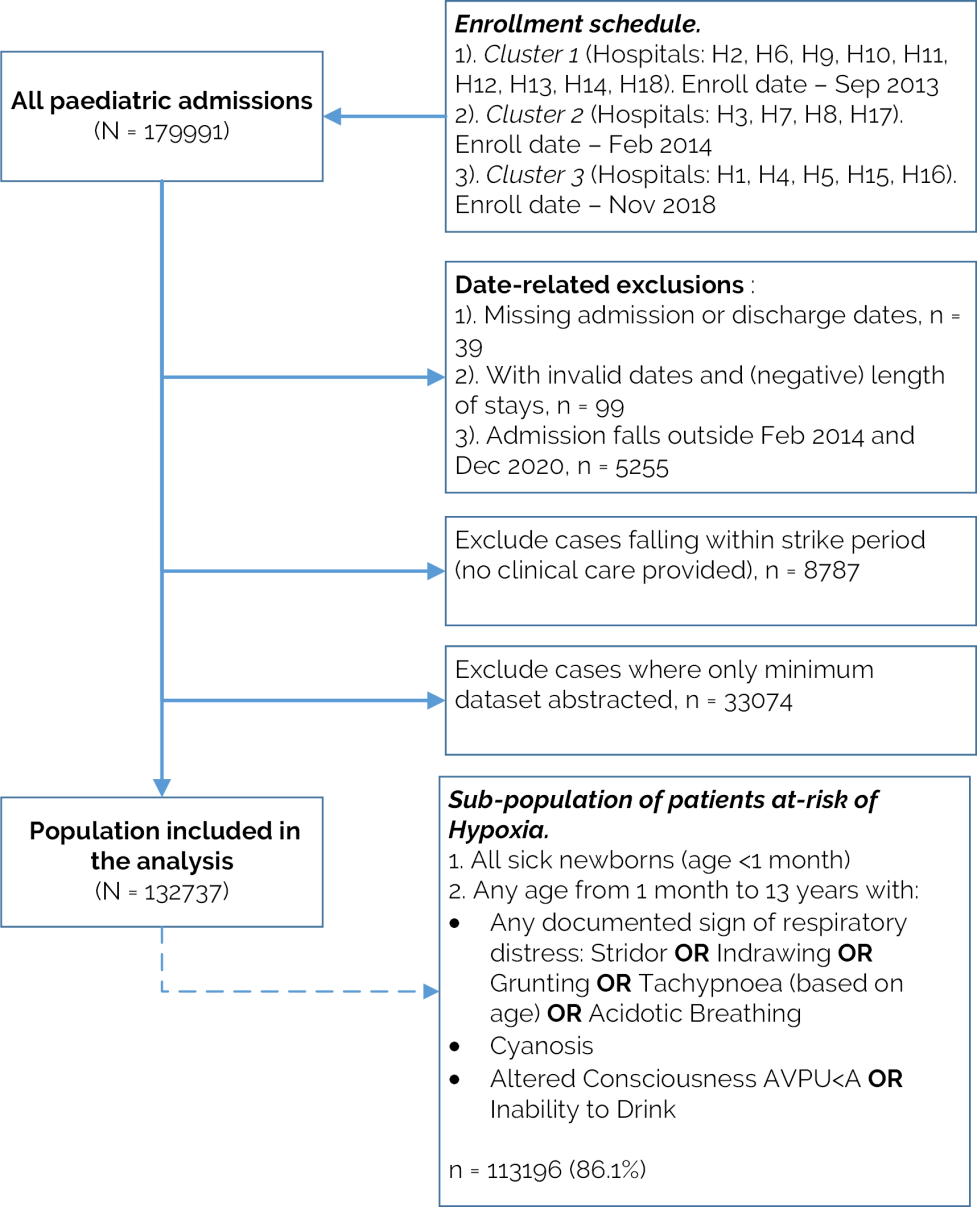
Flow chart of the inclusion criteria. AVPU, Alert, Verbal, Pain, Unresponsive.

Figure 1 depicts the study population inclusion process. Out of the 179 991 paediatric admissions to CIN hospitals, 132 737 (73.7%) were eligible for analysis. Most exclusions were because an admission was randomly sampled for minimum data collection (n=33 074, 70% of the exclusions) or fell in the strike year (n=8787, 19% exclusions). Of the population included in the analysis, 113 196/132 737 (86.1%) demonstrated at least one sign indicating increased risk of hypoxaemia (respiratory distress symptoms or altered consciousness, or age below 1 month). Given this high proportion, we report results based on data from all eligible admissions (figure 1).

**Figure 2:**
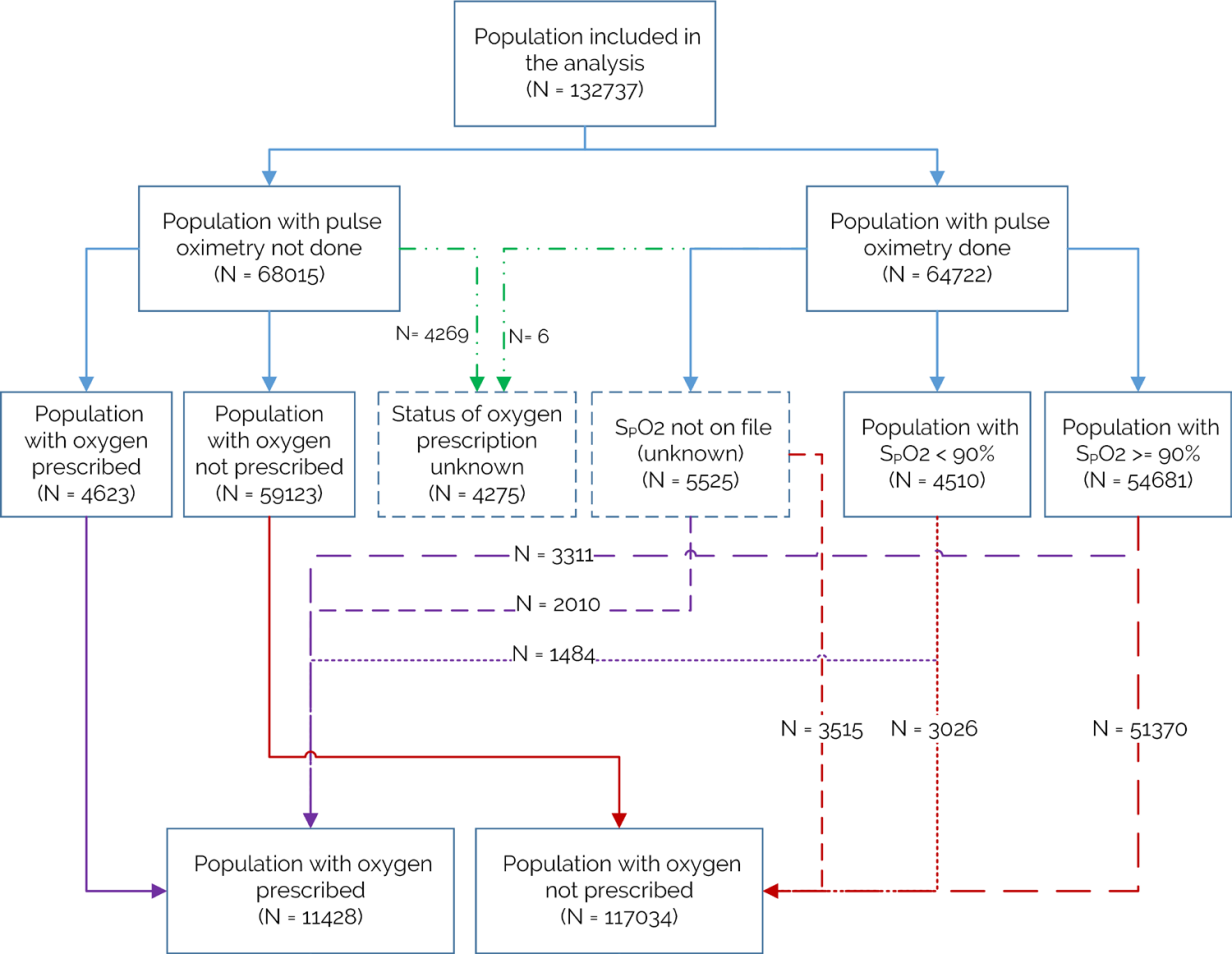
Oxygen prescription and use of pulse oximetry at admission.

Of the 132 737 cases, 48.8% (n=64 722) had a pulse oximetry measurement taken at admission. Of the 64 722 patients with pulse oximetry measured at admission, 4510 (7%) had hypoxaemia (SpO_2_ <90%). Across the same period, 11 428/132 737 (8.6%) were documented to have oxygen prescribed at admission. Of these, only 1484/11 428 (12.8%) were confirmed to be hypoxaemic using pulse oximetry, while 6633/11 428 (58%) had no pulse oximetry measurement and 3311/11 428 (28.9%) were documented to have a pulse oximetry value of 90% or higher at admission. Thus, 87% oxygen use was not pulse oximetry guided (SpO_2_ ≥90% or unknown) and more than twice as many children prescribed oxygen had values ≥90% (3311 compared with 1484) (figure 2).

**Figure 3:**
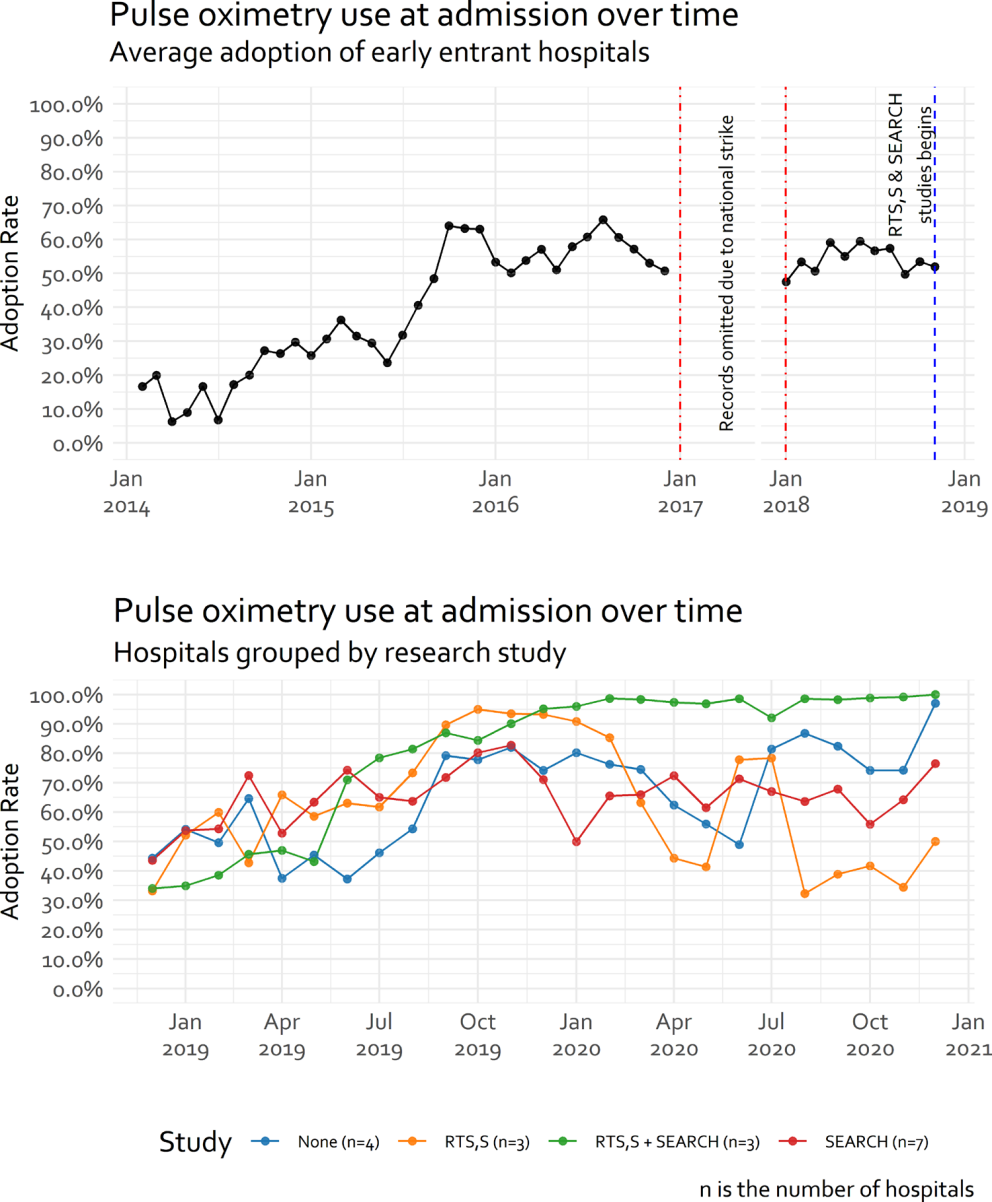
Average pulse oximetry use in the Clinical Information Network (CIN) hospitals over time. The number of hospitals in each study covers both presently involved and those joining the CIN later: there are 13 hospitals in the period between February 2014 and November 2018, with the lower panel representing transition to 18 hospitals by adding 5 hospitals and research studies beginning. The 5 new hospitals’ distribution among the studies in the following way: RTS, S: 2/5; RTS, S+SEARCH: 1/5; SEARCH: 1/5; None: 1/5.

From February 2014 to December 2016, on average, there was a steady improvement in pulse oximetry adoption at admission in the 13 hospitals from between 10% and 20% in 2014 to between 50% and 70% at the end of 2016 (figure 3), even though no pulse oximetry devices were provided through CIN. Following the strikes in 2017, from January 2018 when services resumed up until November 2018, average pulse oximetry adoption in these 13 sites seemed to stabilise at around 50%. By December 2018, one hospital exited and five hospitals joined CIN and different research studies were initiated in subsets of hospitals (table 1, figure 3 and online supplemental table 2). Pulse oximetry adoption ranged from an average of 40% to almost 100% across these subsets (figure 3).

**Figure 4:**
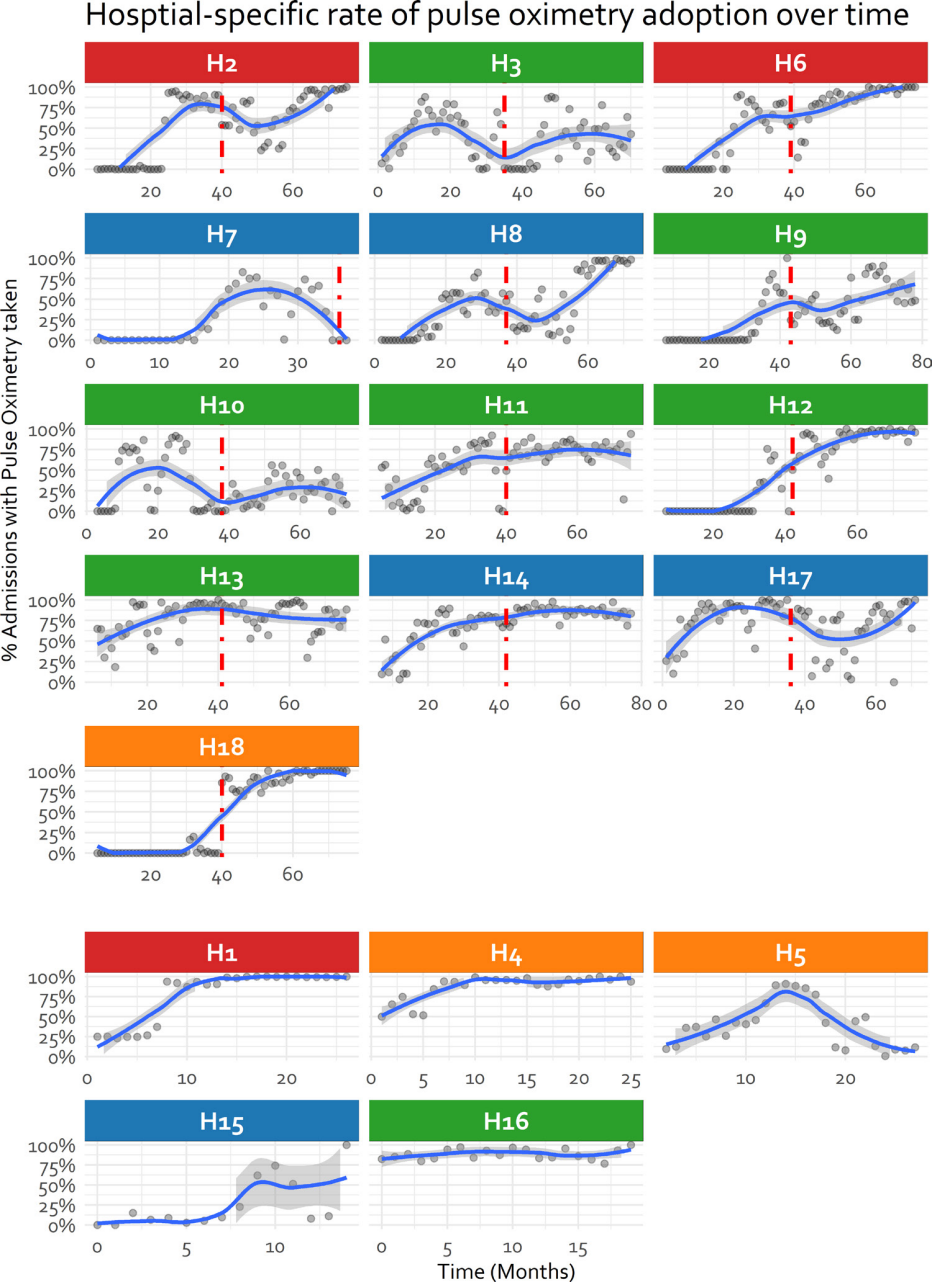
Hospital specific rate of pulse oximetry use at admission over time divided into sites joining from January 2014 and those recruited from December 2018. Vertical red line indicates national strike disruption. The title is colour-coded to reflect the research study the hospital was recruited into (Blue: None, Orange: RTS, S, Red: SEARCH, Green: RTS, S+SEARCH). H7 exited from Clinical Information Network and is therefore not included in subsequent analysis.

Exploring each hospital’s adoption trajectory illustrates in many a general improvement in adoption throughout their participation in CIN but also examples of adoption failure (figure 4). Figure 4 tends to confirm as depicted in figure 3, that despite a strike of almost 1 year there was minimal evidence of discontinuity in hospitals’ pattern of adoption from 2016 to 2018. The lack of evidence for discontinuity (supported by additional analyses in online supplemental table 3) supports our treatment of time as ‘months in CIN’ as a continuous variable in place of calendar time in figures 3 and 4 and subsequent models.

From figure 4, 8 of the 13 hospitals recruited into the CIN by February 2014 had no use of pulse oximetry at admission at all (median at baseline 0%), with the highest baseline use being 65% (H13). For the hospitals joining after the strike (ie, from November 2018), use of pulse oximetry during admission was generally low for 4/5 hospitals (median at baseline 25%) but one hospital already used pulse oximetry on 83% admissions (H16) (figure 4). Hospitals demonstrated various patterns of adoption: (1) low adoption when joining the CIN with a sharp improvement later (eg, hospital H2, H12), (2) oscillation between low and high pulse oximetry use (eg, hospital H3, H10, H17), (3) gradual improvement that later stabilised (eg, hospital H1, H4, H14) and (4) failure of adoption (H7 and H5) (figure 4). These varying patterns of adoption, especially after December 2018, do not clearly seem to be related to study participation. Hospitals participating in both new studies, resulting in presence of two additional non-physician clinician research team members, did demonstrate a consistent improvement in pulse oximetry use at admission (figures 3 and 4). However, pulse oximetry adoption at admission was also high in hospitals not participating in any study within the CIN (figures 3 and 4).

### Individual patient data and exploratory modelling

Table 2 provides the characteristics of the children admitted to these hospitals, including the level of missingness in each variable. From the study population, 32% had at least one missing variable, with the median number of variables missing per patient of 2 of 30 (IQR: 0–3). None of the individual variables was missing more than 30% of values. In this paediatric admission population, the prevalence of chest indrawing, tachypnoea and grunting is 22.4%, 33% and 10.3%, respectively, and inability to drink (or breastfeed), a danger sign requiring urgent attention, was present in 72.7% of the admissions; 82.9% of all the admissions had at least one of these signs. The median length of stay was 3 days (IQR: 2–6), with a median of one additional comorbid condition at admission (IQR: 0–1). Pneumonia and malaria were the leading, often comorbid, admission diagnoses (online supplemental figure 1).

**Table 2:** Descriptive summary statistics for the variables of the patient population included in the study

Indicator	Levels	Number, n (%)	Missing data, n (%)
Hospital-level factors			
Malaria endemicity zone	High	53 177 (40.1%)	
	Low	79 560 (59.9%)	
Patient-level factors			
Age*	<1 month	3321 (2.5%)	1221 (0.9%)
	1–11 months	38 235 (28.8%)	
	12–59 months	64 628 (48.7%)	
	≥5 years	25 332 (19.1%)	
Female	Present	58 588 (44.1%)	1199 (0.9%)
Case is readmission	Present	13 557 (10.2%)	37 002 (27.9%)
Case is referral to hospital	Present	19 525 (14.7%)	39 879 (30%)
Presence of comorbidity	Present	82 918 (62.5%)	725 (0.5%)
AVPU†=A	Present	113 378 (85.4%)	10 664 (8%)
Slow capillary refill	Present	2231 (1.7%)	21 902 (16.5%)
Pallor	None	95 612 (72%)	10 799 (8.1%)
	Mild/moderate	15 596 (11.7%)	
	Severe	10 730 (8.1%)	
Fever	Present	105 268 (79.3%)	6952 (5.2%)
Cough	Present	64 819 (48.8%)	9237 (7%)
Crackles	Present	22 835 (17.2%)	11 695 (8.8%)
Central cyanosis	Present	974 (0.7%)	9758 (7.4%)
Acidotic breathing	Present	3098 (2.3%)	12 740 (9.6%)
Difficulty breathing	Present	38 825 (29.2%)	10 454 (7.9%)
Grunting	Present	13 701 (10.3%)	12 255 (9.2%)
Tachypnoea‡	Present	43 832 (33%)	31 136 (23.5%)
Indrawing	Present	29 698 (22.4%)	11 419 (8.6%)
Wheezing	Present	7085 (5.3%)	11 034 (8.3%)
Stridor	Present	2719 (2%)	14 121 (10.6%)
Inability to drink	Present	96 455 (72.7%)	14 846 (11.2%)
Weight-for-age Z-score (WAZ)	> −2SD (normal)	74 035 (55.8%)	33 486 (25.2%)
	−2SD to −3SD (low)	12 377 (9.3%)	
	< −3SD (very low)	12 839 (9.7%)	
Pulse oximetry done at admission‡	Present	64 722 (48.8%)	
Oxygen therapy prescribed at admission	Present	11 428 (8.6%)	4275 (3.2%)
Outcome at discharge	Alive	123 845 (93.3%)	499 (0.4%)
	Died	8393 (6.3%)	


*<1 month represents neonates admitted to paediatric ward; no data from the new-born units were included.

†AVPU scale is an acronym for ‘Alert, Verbal, Pain, Unresponsive’ and is a measure of a patient’s level of consciousness ordered by severity from left to right, with ‘A’ being the least severe.

‡Symptom is derived and calculated as present where either (1) respiratory rate >59 and age <1 month, (2) respiratory rate >49 and age is 1–11 months or (3) respiratory rate is >39 and age is 12 months or older.

§If missing, it was assumed that it was not done. 5525 admission cases reported having done it but did not have the SpO_2_ value on the paper record.

Stridor, wheezing, acidotic breathing and cyanosis have relatively low recorded prevalence of 2%, 5.3%, 2.3% and 0.7%, respectively and were not included in our previous models.^10^ Whether a child is a readmission or referral from another health facility are also newly added data with higher prevalence at 10.2% and 14.7% of the patient population, respectively^10^ (table 2).

### Summary of the growth curve model results

Table 3 provides the results of the growth curve models, where adoption rates between hospitals were adjusted for malaria endemicity, participation in research studies, participants’ demographic characteristics and clinical signs and symptoms at admission. These results are from analysis using 35 imputed datasets (32% of the cohort had at least one missing variable).

**Table 3:** Growth curve model of pulse oximetry adoption and orders for oxygen use at admission

Predictors	Pulse oximetry done?			Oxygen prescribed?		
	OR	95% CI	P value	OR	95% CI	P value
(Intercept)*	0.03	0.01 to 0.17	<0.001	0.01	0.01 to 0.02	<0.001
Hospital factors						
Malaria endemicity zone: high (ref: low)	1.62	0.1 to 25.04	0.729	1.55	0.9 to 2.67	0.112
Time (months in CIN)	1.11	1.05 to 1.18	<0.001	1.02	1 to 1.03	0.045
Patient-level factors						
Ref: patient in any study (No)†						
Patient in any study (prestudy period)‡	2.79	2.58 to 3.02	<0.001	1.02	0.9 to 1.15	0.800
Patient in any study (Yes)	1.46	1.35 to 1.58	<0.001	1.02	0.91 to 1.15	0.715
Referral: Yes (ref: No)	1.05	1.01 to 1.09	0.025	1.19	1.12 to 1.26	<0.001
Readmission: Yes (ref: No)	1.01	0.97 to 1.05	0.732	1.07	1 to 1.15	0.052
Is a weekend admission: Yes (ref: No)	0.98	0.95 to 1.01	0.144	1.07	1.02 to 1.13	0.009
PAR used: Yes (ref: No)	1.99	1.79 to 2.21	<0.001	1.23	1.02 to 1.48	0.031
PAR used * time (months in CIN)	1.01	1 to 1.01	<0.001	1	1 to 1.01	0.562
Ref: age (>59 months)						
Age (<1 month)	0.46	0.41 to 0.51	<0.001	1.96	1.62 to 2.35	<0.001
Age (1–11 months)	1.08	1.03 to 1.13	<0.001	1.43	1.32 to 1.54	<0.001
Age (12–59 months)	1.09	1.05 to 1.13	<0.001	1.08	1 to 1.16	0.054
Female: Yes (ref: No)	1	0.97 to 1.02	0.723	1.02	0.97 to 1.06	0.424
Fever: Yes (Ref: No)	1.18	1.14 to 1.23	<0.001	0.78	0.73 to 0.83	<0.001
Cough: Yes (Ref: No)	1.04	1.01 to 1.08	0.008	1.36	1.29 to 1.44	<0.001
Difficulty breathing: Yes (ref: No)	1.06	1.02 to 1.1	0.002	2.79	2.64 to 2.94	<0.001
Stridor: Yes (ref: No)	1.05	0.95 to 1.16	0.325	1.14	1.02 to 1.27	0.017
Cyanosis: Yes (ref: No)	0.77	0.65 to 0.92	0.004	1.5	1.27 to 1.77	<0.001
Indrawing: Yes (ref: No)	1.25	1.2 to 1.31	<0.001	2.36	2.23 to 2.49	<0.001
Grunting: Yes (ref: No)	0.88	0.84 to 0.93	<0.001	1.9	1.8 to 2	<0.001
Acidotic breathing: Yes (ref: No)	0.89	0.8 to 0.98	0.018	1.34	1.22 to 1.47	<0.001
Wheezing: Yes (ref: No)	1.05	0.98 to 1.12	0.167	1.13	1.06 to 1.21	0.001
Crackles: Yes (ref: No)	1.06	1.01 to 1.11	0.010	1.25	1.19 to 1.32	<0.001
Tachypnoea: Yes (ref: No)	0.96	0.93 to 0.99	0.022	1.59	1.51 to 1.68	<0.001
Alert (AVPU<A): No (ref: Yes)	0.96	0.9 to 1.03	0.238	1.95	1.81 to 2.1	<0.001
Inability to drink: Yes (ref: No)	1.04	1 to 1.08	0.043	0.8	0.76 to 0.85	<0.001
Ref: pallor (none)						
Pallor (mild/moderate)	0.93	0.89 to 0.97	0.002	1.1	1.02 to 1.17	0.008
Pallor (severe)	0.94	0.89 to 0.99	0.024	1.3	1.19 to 1.42	<0.001
Ref: WAZ (normal)						
WAZ (low)	0.97	0.92 to 1.01	0.135	0.98	0.91 to 1.05	0.551
WAZ (very low)	0.94	0.9 to 0.99	0.020	0.93	0.87 to 1	0.059
Slow capillary refill: Yes (ref: No)	0.88	0.78 to 1	0.051	0.97	0.83 to 1.13	0.693
Ref: hypoxaemia (unknown)						
Hypoxaemia (No)				0.59	0.56 to 0.62	<0.001
Hypoxaemia (Yes)				1.92	1.77 to 2.08	<0.001

*Represents the average odds that a patient will have pulse oximetry is done (or oxygen therapy is prescribed) at admission in this sample when all the predictors are set to their reference levels (eg, ‘No’) at the first month of being a member of the Clinical Information Network (CIN).

†'*Patient is in any study’* point estimates and CIs for the ‘*pulse oximetry done?’* outcome do not overlap with ones from complete case analysis (online supplemental table 4). From the complete case sensitivity analysis, there is a material difference in the estimated association of involvement in any research study variable with the pulse oximetry use at admission outcome (table 3, online supplemental table 4); while the direction of association is the same (positive), the CIs do not overlap. With the age variable, the direction of the association is inverted between the complete case and multiple imputation analyses, and children aged <1 month admitted to the paediatric ward are completely missing in the complete case analysis. These material difference might be attributable to the fact that complete case analysis and our multiple imputation approach make different assumptions of missingness mechanism in the data (ie, missing completely at random mechanism—which is potentially more biased, and a missing at random mechanism, respectively).

‡'*Patient is in any study (Pre-study period)’* reflects the time leading up to any research study starting when no study was being conducted, that is, February 2014–November 2018.

AVPU, Alert, Verbal, Pain, Unresponsive; WAZ, Weight-for-age Z-score.

Associations of the hospital and clinical features with the outcomes from table 3 are time-invariant (ie, independent of the effect of time in months in CIN), except for the interaction term of PAR use with time. From online supplemental table 4 and figure 4, the models’ high intraclass correlation coefficient (ICC) is indicative that hospital factors play a bigger role in the adoption of pulse oximetry over time compared with individual patient-level factors. The contribution of hospital level factors is less pronounced for oxygen prescription at admission. The relatively high ICC is not unexpected in longitudinal models with measurements clustered by hospital and given the varying patterns of adoption seen in figure 4.

#### Association with the use of pulse oximetry at admission

Over the entire study period and across all hospitals pulse oximetry use at admission improved as time participating in the CIN increased (month-to-month improvement OR: 1.11, 95% CI 1.05 to 1.18). This was likely driven by noticeable improvement in 10 hospitals and in 5 of these specifically there were substantive positive changes in adoption of pulse oximetry on a month-to-month basis; (online supplemental figure 2). Those improving most tended to have very low use of oximetry at the time they joined the CIN (online supplemental figure 2). This improvement was seen in tandem with the improvement in PAR use which might be expected to encourage better assessment and potentially, therefore, aid clinical decision-making.

From table 3, patients admitted with signs of fever, cough, difficulty breathing, chest indrawing, crackles and inability to drink had odds statistically greater than one of having pulse oximetry measurement taken at admission over time than those without these signs. With a larger dataset, the inclusion of new variables and adjusting for the time in the CIN, our findings conflict with previous findings on the significance of tachypnoea, altered consciousness (AVPU <A), and inability to drink as signs associated with the use of pulse oximetry at admission with a reversal in association (eg, tachypnoea, inability to drink) or no statistically appreciable association (eg, AVPU <A).^10^ Grunting and acidotic breathing symptoms, likely to be associated with malaria and anaemia (online supplemental figure 1), were statistically, not significantly associated with pulse oximetry use at admission (table 3). Cyanosis, grunting and acidotic breathing—which were previously not reported^10^—were significantly associated with the odds of pulse oximetry done at admission being lower than one. Our findings also highlight that referral into the hospital is positively associated with the use of pulse oximeters at admission (OR: 1.05, 95% CI 1.01 to 1.09) (table 3) and that patients admitted to hospitals that were part of an active research study were 49% more likely to have a pulse oximetry measurement taken at admission than in hospitals not participating in any study (table 3). Additional analyses suggest the more clinical signs a child has of respiratory distress the more likely they were to be hypoxaemic (online supplemental figure 3).

#### Associations with oxygen prescription at admission

Admission with any sign of respiratory distress where the pulse oximetry measure is unknown is associated with oxygen prescription at admission (table 3). The specific signs that appear to prompt oxygen prescription, in reducing magnitude of association, include difficulty breathing, indrawing, AVPU <A, grunting and tachypnoea. Admissions with altered consciousness (AVPU <A) had almost twice the odds of oxygen prescription at admission, independent of respiratory signs (table 3). Interestingly, the direction of association with oxygen prescription is inverse to that for use of pulse oximetry for: age of the child, signs of cyanosis, grunting, acidotic breathing, AVPU <A, pallor, tachypnoea, fever and inability to drink.

With the inclusion of new variables and adjusting for the time in CIN, our findings affirm previous analyses but show the magnitude of association of cough and difficulty breathing with oxygen prescription at admission to be higher than previously reported.^10^ Acidotic breathing, stridor and wheezing, clinical signs omitted from the previous study, were shown to be statistically significant and positively associated with the odds of being prescribed oxygen at admission. Grunting and acidotic breathing, likely to be associated with malaria and anaemia diagnoses, were not statistically associated with having pulse oximetry measurement taken at admission but were strongly associated with the prescription of oxygen at admission (table 3).

Our findings also highlight that being a patient referred into the hospital (OR: 1.19, 95% CI 1.12 to 1.26) or admitted during the weekend (OR: 1.07, 95% CI 1.02 to 1.13) is positively associated with oxygen prescription at admission (table 3). Oxygen prescription at admission increased with time as part of CIN, having a statistically significant but clinically small month-to-month change (OR: 1.02, 95% CI 1.00 to 1.03). The improvement in the PAR use (OR: 1.23, 95% CI 1.02 to 1.48) also appears to be aiding clinical decision-making in the prescription of oxygen based on the recording of features of respiratory distress.

## Discussion

Over a period of 7 years, with prolonged strikes precluding data collection in 2017, use of pulse oximetry in a population of admissions where 86% have at least one feature indicating risk of hypoxaemia increased slowly with each month of participation in CIN. Most hospitals at the start of their participation in CIN used pulse oximetry in 25% or fewer children, increasing to 81.5% in all hospitals by the final quarter of 2020. Among the admissions prescribed oxygen in 87%, this was not apparently guided by a pulse oximetry value (SpO2) of <90%, the recommended level for intervention to address hypoxaemia in current pneumonia guidelines.

The highly variable patterns of pulse oximetry adoption across hospitals (figure 4) and high ICCs in our models point to the major impact of hospital-level factors on the use of pulse oximetry. These have been previously described and include equipment supply, maintenance, local leadership and clinical efforts to train and promote oximetry use among others.^10^ CIN provided regular feedback to hospitals including on pulse oximetry use and SpO_2_ values throughout a hospital’s participation in CIN and engaged subsets of hospitals in research projects that resulted in the placement of one or two additional non-physician clinicians (ie, clinical officers, nurses) in some hospitals. In the absence of data from other settings it is hard to determine if the increase in pulse oximetry use is part of a wider secular trend (a phenomenon referred to as the ‘rising tide’^34^) or associated with CIN participation. Whatever the reasons, it is instructive that adoption of pulse oximetry remains incomplete after 7 years even among inpatient populations with severe illness in moderately sized hospitals. This slow pattern of adoption for an established technology that is widely believed to be beneficial has been observed in high-income settings. It suggests we may only realise the proposed benefits of many technologies including diagnostics much more slowly than is often claimed unless much greater attention is paid to strengthening health service delivery at scale.^35 36^

Overall, approximately 9% of patients were prescribed oxygen at admission, with a slight increase in patients with oxygen prescribed with each month in the CIN. Interestingly, in our models the ICCs exploring oxygen use did not suggest strong hospital-level effects, placing more emphasis on patient-level factors. The number of patients with oxygen prescribed who had no SpO_2_ recorded is large; more than twice as many children receiving oxygen had pulse oximetry values 90% or more than values <90% (figure 2) although WHO and Kenyan guidelines recommend targeting oxygen use at children with SpO_2_ <90%.^1^ While the source of discrepancy remains largely unknown, it is unlikely that this reflects diagnoses of shock or severe asthma when oxygen use can be recommended when saturations are higher than 90% as these diagnoses are relatively uncommon in Kenyan hospitals.^1 37^

**Figure 5:**
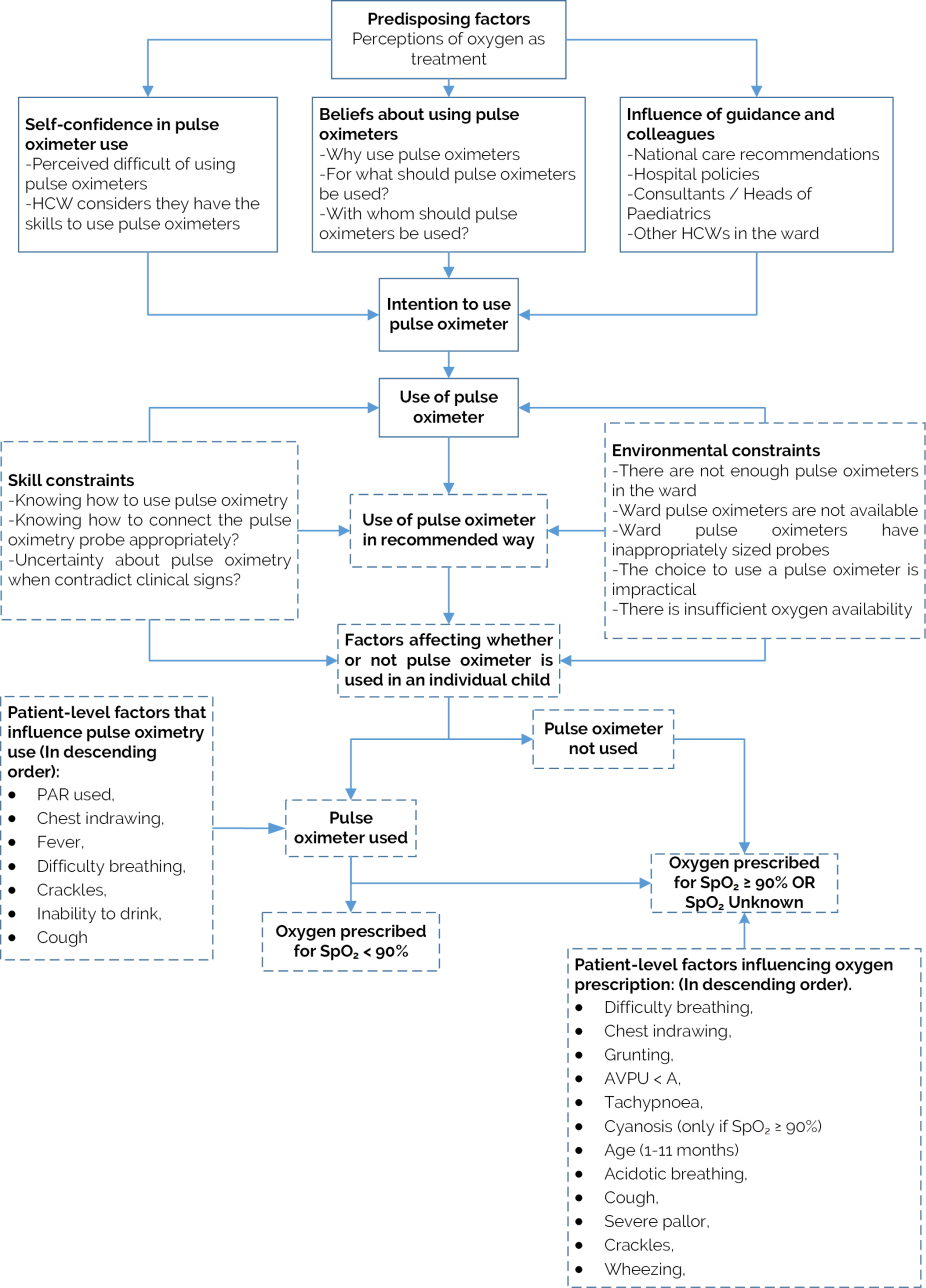
Adapted integrative model of behavioural prediction (Enoch *et al* 10). Dashes indicate components of the model that are modified or added to reflect the findings from our new analyses. AVPU, Alert, Verbal, Pain, Unresponsive; HCW: healthcare worker; PAR, paediatric admission record.

We attempt to integrate our new findings of patient-level factors associated with the use of pulse oximetry and prescription of oxygen at admission with earlier findings that addressed mainly hospital-level factors in figure 5. Lower chest-wall indrawing is strongly and positively associated with pulse oximetry being done at admission and prescription of oxygen when pulse oximetry is not done. We have previously shown an association between indrawing and mortality for pneumonia admissions and others have recently indicated indrawing may be a useful referral criterion from the community or primary care to a hospital in the absence of pulse oximetry.^5 38^ However, randomised trials, and WHO guidance, suggest pneumonia associated with indrawing can be safely treated as an outpatient.^2 37^ Our data do not directly inform the debate on the value of indrawing as screening sign of illness severity but does perhaps suggest it remains an important factor in Kenyan clinicians’ decision-making.

Other signs prompting pulse oximeter use are fever, cough, difficulty breathing, crackles and inability to drink (table 3, figure 5). All respiratory signs are associated with oxygen prescription (table 3, online supplemental table 5, figure 5) and a pulse oximetry value <90% was associated with twice the odds of oxygen prescription compared with those whose oxygen saturation was unknown. Where information from pulse oximetry was not available cyanosis, grunting, acidotic breathing, AVPU <A, pallor and tachypnoea appeared to prompt oxygen use (table 3, figure 5). Several of these signs were not associated with the use of pulse oximetry at admission, perhaps because healthcare providers are taught and then view these signs as indicative of very severe illness (as reported in the literature) requiring intervention with oxygen irrespective of oxygen saturation.^1 37 38^ Interestingly, over time, the use of the standardised PAR was associated with twice the odds of a patient having SpO_2_ measurement and 23% increase in oxygen therapy prescribed at admission, where indicated (table 3). It is possible this effect reflects its role as a prompt to conduct a complete assessment and promote reflection on the use of core interventions recommended in guidelines.

Why infants less than 1 month old have oximetry measured so infrequently remains unknown and warrants more investigation (table 3). It is highly likely that the paediatric wards may not have appropriately sized pulse oximetry probes for the neonates and/or that many of the neonate admissions to the paediatric ward have a diagnosis of sepsis or jaundice which have less clear guidelines on performing routine pulse oximetry on all admissions. Overall, neonates with respiratory problems who mostly tend to be aged closer to time of birth, are sent to newborn wards and thus not meant to be captured in this paediatric admissions dataset (table 2).

It has been well described in many settings that the logical world represented in clinical guidelines is often not replicated in the more complex world of everyday practice.^39–41^ While there remain debates in the scientific world around which oxygen saturation threshold for which condition in which setting should guide the use of oxygen,^5 42 43^ our data remind us that clinicians are often influenced by many other factors in making such decisions.^44^ Clinical decision-making is not straightforward, and it is difficult to capture from routinely collected observational data. Reducing the decision of whether to prescribe or withhold oxygen to a pulse oximetry reading might inadequately reflect the complexities that a clinician encounters at the patient bedside. This has important implications as the presence or prevalence of hypoxaemia, however defined, may be used as proxies for rational use or need for oxygen, respectively. Clinicians’ decision to deviate from pulse oximetry defined hypoxaemia thresholds in determining oxygen use, and the subsequent effects this would have on resource use or patient outcomes, remains largely unaddressed in research.^2^

### Strengths and limitations

The data we present are a rare example of long-term tracking of hospital practice in a routine LMIC setting covering common childhood illnesses (online supplemental table 6). The longitudinal nature of our observations offers important insights but also poses challenges. Major changes may occur in the health sector over long periods, as exemplified by the prolonged strikes in Kenya. Many factors ranging from national and local procurement or equipment donations, to changes in local leadership and hospital’s participation in specific studies might influence the adoption of pulse oximetry and use of oxygen we describe. Furthermore, we have no contemporaneous data from Kenyan settings that do not belong to the CIN for comparison and most other studies exploring the use of pulse oximetry are essentially cross-sectional. We do, however, feel our data are at least a useful illustration of the slow and sometimes erratic process of pulse oximetry adoption in LMIC hospitals that has broader implications for thinking on technology adoption.

Another limitation of this study is the absence of new qualitative data especially exploring why oxygen use is often not linked to pulse oximetry values. However, we have previously shown the importance of local clinical leadership in these settings^45^ and the continuing challenge of promoting guideline adherence in these complex hospital settings where junior clinicians who manage patients rotate frequently and may err on the side of caution to use interventions rather than risk withholding them.^46–48^

Further challenges of the large-scale, longitudinal nature of our work are data quality and analytic methods. The CIN has made efforts to capture high-quality data through continuous training of clerks linked to error checking procedures and regular engagement with hospitals that include monitoring and feedback.^18 28 29^ Nonetheless, imputation procedures that assume a MAR mechanism had to be employed to enable analyses of all eligible case records.^32 49^ We also had to employ a complex modelling framework to explore patient-level associations with our outcomes. These attempted to accommodate and account for between hospital variability, within hospital non-linear trends in the outcomes over time and entry into the CIN at different time points. We tested our assumptions where possible (eg, that the strike year was not associated with major changes in practice). However, while our large dataset enabled us to identify many credible associations specific findings should be interpreted cautiously.

### Conclusions

Over time, there has been a progressive increase in the use of pulse oximeters at admission in the Kenyan hospitals we studied. After 7 years, however, use in this high mortality population where almost 9 out of 10 children have least one sign indicating increased risk of hypoxaemia is not universal and major differences exist between hospitals in the adoption of this technology. Guidelines suggest pulse oximetry should determine which children receive oxygen. Our data suggest this is often not the case even in late 2020 during the COVID-19 pandemic, and that many organisational and clinical factors influence whether oximetry is used at all or whether recommended hypoxaemia definitions are used to guide the use of oxygen. As global efforts gain pace to provide pulse oximeters and oxygen systems our data show it is perhaps naïve to expect they will be adopted and employed in the rational ways imagined in guidelines and policies.

## Supplementary Material

Reviewer comments

Author's manuscript

## Data Availability

Data are available upon reasonable request. Data are available upon reasonable request. The source data are owned by the Kenyan Ministry of Health, County Governments and as the data might be used to deidentify hospitals the study authors are not permitted to share the source data directly. Users who wish to reuse the source data can make a request initially through the KEMRI-Wellcome Trust Research Programme data governance committee. This committee will supply contact information for the KEMRI Scientific and Ethical Review unit, County Governments, and individual hospitals as appropriate. The KEMRI-Wellcome Trust Research data governance committee can be contacted on: dgc@kemri-wellcome.org.

## References

[1] World Health Organization. Manual on use of oxygen therapy in children. World Health Organization, 2016.

[2] Enoch AJ, English M, Shepperd S. Does pulse oximeter use impact health outcomes? A systematic review. Arch Dis Child 2016;101:694–700. 10.1136/archdischild-2015-309638 26699537 PMC4975806

[3] Jouffroy R, Jost D, Prunet B. Prehospital pulse oximetry: a red flag for early detection of silent hypoxemia in COVID-19 patients. Crit Care 2020;24:1–2. 10.1186/s13054-020-03036-9 32513249 PMC7278215

[4] Starr N, Rebollo D, Asemu YM, et al. Pulse oximetry in low-resource settings during the COVID-19 pandemic. Lancet Glob Health 2020;8:e1121–2. 10.1016/S2214-109X(20)30287-4 32628910 PMC7333981

[5] Colbourn T, King C, Beard J, et al. Predictive value of pulse oximetry for mortality in infants and children presenting to primary care with clinical pneumonia in rural Malawi: a data linkage study. PLoS Med 2020;17:e1003300. 10.1371/journal.pmed.1003300 33095763 PMC7584207

[6] Orimadegun A, Ogunbosi B, Orimadegun B. Hypoxemia predicts death from severe falciparum malaria among children under 5 years of age in Nigeria: the need for pulse oximetry in case management. Afr Health Sci 2014;14:397–407. 10.4314/ahs.v14i2.16 25320590 PMC4196412

[7] Lozano J. Epidemiology of hypoxaemia in children with acute lower respiratory infection [oxygen therapy in children]. Int J Tub Lung Dis 2001;5:496–504. 11409574

[8] Liu L, Oza S, Hogan D, et al. Global, regional, and national causes of under-5 mortality in 2000–15: an updated systematic analysis with implications for the Sustainable Development Goals. Lancet 2016;388:3027–35. 10.1016/S0140-6736(16)31593-8 27839855 PMC5161777

[9] Gray AZ, Morpeth M, Duke T, et al. Improved oxygen systems in district hospitals in Lao PDR: a prospective field trial of the impact on outcomes for childhood pneumonia and equipment sustainability. BMJ Paediatr Open 2017;1:e000083. 10.1136/bmjpo-2017-000083 PMC5862216 29637121

[10] Enoch AJ, English M, et al, Clinical Information Network. Variability in the use of pulse oximeters with children in Kenyan hospitals: a mixed-methods analysis. PLoS Med 2019;16:e1002987. 10.1371/journal.pmed.1002987 31891572 PMC6938307

[11] Graham H, Tosif S, Gray A, et al. Providing oxygen to children in hospitals: a realist review. Bull World Health Organ 2017;95:288–302. 10.2471/BLT.16.186676 28479624 PMC5407252

[12] Irimu G, Ogero M, Mbevi G, et al. Tackling health professionals’ strikes: an essential part of health system strengthening in Kenya. BMJ Glob Health 2018;3:e001136. 10.1136/bmjgh-2018-001136 PMC6278918 30588346

[13] García-Romero A, Escribano Álvaro, Tribó JA. The impact of health research on length of stay in Spanish public hospitals. Res Policy 2017;46:591–604. 10.1016/j.respol.2017.01.006

[14] Lichten CA, Marsden G, Pollitt A, et al. Does a biomedical research centre affect patient care in local hospitals? Health Res Policy Syst 2017;15:2. 10.1186/s12961-016-0163-7 28109282 PMC5251230

[15] Gathara D, Malla L, Ayieko P, et al. Variation in and risk factors for paediatric inpatient all-cause mortality in a low income setting: data from an emerging clinical information network. BMC Pediatr 2017;17:99. 10.1186/s12887-017-0850-8 28381208 PMC5382487

[16] von Elm E, Altman DG, Egger M, et al. The strengthening the reporting of observational studies in epidemiology (STROBE) statement: guidelines for reporting observational studies. Ann Intern Med 2007;147:573–7. 10.7326/0003-4819-147-8-200710160-00010 17938396

[17] English M, Irimu G, Agweyu A, et al. Building learning health systems to accelerate research and improve outcomes of clinical care in low- and middle-income countries. PLoS Med 2016;13:e1001991. 10.1371/journal.pmed.1001991 27070913 PMC4829240

[18] Irimu G, Ogero M, Mbevi G, et al. Approaching quality improvement at scale: a learning health system approach in Kenya. Arch Dis Child 2018;103:archdischild-2017-314348. 10.1136/archdischild-2017-314348 PMC6278651 29514814

[19] English M, Irimu G, Akech S, et al. Employing learning health system principles to advance research on severe neonatal and paediatric illness in Kenya. BMJ Glob Health 2021;6:e005300. 10.1136/bmjgh-2021-005300 PMC7993294 33758014

[20] English M, Nzinga J, Irimu G, et al. Programme theory and linked intervention strategy for large-scale change to improve hospital care in a low and middle-income country - A Study Pre-Protocol. Wellcome Open Res 2020;5:265. 10.12688/wellcomeopenres.16379.2 33274301 PMC7684682

[21] Ayieko P, Ogero M, Makone B, et al. Characteristics of admissions and variations in the use of basic investigations, treatments and outcomes in Kenyan hospitals within a new clinical information network. Arch Dis Child 2016;101:223–9. 10.1136/archdischild-2015-309269 26662925 PMC4789757

[22] Ministry of Health. Kenya service availability and readiness assessment mapping (SARAM). Kenya: Government of Kenya Nairobi, 2014.

[23] Gachau S, Ayieko P, Gathara D, et al. Does audit and feedback improve the adoption of recommended practices? Evidence from a longitudinal observational study of an emerging clinical network in Kenya. BMJ Glob Health 2017;2:e000468. 10.1136/bmjgh-2017-000468 PMC5663259 29104769

[24] Mwakyusa S, Wamae A, Wasunna A, et al. Implementation of a structured paediatric admission record for district hospitals in Kenya--results of a pilot study. BMC Int Health Hum Rights 2006;6:9. 10.1186/1472-698X-6-9 16857044 PMC1555611

[25] English M, Wamae A, Nyamai R, et al. Implementing locally appropriate guidelines and training to improve care of serious illness in Kenyan hospitals: a story of scaling-up (and down and left and right). Arch Dis Child 2011;96:285–90. 10.1136/adc.2010.189126 21220265 PMC3039658

[26] Ministry of Health. Basic paediatric protocols. 4th edn, 2016. http://guidelines.health.go.ke/#/category/27/266/meta

[27] Health Services Unit KEMRI–Wellcome Trust Research Programme. Paediatric tools & hospital forms 2016, 2016. Available: http://www.kenyapaediatric.org/idoc/index.php/en/etat/instructors/paeditric-tools-a-hospital-forms.html

[28] Tuti T, Bitok M, Paton C, et al. Innovating to enhance clinical data management using non-commercial and open source solutions across a multi-center network supporting inpatient pediatric care and research in Kenya. J Am Med Inform Assoc 2016;23:184–92. 10.1093/jamia/ocv028 26063746 PMC4681113

[29] Tuti T, Bitok M, Malla L, et al. Improving documentation of clinical care within a clinical information network: an essential initial step in efforts to understand and improve care in Kenyan hospitals. BMJ Glob Health 2016;1:e000028. 10.1136/bmjgh-2016-000028 PMC4934599 27398232

[30] Curran PJ, Obeidat K, Losardo D. Twelve frequently asked questions about growth curve modeling. J Cogn Dev 2010;11:121–36. 10.1080/15248371003699969 21743795 PMC3131138

[31] Kristoffer M. Using R and lme/lmer to fit different two- and three-level longitudinal models, 2015. Available: https://rpsychologist.com/r-guide-longitudinal-lme-lmer

[32] Buuren Sv, Groothuis-Oudshoorn K. mice: multivariate imputation by chained equations in R. J Stat Softw 2010:1–68.

[33] Gachau S, Quartagno M, Njagi EN, et al. Handling missing data in modelling quality of clinician-prescribed routine care: sensitivity analysis of departure from missing at random assumption. Stat Methods Med Res 2020;29:3076–92. 10.1177/0962280220918279 32390503 PMC7116368

[34] Chen Y-F, Hemming K, Stevens AJ, et al. Secular trends and evaluation of complex interventions: the rising tide phenomenon. BMJ Qual Saf 2016;25:303–10. 10.1136/bmjqs-2015-004372 PMC4853562 26442789

[35] Oliwa JN, Gathara D, Ogero M, et al. Diagnostic practices and estimated burden of tuberculosis among children admitted to 13 government hospitals in Kenya: an analysis of two years’ routine clinical data. PLoS One 2019;14:e0221145. 10.1371/journal.pone.0221145 31483793 PMC6726144

[36] Graham HR, Bakare AA, Gray A, et al. Adoption of paediatric and neonatal pulse oximetry by 12 hospitals in Nigeria: a mixed-methods realist evaluation. BMJ Glob Health 2018;3:e000812. 10.1136/bmjgh-2018-000812 PMC6035503 29989086

[37] World Health Organization. Pocket book of hospital care for children: guidelines for the management of common childhood illnesses. World Health Organization, 2013. 24006557

[38] Agweyu A, Lilford RJ, English M, et al. Appropriateness of clinical severity classification of new WHO childhood pneumonia guidance: a multi-hospital, retrospective, cohort study. Lancet Glob Health 2018;6:e74–83. 10.1016/S2214-109X(17)30448-5 29241618 PMC5732316

[39] Gabbay J, le May A. Mindlines: making sense of evidence in practice. Br J Gen Pract 2016;66:402–3. 10.3399/bjgp16X686221 27481961 PMC4979949

[40] Murray E, Treweek S, Pope C, et al. Normalisation process theory: a framework for developing, evaluating and implementing complex interventions. BMC Med 2010;8:1–11. 10.1186/1741-7015-8-63 20961442 PMC2978112

[41] Thompson DS, Fazio X, Kustra E, et al. Scoping review of complexity theory in health services research. BMC Health Serv Res 2016;16:1–16. 10.1186/s12913-016-1343-4 26968157 PMC4788824

[42] Serpa Neto A, Checkley W, Sivakorn C, et al. Pragmatic recommendations for the management of acute respiratory failure and mechanical ventilation in patients with COVID-19 in low- and middle-income countries. Am J Trop Med Hyg 2021:tpmd200796. 10.4269/ajtmh.20-0796 33534774 PMC7957237

[43] Maitland K, Kiguli S, Opoka RO, et al. Children’s Oxygen Administration Strategies Trial (COAST): A randomised controlled trial of high flow versus oxygen versus control in African children with severe pneumonia. Wellcome Open Res 2017;2:100. 10.12688/wellcomeopenres.12747.1 29383331 PMC5771148

[44] WHO, lifebox, ALIMA. Using pulse oximetry for decision support for COVID-19, 2020. Available: https://www.lifebox.org/wp-content/uploads/2020/04/Pulse-Oximetry-for-COVID-19-Decision-Tool-Lifebox-FINAL1.pdf [Accessed 11th Apr 2020].

[45] Nzinga J, McGivern G, English M. Examining clinical leadership in Kenyan public hospitals through the distributed leadership lens. Health Policy Plan 2018;33:ii27–34. 10.1093/heapol/czx167 30053035 PMC6037084

[46] Maina M, Mwaniki P, Odira E, et al. Antibiotic use in Kenyan public hospitals: prevalence, appropriateness and link to guideline availability. Int J Infect Dis 2020;99:10–18. 10.1016/j.ijid.2020.07.084 32781162 PMC7562818

[47] McKnight J, Maina M, Zosi M, et al. Evaluating hospital performance in antibiotic stewardship to guide action at national and local levels in a lower-middle income setting. Glob Health Action 2019;12:1761657. 10.1080/16549716.2020.1761657 32588784 PMC7782734

[48] Ogero M, Akech S, Malla L, et al. Examining which clinicians provide admission hospital care in a high mortality setting and their adherence to guidelines: an observational study in 13 hospitals. Arch Dis Child 2020;105:648–54. 10.1136/archdischild-2019-317256 32169853 PMC7361020

[49] Sperrin M, Martin GP. Multiple imputation with missing indicators as proxies for unmeasured variables: simulation study. BMC Med Res Methodol 2020;20:1–11. 10.1186/s12874-020-01068-x PMC7346454 32640992

