# Supplementary material for: Pulse oximetry adoption and oxygen orders at paediatric admission over 7 years in Kenya: a multihospital retrospective cohort study
Source: BMJ Open. 2021 Sep 7;11(9):e050995. doi: 10.1136/bmjopen-2021-050995 (PMC8424839; doi:10.1136/bmjopen-2021-050995)
Supplement: Supplementary data [file bmjopen-2021-050995supp001.pdf]

Supplementary Material

Supplementary Table 1: Number of patients included into the study per hospital

Hospital	n (%)	Pulse oximeters donated to the hospital by CIN	Current number of pulse oximeters at the paediatric ward	Current status of pulse oximeters*
**H1**	6065 (4.6%)	1	1	All functioning well
**H2**	11636 (8.8%)	1	2	All functioning well
**H3**	9584 (7.2%)	2	2	All functioning well
**H4**	1841 (1.4%)	1	1	All functioning well
**H5**	4021 (3%)	0	0	All oximeters in the neonatal ward
**H6**	13107 (9.9%)	2	2	All functioning well
**H7****	4164 (3.1%)	N/A	N/A	N/A
**H8**	8983 (6.8%)	0	2	Available intermittently. Waiting for supplier to provide longer-time solution
**H9**	9343 (7%)	2	1	One is faulty returned to supplier for repair
**H10**	8764 (6.6%)	2	2	All functioning well
**H11**	11538 (8.7%)	2	2	All functioning well
**H12**	6946 (5.2%)	2	1	One is faulty returned to supplier for repair
**H13**	10055 (7.6%)	1	3	All functioning well
**H14**	8494 (6.4%)	1	0	Stolen. Hospital following up
**H15**	919 (0.7%)	1	1	All functioning well
**H16**	1257 (0.9%)	2	1	One is faulty returned to supplier for repair
**H17**	9196 (6.9%)	0	17	All functional
**H18**	6824 (5.1%)	0	1	Functional. Shared with the Newborn ward.
**Note:**				

**We do not know the brands or specific types; majority of pulse oximeter machines will be portable devices based on authors' experiences of multiple visits to these facilities over this period.*

***Hospital exited from the CIN; Current status of pulse oximetry is therefore not available.*

Supplementary Table 2: Hospital participation in various studies by cluster

Group*	Study	Hospitals
**1**	None	H14
**1**	RTS, S	H18
**1**	RTS, S + SEARCH	H2, H6
**1**	SEARCH	H9, H10, H11, H12, H13
**2**	None	H7, H8, H17
**2**	SEARCH	H3
**3**	RTS, S	H4, H5
**3**	RTS, S + SEARCH	H1
**3**	SEARCH	H16
**3**	None	H15

Note: *The group represents the collection of hospitals in CIN based on joining date. The joining date for Groups 1, 2, and 3, was September 2013, February 2014, from November 2018 respectively. Group 3 hospitals were recruited to aid in increasing hospitals involved in RTS, S and SEARCH studies.

Supplementary Table 3: Discontinuity check due to strike period

(Outcome: Adoption rate per month)

Hospital*	Term**	Estimate***	std.error	statistic	p.value
**H11**	Post Strike Period:I(Time)	2.4660123	0.4525087	5.4496458	**0.012**
**H13**	Post Strike Period:I(Time)	-0.8160995	0.3053929	-2.6722938	0.076
**H10**	Post Strike Period:I(Time)	1.3733025	0.5193376	2.6443346	0.077
**H14**	Post Strike Period:I(Time)	0.2537052	0.1188514	2.1346419	0.122
**H8**	Post Strike Period:I(Time)	-0.9380901	0.4765890	-1.9683421	0.144
**H3**	Post Strike Period:I(Time)	-0.8265731	0.4394489	-1.8809311	0.157
**H18**	Post Strike Period:I(Time)	0.3832935	0.2233908	1.7157982	0.185
**H12**	Post Strike Period:I(Time)	1.2696169	1.0149519	1.2509134	0.300

Supplementary Table 3: Discontinuity check due to strike period (Outcome: Adoption rate per month)					
**Hospital***	**Term****	**Estimate*****	**std.error**	**statistic**	**p.value**
**H6**	Post Strike Period:I(Time)	-0.5999279	0.5146869	-1.1656172	0.328
**H2**	Post Strike Period:I(Time)	-0.3290568	0.2865754	-1.1482383	0.334
**H9**	Post Strike Period:I(Time)	-0.7842015	0.9577486	-0.8187968	0.473
**H17**	Post Strike Period:I(Time)	-0.0119919	0.7642561	-0.0156909	0.988

Note:

*Only hospitals recruited into CIN before the strike

**Time is in months, referencing 3 months before, and 3 months after the strike.

***Estimate is in logit units.

Supplementary Table 4: Complete Case Analysis						
	**Pulse Oximetry Done? (n = 42776)**			**Oxygen Prescribed (n = 42772)**		
**Predictors**	**OR**	**95% CI**	**p-value**	**OR**	**95% CI**	**p-value**
(Intercept)	0.33	0.05 – 2.02	0.231	0.12	0.05 – 0.28	<0.001
**Hospital-level factors**						
Malaria Endemicity Zone [High]	1.03	0.31 – 3.36	0.963	1.02	0.58 – 1.78	0.947
Time (in months)	1.06	1.00 – 1.11	0.033	0.98	0.95 – 1.00	0.076
**Patient-level factors**						
Ref: Patient in any study [No]						
Patient in any study [Pre-study period]‡	1.47	1.30 – 1.67	<0.001	0.9	0.75 – 1.07	0.229
Patient in any study [Yes]	1.15	1.00 – 1.32	0.045	0.99	0.84 – 1.17	0.949
Referral: Yes (Ref: No)	1.05	0.98 – 1.12	0.143	1.24	1.14 – 1.35	<0.001
Readmission: Yes (Ref: No)	1.05	0.97 – 1.13	0.207	0.99	0.89 – 1.11	0.93
Is a weekend admission: Yes (Ref: No)	0.99	0.94 – 1.05	0.761	1.09	1.00 – 1.17	0.04

Supplementary Table 4: Complete Case Analysis						
	Pulse Oximetry Done? (n = 42776)			Oxygen Prescribed (n = 42772)		
**Predictors**	**OR**	**95% CI**	**p-value**	**OR**	**95% CI**	**p-value**
PAR used: Yes (Ref: No)	0.7	0.46 – 1.06	0.09	0.44	0.22 – 0.88	0.02
Time (in months)* PAR used: Yes (Ref: No)	1.01	1.00 – 1.03	0.051	1.03	1.01 – 1.06	0.005
Ref: Age [> 59 months]						
Age [1-11 months]	0.75	0.64 – 0.89	0.001	0.73	0.58 – 0.94	0.013
Age [12-59 months]	0.72	0.61 – 0.84	<0.001	0.56	0.44 – 0.71	<0.001
Female: Yes (Ref: No)	1.03	0.98 – 1.08	0.229	1.03	0.96 – 1.10	0.412
Fever: Yes (Ref: No)	1.03	0.97 – 1.11	0.324	0.81	0.74 – 0.89	<0.001
Cough: Yes (Ref: No)	0.99	0.94 – 1.04	0.626	1.3	1.19 – 1.41	<0.001
Difficulty Breathing: Yes (Ref: No)	1.09	1.02 – 1.15	0.007	2.54	2.34 – 2.76	<0.001
Stridor: Yes (Ref: No)	1.01	0.86 – 1.19	0.926	0.97	0.82 – 1.15	0.739
Cyanosis: Yes (Ref: No)	0.91	0.69 – 1.20	0.499	1.62	1.25 – 2.10	<0.001
Indrawing: Yes (Ref: No)	1.38	1.29 – 1.48	<0.001	2.12	1.95 – 2.31	<0.001
Grunting: Yes (Ref: No)	0.88	0.81 – 0.95	0.001	1.93	1.78 – 2.08	<0.001
Acidotic Breathing: Yes (Ref: No)	0.9	0.77 – 1.04	0.161	1.31	1.13 – 1.51	<0.001
Wheezing: Yes (Ref: No)	1.08	0.97 – 1.20	0.14	0.98	0.89 – 1.09	0.769
Crackles: Yes (Ref: No)	1.06	0.99 – 1.13	0.115	1.2	1.12 – 1.30	<0.001
Tachypnoea: Yes (Ref: No)	1.09	1.03 – 1.15	0.002	1.64	1.51 – 1.77	<0.001
Alert (AVPU < A): Yes (Ref: No)	0.89	0.79 – 1.00	0.042	1.77	1.57 – 2.01	<0.001
Inability to drink: Yes (Ref: No)	0.96	0.89 – 1.03	0.226	0.76	0.69 – 0.83	<0.001
Ref: Pallor [None]						
Pallor [Mild/Moderate]	1.02	0.94 – 1.12	0.574	1.23	1.10 – 1.37	<0.001
Pallor [Severe]	0.94	0.83 – 1.07	0.336	1.18	0.99 – 1.40	0.067
Ref: WAZ [Normal]						
WAZ [Low]	0.97	0.90 – 1.04	0.388	0.95	0.86 – 1.05	0.327
WAZ [Very Low]	1	0.93 – 1.08	0.97	0.9	0.82 – 0.99	0.033

**Supplementary Table 4: Complete Case Analysis**						
	**Pulse Oximetry Done? (n = 42776)**			**Oxygen Prescribed (n = 42772)**		
**Predictors**	**OR**	**95% CI**	**p-value**	**OR**	**95% CI**	**p-value**
Slow Capillary Refill: Yes (Ref: No)	0.76	0.61 – 0.94	0.013	0.8	0.60 – 1.06	0.119
Ref: Hypoxic [Unknown]						
Hypoxic [No]				0.54	0.50 – 0.59	<0.001
Hypoxic [Yes]				1.71	1.53 – 1.92	<0.001
Random effects						
**σ^2**	3.29			3.29		
**τ_{00}**	12.40 Hospital			0.23 Hospital		
**τ_{11}**	0.01 Hospital.Time			0.00 Hospital.Time		
**ρ_{01}**	-0.96 Hospital			-0.11 Hospital		
**ICC**	0.57			0.23		
**N**	17 Hospital			17 Hospital		
**Marginal R^2 / Conditional R^2**	0.144/ 0.631			0.271 / 0.442		

**Supplementary Table 5: Oxygen prescription for SpO_2 ≥ 90% OR SpO_2 Unknown**						
	**MICE model***			**Complete Case Analysis****		
**Predictors**	**OR**	**95% CI**	**p-value**	**OR**	**95% CI**	**p-value**
(Intercept)	0.04	0.02 - 0.08	<0.001	0.08	0.03 – 0.19	<0.001
**Hospital-level factors**						
Malaria Endemicity Zone [High]	1.61	0.9 - 2.87	0.106	1.12	0.61 – 2.04	0.719
Time (in months)	1.01	1 - 1.02	0.152	0.97	0.95 – 1.00	0.032
**Patient-level factors**						
Ref: Patient in any study [No]						

Supplementary Table 5: Oxygen prescription for $SpO_2 \geq 90\%$ OR SpO_2 Unknown						
	MICE model*			Complete Case Analysis**		
**Predictors**	**OR**	**95% CI**	**p-value**	**OR**	**95% CI**	**p-value**
Patient in any study [Pre-study period]ǂ	0.99	0.86 - 1.12	0.824	0.88	0.73 – 1.06	0.168
Patient in any study [Yes]	0.94	0.83 - 1.07	0.349	0.92	0.78 – 1.10	0.369
Referral: Yes (Ref: No)	1.18	1.11 - 1.26	<0.001	1.28	1.17 – 1.40	<0.001
Readmission: Yes (Ref: No)	1.07	1 - 1.16	0.066	1.01	0.90 – 1.14	0.846
Is a weekend admission: Yes (Ref: No)	1.08	1.02 - 1.14	0.005	1.11	1.02 – 1.21	0.014
PAR used: Yes (Ref: No)	1.18	0.96 - 1.44	0.120	0.46	0.22 – 0.96	0.039
Time (in months)*PAR used: Yes (Ref: No)	1	1 - 1.01	0.560	1.03	1.01 – 1.06	0.013
Ref: Age [> 59 months]						
Age [< 1 month]	1.9	1.56 - 2.3	<0.001			
Age [1-11 months]	1.42	1.31 - 1.54	<0.001	0.79	0.61 – 1.03	0.078
Age [12-59 months]	1.06	0.98 - 1.15	0.163	0.59	0.46 – 0.76	<0.001
Female: Yes (Ref: No)	1.03	0.98 - 1.07	0.288	1.04	0.97 – 1.12	0.256
Fever: Yes (Ref: No)	0.76	0.72 - 0.81	<0.001	0.8	0.72 – 0.89	<0.001
Cough: Yes (Ref: No)	1.36	1.28 - 1.44	<0.001	1.3	1.19 – 1.42	<0.001
Difficulty Breathing: Yes (Ref: No)	2.88	2.72 - 3.05	<0.001	2.66	2.43 – 2.91	<0.001
Stridor: Yes (Ref: No)	1.12	1.01 - 1.26	0.039	0.99	0.83 – 1.17	0.865
Cyanosis: Yes (Ref: No)	1.61	1.36 - 1.91	<0.001	1.94	1.48 – 2.56	<0.001
Indrawing: Yes (Ref: No)	2.48	2.34 - 2.63	<0.001	2.27	2.07 – 2.48	<0.001
Grunting: Yes (Ref: No)	2.03	1.92 - 2.15	<0.001	2.09	1.92 – 2.27	<0.001
Acidotic Breathing: Yes (Ref: No)	1.37	1.24 - 1.51	<0.001	1.38	1.19 – 1.61	<0.001
Wheezing: Yes (Ref: No)	1.17	1.08 - 1.26	<0.001	1.04	0.93 – 1.16	0.542
Crackles: Yes (Ref: No)	1.27	1.2 - 1.34	<0.001	1.21	1.12 – 1.32	<0.001
Tachypnoea: Yes (Ref: No)	1.63	1.54 - 1.72	<0.001	1.7	1.56 – 1.85	<0.001
Alert (AVPU < A): Yes (Ref: No)	2.02	1.86 - 2.18	<0.001	1.83	1.60 – 2.08	<0.001
Inability to drink: Yes (Ref: No)	0.8	0.75 - 0.85	<0.001	0.76	0.69 – 0.84	<0.001

Supplementary Table 5: Oxygen prescription for $SpO_2 \geq 90\%$ OR SpO_2 Unknown						
	MICE model*			Complete Case Analysis**		
**Predictors**	**OR**	**95% CI**	**p-value**	**OR**	**95% CI**	**p-value**
Ref: Pallor [None]						
Pallor [Mild/Moderate]	1.11	1.04 - 1.2	<0.001	1.26	1.12 – 1.41	<0.001
Pallor [Severe]	1.34	1.22 - 1.46	<0.001	1.14	0.95 – 1.38	0.153
Ref: WAZ [Normal]						
WAZ [Low]	1	0.93 - 1.07	0.952	0.97	0.87 – 1.08	0.596
WAZ [Very Low]	0.96	0.89 - 1.03	0.273	0.92	0.83 – 1.02	0.122
Slow Capillary Refill: Yes (Ref: No)	1.03	0.88 - 1.2	0.708	0.87	0.65 – 1.18	0.371

**Note: ***n=123,952; 4275 cases omitted from MICE analysis because of missing outcome (Figure 4)*
****n=40,491*

Supplementary Table 6: Admission diagnoses for patients getting oxygen with pulse oximetry ≥ 90% at admission.

**Diagnosis**	**Documented Cases, N (%)**
Pneumonia	18668 (34.14%)
Malaria	16559 (30.28%)
Dehydration	10641 (19.46%)
Diarrhoea	10592 (19.37%)
Anaemia	8018 (14.66%)
Meningitis	5875 (10.74%)
Malnutrition	5351 (9.78%)
Sickle Cell	2380 (4.35%)
Asthma	1240 (2.27%)
Rickets	1084 (1.98%)
TB	907 (1.6s6%)
Sepsis	708 (1.29%)
HIV	716 (1.31%)

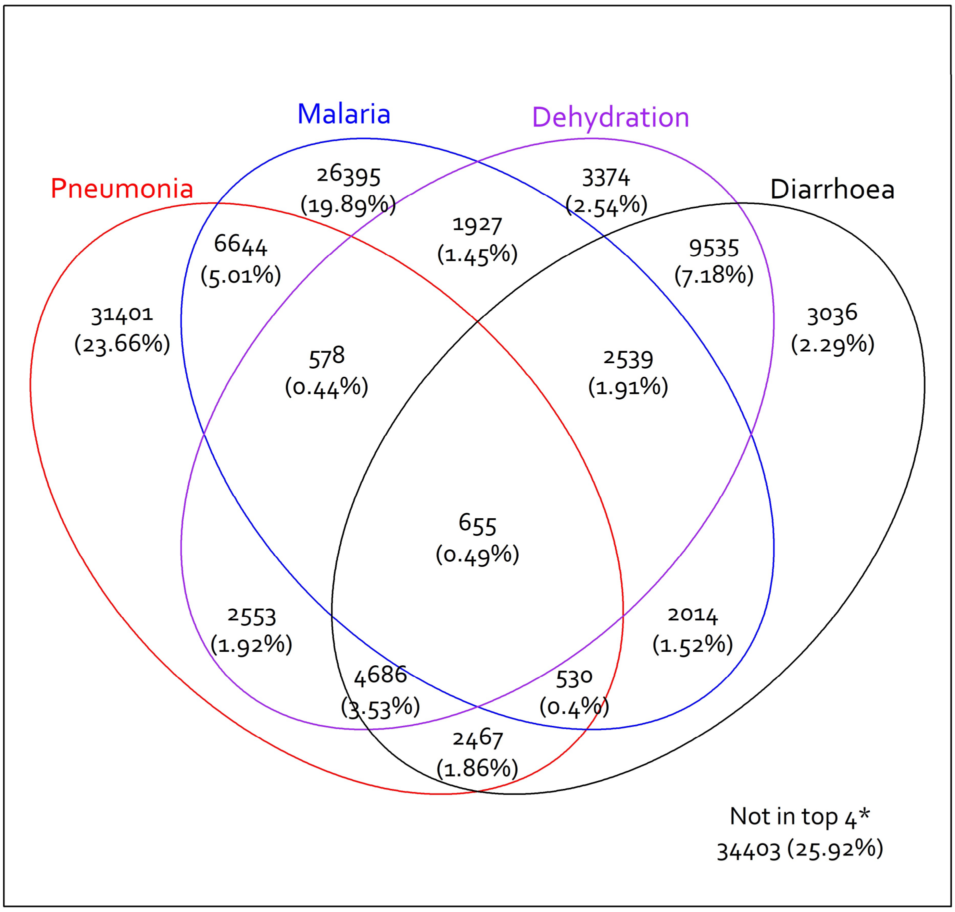

Supplementary Figure 1: Common admission diagnoses in CIN hospitals.

From the linear growth-curve model, the random intercept conveys the hospital's average pulse oximetry adoption rate over the time period in months it has participated in CIN. The random slope conveys the hospital's average monthly increase or decrease in adoption rate over the time period they have been part of the CIN. The baseline for the random intercept and slope in the growth curve model is the overall level of the response which is allowed to vary between hospitals after controlling for covariates.

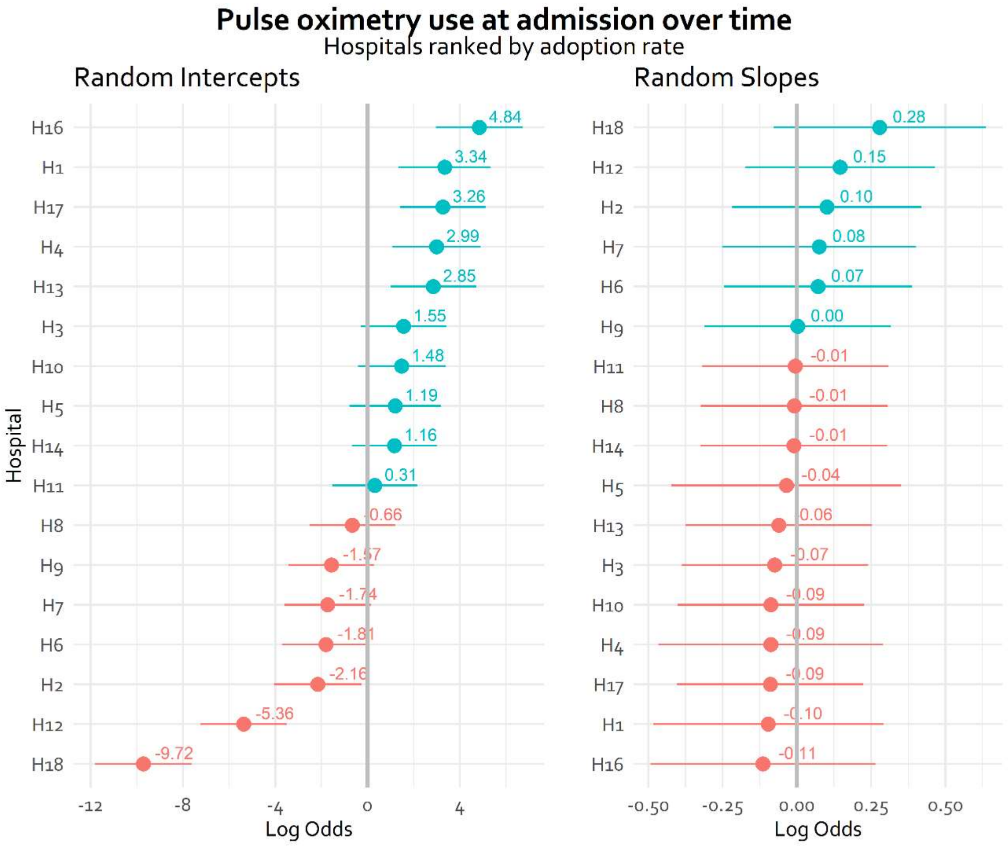

Supplementary Figure 2: Ranking of hospitals pulse oximetry use at admission

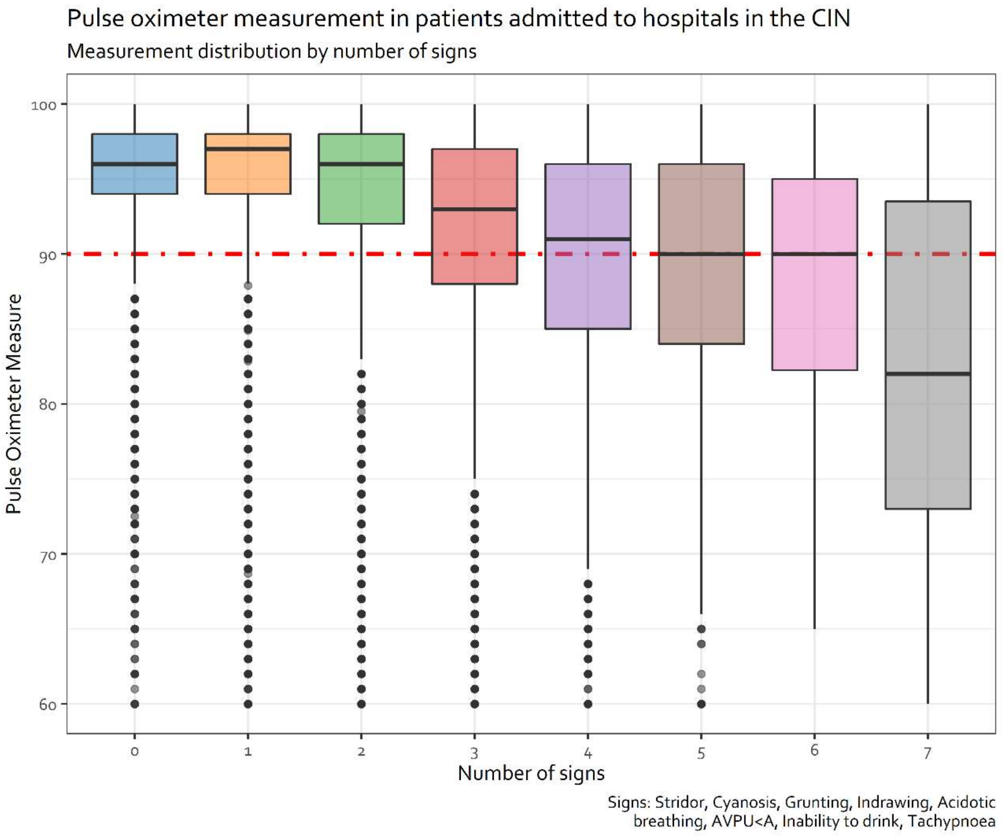

Supplementary Figure 3: Oxygen saturation measure by the number of respiratory distress symptoms
