# Supplementary material for: Pulse oximetry adoption and oxygen orders at paediatric admission over 7 years in Kenya: a multihospital retrospective cohort study
Source: BMJ Open. 2021 Sep 7;11(9):e050995. doi: 10.1136/bmjopen-2021-050995 (PMC8424839; doi:10.1136/bmjopen-2021-050995)
Supplement: Author's manuscript [file bmjopen-2021-050995.draft_revisions.pdf]

BMJ Open is committed to open peer review. As part of this commitment we make the peer review history of every article we publish publicly available.

When an article is published we post the peer reviewers' comments and the authors' responses online. We also post the versions of the paper that were used during peer review. These are the versions that the peer review comments apply to.

The versions of the paper that follow are the versions that were submitted during the peer review process. They are not the versions of record or the final published versions. They should not be cited or distributed as the published version of this manuscript.

BMJ Open is an open access journal and the full, final, typeset and author-corrected version of record of the manuscript is available on our site with no access controls, subscription charges or pay-per-view fees (http://bmjopen.bmj.com).

If you have any questions on BMJ Open's open peer review process please email
info.bmjopen@bmj.com

BMJ Open

Pulse oximetry adoption and oxygen orders at paediatric admission over 7 years in Kenya: A multi-hospital retrospective cohort study

Journal:	*BMJ Open*
Manuscript ID	bmjopen-2021-050995
Article Type:	Original research
Date Submitted by the Author:	08-Mar-2021
Complete List of Authors:	Tuti, Timothy; KEMRI-Wellcome Trust Research Programme Nairobi, Health Services Unit<br>Tuti, Timothy; University of Oxford, Kellogg College; Centre for Geographic Medicine Research Coast, Health Services Unit<br>Aluvaala, Jalemba ; Kenya Medical Research Institute/Wellcome Trust Research Programme, Health Services Unit; University of Nairobi, Department of Paediatrics and Child Health<br>Akech, Samuel; KEMRI-Wellcome Trust Research Programme Nairobi<br>Agweyu, Ambrose; KEMRI-Wellcome Trust Research Programme Nairobi, Health Services Unit<br>Irimu, Grace ; Kenya Medical Research Institute, Health Services Unit; University of Nairobi College of Health Sciences, Department of Paediatrics and Child Health<br>English, Mike; University of Oxford, Nuffield Department of Medicine and Department of Paediatrics; KEMRI-Wellcome Trust Research Programme Nairobi, Health Services Unit
Keywords:	Quality in health care < HEALTH SERVICES ADMINISTRATION & MANAGEMENT, PAEDIATRICS, AUDIT, EPIDEMIOLOGY, Protocols & guidelines < HEALTH SERVICES ADMINISTRATION & MANAGEMENT, Tropical medicine < INFECTIOUS DISEASES

SCHOLARONE™
Manuscripts

I, the Submitting Author has the right to grant and does grant on behalf of all authors of the Work (as defined in the below author licence), an exclusive licence and/or a non-exclusive licence for contributions from authors who are: i) UK Crown employees; ii) where BMJ has agreed a CC-BY licence shall apply, and/or iii) in accordance with the terms applicable for US Federal Government officers or employees acting as part of their official duties; on a worldwide, perpetual, irrevocable, royalty-free basis to BMJ Publishing Group Ltd ("BMJ") its licensees and where the relevant Journal is co-owned by BMJ to the co-owners of the Journal, to publish the Work in this journal and any other BMJ products and to exploit all rights, as set out in our licence.

The Submitting Author accepts and understands that any supply made under these terms is made by BMJ to the Submitting Author unless you are acting as an employee on behalf of your employer or a postgraduate student of an affiliated institution which is paying any applicable article publishing charge ("APC") for Open Access articles. Where the Submitting Author wishes to make the Work available on an Open Access basis (and intends to pay the relevant APC), the terms of reuse of such Open Access shall be governed by a Creative Commons licence – details of these licences and which Creative Commons licence will apply to this Work are set out in our licence referred to above.

Other than as permitted in any relevant BMJ Author's Self Archiving Policies, I confirm this Work has not been accepted for publication elsewhere, is not being considered for publication elsewhere and does not duplicate material already published. I confirm all authors consent to publication of this Work and authorise the granting of this licence.

For peer review only

Pulse oximetry adoption and oxygen orders at paediatric admission over 7 years in Kenya: A multi-hospital retrospective cohort study

Timothy Tuti[1*], Jalemba Aluvaala[1,2], Samuel Akech[1], Ambrose Agweyu[1], Grace Irimu[1,2], Mike English[1,3] on behalf of the Clinical Information Network,

[1] Health Services Unit, KEMRI-Wellcome Trust Research Programme

[2] Department of Paediatrics and Child Health, University of Nairobi, Nairobi, Kenya

[3] Oxford Centre for Global Health Research, University of Oxford, Oxford, UK

*Corresponding Author: P.O. Box 43640 – 00100, Nairobi, Kenya; Email: ttuti@kemri-wellcome.org

For peer review only

Abstract:

Objectives: To characterise adoption and explore specific factors that might influence pulse oximetry and oxygen use in low- and middle-income countries (LMIC) over time; To highlight experiences that may be useful for entities working on programs to improve access to pulse oximetry and oxygen due to COVID-19.

Design: A multi-hospital retrospective cohort study

Settings: All admissions (n=132737) to paediatric wards of 18 purposely selected public hospitals in Kenya that joined a Clinical Information Network (CIN) between March 2014 and December 2020.

Outcomes: Pulse oximetry use and oxygen prescription on admission over time; We explored their association with presenting clinical features while adjusting for hospital factors using growth curve models.

Results: Overall, pulse oximetry was used in 48.8% (64722/132737) of all admission cases. Use rose on average with each month of participation in the CIN (OR: 1.11, 95% CI 1.05–1.18) but patterns of adoption were highly variable across hospitals suggesting important factors at hospital level influence

use of pulse oximetry. Of those with pulse oximetry measurement, 7% (4510 /64722) had hypoxaemia (SpO_2 <90%). Across the same period, 8.6% (11428/132737) had oxygen prescribed but in 87%, pulse oximetry was either not done or the hypoxemia threshold (SpO_2 <90%) was not met. Lower chest-wall indrawing and other respiratory symptoms were associated with pulse oximetry use at admission and were also associated with oxygen prescription in the absence of pulse oximetry or hypoxemia.

Conclusion: The adoption of pulse oximetry recommended in international guidelines for assessing children with severe illness has been slow and erratic, reflecting system and organisational weaknesses. Most oxygen orders at admission seem driven by clinical and situational factors other than the presence of hypoxemia. Programmes aiming to implement pulse oximetry and oxygen systems will likely need a long-term vision to promote adoption, guideline development and adherence and continuously examine impact.

Keywords: Quality Improvement, Health Systems, Paediatric Care, Diagnostics, Evaluation, Hospital

Word Count: 5,120

Strengths and limitations

- The data presented are a rare example of long-term tracking of hospital practice in a routine LMICs setting. They present a useful illustration of the slow and sometimes erratic process of pulse oximetry adoption in LMIC hospitals that has broader implications for thinking on technology adoption.
- We employ a complex modelling framework to explore patient-level associations with outcomes of interest. These accommodate and account for between hospital variability, within hospital non-linear trends in the outcomes over time and entry into the CIN at different time points.
- Many factors ranging from national and local procurement or equipment donations, to changes in local leadership and hospital's participation in specific studies might influence the

For peer review only

For peer review only - http://bmjopen.bmj.com/site/about/guidelines.xhtml

adoption of pulse oximetry and use of oxygen we describe; There is limited data to explore their influence on adoption.

- We have no contemporaneous data from Kenyan settings that do not belong to the CIN for comparison and most other studies exploring the use of pulse oximetry are essentially cross-sectional.

Author summary

Why was this study done?

- Pulse oximetry, an inexpensive diagnostic tool, is more sensitive at detecting hypoxemia than clinical signs alone and is therefore recommended for use in severely ill children as part of their assessment and to guide oxygen therapy if hypoxemia is identified
- Prior work explored the adoption of pulse oximetry in selected hospitals in Kenya over a modest timeframe and results suggested factors at the hospital level were important determinants of use.
- There is a lack of information on the adoption of pulse oximetry in routine hospital settings over extended timeframes and on factors associated with oxygen use.

What did the researchers do and find?

- We used routine paediatric inpatient admission data collected over seven years from a Clinical Information Network in Kenya beginning with 13 and ending with 18 hospitals to explore the adoption of pulse oximetry measurement at admission over time.
- While there was a progressive increase over this period, pulse oximetry was only used in approximately half of the children on admission and individual hospitals had highly variable patterns of adoption.
- Over the same period, approximately 9% of children were prescribed oxygen, however, 85% of oxygen prescriptions did not seem to be informed by a pulse oximetry measurement or

For peer review only - http://bmjopen.bmj.com/site/about/guidelines.xhtml

were in children with an oxygen saturation of ≥90% that is above the threshold considered to indicate hypoxemia and need for oxygen in national and global guidelines for pneumonia.

- Common clinical signs of respiratory distress or severe illness more broadly were associated with pulse oximetry use and were also associated with oxygen prescription in those without a pulse oximetry measurement or in the apparent absence of hypoxemia.

What do these findings mean?

- Our findings suggest that despite being a cheap and widely recommended technology. adoption of pulse oximetry as part of the assessment of severe childhood illness in Kenyan hospitals has been slow and erratic but is improving.
- Even when pulse oximetry is used clinicians' decisions on the prescription of oxygen appear to be influenced by many other factors resulting in apparent non-compliance with current guidance.
- Our data suggest programmes seeking to implement pulse oximetry, improved oxygen systems or other technologies at scale in routine settings in low- and middle-income countries will need to pay special attention to the organisational context and go beyond the simple provision of equipment or support for short-term maintenance, and should include evaluations of impact over sufficient timescales.

For peer review only

Introduction

Background

Compared to clinical signs (e.g. central cyanosis, indrawing), pulse oximetry is felt to be easy to use and posited to be at least 20% more accurate in identifying children at risk of hypoxemia (defined as a blood oxygen saturation of <90%) [1, 2]. As pulse oximetry is an inexpensive tool it is now recommended for guiding the assessment of illness severity and treatment for respiratory illnesses, including increasing emphasis on its role for screening children with respiratory illness in primary care in LMIC [1, 2]. As hypoxemia may be found in all severe childhood illnesses such as sepsis, meningitis,

For peer review only - http://bmjopen.bmj.com/site/about/guidelines.xhtml

severe acute malnutrition and malaria its use is widely recommended as a screening tool in all paediatric admissions as a fifth vital sign. The advent of COVID-19 has further invigorated programmatic efforts to scale up pulse oximetry together with improved oxygen delivery systems [3-9].

Despite being recommended for many years, there is little long-term data on the adoption of pulse oximetry in LMIC, even from hospitals. Data on use during the COVID-19 pandemic are also lacking from such settings as are large studies from routine settings on whether pulse oximeter readings influence the use of oxygen. We and others have described that adoption is undermined by inadequate supply and repair of oximeters and if healthcare workers have insufficient training on when, how, and why to use them and interpret their results [9, 10]. Our earlier work used data from 7 Kenyan hospitals between September 2013 and February 2016. Here we update and extend these analyses to include data from paediatric admissions in a growing network of Kenyan hospitals before and after prolonged national health workers strikes in 2017, including data obtained during the COVID-19 pandemic [11]. The objective of this study was to characterise adoption and explore specific factors that might influence pulse oximetry and oxygen use and highlight experiences that may be useful for entities working on programs to improve access to pulse oximetry and oxygen due to COVID-19[12-14].

Methods

Ethics and Reporting

The reporting of this observational study follows the Strengthening of reporting of observational studies in epidemiology (STROBE) statement [15]. The Scientific and Ethics Review Unit of the Kenya Medical Research Institute (KEMRI) approved the collection of the de-identified data that provides the basis for this study as part of the Clinical Information Network (CIN). The CIN is run in partnership with the Ministry of Health and participating hospitals with aims to improve the quality of routine paediatric hospital data for use in improvement activities, observational and interventional research [16, 17]. Individual consent for access to de-identified patient data was not required.

For peer review only - http://bmjopen.bmj.com/site/about/guidelines.xhtml

Patient and Public Involvement

No patients were involved in the design, conduct, reporting, or dissemination plans of our research except through the KEMRI ethical review process where they have representatives.

Study design and setting

We report a retrospective cohort study of 18 public hospitals in Kenya largely providing first-referral level care purposefully selected to be of at least moderate size and representative of different malaria transmission zones. Hospitals joined the CIN at different calendar time points between 2013 and 2017. The hospitals receive 3 monthly clinical audit and feedback reports on the quality of care they provide for common conditions [18]. Paediatric team leaders (paediatricians and nurses) met face to face once or twice annually until 2019 (before the pandemic) to discuss these reports and how to improve clinical care.

From 2018, participation in more specific research studies was also discussed in meetings with hospitals. This resulted in two studies being initiated in subsets of the CIN hospitals which might have influenced the adoption of pulse oximetry. Hospital participation in these studies was not mutually exclusive: a hospital could be part of one, both or none of the research studies. Not all hospitals recruited in the same study were recruited at the same time. Summary details of these studies are provided in Table 1 with greater detail on which hospitals participated in the studies provided in Supplementary Tables 1 and 2.

Table 1: Description of studies undertaken in CIN hospitals

**Study name**	**Study site selection rationale and period**	**Goal**	**Extra resources provided to hospitals (H) by projects***	**Extra notes**
**RTS, S/AS01 trial: (ClinicalTrials.gov Identifier: NCT03806465)**	Study start: December 2018<br><br>Site Selection: By MoH/WHO, selecting high volume public hospitals in regions getting the vaccine.	The evaluation of the feasibility, safety and impact of the RTS, S/AS01 malaria vaccine in paediatric cases in Kenya	One extra healthcare worker provided per paediatric ward. Extra pulse oximetry equipment provided per ward:	Clinician role: Promote compliance with guidelines for meningitis and malaria including complete assessment and investigations including the performance of lumbar punctures, and consenting participants for storage of cerebrospinal fluid (CSF) storage

For peer review only - http://bmjopen.bmj.com/site/about/guidelines.xhtml

			• One item: H4	
**SEARCH trial (ClinicalTrials.gov Identifier: NCT04041791)**	Started in two phases:<br>• March 2019 Hospitals: H3, H9, H12, H13, H16<br>• June 2019 Hospitals: H1, H2, H6, H10, H11<br><br>Site selection: From the expected size of pneumonia population, the geographical balance of hospitals and support from local hospital teams	Pragmatic factorial individually randomized controlled trial to compare alternative antibiotics and alternative modes of fluid therapy for children with severe pneumonia	One extra healthcare worker provided per participating hospital<br><br>Extra pulse oximetry equipment provided to the hospital:<br>• Two items: H3, H6, H9, H10, H11, H12, H13, H16<br>• One item: H1, H2	Clinician role: Promote compliance with the study protocol, including completing patient assessment, investigations and diagnoses. Ensure appropriate administration of study interventions.<br><br>Recruitment of study participants was suspended on the 8th of April 2020 due to COVID-19 pandemic.<br><br>Adult inpatient surveillance in addition to paediatric surveillance began on the 4th of May 2020.
**Note:**<br>*H15 got one pulse oximeter in June 2020 even though it was not part of any study*<br>** We were not able to track and record introduction of pulse oximeters procured directly by hospitals or other sources, or those personally owned by healthcare workers, nor ability to repair existing pulse oximeters when required.*				

Recording of pulse oximetry values has been included as part of a structured Paediatric Admission Record (PAR) [19] used since 2013. Hospitals joining the CIN agree to provide the PAR themselves and promote its use – the purpose of the PAR is to prompt admitting clinicians to fully assess children and rapidly document their findings [19]. Emergency Triage Assessment and Treatment plus Admission Care (ETAT+) training [20] has been used in Kenya since 2008 and was adapted in 2013 so that pulse oximetry was a recommended part of the assessment in all sick children and especially those with danger signs. Many of the junior and senior medical staff in CIN hospitals would have received ETAT+ training (e.g. as an undergraduate or postgraduate course or as in-service training) [20]. Additionally, the Basic Paediatric Protocols that are widely disseminated have specifically referred to the use of pulse oximetry – if available – for all pneumonia cases since 2016 [21].

For peer review only

For peer review only - http://bmjopen.bmj.com/site/about/guidelines.xhtml

Study participants

All medical admissions to paediatric wards (ages 0 to 13 years), but not those admitted to specific newborn units, to the selected hospitals between February 1st 2014, or the date at which a hospital joined CIN, and December 31st 2020 were eligible for inclusion in this study. We excluded children whose admission or discharge dates were missing or improbable (e.g. discharge date is earlier than admission date), and those whose admission fell within 2017, a period of prolonged health worker strikes that resulted in major disruption to health care delivery [11]. The expectation was that all would have pulse oximetry performed at admission given they were sick enough to warrant inpatient care but amongst these we also report the proportion of children with danger signs comprising any level of altered consciousness or with recorded signs of respiratory distress especially deserving of pulse oximetry [10, 21, 22].

Data sources and management

Methods of collection and cleaning of data in the CIN are reported in detail elsewhere [23]. Clinical data for paediatric admissions to the hospitals within the CIN are captured through PAR forms [22] that are approved by the Ministry of Health. The PAR prompts the clinician with a checklist of fields including patient biodata, clinical assessment, admission and discharge diagnoses, treatments, and to record outcome (survival or death). The CIN supports one data clerk in each hospital to extract data from paper medical records, nursing charts, treatment charts, and available laboratory reports each day after children's discharge into the primary data collection tool developed in Research Electronic Data Capture (REDCap). Automated error checking happens at the point of entry by daily review, every week centrally and both are complemented by regular external data quality assurance reviews [23]. A minimal dataset – which is unsuitable for these pulse oximetry analyses - is collected for (1) all admissions with a burn or a surgical diagnosis to the paediatric ward(s), (2) admissions during major holiday breaks, (3) admissions when the data clerk was on leave, and (4) on a random selection of records in hospitals where the workload is very high. This process is explained in detail elsewhere [23, 24].

For peer review only

For peer review only - http://bmjopen.bmj.com/site/about/guidelines.xhtml

Descriptive analysis

To characterise adoption of pulse oximetry at admission over time we use pooled data from the period February 2014 to November 2018 (excluding 2017) from 13 network hospitals. From December 2018 data were available from 17 hospitals after 5 joined the CIN linked to new studies, and one (H7) exited. To explore patterns in more detail we employed two approaches: i) for the period from December 2018 to December 2020 we plot data pooled for hospital sub-groups depending on which research studies they were taking part in as these might influence pulse oximetry use; ii) We plot adoption within each hospital for the number of months it was part of the network, again excluding 2017 where relevant. This latter approach was used to reveal the extent of variability in adoption at the hospital level.

For the individual patient population, we tabulated and summarised categorical data as proportions, with continuous variables reported as medians (IQR) if they were not normally distributed. From the included paediatric admission patient population data, we report the prevalence of important clinical features such as respiratory distress symptoms (e.g. central cyanosis, indrawing, grunting, and difficulty breathing), circulatory symptoms (e.g. slow capillary refill, less than three seconds), level of consciousness, and whether the patient was being readmitted or referred to the hospital. We also report the prevalence of summary clinical features such as having any sign of respiratory distress and any danger sign and the proportion for whom oxygen was ordered as a treatment at admission.

For peer review only

Statistical methods

To examine pulse oximetry use and oxygen orders we modelled individual patient data. Our key explanatory variable was time as part of (or exposure to) the CIN, computed as the number of months since joining the CIN for a specific hospital. This allowed associations with these outcomes to be explored in hospitals while accounting for clustering by both hospital and time in months since the hospital joined the CIN; the analysis of pulse oximetry use employs a much larger dataset to extend earlier findings [10].

For peer review only - http://bmjopen.bmj.com/site/about/guidelines.xhtml

Predictors included in our models were patients' age and sex, and the record of presence or absence of respiratory signs and symptoms, circulatory symptoms, level of consciousness, whether the patient was being readmitted or referred to the hospital. At the hospital level, we included terms for their specific identity and malaria endemicity zone. Hospital participation in one of the four research study sub-groups was included as a covariable at the patient level to take account of possible effects occurring only after a hospital joined specific studies.

We modelled whether oxygen was prescribed at admission with an interest in whether patients with a recorded pulse oximetry value (SpO_2) <90% at admission would be started on oxygen therapy. Where the pulse oximetry value at admission was missing, based on findings from a previous CIN study, the assumption was that the clinician did not have the information [10]. The analysis therefore explored (1) whether pulse oximetry values (SpO_2 <90%) were associated with oxygen prescription and (2) in the absence of a pulse oximetry value <90%, which clinical signs are associated with the prescription of oxygen therapy.

We included the variables of interest in hierarchical multivariate logistic regression models, with a random intercept for each hospital and a random slope for the time within hospital. This model specification commonly referred to as a growth curve model, allowed the explanatory variable of time to have a different effect for each hospital. A *growth curve model* typically refers to statistical methods that allow, in our case, the estimation of inter-hospital variability in intra-hospital patterns of change over time [25]. Our approach to the growth-curve model fitting is within the multilevel modelling framework, with patients nested in hospitals nested in time points [25]. Different ways of specifying the growth-curve model using a multilevel modelling framework are explained in detail elsewhere [26]. Before embarking on the growth-curve modelling we examined for each hospital whether the 2017 strike resulted in a significant discontinuity in pulse oximetry use to check if treating time as months in the CIN, a continuous variable, instead of as calendar time was justifiable given the strike caused a 12-month data gap.

For peer review only

For peer review only - http://bmjopen.bmj.com/site/about/guidelines.xhtml

Where there was missing patient-level data, we applied fully conditional specification (FCS) multivariate imputation by chained equations (MICE) [27] to generate imputed datasets under the missing at random (MAR) assumption to allow us to analyse all eligible patients. Previous studies indicate that MAR is a reasonable approach for CIN data [28]. We contrasted findings using imputation with the findings of the complete case analysis.

Results

Figure 1 depicts the study population inclusion process. Out of the 179991 paediatric admissions to CIN hospitals, 132737 (73.7%) were eligible for analysis. Most exclusions were because an admission was randomly sampled for minimum data collection (n=33074, 70% of the exclusions) or fell in the strike year (n=8787, 19% exclusions). Of the population included in the analysis, 113196/132737 (86.1%) demonstrated at least one sign indicating increased risk of hypoxemia (respiratory distress symptoms or altered consciousness, or age below one month) . Given this high proportion, we report results based on data from all eligible admissions (Figure 1).

Figure 1: Flow chart of the inclusion criteria

Descriptive findings

From February 2014 to December 2016, on average, there was a steady improvement in pulse oximetry adoption at admission in the 13 hospitals from between 10%-20% in 2014 to between 50%-70% at the end of 2016 (Figure 2), even though no pulse oximetry devices were provided through CIN. Following the strikes in 2017, from January 2018 when services resumed up until November 2018, average pulse oximetry adoption in these 13 sites seemed to stabilise at around 50%. By December 2018, one hospital exited and 5 hospitals joined CIN and different research studies were initiated in subsets of hospitals (Table 1, Figure 2 and Supplementary Table 2). Pulse oximetry adoption ranged from an average of 40% to almost 100% across these subsets (Figure 2).

For peer review only - http://bmjopen.bmj.com/site/about/guidelines.xhtml

Figure 2: Average pulse oximetry use in the CIN hospitals over time. The number of hospitals in each study covers both presently involved and those joining the CIN later: There are 13 hospitals in the period between Feb 2014 – November 2018, with the lower panel representing transition to 18 hospitals by adding 5 hospitals and research studies beginning. The 5 new hospitals' distribution among the studies in the following way: RTS,S: 2/5; RTS,S + SEARCH: 1/5; SEARCH: 1/5; None: 1/5.

Exploring each hospital's adoption trajectory illustrates in many a general improvement in adoption throughout their participation in CIN but also examples of adoption failure (Figure 3). Figure 3 tends to confirm as depicted in Figure 2, that despite a strike of almost one year there was minimal evidence of discontinuity in hospitals' pattern of adoption from 2016 to 2018. The lack of evidence for discontinuity (supported by additional analyses in Supplementary Table 3) supports our treatment of time as 'months in CIN' as a continuous variable in place of calendar time in Figure 2 and 3 and subsequent models.

From Figure 3, 8 of the 13 hospitals recruited into the CIN by February 2014 had no use of pulse oximetry at admission at all (median at baseline 0%), with the highest baseline use being 65% (H13). For the hospitals joining after the strike (i.e. from November 2018), use of pulse oximetry during admission was generally low for 4/5 hospitals (median at baseline 25%) but one hospital already used pulse oximetry on 83% admissions (H16) (Figure 3). Hospitals demonstrated various patterns of adoption: i) low adoption when joining the CIN with a sharp improvement later (e.g. Hospital H2, H12), ii) oscillation between low and high pulse oximetry use (e.g. Hospital H3, H10, H17), iii) gradual improvement that later stabilised (e.g. Hospital H1, H4, H14), and iv) failure of adoption (H7 and H5)(Figure 3). These varying patterns of adoption, especially after December 2018, do not clearly seem to be related to study participation. Hospitals participating in both new studies, resulting in presence of two additional non-physician clinician research team members, did demonstrate a consistent improvement in pulse oximetry use at admission (Figure 2 & Figure 3). However, pulse oximetry

For peer review only - http://bmjopen.bmj.com/site/about/guidelines.xhtml

adoption at admission was also high in hospitals not participating in any study within the CIN (Figure 2 & Figure 3).

Figure 3: Hospital specific rate of pulse oximetry use at admission over time divided into sites joining from Jan 2014 and those recruited from Dec 2018. Vertical red-line indicates national strike disruption. The title is colour-coded to reflect the research study the hospital was recruited into [Blue: None, Orange: RTS,S, Red: SEARCH, Green: RTS,S+SEARCH]. H7 exited from CIN and is therefore not included in subsequent analysis.

Individual patient data and exploratory modelling

Table 2 provides the characteristics of the children admitted to these hospitals, including the level of missingness in each variable. From the study population, 32% had at least one missing variable, with the median number of variables missing per patient of 2 of 30 (IQR: 0 - 3). None of the individual variables was missing more than 30% of values. In this paediatric admission population, the prevalence of chest indrawing, tachypnoea, and grunting is 22.4%, 33% and 10.3% respectively and inability to drink (or breastfeed), a danger sign requiring urgent attention, was present in 72.7% of the admissions; 82.9% of all the admissions had at least one of these signs. The median length of stay was 3 days (IQR: 2 - 6), with a median of one additional comorbid condition at admission (IQR: 0 – 1). Pneumonia and Malaria were the leading, often comorbid, admission diagnoses (Supplementary Figure 1).

Of the 132737 cases, 48.8% (n= 64722) had a pulse oximetry measurement taken at admission. Of the 64722 patients with pulse oximetry measured at admission, 4510 (7%) had hypoxaemia (SpO_2 < 90%). Across the same period, 11428/132737 (8.6%), were documented to have oxygen prescribed at admission. Only 1484/11428 (12.8%) were confirmed to be hypoxaemic using pulse oximetry, while 6633/11428 (58%) had no pulse oximetry measurement and 3311/11428 (28.9%) were documented to have a pulse oximetry value of 90% or higher at admission. Thus, 87% oxygen use was not pulse

For peer review only - http://bmjopen.bmj.com/site/about/guidelines.xhtml

oximetry guided ($SpO_2 \geq 90\%$ or unknown) and more than twice as many children prescribed oxygen had values of 90% or more than had values <90% (3311 compared with 1484)(Figure 4).

Figure 4: Oxygen prescription and use of pulse oximetry at admission

Table 2: Descriptive summary statistics for the variables of the patient population included in the study

Indicator	Levels	Number, n (%)	Missing data, n (%)
Hospital level factors			
Malaria endemicity zone	High	53177 (40.1%)	
	Low	79560 (59.9%)	
Patient-level factors			
Age*	< 1 month	3321 (2.5%)	1221 (0.9%)
	1-11 months	38235 (28.8%)	
	12-59 months	64628 (48.7%)	
	>= 5 years	25332 (19.1%)	
Female	Present	58588 (44.1%)	1199 (0.9%)
Case is readmission	Present	13557 (10.2%)	37002 (27.9%)
Case is referral to hospital	Present	19525 (14.7%)	39879 (30%)
Presence of comorbidity	Present	82918 (62.5%)	725 (0.5%)
AVPU** = A	Present	113378 (85.4%)	10664 (8%)
Slow capillary refill	Present	2231 (1.7%)	21902 (16.5%)
Pallor	None	95612 (72%)	10799 (8.1%)
	Mild/Moderate	15596 (11.7%)	
	Severe	10730 (8.1%)	
Fever	Present	105268 (79.3%)	6952 (5.2%)
Cough	Present	64819 (48.8%)	9237 (7%)
Crackles	Present	22835 (17.2%)	11695 (8.8%)
Central cyanosis	Present	974 (0.7%)	9758 (7.4%)
Acidotic breathing	Present	3098 (2.3%)	12740 (9.6%)
Difficulty breathing	Present	38825 (29.2%)	10454 (7.9%)
Grunting	Present	13701 (10.3%)	12255 (9.2%)
Tachypnoea***	Present	43832 (33%)	31136 (23.5%)
Indrawing	Present	29698 (22.4%)	11419 (8.6%)
Wheezing	Present	7085 (5.3%)	11034 (8.3%)
Stridor	Present	2719 (2%)	14121 (10.6%)
Inability to drink	Present	96455 (72.7%)	14846 (11.2%)
Weight-for-Age Z-score	> -2SD (Normal)	74035 (55.8%)	33486 (25.2%)
	-2SD to -3SD (Low)	12377 (9.3%)	
	< -3SD (Very low)	12839 (9.7%)	
Pulse oximetry done at admission****	Present	64722 (48.8%)	
Oxygen therapy prescribed at admission	Present	11428 (8.6%)	4275 (3.2%)

For peer review only

For peer review only - http://bmjopen.bmj.com/site/about/guidelines.xhtml

Table 2: Descriptive summary statistics for the variables of the patient population included in the study			
**Indicator**	**Levels**	**Number, n (%)**	**Missing data, n (%)**
Outcome at discharge	Alive	123845 (93.3%)	499 (0.4%)
	Died	8393 (6.3%)	

Note:
* < 1 month represents neonates admitted to paediatric ward; no data from the new-born units were included.
** AVPU scale is an acronym for "Alert, Verbal, Pain, Unresponsive" and is a measure of a patient's level of consciousness ordered by severity from left to right, with "A" being the least severe.
*** Symptom is derived and calculated as present where either (1) respiratory rate > 59 and age < 1 month, (2) respiratory rate > 49 and age is 1-11 months, or (3) respiratory rate is > 39 and age is 12 months or older.
**** If missing, it was assumed that it was not done. 5525 admission cases reported having done it but did not have the SpO_2 value on the paper record.

Stridor, wheezing, acidotic breathing and cyanosis have relatively low recorded prevalence of 2%, 5.3%, 2.3% and 0.7% respectively and were not included in our previous models [10]. Whether a child is a readmission or referral from another health facility are also newly added data with higher prevalence at 10.2% and 14.7% of the patient population respectively [10] (Table 2).

Summary of the growth curve model results

Table 3 provides the results of the growth curve models, where adoption rates between hospitals were adjusted for malaria endemicity, participation in research studies, participants' demographic characteristics and clinical signs and symptoms at admission. These results are from analysis using 35 imputed datasets (32% of the cohort had at least one missing variable).

Table 3: Growth curve model of pulse oximetry adoption and orders for oxygen use at admission						
	**Pulse Oximetry Done?**			**Oxygen Prescribed?**		
**Predictors**	**OR**	**95% CI**	**p-value**	**OR**	**95% CI**	**p-value**
(Intercept)	0.03	0.01 - 0.17	<0.001	0.01	0.01 - 0.02	<0.001
Hospital factors						
Malaria Endemicity Zone: High (Ref: Low)	1.62	0.1 - 25.04	0.729	1.55	0.9 - 2.67	0.112
Time (Months in CIN)	1.11	1.05 - 1.18	<0.001	1.02	1 - 1.03	0.045
Patient-level factors						
Ref: Patient in any study [No] ǂ						

For peer review only - http://bmjopen.bmj.com/site/about/guidelines.xhtml

For peer review only

Table 3: Growth curve model of pulse oximetry adoption and orders for oxygen use at admission						
	Pulse Oximetry Done?			Oxygen Prescribed?		
Patient in any study [Pre-study period]‡ ‡	2.79	2.58 - 3.02	<0.001	1.02	0.9 - 1.15	0.800
Patient in any study [Yes]	1.46	1.35 - 1.58	<0.001	1.02	0.91 - 1.15	0.715
Referral: Yes (Ref: No)	1.05	1.01 - 1.09	0.025	1.19	1.12 - 1.26	<0.001
Readmission: Yes (Ref: No)	1.01	0.97 - 1.05	0.732	1.07	1 - 1.15	0.052
Is a weekend admission: Yes (Ref: No)	0.98	0.95 - 1.01	0.144	1.07	1.02 - 1.13	0.009
PAR used: Yes (Ref: No)	1.99	1.79 - 2.21	<0.001	1.23	1.02 - 1.48	0.031
PAR used * Time (Months in CIN)	1.01	1 - 1.01	<0.001	1	1 - 1.01	0.562
Ref: Age [> 59 months]						
Age [< 1 month]	0.46	0.41 - 0.51	<0.001	1.96	1.62 - 2.35	<0.001
Age [1-11 months]	1.08	1.03 - 1.13	<0.001	1.43	1.32 - 1.54	<0.001
Age [12-59 months]	1.09	1.05 - 1.13	<0.001	1.08	1 - 1.16	0.054
Female: Yes (Ref: No)	1	0.97 - 1.02	0.723	1.02	0.97 - 1.06	0.424
Fever: Yes (Ref: No)	1.18	1.14 - 1.23	<0.001	0.78	0.73 - 0.83	<0.001
Cough: Yes (Ref: No)	1.04	1.01 - 1.08	0.008	1.36	1.29 - 1.44	<0.001
Difficulty Breathing: Yes (Ref: No)	1.06	1.02 - 1.1	0.002	2.79	2.64 - 2.94	<0.001
Stridor: Yes (Ref: No)	1.05	0.95 - 1.16	0.325	1.14	1.02 - 1.27	0.017
Cyanosis: Yes (Ref: No)	0.77	0.65 - 0.92	0.004	1.5	1.27 - 1.77	<0.001
Indrawing: Yes (Ref: No)	1.25	1.2 - 1.31	<0.001	2.36	2.23 - 2.49	<0.001
Grunting: Yes (Ref: No)	0.88	0.84 - 0.93	<0.001	1.9	1.8 - 2	<0.001
Acidotic Breathing: Yes (Ref: No)	0.89	0.8 - 0.98	0.018	1.34	1.22 - 1.47	<0.001
Wheezing: Yes (Ref: No)	1.05	0.98 - 1.12	0.167	1.13	1.06 - 1.21	0.001
Crackles: Yes (Ref: No)	1.06	1.01 - 1.11	0.010	1.25	1.19 - 1.32	<0.001
Tachypnoea: Yes (Ref: No)	0.96	0.93 - 0.99	0.022	1.59	1.51 - 1.68	<0.001
Alert (AVPU < A): No (Ref: Yes)	0.96	0.9 - 1.03	0.238	1.95	1.81 - 2.1	<0.001
Inability to drink: Yes (Ref: No)	1.04	1 - 1.08	0.043	0.8	0.76 - 0.85	<0.001
Ref: Pallor [None]						
Pallor [Mild/Moderate]	0.93	0.89 - 0.97	0.002	1.1	1.02 - 1.17	0.008
Pallor [Severe]	0.94	0.89 - 0.99	0.024	1.3	1.19 - 1.42	<0.001
Ref: WAZ [Normal]						
WAZ [Low]	0.97	0.92 - 1.01	0.135	0.98	0.91 - 1.05	0.551
WAZ [Very Low]	0.94	0.9 - 0.99	0.020	0.93	0.87 - 1	0.059

For peer review only - http://bmjopen.bmj.com/site/about/guidelines.xhtml

For peer review only

Table 3: Growth curve model of pulse oximetry adoption and orders for oxygen use at admission

	Pulse Oximetry Done?			Oxygen Prescribed?		
Slow Capillary Refill: Yes (Ref: No)	0.88	0.78 - 1	0.051	0.97	0.83 - 1.13	0.693
Ref: Hypoxaemia [Unknown]						
Hypoxaemia [No]				0.59	0.56 - 0.62	<0.001
Hypoxaemia [Yes]				1.92	1.77 - 2.08	<0.001

Note:

ǂ"*Patient is in any study*" point estimates and confidence intervals for the *"pulse oximetry done?"* outcome do not overlap with ones from complete case analysis (Supplementary Table 4). From the complete case sensitivity analysis, there is a material difference in the estimated association of involvement in any research study variable with the pulse oximetry use at admission outcome (Table 3, Supplementary Table 4); While the direction of association is the same (positive), the confidence intervals do not overlap. With the age variable, the direction of the association is inverted between the complete case and multiple imputation analyses, and children aged < 1 month admitted to the paediatric ward are completely missing in the complete case analysis. These material difference might be attributable to the fact that complete case analysis and our multiple imputation approach make different assumptions of missingness mechanism in the data (i.e. Missing Completely At Random (MCAR) mechanism -which is potentially more biased, and a MAR mechanism respectively).

ǂǂ"*Patient is in any study[Pre-study period]*" reflects the time leading up to any research study starting when no study was being conducted i.e. February 2014 – November 2018.

Associations of the hospital and clinical features with the outcomes from Table 3 are time-invariant (i.e. independent of the effect of time in months in CIN), except for the interaction term of PAR use with time. From Supplementary Table 4 and Figure 3, the models' high Intraclass Correlation Coefficient (ICC) is indicative that hospital factors play a bigger role in the adoption of pulse oximetry over time compared to individual patient-level factors. The contribution of hospital level factors is less pronounced for oxygen prescription at admission. The relatively high ICC is not unexpected in longitudinal models with measurements clustered by hospital and given the varying patterns of adoption seen in Figure 3.

Association with the use of pulse oximetry at admission

Over the entire study period and across all hospitals pulse oximetry use at admission improved as time participating in the CIN increased (month-to-month improvement OR: 1.11, 95% CI: 1.05 – 1.18). This

For peer review only - http://bmjopen.bmj.com/site/about/guidelines.xhtml

For peer review only

was likely driven by noticeable improvement in 10 hospitals and in 5 of these specifically there were substantive positive changes in adoption of pulse oximetry on a month-to-month basis; (Supplementary Figure 2). Those improving most tended to have very low use of oximetry at the time they joined the CIN (Supplementary Figure 2). This improvement was seen in tandem with the improvement in PAR use which might be expected to encourage better assessment and potentially, therefore, aid clinical decision making.

From Table 3, patients admitted with signs of fever, cough, difficulty breathing, chest indrawing, crackles, and inability to drink had significantly higher odds of having pulse oximetry measurement taken at admission over time than those without these signs. With a larger dataset, the inclusion of new variables and adjusting for the time in the CIN, our findings conflict with previous findings on the significance of tachypnoea, altered consciousness (AVPU < A), and inability to drink as signs associated with the use of pulse oximetry at admission with a reversal in association (e.g. Tachypnoea, Inability to drink) or no statistically appreciable association (e.g. AVPU <A)[10]. Grunting and acidotic breathing symptoms, likely to be associated with malaria and anaemia (Supplementary Figure 1), were statistically, not significantly associated with pulse oximetry use at admission (Table 3). Cyanosis, grunting, and acidotic breathing –which were previously not reported [10]- were significantly associated with the odds of pulse oximetry done at admission being lower than one. Our findings also highlight that referral into the hospital is positively associated with the use of pulse oximeters at admission (OR: 1.05, 95% CI: 1.01 - 1.09)(Table 3) and that patients admitted to hospitals that were part of an active research study were 49% more likely to have a pulse oximetry measurement taken at admission than in hospitals not participating in any study (Table 3). Additional analyses suggest the more clinical signs a child has of respiratory distress the more likely they were to be hypoxemic (Supplementary Figure 3).

Associations with oxygen prescription at admission

Admission with any sign of respiratory distress where the pulse oximetry measure is unknown is associated with oxygen prescription at admission (Table 3). The specific signs that appear to prompt

For peer review only - http://bmjopen.bmj.com/site/about/guidelines.xhtml

oxygen prescription, in reducing magnitude of association, include difficulty breathing, indrawing, AVPU <A, grunting, and tachypnoea. Admissions with altered consciousness (AVPU < A) had almost twice the odds of oxygen prescription at admission, independent of respiratory signs (Table 3). Interestingly, the direction of association with oxygen prescription is inverse to that for use of pulse oximetry for: age of the child, signs of cyanosis, grunting, acidotic breathing, AVPU <A, pallor, tachypnoea, fever and inability to drink.

With the inclusion of new variables and adjusting for the time in CIN, our findings affirm previous analyses but show the magnitude of association of cough and difficulty breathing with oxygen prescription at admission to be higher than previously reported [10]. Acidotic breathing, stridor and wheezing, clinical signs omitted from the previous study, were shown to be statistically significant and positively associated with the odds of being prescribed oxygen at admission. Grunting and acidotic breathing, likely to be associated with Malaria and Anaemia diagnoses, were not statistically associated with having pulse oximetry measurement taken at admission but were strongly associated with the prescription of oxygen at admission (Table 3).

Our findings also highlight that being a patient referred into the hospital (OR: 1.19, 95% CI: 1.12 - 1.26) or admitted during the weekend (OR: 1.07, 95% CI: 1.02 - 1.13) is positively associated with oxygen prescription at admission (Table 3). Oxygen prescription at admission increased with time as part of CIN, having a statistically significant but clinically small month-to-month change (OR: 1.02, 95% CI: 1.00 – 1.03). The improvement in the PAR use (OR: 1.23, 95% CI: 1.02 - 1.48) also appears to be aiding clinical decision making in the prescription of oxygen based on the recording of features of respiratory distress.

Discussion

Over a period of 7 years, with prolonged strikes precluding data collection in 2017, use of pulse oximetry in a population of admissions where 86% have at least one feature indicating risk of hypoxemia increased slowly with each month of participation in CIN (OR: 1.11, 95% CI: 1.05 – 1.18).

For peer review only

For peer review only - http://bmjopen.bmj.com/site/about/guidelines.xhtml

Most hospitals at the start of their participation in CIN used pulse oximetry in 25% or fewer children. In the final quarter of 2020, nine months into the COVID-19 pandemic, use of pulse oximetry across all hospitals was 81.5%. The highly variable patterns of pulse oximetry adoption across hospitals (Figure 3) and high intraclass correlation coefficients in our models point to the major impact of hospital-level factors on the use of pulse oximetry. These have been previously described and include equipment supply, maintenance, local leadership and clinical efforts to train and promote oximetry use amongst others [10]. CIN provided regular feedback to hospitals including on pulse oximetry use and SpO_2 values throughout a hospital's participation in CIN and engaged subsets of hospitals in research projects that resulted in the placement of one or two additional non-physician clinicians (i.e. clinical officers, nurses) in some hospitals. In the absence of data from other settings it is hard to determine if the increase in pulse oximetry use is part of a wider secular trend (a phenomenon referred to as the "rising tide" [29]) or associated with CIN participation. Whatever the reasons, it is instructive that adoption of pulse oximetry remains incomplete after 7 years even amongst inpatient populations with severe illness in moderately sized hospitals. This slow pattern of adoption for an established technology that is widely believed to be beneficial has been observed in high-income settings. It suggests we may only realise the proposed benefits of many technologies including diagnostics much more slowly than is often claimed unless much greater attention is paid to strengthening health service delivery at scale [30, 31].

Overall, approximately 9% of admissions were prescribed oxygen with a small increase associated with each month of participation in CIN (OR: 1.02, 95% CI: 1.00 – 1.03). Interestingly, in our models the intraclass correlation coefficients exploring oxygen use did not suggest strong hospital-level effects, placing more emphasis on patient-level factors. Amongst the admissions prescribed oxygen in 87% this was not apparently guided by a pulse oximetry value (SpO2) of < 90%, the recommended level for intervention to address hypoxemia in current pneumonia guidelines. It may have been that in some patients this was because of a diagnosis of shock or severe asthma where the normal SpO_2 minimum threshold is 94%, however these diagnoses are relatively uncommon in Kenyan hospitals [1, 32]. In

For peer review only - http://bmjopen.bmj.com/site/about/guidelines.xhtml

fact, more than twice as many children receiving oxygen had pulse oximetry values 90% or more than values < 90% (Figure 4). From WHO guidelines, any child with SpO2 < 90% should receive oxygen [1].

We attempt to integrate our new findings of patient-level factors associated with the use of pulse oximetry and prescription of oxygen at admission with earlier findings that addressed mainly hospital-level factors in Figure 5. Lower chest-wall indrawing is strongly and positively associated with pulse oximetry being done at admission and prescription of oxygen when pulse oximetry is not done. We have previously shown an association between indrawing and mortality for pneumonia admissions and others have recently indicated indrawing may be a useful referral criterion from the community or primary care to a hospital in the absence of pulse oximetry [5, 33]. However, randomised trials, and WHO guidance, suggest pneumonia associated with indrawing can be safely treated as an outpatient [2, 32]. Our data does not directly inform the debate on the value of indrawing as screening sign of illness severity but does perhaps suggest it remains an important factor in Kenyan clinicians' decision-making.

Other signs prompting pulse oximeter use are fever, cough, difficulty breathing, crackles and inability to drink (Table 3, Figure 5). All respiratory signs are associated with oxygen prescription (Table 3, Supplementary Table 5, Figure 5) and a pulse oximetry value <90% was associated with twice the odds of oxygen prescription compared with those whose oxygen saturation was unknown. Where information from pulse oximetry was not available cyanosis, grunting, acidotic breathing, AVPU <A, pallor, and tachypnoea appeared to prompt oxygen use (Table 3, Figure 5). Several of these signs were not associated with the use of pulse oximetry at admission, perhaps because healthcare providers are taught and then view these signs as indicative of very severe illness (as reported in the literature) requiring intervention with oxygen irrespective of oxygen saturation [1, 32, 33]. Interestingly, use of the standardised paediatric admission record (PAR) was associated with twice the odds of a patient having a pulse oximetry measurement and 1.23 times the odds of having oxygen therapy prescribed at admission, where indicated (Table 3). It is possible this effect reflects its role as a prompt to conduct

For peer review only - http://bmjopen.bmj.com/site/about/guidelines.xhtml

a complete assessment and promote reflection on the use of core interventions recommended in guidelines.

It has been well described in many settings that the logical world represented in clinical guidelines is often not replicated in the more complex world of everyday practice [34-36]. While there remain debates in the scientific world around which oxygen saturation threshold for which condition in which setting should guide the use of oxygen [5, 37, 38], our data remind us that clinicians are often influenced by many other factors in making such decisions [39]. Clinical decision making is not straightforward, and it is difficult to capture from routinely collected observational data. Reducing the decision of whether to prescribe or withhold oxygen to a pulse oximetry reading might inadequately reflect the complexities that a clinician encounters at the patient bedside. This has important implications as the presence or prevalence of hypoxemia, however defined, may be used as proxies for rational use or need for oxygen respectively. Whether clinicians should be strongly advised to adhere to pulse oximetry defined hypoxemia thresholds to determine oxygen use remains unclear as research has rarely addressed the effects this would have on resource use or patient outcomes [2].

Figure 5: Adapted Integrative Model of Behavioural Prediction (Enoch et al. [10]). Dashes indicate components of the model that are modified or added to reflect the findings from our new analyses. HCW: healthcare worker.

Strengths and limitations

The data we present are a rare example of long-term tracking of hospital practice in a routine LMIC setting. The longitudinal nature of our observations offers important insights but also poses challenges. Major changes may occur in the health sector over long periods, as exemplified by the prolonged strikes in Kenya. Many factors ranging from national and local procurement or equipment donations, to changes in local leadership and hospital's participation in specific studies might influence the adoption of pulse oximetry and use of oxygen we describe. Furthermore, we have no contemporaneous data from Kenyan settings that do not belong to the CIN for comparison and most

For peer review only

For peer review only - http://bmjopen.bmj.com/site/about/guidelines.xhtml

other studies exploring the use of pulse oximetry are essentially cross-sectional. We do, however, feel our data are at least a useful illustration of the slow and sometimes erratic process of pulse oximetry adoption in LMIC hospitals that has broader implications for thinking on technology adoption. Further challenges of the large-scale, longitudinal nature of our work are data quality and analytic methods. The CIN has made efforts to capture high-quality data through continuous training of clerks linked to error checking procedures and regular engagement with hospitals that include monitoring and feedback [17, 23, 24]. Nonetheless, imputation procedures that assume a missing at random mechanism had to be employed to enable analyses of all eligible case records [27, 40]. We also had to employ a complex modelling framework to explore patient-level associations with our outcomes. These attempted to accommodate and account for between hospital variability, within hospital non-linear trends in the outcomes over time and entry into the CIN at different time points. We tested our assumptions where possible (e.g. that the strike year was not associated with major changes in practice). However, while our large dataset enabled us to identify many credible associations specific findings should be interpreted cautiously.

Conclusions

Over time, there has been a progressive increase in the use of pulse oximeters at admission in the Kenyan hospitals we studied. After seven years, however, use in this high mortality population where almost 9 out of 10 children have least one sign indicating increased risk of hypoxemia is not universal and major differences exist between hospitals in the adoption of this technology. Guidelines suggest pulse oximetry should determine which children receive oxygen. Our data suggest this is often not the case even in late 2020 during the COVID-19 pandemic, and that many organisational and clinical factors influence whether oximetry is used at all or whether recommended hypoxemia definitions are used to guide the use of oxygen. As global efforts gain pace to provide pulse oximeters and oxygen systems our data show it is perhaps naïve to expect they will be adopted and employed in the rational ways imagined in guidelines and policies.

For peer review only

Appendix

CRediT author statement

Timothy Tuti: Conceptualization, Methodology, Software, Data curation, Visualization, Writing- Original draft, Writing - Reviewing and Editing. **Jalemba Aluvaala**: Methodology, Investigation, Writing- Reviewing and Editing. **Samuel Akech**: Methodology, Investigation, Writing- Reviewing and Editing. **Ambrose Agweyu**: Methodology, Investigation, Writing- Reviewing and Editing. **Grace Irimu**: Writing- Reviewing and Editing **Mike English**: Conceptualization, Methodology, Supervision, Investigation, Writing- Reviewing and Editing.

Ethics approval and consent to participate

This CIN study was approved by the Kenya Medical Research Institute's (KEMRI) Scientific and Ethics Review Unit (#SERU 3459). Additionally, it was approved by the Ministry of Health, with the Medical Superintendents of participant hospitals given consent for participation. Individual consent for access to de-identified patient data was not required.

Consent to publish

This paper is published with the permission of the Director of KEMRI.

Acknowledgements

The Clinical Information Network Author Group: The CIN author group who contributed to the network's development, data collection, data management, implementation of audit and feedback and who reviewed and approved this publication include: 1) Central CIN team members: Lynda Isaaka, George Mbevi, Cynthia Khaenzi, Paul Mwaniki, Mercy Chepkirui, John Wainaina, Livingstone Mumelo, Edith Gicheha, Monica Musa, Naomi Muinga and Muthoni Ogola. 2) National partners: Laura Oyiengo, Caroline Mwangi, Fred Were, and (3) Hospital teams: (**Paediatricians)** Juma Vitalis, Nyumbile Bonface, Roselyne Malangachi, Christine Manyasi, Catherine Mutinda, David Kibiwott Kimutai, Rukia Aden, Caren Emadau, Elizabeth Atieno Jowi, Cecilia Muithya, Charles Nzioki, Supa Tunje, Dr. Penina Musyoka, Wagura Mwangi, Agnes Mithamo, Magdalene Kuria, Esther Njiru, Mwangi Ngina, Penina Mwangi, Rachel Inginia, Melab Musabi, Emma Namulala, Grace Ochieng, Lydia Thuranira, Felicitas Makokha,

For peer review only

Josephine Ojigo, Beth Maina, Catherine Mutinda, Mary Waiyego, Bernadette Lusweti, Angeline Ithondeka, Julie Barasa, Meshack Liru, Elizabeth Kibaru, Alice Nkirote Nyaribari, Joyce Akuka, Joyce Wangari;

(Nurses) Amilia Ngoda, Aggrey Nzavaye Emenwa, Dolphine Mochache, Patricia Nafula Wesakania, George Lipesa, Jane Mbungu, Marystella Mutenyo, Joyce Mbogho, Joan Baswetty, Ann Jambi, Josephine Aritho, Beatrice Njambi, Felisters Mucheke, Zainab Kioni, Jeniffer, Lucy Kinyua, Margaret Kethi, Alice Oguda, Salome Nashimiyu Situma, Nancy Gachaja, Loise N. Mwangi, Ruth Mwai, irginia Wangari Muruga, Nancy Mburu, Celestine Muteshi, Abigael Bwire, Salome Okisa Muyale, Naomi Situma, Faith Mueni, Hellen Mwaura, Rosemary Mututa, Caroline Lavu, Joyce Oketch, Jane Hore Olum, Orina Nyakina, Faith Njeru, Rebecca Chelimo, Margaret Wanjiku Mwaura, Ann Wambugu, Epharus Njeri Mburu, Linda Awino Tindi, Jane Akumu, Ruth Otieno, Slessor Osok;

(HRIOs) Seline Kulubi, Susan Wanjala, Pauline Njeru, Rebbecca Mukami Mbogo, John Ollongo, Samuel Soita, Judith Mirenja, Mary Nguri, Margaret Waweru, Mary Akoth Oruko, Jeska Kuya, Caroline Muthuri, Esther Muthiani, Esther Mwangi, Joseph Nganga, Benjamin Tanui, Alfred Wanjau, Judith Onsongo, Peter Muigai, Arnest Namayi, Elizabeth Kosiom, Dorcas Cherop, Faith Marete, Johanness Simiyu, Collince Danga, Arthur Otieno Oyugi, Fredrick Keya Okoth.

Data Availability

Patient-level data used in this report are under the primary jurisdiction of the Ministry of Health in Kenya. The authors had full access to all the quantitative data in the study and take responsibility for the integrity of the data and the accuracy of the data analysis. Researchers can contact the KEMRI Wellcome Trust Research Programme Data Governance Committee (dgc@kemri-wellcome.org) for advice on accessing the data including whether a new ethical review submission and approval from the Ministry of Health in Kenya would be required.

Funding

The work described was supported by multiple funders. These include the Wellcome Trust (#207522) through an award to ME as a Senior Fellowship and a core grant awarded to the KEMRI-Wellcome

For peer review only

Trust Research Programme (#092654); by a grant from World Health Organization: An evaluation in Kenya of the cluster-randomized RTS,S/AS01 malaria vaccine implementation pilot through routine health systems in moderate to high malaria transmission settings (WHO-Project requisition number 2018/854999); by a grant to the NEST program from the John D. and Catherine T. MacArthur Foundation, the Bill & Melinda Gates Foundation, ELMA Philanthropies, and The Children's Investment Fund Foundation UK under agreements to William Marsh Rice University with a sub-agreement to University of Oxford Centre for Tropical Medicine and Global Health; and an award to AA (MR/R006083/1) from the Foreign, Commonwealth and Development Office (FCDO), the MRC, the National Institute for Health Research (NIHR) and Wellcome Trust Joint Global Health Trials Scheme.
For the purpose of Open Access, the author has applied a CC-BY public copyright licence to any author accepted manuscript version arising from this submission.

The funders had no role in the preparation of this report or the decision to submit for publication.

Competing interests

We have read the journal's policy and the authors of this manuscript have no competing interests to declare

Abbreviations:

CI, confidence interval; CIN, Clinical Information Network; FCS, fully conditional specification; HCW, healthcare worker; HRIO, Hospital Records Information Officer; ICC, Intra-cluster Correlation Coefficient; KEMRI, Kenya Medical Research Institute; LMIC, low- and middle-income country; MAR, Missing At Random; MCAR, Missing Completely At Random; MICE, Multivariate Imputation by Chained Equations; OR, odds ratio; PAR, Paediatric Admission Record; SERU, KEMRI's Scientific and Ethics Review Unit; WHO, World Health Organization

References

1. World Health Organization, *Manual on use of oxygen therapy in children.* World Health Organization (WHO), 2016.

For peer review only

2. Enoch, A.J., M. English, and S. Shepperd, *Does pulse oximeter use impact health outcomes? A systematic review.* Archives of disease in childhood, 2016. **101**(8): p. 694-700.

3. Jouffroy, R., D. Jost, and B. Prunet, *Prehospital pulse oximetry: a red flag for early detection of silent hypoxemia in COVID-19 patients.* Critical Care, 2020. **24**(1): p. 1-2.

4. Starr, N., et al., *Pulse oximetry in low-resource settings during the COVID-19 pandemic.* The Lancet Global Health, 2020. **8**(9): p. e1121-e1122.

5. Colbourn, T., et al., *Predictive value of pulse oximetry for mortality in infants and children presenting to primary care with clinical pneumonia in rural Malawi: A data linkage study.* PLoS medicine, 2020. **17**(10): p. e1003300.

6. Adebola, O., O. Babatunde, and O. Bose, *Hypoxemia predicts death from severe falciparum malaria among children under 5 years of age in Nigeria: the need for pulse oximetry in case management.* African health sciences, 2014. **14**(2): p. 397-407.

7. Lozano, J., *Epidemiology of hypoxaemia in children with acute lower respiratory infection [oxygen therapy in children].* The international journal of tuberculosis and lung disease, 2001. **5**(6): p. 496-504.

8. Liu, L., et al., *Global, regional, and national causes of under-5 mortality in 2000–15: an updated systematic analysis with implications for the Sustainable Development Goals.* The Lancet, 2016. **388**(10063): p. 3027-3035.

9. Gray, A.Z., et al., *Improved oxygen systems in district hospitals in Lao PDR: a prospective field trial of the impact on outcomes for childhood pneumonia and equipment sustainability.* BMJ paediatrics open, 2017. **1**(1).

For peer review only

For peer review only - http://bmjopen.bmj.com/site/about/guidelines.xhtml

10. Enoch, A.J., et al., *Variability in the use of pulse oximeters with children in Kenyan hospitals: A mixed-methods analysis.* PLoS medicine, 2019. **16**(12): p. e1002987.
11. Irimu, G., et al., *Tackling health professionals' strikes: an essential part of health system strengthening in Kenya.* BMJ global health, 2018. **3**(6).
12. García-Romero, A., Á. Escribano, and J.A. Tribó, *The impact of health research on length of stay in Spanish public hospitals.* Research Policy, 2017. **46**(3): p. 591-604.
13. Lichten, C.A., et al., *Does a biomedical research centre affect patient care in local hospitals?* Health research policy and systems, 2017. **15**(1): p. 2.
14. Gathara, D., et al., *Variation in and risk factors for paediatric inpatient all-cause mortality in a low income setting: data from an emerging clinical information network.* BMC pediatrics, 2017. **17**(1): p. 99.
15. Von Elm, E., et al., *The Strengthening the Reporting of Observational Studies in Epidemiology (STROBE) statement: guidelines for reporting observational studies.* Annals of internal medicine, 2007. **147**(8): p. 573-577.
16. English, M., et al., *Building learning health systems to accelerate research and improve outcomes of clinical care in low-and middle-income countries.* PLoS medicine, 2016. **13**(4): p. e1001991.
17. Irimu, G., et al., *Approaching quality improvement at scale: a learning health system approach in Kenya.* Archives of disease in childhood, 2018. **103**(11): p. 1013-1019.
18. Gachau, S., et al., *Does audit and feedback improve the adoption of recommended practices? Evidence from a longitudinal observational study of an emerging clinical network in Kenya.* BMJ global health, 2017. **2**(4): p. e000468.

For peer review only

For peer review only - http://bmjopen.bmj.com/site/about/guidelines.xhtml

19. Mwakyusa, S., et al., *Implementation of a structured paediatric admission record for district hospitals in Kenya–results of a pilot study.* BMC international health and human rights, 2006. **6**(1): p. 9.
20. English, M., et al., *Implementing locally appropriate guidelines and training to improve care of serious illness in Kenyan hospitals: a story of scaling-up (and down and left and right).* Archives of disease in childhood, 2011. **96**(3): p. 285-290.
21. Ministry of Health. *Basic Paediatric Protocols*. 2016 [cited 2020 11th Nov]; 4th Edition:[Available from: http://guidelines.health.go.ke/#/category/27/266/meta.
22. Health Services Unit KEMRI–Wellcome Trust Research Programme. *Paediatric Tools & Hospital forms 2016*. 2016 [cited 2020 21st August]; Available from: http://www.kenyapaediatric.org/idoc/index.php/en/etat/instructors/paeditric-tools-a-hospital-forms.html.
23. Tuti, T., et al., *Innovating to enhance clinical data management using non-commercial and open source solutions across a multi-center network supporting inpatient pediatric care and research in Kenya.* Journal of the American Medical Informatics Association, 2016. **23**(1): p. 184-192.
24. Tuti, T., et al., *Improving documentation of clinical care within a clinical information network: an essential initial step in efforts to understand and improve care in Kenyan hospitals.* BMJ global health, 2016. **1**(1): p. e000028-e000028.
25. Curran, P.J., K. Obeidat, and D. Losardo, *Twelve frequently asked questions about growth curve modeling.* Journal of cognition and development, 2010. **11**(2): p. 121-136.
26. Kristoffer, M. *Using R and lme/lmer to fit different two- and three-level longitudinal models*. 2015 [cited 2020 30th September]; Available from: https://rpsychologist.com/r-guide-longitudinal-lme-lmer.

For peer review only - http://bmjopen.bmj.com/site/about/guidelines.xhtml

27. Buuren, S.v. and K. Groothuis-Oudshoorn, *mice: Multivariate imputation by chained equations in R.* Journal of statistical software, 2010: p. 1-68.
28. Gachau, S., et al., *Handling missing data in modelling quality of clinician-prescribed routine care: Sensitivity analysis of departure from missing at random assumption.* Statistical Methods in Medical Research, 2020: p. 0962280220918279.
29. Chen, Y.-F., et al., *Secular trends and evaluation of complex interventions: the rising tide phenomenon.* BMJ quality & safety, 2016. **25**(5): p. 303-310.
30. Oliwa, J.N., et al., *Diagnostic practices and estimated burden of tuberculosis among children admitted to 13 government hospitals in Kenya: an analysis of two years' routine clinical data.* PloS one, 2019. **14**(9): p. e0221145.
31. Graham, H.R., et al., *Adoption of paediatric and neonatal pulse oximetry by 12 hospitals in Nigeria: a mixed-methods realist evaluation.* BMJ global health, 2018. **3**(3): p. e000812.
32. World Health Organization, *Pocket book of hospital care for children: guidelines for the management of common childhood illnesses*. 2013: World Health Organization.
33. Agweyu, A., et al., *Appropriateness of clinical severity classification of new WHO childhood pneumonia guidance: a multi-hospital, retrospective, cohort study.* The Lancet Global health, 2018. **6**(1): p. e74-e83.
34. Gabbay, J. and A. le May, *Mindlines: making sense of evidence in practice*. 2016, British Journal of General Practice.
35. Murray, E., et al., *Normalisation process theory: a framework for developing, evaluating and implementing complex interventions.* BMC medicine, 2010. **8**(1): p. 1-11.
36. Thompson, D.S., et al., *Scoping review of complexity theory in health services research.* BMC health services research, 2016. **16**(1): p. 1-16.

For peer review only - http://bmjopen.bmj.com/site/about/guidelines.xhtml

37. Serpa Neto, A., et al., *Pragmatic Recommendations for the Management of Acute Respiratory Failure and Mechanical Ventilation in Patients with COVID-19 in Low-and Middle-Income Countries.* The American Journal of Tropical Medicine and Hygiene, 2021: p. tpmd200796.

38. Maitland, K., et al., *Children's Oxygen Administration Strategies Trial (COAST): A randomised controlled trial of high flow versus oxygen versus control in African children with severe pneumonia.* Wellcome Open Research, 2017. **2**.

39. WHO;, lifebox;, and ALIMA;. *Using pulse oximetry for decision support for COVID-19*. 2020 11th April 2020 [cited 2020 30th October]; Available from: https://www.lifebox.org/wp-content/uploads/2020/04/Pulse-Oximetry-for-COVID-19-Decision-Tool-Lifebox-FINAL1.pdf.

40. Sperrin, M. and G.P. Martin, *Multiple imputation with missing indicators as proxies for unmeasured variables: simulation study.* BMC Medical Research Methodology, 2020. **20**(1): p. 1-11.

For peer review only

For peer review only - http://bmjopen.bmj.com/site/about/guidelines.xhtml

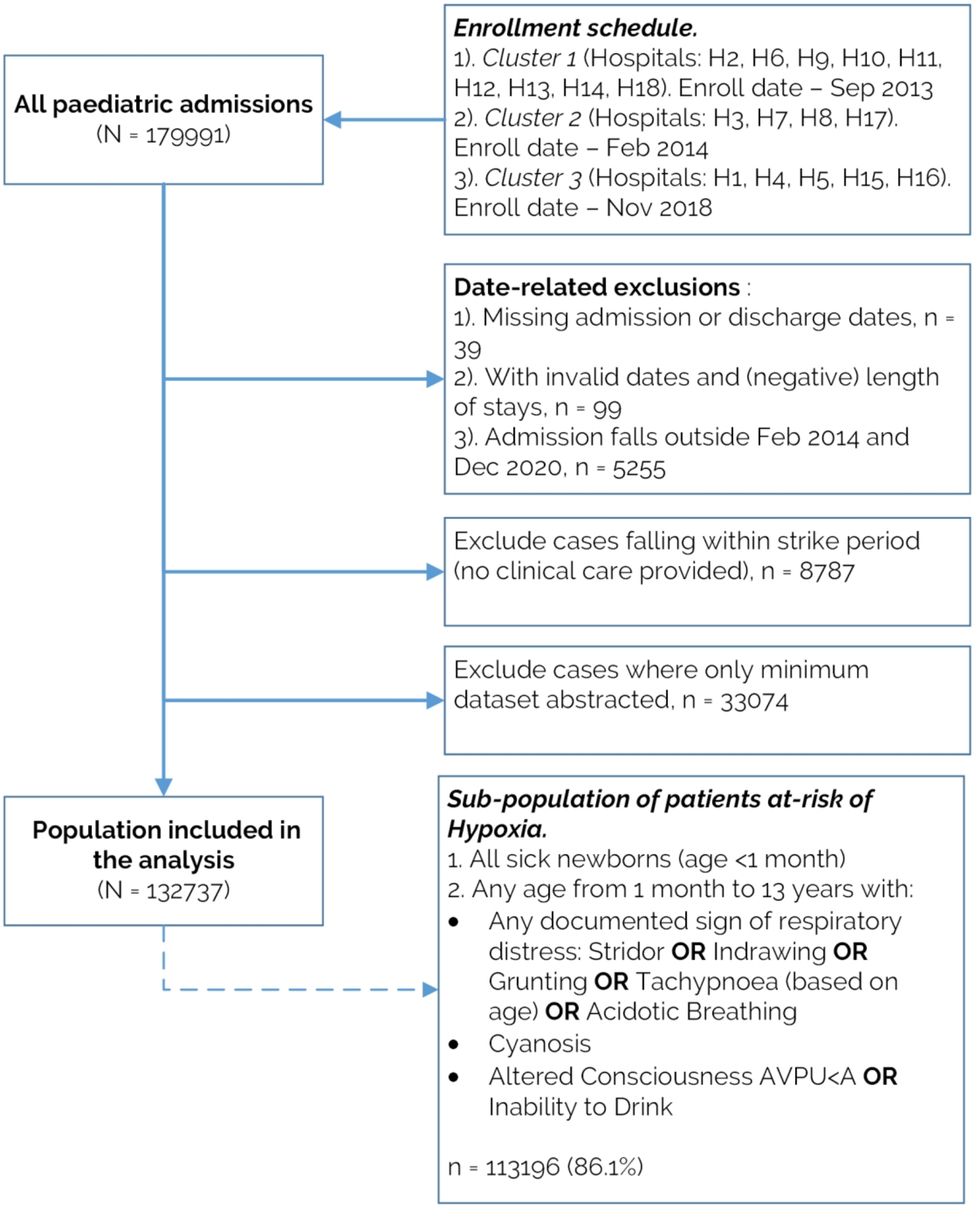

Flow chart of the inclusion criteria

146x180mm (300 x 300 DPI)

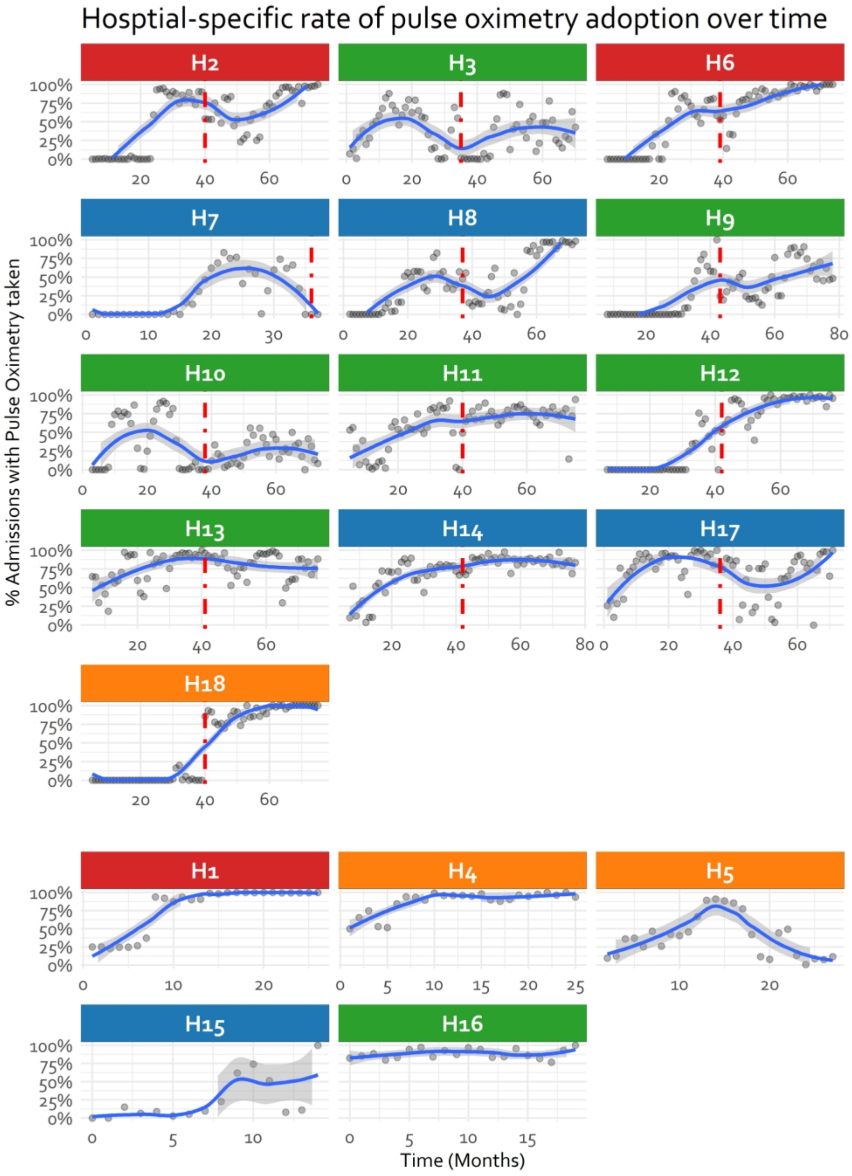

Hospital specific rate of pulse oximetry use at admission over time divided into sites joining from Jan 2014 and those recruited from Dec 2018. Vertical red-line indicates national strike disruption. The title is colour-coded to reflect the research study the hospital was recruited into [Blue: None, Orange: RTS,S, Red: SEARCH, Green: RTS,S+SEARCH]. H7 exited from CIN and is therefore not included in subsequent analysis.

179x246mm (300 x 300 DPI)

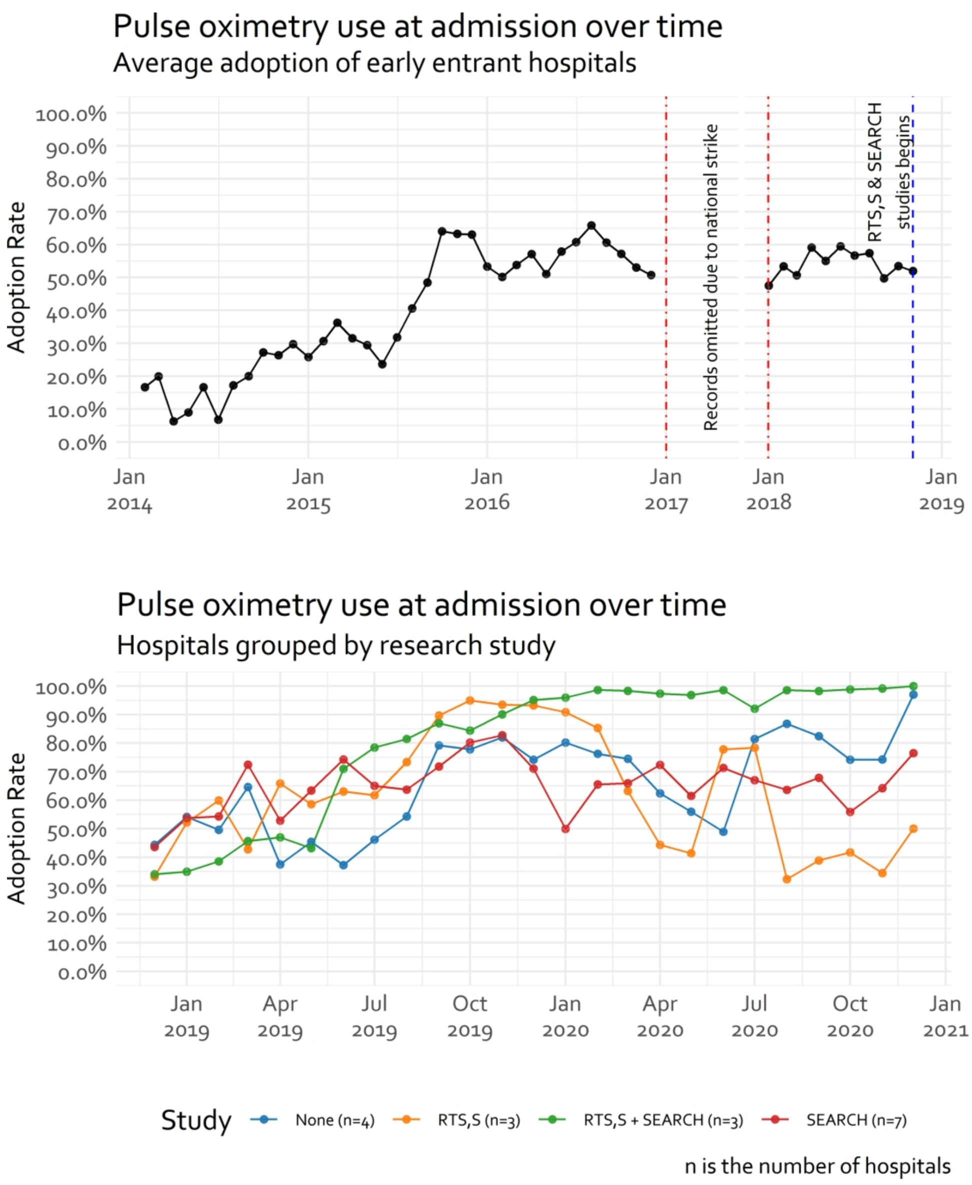

Average pulse oximetry use in the CIN hospitals over time. The number of hospitals in each study covers both presently involved and those joining the CIN later: There are 13 hospitals in the period between Feb 2014 – November 2018, with the lower panel representing transition to 18 hospitals by adding 5 hospitals and research studies beginning. The 5 new hospitals' distribution among the studies in the following way: RTS,S: 2/5; RTS,S + SEARCH: 1/5; SEARCH: 1/5; None: 1/5.

179x209mm (300 x 300 DPI)

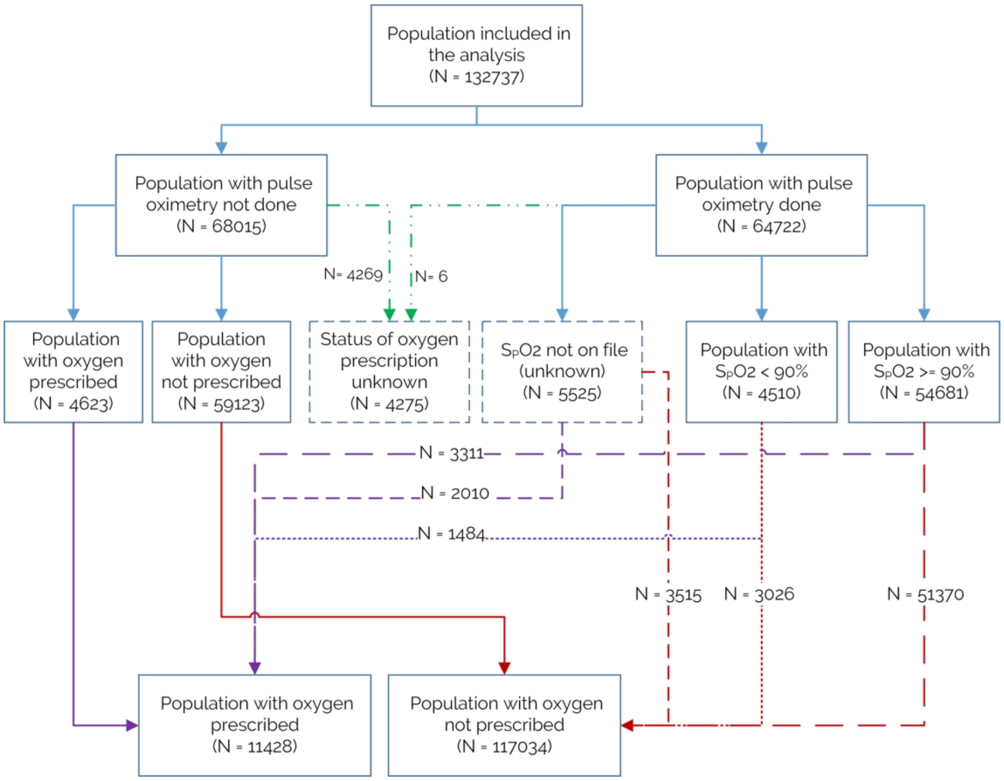

Oxygen prescription and use of pulse oximetry at admission

179x138mm (300 x 300 DPI)

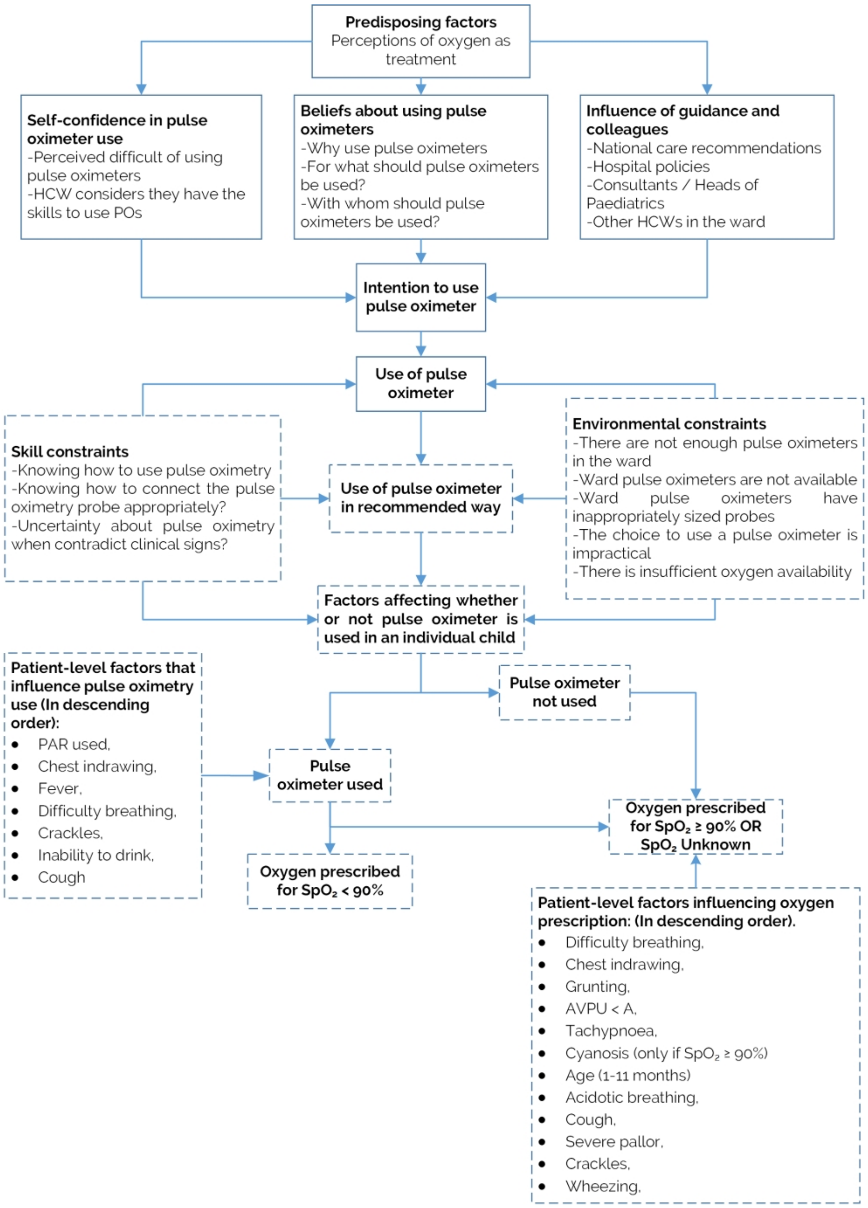

Adapted Integrative Model of Behavioural Prediction (Enoch et al. [10]). Dashes indicate components of the model that are modified or added to reflect the findings from our new analyses. HCW: healthcare worker.

180x248mm (300 x 300 DPI)

Supplementary Material

Supplementary Table 1: Number of patients included into the study per hospital	
**Hospital**	**n (%)**
**H1**	6065 (4.6%)
**H2**	11636 (8.8%)
**H3**	9584 (7.2%)
**H4**	1841 (1.4%)
**H5**	4021 (3%)
**H6**	13107 (9.9%)
**H7**	4164 (3.1%)
**H8**	8983 (6.8%)
**H9**	9343 (7%)
**H10**	8764 (6.6%)
**H11**	11538 (8.7%)
**H12**	6946 (5.2%)
**H13**	10055 (7.6%)
**H14**	8494 (6.4%)
**H15**	919 (0.7%)
**H16**	1257 (0.9%)
**H17**	9196 (6.9%)
**H18**	6824 (5.1%)

Supplementary Table 2: Hospital participation in various studies by cluster		
**Group***	**Study**	**Hospitals**
**1**	None	H14
**1**	RTS, S	H18
**1**	RTS, S + SEARCH	H2, H6
**1**	SEARCH	H9, H10, H11, H12, H13
**2**	None	H7, H8, H17
**2**	SEARCH	H3
**3**	RTS, S	H4, H5
**3**	RTS, S + SEARCH	H1

For peer review only

Supplementary Table 2: Hospital participation in various studies by cluster		
**Group***	**Study**	**Hospitals**
**3**	SEARCH	H16
**3**	None	H15

Note: *The group represents the collection of hospitals in CIN based on joining date. The joining date for Groups 1, 2, and 3, was September 2013, February 2014, from November 2018 respectively. Group 3 hospitals were recruited to aid in increasing hospitals involved in RTS, S and SEARCH studies.

Supplementary Table 3: Discontinuity check due to strike period

(Outcome: Adoption rate per month)

Hospital*	Term**	Estimate***	std.error	statistic	p.value
**H11**	Post Strike Period:I(Time)	2.4660123	0.4525087	5.4496458	**0.012**
**H13**	Post Strike Period:I(Time)	-0.8160995	0.3053929	-2.6722938	0.076
**H10**	Post Strike Period:I(Time)	1.3733025	0.5193376	2.6443346	0.077
**H14**	Post Strike Period:I(Time)	0.2537052	0.1188514	2.1346419	0.122
**H8**	Post Strike Period:I(Time)	-0.9380901	0.4765890	-1.9683421	0.144
**H3**	Post Strike Period:I(Time)	-0.8265731	0.4394489	-1.8809311	0.157
**H18**	Post Strike Period:I(Time)	0.3832935	0.2233908	1.7157982	0.185
**H12**	Post Strike Period:I(Time)	1.2696169	1.0149519	1.2509134	0.300
**H6**	Post Strike Period:I(Time)	-0.5999279	0.5146869	-1.1656172	0.328
**H2**	Post Strike Period:I(Time)	-0.3290568	0.2865754	-1.1482383	0.334
**H9**	Post Strike Period:I(Time)	-0.7842015	0.9577486	-0.8187968	0.473
**H17**	Post Strike Period:I(Time)	-0.0119919	0.7642561	-0.0156909	0.988

Note:

*Only hospitals recruited into CIN before the strike

**Time is in months, referencing 3 months before, and 3 months after the strike.

***Estimate is in logit units.

For peer review only

Supplementary Table 4: Complete Case Analysis						
	Pulse Oximetry Done? (n = 42776)			Oxygen Prescribed (n = 42772)		
**Predictors**	**OR**	**95% CI**	**p-value**	**OR**	**95% CI**	**p-value**
(Intercept)	0.33	0.05 – 2.02	0.231	0.12	0.05 – 0.28	<0.001
**Hospital-level factors**						
Malaria Endemicity Zone [High]	1.03	0.31 – 3.36	0.963	1.02	0.58 – 1.78	0.947
Time (in months)	1.06	1.00 – 1.11	0.033	0.98	0.95 – 1.00	0.076
**Patient-level factors**						
Ref: Patient in any study [No]						
Patient in any study [Pre-study period]ǂ	1.47	1.30 – 1.67	<0.001	0.9	0.75 – 1.07	0.229
Patient in any study [Yes]	1.15	1.00 – 1.32	0.045	0.99	0.84 – 1.17	0.949
Referral: Yes (Ref: No)	1.05	0.98 – 1.12	0.143	1.24	1.14 – 1.35	<0.001
Readmission: Yes (Ref: No)	1.05	0.97 – 1.13	0.207	0.99	0.89 – 1.11	0.93
Is a weekend admission: Yes (Ref: No)	0.99	0.94 – 1.05	0.761	1.09	1.00 – 1.17	0.04
PAR used: Yes (Ref: No)	0.7	0.46 – 1.06	0.09	0.44	0.22 – 0.88	0.02
Time (in months)* PAR used: Yes (Ref: No)	1.01	1.00 – 1.03	0.051	1.03	1.01 – 1.06	0.005
Ref: Age [> 59 months]						
Age [1-11 months]	0.75	0.64 – 0.89	0.001	0.73	0.58 – 0.94	0.013
Age [12-59 months]	0.72	0.61 – 0.84	<0.001	0.56	0.44 – 0.71	<0.001
Female: Yes (Ref: No)	1.03	0.98 – 1.08	0.229	1.03	0.96 – 1.10	0.412
Fever: Yes (Ref: No)	1.03	0.97 – 1.11	0.324	0.81	0.74 – 0.89	<0.001
Cough: Yes (Ref: No)	0.99	0.94 – 1.04	0.626	1.3	1.19 – 1.41	<0.001
Difficulty Breathing: Yes (Ref: No)	1.09	1.02 – 1.15	0.007	2.54	2.34 – 2.76	<0.001
Stridor: Yes (Ref: No)	1.01	0.86 – 1.19	0.926	0.97	0.82 – 1.15	0.739

For peer review only

Supplementary Table 4: Complete Case Analysis						
	Pulse Oximetry Done? (n = 42776)			Oxygen Prescribed (n = 42772)		
**Predictors**	**OR**	**95% CI**	**p-value**	**OR**	**95% CI**	**p-value**
Cyanosis: Yes (Ref: No)	0.91	0.69 – 1.20	0.499	1.62	1.25 – 2.10	<0.001
Indrawing: Yes (Ref: No)	1.38	1.29 – 1.48	<0.001	2.12	1.95 – 2.31	<0.001
Grunting: Yes (Ref: No)	0.88	0.81 – 0.95	0.001	1.93	1.78 – 2.08	<0.001
Acidotic Breathing: Yes (Ref: No)	0.9	0.77 – 1.04	0.161	1.31	1.13 – 1.51	<0.001
Wheezing: Yes (Ref: No)	1.08	0.97 – 1.20	0.14	0.98	0.89 – 1.09	0.769
Crackles: Yes (Ref: No)	1.06	0.99 – 1.13	0.115	1.2	1.12 – 1.30	<0.001
Tachypnoea: Yes (Ref: No)	1.09	1.03 – 1.15	0.002	1.64	1.51 – 1.77	<0.001
Alert (AVPU < A): Yes (Ref: No)	0.89	0.79 – 1.00	0.042	1.77	1.57 – 2.01	<0.001
Inability to drink: Yes (Ref: No)	0.96	0.89 – 1.03	0.226	0.76	0.69 – 0.83	<0.001
Ref: Pallor [None]						
Pallor [Mild/Moderate]	1.02	0.94 – 1.12	0.574	1.23	1.10 – 1.37	<0.001
Pallor [Severe]	0.94	0.83 – 1.07	0.336	1.18	0.99 – 1.40	0.067
Ref: WAZ [Normal]						
WAZ [Low]	0.97	0.90 – 1.04	0.388	0.95	0.86 – 1.05	0.327
WAZ [Very Low]	1	0.93 – 1.08	0.97	0.9	0.82 – 0.99	0.033
Slow Capillary Refill: Yes (Ref: No)	0.76	0.61 – 0.94	0.013	0.8	0.60 – 1.06	0.119
Ref: Hypoxic [Unknown]						
Hypoxic [No]				0.54	0.50 – 0.59	<0.001
Hypoxic [Yes]				1.71	1.53 – 1.92	<0.001
Random effects						
σ^2	3.29			3.29		
τ_{00}	12.40 Hospital			0.23 Hospital		
τ_{11}	0.01 Hospital.Time			0.00 Hospital.Time		
ρ_{01}	-0.96 Hospital			-0.11 Hospital		
**ICC**	0.57			0.23		
**N**	17 Hospital			17 Hospital		

Supplementary Table 4: Complete Case Analysis						
	Pulse Oximetry Done? (n = 42776)			Oxygen Prescribed (n = 42772)		
**Predictors**	**OR**	**95% CI**	**p-value**	**OR**	**95% CI**	**p-value**
**Marginal R^2 / Conditional R^2**	0.144/ 0.631			0.271 / 0.442		

Supplementary Table 5: Oxygen prescription for $SpO_2 \geq 90\%$ OR SpO_2 Unknown						
	MICE model*			Complete Case Analysis**		
**Predictors**	**OR**	**95% CI**	**p-value**	**OR**	**95% CI**	**p-value**
(Intercept)	0.04	0.02 - 0.08	<0.001	0.08	0.03 – 0.19	<0.001
**Hospital-level factors**						
Malaria Endemicity Zone [High]	1.61	0.9 - 2.87	0.106	1.12	0.61 – 2.04	0.719
Time (in months)	1.01	1 - 1.02	0.152	0.97	0.95 – 1.00	0.032
**Patient-level factors**						
Ref: Patient in any study [No]						
Patient in any study [Pre-study period]‡	0.99	0.86 - 1.12	0.824	0.88	0.73 – 1.06	0.168
Patient in any study [Yes]	0.94	0.83 - 1.07	0.349	0.92	0.78 – 1.10	0.369
Referral: Yes (Ref: No)	1.18	1.11 - 1.26	<0.001	1.28	1.17 – 1.40	<0.001
Readmission: Yes (Ref: No)	1.07	1 - 1.16	0.066	1.01	0.90 – 1.14	0.846
Is a weekend admission: Yes (Ref: No)	1.08	1.02 - 1.14	0.005	1.11	1.02 – 1.21	0.014
PAR used: Yes (Ref: No)	1.18	0.96 - 1.44	0.120	0.46	0.22 – 0.96	0.039
Time (in months)*PAR used: Yes (Ref: No)	1	1 - 1.01	0.560	1.03	1.01 – 1.06	0.013
Ref: Age [> 59 months]						
Age [< 1 month]	1.9	1.56 - 2.3	<0.001			

For peer review only

Supplementary Table 5: Oxygen prescription for $SpO_2 \geq 90\%$ OR SpO_2 Unknown						
	MICE model*			Complete Case Analysis**		
**Predictors**	**OR**	**95% CI**	**p-value**	**OR**	**95% CI**	**p-value**
Age [1-11 months]	1.42	1.31 - 1.54	<0.001	0.79	0.61 – 1.03	0.078
Age [12-59 months]	1.06	0.98 - 1.15	0.163	0.59	0.46 – 0.76	<0.001
Female: Yes (Ref: No)	1.03	0.98 - 1.07	0.288	1.04	0.97 – 1.12	0.256
Fever: Yes (Ref: No)	0.76	0.72 - 0.81	<0.001	0.8	0.72 – 0.89	<0.001
Cough: Yes (Ref: No)	1.36	1.28 - 1.44	<0.001	1.3	1.19 – 1.42	<0.001
Difficulty Breathing: Yes (Ref: No)	2.88	2.72 - 3.05	<0.001	2.66	2.43 – 2.91	<0.001
Stridor: Yes (Ref: No)	1.12	1.01 - 1.26	0.039	0.99	0.83 – 1.17	0.865
Cyanosis: Yes (Ref: No)	1.61	1.36 - 1.91	<0.001	1.94	1.48 – 2.56	<0.001
Indrawing: Yes (Ref: No)	2.48	2.34 - 2.63	<0.001	2.27	2.07 – 2.48	<0.001
Grunting: Yes (Ref: No)	2.03	1.92 - 2.15	<0.001	2.09	1.92 – 2.27	<0.001
Acidotic Breathing: Yes (Ref: No)	1.37	1.24 - 1.51	<0.001	1.38	1.19 – 1.61	<0.001
Wheezing: Yes (Ref: No)	1.17	1.08 - 1.26	<0.001	1.04	0.93 – 1.16	0.542
Crackles: Yes (Ref: No)	1.27	1.2 - 1.34	<0.001	1.21	1.12 – 1.32	<0.001
Tachypnoea: Yes (Ref: No)	1.63	1.54 - 1.72	<0.001	1.7	1.56 – 1.85	<0.001
Alert (AVPU < A): Yes (Ref: No)	2.02	1.86 - 2.18	<0.001	1.83	1.60 – 2.08	<0.001
Inability to drink: Yes (Ref: No)	0.8	0.75 - 0.85	<0.001	0.76	0.69 – 0.84	<0.001
Ref: Pallor [None]						
Pallor [Mild/Moderate]	1.11	1.04 - 1.2	<0.001	1.26	1.12 – 1.41	<0.001
Pallor [Severe]	1.34	1.22 - 1.46	<0.001	1.14	0.95 – 1.38	0.153
Ref: WAZ [Normal]						
WAZ [Low]	1	0.93 - 1.07	0.952	0.97	0.87 – 1.08	0.596
WAZ [Very Low]	0.96	0.89 - 1.03	0.273	0.92	0.83 – 1.02	0.122
Slow Capillary Refill: Yes (Ref: No)	1.03	0.88 - 1.2	0.708	0.87	0.65 – 1.18	0.371

Note: ******n=123,952; 4275 cases omitted from MICE analysis because of missing outcome (Figure 4)*

*******n=40,491*

For peer review only

Supplementary Table 6: Admission diagnoses for patients getting oxygen with pulse oximetry ≥ 90% at admission.	
**Diagnosis**	**Documented Cases, N (%)**
Pneumonia	18668 (34.14%)
Malaria	16559 (30.28%)
Dehydration	10641 (19.46%)
Diarrhoea	10592 (19.37%)
Anaemia	8018 (14.66%)
Meningitis	5875 (10.74%)
Malnutrition	5351 (9.78%)
Sickle Cell	2380 (4.35%)
Asthma	1240 (2.27%)
Rickets	1084 (1.98%)
TB	907 (1.6s6%)
Sepsis	708 (1.29%)
HIV	716 (1.31%)

For peer review only

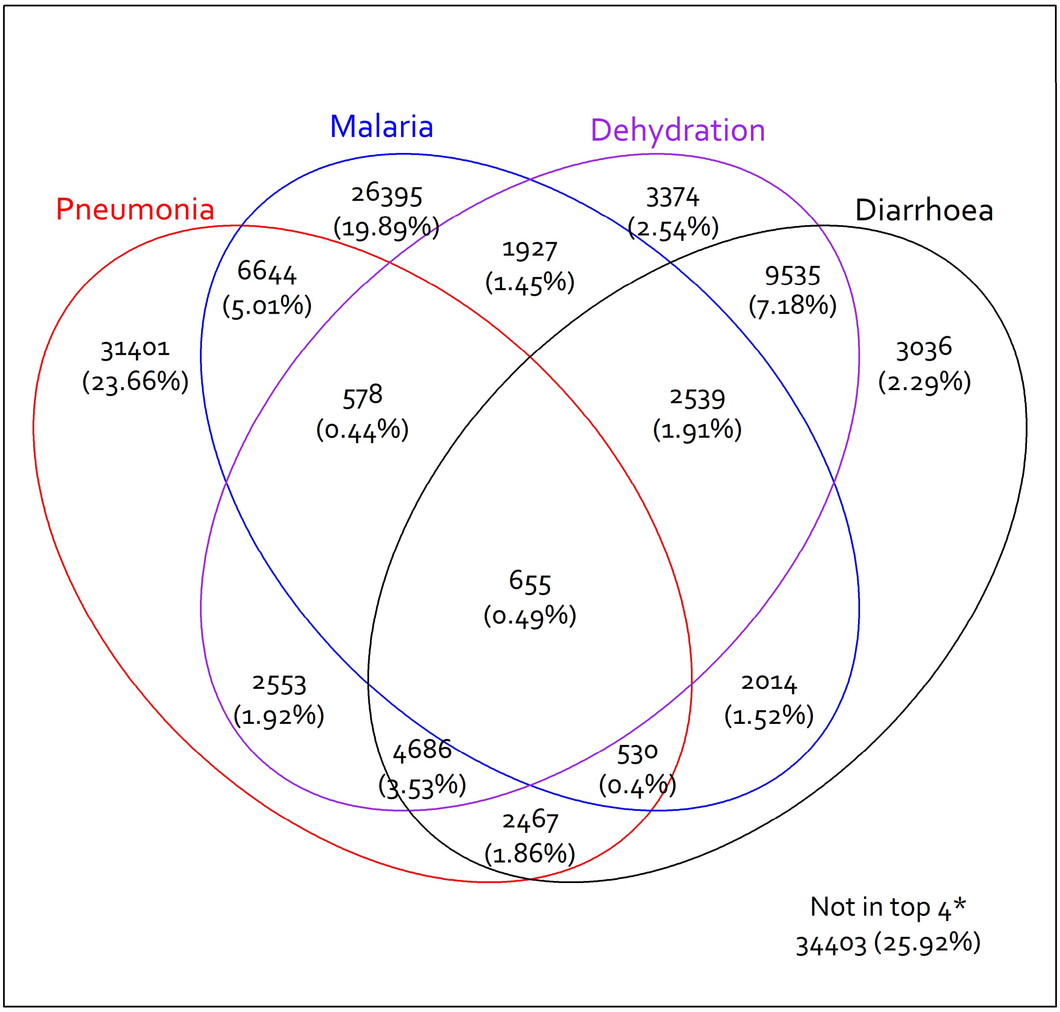

Supplementary Figure 1: Common admission diagnoses in CIN hospitals.

From the linear growth-curve model, the random intercept conveys the hospital's average pulse oximetry adoption rate over the time period in months it has participated in CIN. The random slope conveys the hospital's average monthly increase or decrease in adoption rate over the time period they have been part of the CIN. The baseline for the random intercept and slope in the growth curve model is the overall level of the response which is allowed to vary between hospitals after controlling for covariates.

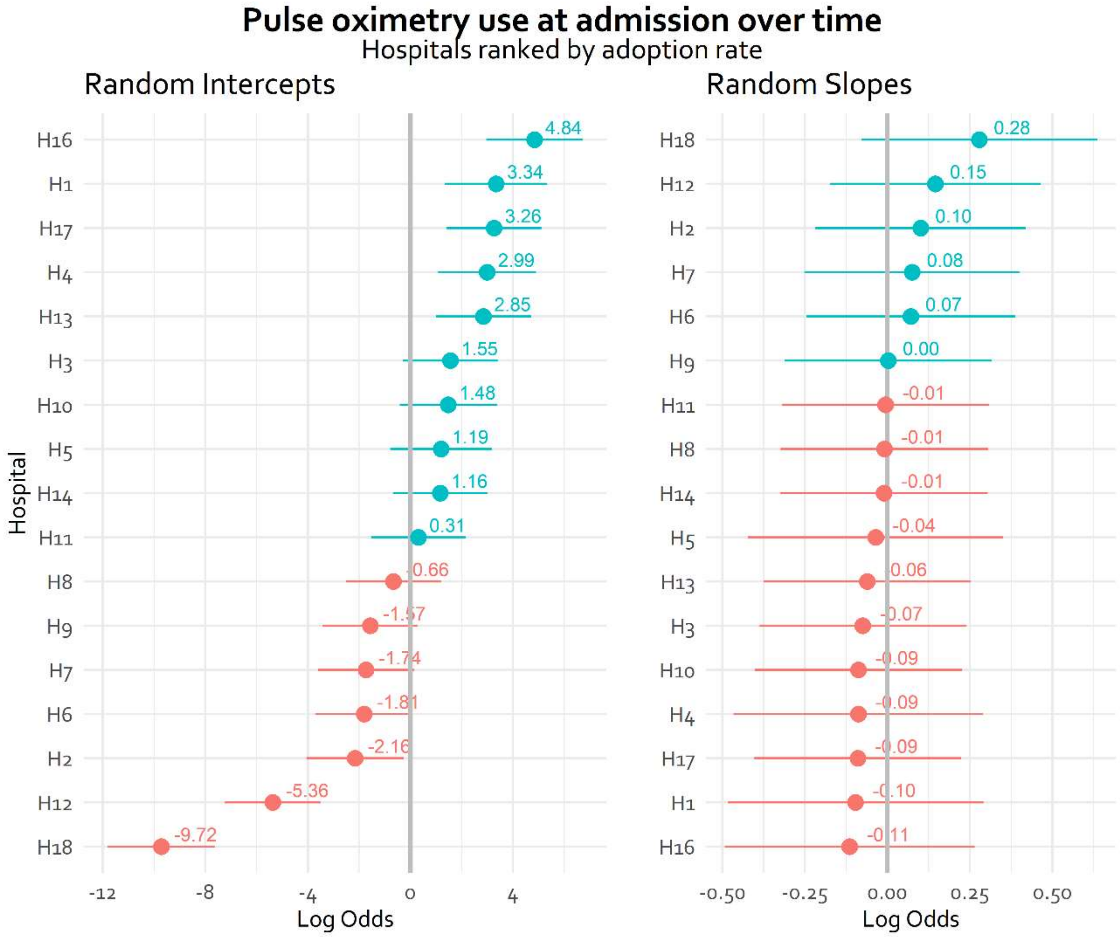

Supplementary Figure 2: Ranking of hospitals pulse oximetry use at admission

review only

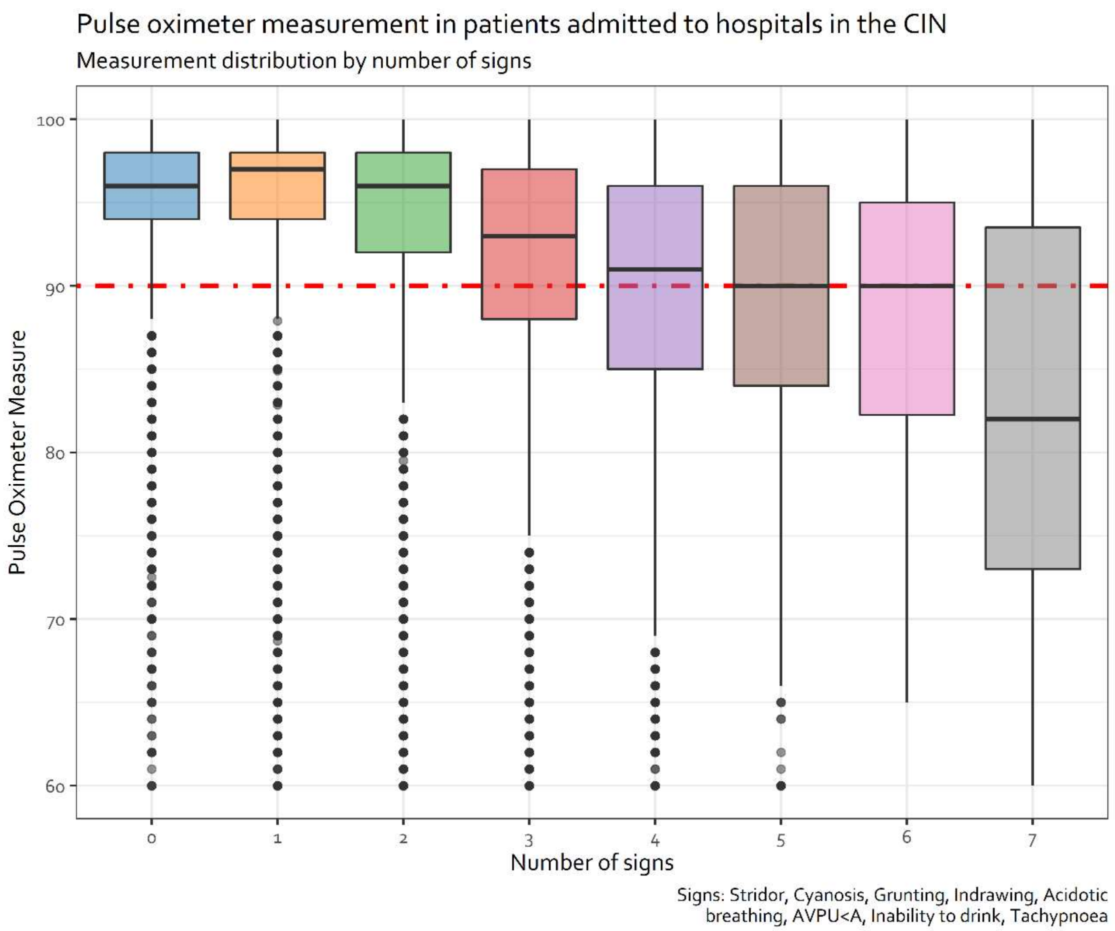

Supplementary Figure 3: Oxygen saturation measure by the number of respiratory distress symptoms

view only

STROBE Statement—checklist of items that should be included in reports of observational studies

	Item No	Recommendation	Page No
**Title and abstract**	1	(*a*) Indicate the study's design with a commonly used term in the title or the abstract	1
		(*b*) Provide in the abstract an informative and balanced summary of what was done and what was found	1
**Introduction**			
Background/rationale	2	Explain the scientific background and rationale for the investigation being reported	92-114
Objectives	3	State specific objectives, including any prespecified hypotheses	111-114, 213-216
**Methods**			
Study design	4	Present key elements of study design early in the paper	127-152
Setting	5	Describe the setting, locations, and relevant dates, including periods of recruitment, exposure, follow-up, and data collection	127-152
Participants	6	(*a*) *Cohort study*—Give the eligibility criteria, and the sources and methods of selection of participants. Describe methods of follow-up<br>*Case-control study*—Give the eligibility criteria, and the sources and methods of case ascertainment and control selection. Give the rationale for the choice of cases and controls<br>*Cross-sectional study*—Give the eligibility criteria, and the sources and methods of selection of participants	153-163
		(*b*) *Cohort study*—For matched studies, give matching criteria and number of exposed and unexposed<br>*Case-control study*—For matched studies, give matching criteria and the number of controls per case	
Variables	7	Clearly define all outcomes, exposures, predictors, potential confounders, and effect modifiers. Give diagnostic criteria, if applicable	179-196
Data sources/ measurement	8*	For each variable of interest, give sources of data and details of methods of assessment (measurement). Describe comparability of assessment methods if there is more than one group	164-178
Bias	9	Describe any efforts to address potential sources of bias	225-233
Study size	10	Explain how the study size was arrived at	153-163
Quantitative variables	11	Explain how quantitative variables were handled in the analyses. If applicable, describe which groupings were chosen and why	189-196
Statistical methods	12	(*a*) Describe all statistical methods, including those used to control for confounding	198-228
		(*b*) Describe any methods used to examine subgroups and interactions	210-216
		(*c*) Explain how missing data were addressed	229-233

For peer review only - http://bmjopen.bmj.com/site/about/guidelines.xhtml

(*d*) *Cohort study*—If applicable, explain how loss to follow-up was addressed<br>*Case-control study*—If applicable, explain how matching of cases and controls was addressed<br>*Cross-sectional study*—If applicable, describe analytical methods taking account of sampling strategy	217-228
(*e*) Describe any sensitivity analyses	

Continued on next page

For peer review only

For peer review only - http://bmjopen.bmj.com/site/about/guidelines.xhtml

**Results**			
Participants	13*	(a) Report numbers of individuals at each stage of study—eg numbers potentially eligible, examined for eligibility, confirmed eligible, included in the study, completing follow-up, and analysed	235 - 241
		(b) Give reasons for non-participation at each stage	Figure 1
		(c) Consider use of a flow diagram	Figure 1
Descriptive data	14*	(a) Give characteristics of study participants (eg demographic, clinical, social) and information on exposures and potential confounders	235 – 241, 246 - 287
		(b) Indicate number of participants with missing data for each variable of interest	Table 2
		(c) *Cohort study*—Summarise follow-up time (eg, average and total amount)	
Outcome data	15*	*Cohort study*—Report numbers of outcome events or summary measures over time	Table 2, Figures 2-3
		Case-control study—Report numbers in each exposure category, or summary measures of exposure	
		Cross-sectional study—Report numbers of outcome events or summary measures	246-313
Main results	16	(*a*) Give unadjusted estimates and, if applicable, confounder-adjusted estimates and their precision (eg, 95% confidence interval). Make clear which confounders were adjusted for and why they were included	314-377
		(*b*) Report category boundaries when continuous variables were categorized	299-300
		(*c*) If relevant, consider translating estimates of relative risk into absolute risk for a meaningful time period	
Other analyses	17	Report other analyses done—eg analyses of subgroups and interactions, and sensitivity analyses	261- 267, Supplementary Tables 3 - 5, Table 3
**Discussion**			
Key results	18	Summarise key results with reference to study objectives	379-386
Limitations	19	Discuss limitations of the study, taking into account sources of potential bias or imprecision. Discuss both direction and magnitude of any potential bias	452-472
Interpretation	20	Give a cautious overall interpretation of results considering objectives, limitations, multiplicity of analyses, results from similar studies, and other relevant evidence	386-450
Generalisability	21	Discuss the generalisability (external validity) of the study results	452-462
**Other information**			
Funding	22	Give the source of funding and the role of the funders for the present study and, if applicable, for the original study on which the present article is based	533-547

*Give information separately for cases and controls in case-control studies and, if applicable, for exposed and unexposed groups in cohort and cross-sectional studies.

For peer review only

Note: An Explanation and Elaboration article discusses each checklist item and gives methodological background and published examples of transparent reporting. The STROBE checklist is best used in conjunction with this article (freely available on the Web sites of PLoS Medicine at http://www.plosmedicine.org/, Annals of Internal Medicine at http://www.annals.org/, and Epidemiology at http://www.epidem.com/). Information on the STROBE Initiative is available at www.strobe-statement.org.

For peer review only

BMJ Open

Pulse oximetry adoption and oxygen orders at paediatric admission over 7 years in Kenya: A multi-hospital retrospective cohort study

Journal:	*BMJ Open*
Manuscript ID	bmjopen-2021-050995.R1
Article Type:	Original research
Date Submitted by the Author:	09-Jun-2021
Complete List of Authors:	Tuti, Timothy; University of Oxford, Kellogg College; KEMRI-Wellcome Trust Research Programme, Health Services Unit<br>Aluvaala, Jalemba ; Kenya Medical Research Institute/Wellcome Trust Research Programme, Health Services Unit; University of Nairobi, Department of Paediatrics and Child Health<br>Akech, Samuel; KEMRI-Wellcome Trust Research Programme Nairobi<br>Agweyu, Ambrose; KEMRI-Wellcome Trust Research Programme Nairobi, Health Services Unit<br>Irimu, Grace ; Kenya Medical Research Institute, Health Services Unit; University of Nairobi College of Health Sciences, Department of Paediatrics and Child Health<br>English, Mike; University of Oxford, Nuffield Department of Medicine and Department of Paediatrics; KEMRI-Wellcome Trust Research Programme Nairobi, Health Services Unit
**Primary Subject Heading**:	Paediatrics
Secondary Subject Heading:	Epidemiology, Global health, Health services research, Paediatrics
Keywords:	Quality in health care < HEALTH SERVICES ADMINISTRATION & MANAGEMENT, PAEDIATRICS, AUDIT, EPIDEMIOLOGY, Protocols & guidelines < HEALTH SERVICES ADMINISTRATION & MANAGEMENT, Tropical medicine < INFECTIOUS DISEASES

SCHOLARONE™
Manuscripts

I, the Submitting Author has the right to grant and does grant on behalf of all authors of the Work (as defined in the below author licence), an exclusive licence and/or a non-exclusive licence for contributions from authors who are: i) UK Crown employees; ii) where BMJ has agreed a CC-BY licence shall apply, and/or iii) in accordance with the terms applicable for US Federal Government officers or employees acting as part of their official duties; on a worldwide, perpetual, irrevocable, royalty-free basis to BMJ Publishing Group Ltd ("BMJ") its licensees and where the relevant Journal is co-owned by BMJ to the co-owners of the Journal, to publish the Work in this journal and any other BMJ products and to exploit all rights, as set out in our licence.

The Submitting Author accepts and understands that any supply made under these terms is made by BMJ to the Submitting Author unless you are acting as an employee on behalf of your employer or a postgraduate student of an affiliated institution which is paying any applicable article publishing charge ("APC") for Open Access articles. Where the Submitting Author wishes to make the Work available on an Open Access basis (and intends to pay the relevant APC), the terms of reuse of such Open Access shall be governed by a Creative Commons licence – details of these licences and which Creative Commons licence will apply to this Work are set out in our licence referred to above.

Other than as permitted in any relevant BMJ Author's Self Archiving Policies, I confirm this Work has not been accepted for publication elsewhere, is not being considered for publication elsewhere and does not duplicate material already published. I confirm all authors consent to publication of this Work and authorise the granting of this licence.

For peer review only

Pulse oximetry adoption and oxygen orders at paediatric admission over 7 years in Kenya: A multi-hospital retrospective cohort study

Timothy Tuti[1*], Jalemba Aluvaala[1,2], Samuel Akech[1], Ambrose Agweyu[1], Grace Irimu[1,2], Mike English[1,3] on behalf of the Clinical Information Network,

[1] Health Services Unit, KEMRI-Wellcome Trust Research Programme

[2] Department of Paediatrics and Child Health, University of Nairobi, Nairobi, Kenya

[3] Oxford Centre for Global Health Research, University of Oxford, Oxford, UK

*Corresponding Author: P.O. Box 43640, Nairobi, Kenya; Email: timothy.tuti@kellogg.ox.ac.uk

For peer review only

Abstract:

Objectives: To characterise adoption and explore specific clinical and patient factors that might influence pulse oximetry and oxygen use in low- and middle-income countries (LMICs) over time; To highlight useful considerations for entities working on programs to improve access to pulse oximetry and oxygen.

Design: A multi-hospital retrospective cohort study

Settings: All admissions (n=132737) to paediatric wards of 18 purposely selected public hospitals in Kenya that joined a Clinical Information Network (CIN) between March 2014 and December 2020.

Outcomes: Pulse oximetry use and oxygen prescription on admission; We performed growth-curve modelling to investigate the association of patient factors with study outcomes over time while adjusting for hospital factors.

Results: Overall, pulse oximetry was used in 48.8% (64722/132737) of all admission cases. Use rose on average with each month of participation in the CIN (OR: 1.11, 95% CI 1.05–1.18) but patterns of adoption were highly variable across hospitals suggesting important factors at hospital level influence

use of pulse oximetry. Of those with pulse oximetry measurement, 7% (4510 /64722) had hypoxaemia (SpO_2 <90%). Across the same period, 8.6% (11428/132737) had oxygen prescribed but in 87%, pulse oximetry was either not done or the hypoxemia threshold (SpO_2 <90%) was not met. Lower chest-wall indrawing and other respiratory symptoms were associated with pulse oximetry use at admission and were also associated with oxygen prescription in the absence of pulse oximetry or hypoxemia.

Conclusion: The adoption of pulse oximetry recommended in international guidelines for assessing children with severe illness has been slow and erratic, reflecting system and organisational weaknesses. Most oxygen orders at admission seem driven by clinical and situational factors other than the presence of hypoxemia. Programmes aiming to implement pulse oximetry and oxygen systems will likely need a long-term vision to promote adoption, guideline development and adherence and continuously examine impact.

Keywords: Quality Improvement, Health Systems, Paediatric Care, Diagnostics, Evaluation, Hospital

Word Count: 5,120

Strengths and limitations

- The data presented are a rare example of long-term tracking of hospital practice in a routine LMICs setting. They present a useful illustration of the slow and sometimes erratic process of pulse oximetry adoption in LMIC hospitals that has broader implications for thinking on technology adoption.
- We employ a complex modelling framework to explore patient- and hospital- level factors influencing outcomes of interest at admission (pulse oximetry done, and, oxygen prescribed) over time. These accommodate and account for between hospital variability, within hospital non-linear trends in the outcomes over time and entry into the CIN at different time points.
- Many factors ranging from local procurement or equipment donations, to national clinical guidelines and protocols dissemination, and hospital’s participation in specific studies might

For peer review only

For peer review only - http://bmjopen.bmj.com/site/about/guidelines.xhtml

influence the adoption of pulse oximetry and use of oxygen we describe; There is limited data to characterise their influence on adoption.

- We have no contemporaneous data from Kenyan settings that do not belong to the CIN for comparison and most other studies exploring the use of pulse oximetry are essentially cross-sectional.

Introduction

Background

Compared to clinical signs (e.g. central cyanosis, indrawing), pulse oximetry is felt to be easy to use and posited to be at least 20% more accurate in identifying children at risk of hypoxemia (defined as a blood oxygen saturation of <90%) [1, 2]. As pulse oximetry is an inexpensive tool it is now recommended for guiding the assessment of illness severity and treatment for respiratory illnesses, including increasing emphasis on its role for screening children with respiratory illness in primary care in LMIC [1, 2]. As hypoxemia may be found in all severe childhood illnesses such as sepsis, meningitis, severe acute malnutrition and malaria its use is widely recommended as a screening tool in all paediatric admissions as a fifth vital sign. The advent of COVID-19 has further invigorated programmatic efforts to scale up pulse oximetry together with improved oxygen delivery systems [3-9].

Despite being recommended for many years, there is little long-term data on the adoption of pulse oximetry in LMIC, even from hospitals. Data on use during the COVID-19 pandemic are also lacking from such settings as are large studies from routine settings on whether pulse oximeter readings influence the use of oxygen. We and others have described that adoption is undermined by system factors such as inadequate supply and repair of oximeters and if healthcare workers have insufficient training on when, how, and why to use them and interpret their results [9, 10]. This compounds wider and similar problems in managing oxygen systems themselves [11]. Our earlier work used data from 7 Kenyan hospitals between September 2013 and February 2016. Here we update and extend these analyses to include data from paediatric admissions in a growing network of Kenyan hospitals before

For peer review only

and after prolonged national health workers strikes in 2017, including data obtained during the COVID-19 pandemic [12]. The objective of this study was to characterise adoption and explore specific patient, clinical and hospital factors that might influence pulse oximetry and oxygen use and highlight useful considerations for entities working on programs to improve access to pulse oximetry and oxygen [13-15].

Methods

Ethics and Reporting

The reporting of this observational study follows the Strengthening of reporting of observational studies in epidemiology (STROBE) statement [16]. The Scientific and Ethics Review Unit of the Kenya Medical Research Institute (KEMRI) approved the collection of the de-identified data that provides the basis for this study as part of the Clinical Information Network (CIN). The CIN is run in partnership with the Ministry of Health and participating hospitals with aims to improve the quality of routine paediatric hospital data for use in improvement activities, observational and interventional research [17, 18]. Individual consent for access to de-identified patient data was not required.

Patient and Public Involvement

No patients were involved in the design, conduct, reporting, or dissemination plans of our research except through the KEMRI ethical review process where they have representatives.

Study design and setting

From a broad context perspective, in many LMICs including Kenya, hospital management and monitoring systems are weak, with major human and material resource constraints. These challenges affect hospitals' delivery of inpatient maternal, surgical, and adult medical care as well as paediatric and neonatal care. Consequently, there is very limited organisational and resource slack to mobilise for any new purpose. Interventions such as oximetry and oxygen seeking to achieve large scale change must therefore either consider how to mobilise new resources or consider what is achievable with limited resources [19, 20]. We have described the broader context of the Kenyan healthcare system with reference to the paediatric burden of disease in great detail elsewhere [19, 20].

For peer review only - http://bmjopen.bmj.com/site/about/guidelines.xhtml

We report a retrospective cohort study of 18 public hospitals in Kenya largely providing first-referral level care predominantly admitting patients that present directly to the facility, and purposefully selected to be of at least moderate size and representative of different malaria transmission zones. Hospitals joined the CIN at different calendar time points between 2013 and 2017. Few of their patients may be formally referred from primacy care facilities but there are few functional referral mechanisms such as ambulance systems [21]. Pulse oximetry has generally not been available outside hospitals in the public sector [22]. Pneumonia is the major killer of children across the country except in settings where malaria is highly endemic (9/18 hospitals in this study) [21].

The hospitals receive 3 monthly clinical audit and feedback reports on the quality of care they provide for common conditions [23]. Paediatric team leaders (paediatricians and nurses) met face to face once or twice annually until 2019 (before the pandemic) to discuss these reports and how to improve clinical care. From 2018, participation in more specific research studies was also discussed in meetings with hospitals. This resulted in two studies being initiated in subsets of the CIN hospitals which might have influenced the adoption of pulse oximetry. Hospital participation in these studies was not mutually exclusive: a hospital could be part of one, both or none of the research studies. Not all hospitals recruited in the same study were recruited at the same time. Summary details of these studies are provided in Table 1 with greater detail on which hospitals participated in the studies provided in Supplementary Tables 1 and 2.

For peer review only

Table 1: Description of studies undertaken in CIN hospitals

**Study name**	**Study site selection rationale and period**	**Goal**	**Extra resources provided to hospitals (H) by projects***	**Extra notes**
**RTS, S/AS01 trial: (ClinicalTrials.gov Identifier: NCT03806465)**	Study start: December 2018<br><br>Site Selection: By MoH/WHO, selecting high volume public hospitals in	The evaluation of the feasibility, safety and impact of the RTS, S/AS01 malaria vaccine in paediatric cases in Kenya	One extra healthcare worker provided per paediatric ward.<br><br>Extra pulse oximetry equipment	Clinician role: Promote compliance with guidelines for meningitis and malaria including complete assessment and investigations including the performance of lumbar punctures, and consenting participants for storage of cerebrospinal fluid (CSF) storage

For peer review only - http://bmjopen.bmj.com/site/about/guidelines.xhtml

	regions getting the vaccine.		provided per ward:<br>• One item: H4	
**SEARCH trial (ClinicalTrials.gov Identifier: NCT04041791)**	Started in two phases:<br>• March 2019 Hospitals: H3, H9, H12, H13, H16<br>• June 2019 Hospitals: H1, H2, H6, H10, H11<br><br>Site selection: From the expected size of pneumonia population, the geographical balance of hospitals and support from local hospital teams	Pragmatic factorial individually randomized controlled trial to compare alternative antibiotics and alternative modes of fluid therapy for children with severe pneumonia	One extra healthcare worker provided per participating hospital.<br><br>Extra pulse oximetry equipment provided to the hospital:<br>• Two items: H3, H6, H9, H10, H11, H12, H13, H16<br>• One item: H1, H2	Clinician role: Promote compliance with the study protocol, including completing patient assessment, investigations and diagnoses. Ensure appropriate administration of study interventions.<br><br>Recruitment of study participants was suspended on the 8th of April 2020 due to COVID-19 pandemic.<br><br>Adult inpatient surveillance in addition to paediatric surveillance began on the 4th of May 2020.

Note:
H15 got one pulse oximeter in June 2020 even though it was not part of any study
** We were not able to track and record introduction of pulse oximeters procured directly by hospitals or other sources, or those personally owned by healthcare workers, nor ability to repair existing pulse oximeters when required.*

Recording of pulse oximetry values has been included as part of a structured Paediatric Admission Record (PAR) [24] used since 2013. Hospitals joining the CIN agree to provide the PAR themselves and promote its use – the purpose of the PAR is to prompt admitting clinicians to fully assess children and rapidly document their findings [24]. Emergency Triage Assessment and Treatment plus Admission Care (ETAT+) training [25] has been used in Kenya since 2008 and was adapted in 2013 so that pulse oximetry was a recommended part of the assessment in all sick children and especially those with danger signs. Many of the junior and senior medical staff in CIN hospitals would have received ETAT+ training (e.g. as an undergraduate or postgraduate course or as in-service training) [25]. Additionally, the Basic Paediatric Protocols that are widely disseminated have specifically referred to the use of

For peer review only

pulse oximetry – if available – for all pneumonia cases since 2016 [26]. The promotion of PAR use by hospitals, adaption and scale up of ETAT+ training, and dissemination of the basic paediatric protocols to clinicians nationally are posited to have a system-wide effect on the adoption of pulse oximetry use.

Study participants

All medical admissions to paediatric wards (ages 0 to 13 years), but not those admitted to specific newborn units, to the selected hospitals between February 1st 2014, or the date at which a hospital joined CIN, and December 31st 2020 were eligible for inclusion in this study. We excluded children whose admission or discharge dates were missing or improbable (e.g. discharge date is earlier than admission date), and those whose admission fell within 2017, a period of prolonged health worker strikes that resulted in major disruption to health care delivery [12]. The expectation was that all would have pulse oximetry performed at admission given they were sick enough to warrant inpatient care but amongst these we also report the proportion of children with danger signs comprising any level of altered consciousness or with recorded signs of respiratory distress especially deserving of pulse oximetry [10, 26, 27].

Data sources and management

Methods of collection and cleaning of data in the CIN are reported in detail elsewhere [28]. Clinical data for paediatric admissions to the hospitals within the CIN are captured through PAR forms [27] that are approved by the Ministry of Health. The PAR prompts the clinician with a checklist of fields including patient biodata, clinical assessment, admission and discharge diagnoses, treatments, and to record outcome (survival or death). The CIN supports one data clerk in each hospital to extract data from paper medical records, nursing charts, treatment charts, and available laboratory reports each day after children's discharge into the primary data collection tool developed in Research Electronic Data Capture (REDCap). Automated error checking happens at the point of entry by daily review, every week centrally and both are complemented by regular external data quality assurance reviews [28]. A minimal dataset – which is unsuitable for these pulse oximetry analyses - is collected for (1) all

For peer review only - http://bmjopen.bmj.com/site/about/guidelines.xhtml

For peer review only

admissions with a burn or a surgical diagnosis to the paediatric ward(s), (2) admissions during major holiday breaks, (3) admissions when the data clerk was on leave, and (4) on a random selection of records in hospitals where the workload is very high. This process is explained in detail elsewhere [28, 29].

Descriptive analysis

To characterise adoption of pulse oximetry at admission over time we use pooled data from the period February 2014 to November 2018 (excluding 2017) from 13 network hospitals. From December 2018 data were available from 17 hospitals after 5 joined the CIN linked to new studies, and one (H7) exited. To explore patterns in more detail we employed two approaches: i) for the period from December 2018 to December 2020 we plot data pooled for hospital sub-groups depending on which research studies they were taking part in as these might influence pulse oximetry use; ii) We plot adoption within each hospital for the number of months it was part of the network, again excluding 2017 where relevant. This latter approach was used to reveal the extent of variability in adoption at the hospital level.

For the individual patient population, we tabulated and summarised categorical data as proportions, with continuous variables reported as medians (IQR) if they were not normally distributed. From the included paediatric admission patient population data, we report the prevalence of important clinical features such as respiratory distress symptoms (e.g. central cyanosis, indrawing, grunting, and difficulty breathing), circulatory symptoms (e.g. slow capillary refill, less than three seconds), level of consciousness, and whether the patient was being readmitted or referred to the hospital. We also report the prevalence of summary clinical features such as having any sign of respiratory distress and any danger sign and the proportion for whom oxygen was ordered as a treatment at admission.

Statistical methods

To examine two binary outcomes, (1) pulse oximetry used and, (2) oxygen ordered at admission, we modelled individual patient data. Our key explanatory variable was time as part of (or exposure to) the CIN, computed as the number of months since joining the CIN for a specific hospital. This allowed

For peer review only - http://bmjopen.bmj.com/site/about/guidelines.xhtml

associations with these outcomes to be explored in hospitals while accounting for clustering by both hospital and time in months since the hospital joined the CIN; the analysis of pulse oximetry use employs a much larger dataset to extend our earlier findings [10].

Predictors included in our models were patients' age and sex, and the record of presence or absence of respiratory signs and symptoms, circulatory symptoms, level of consciousness, whether the patient was being readmitted or referred to the hospital. At the hospital level, we included terms for their specific identity and malaria endemicity zone. Hospital participation in one of the four research study sub-groups was included as a covariable at the patient level to take account of possible effects occurring only after a hospital joined specific studies.

We modelled whether oxygen was prescribed at admission with an interest in whether patients with a recorded pulse oximetry value (SpO_2) <90% at admission would be started on oxygen therapy. Where the pulse oximetry value at admission was missing, based on findings from a previous CIN study, the assumption was that the clinician did not have the information [10]. The analysis therefore explored (1) whether pulse oximetry values (SpO_2 <90%) were associated with oxygen prescription and (2) in the absence of a pulse oximetry value <90%, which clinical signs are associated with the prescription of oxygen therapy.

We included the variables of interest in hierarchical multivariate logistic regression models, with a random intercept for each hospital and a random slope for the time within hospital. This model specification commonly referred to as a growth curve model, allowed the explanatory variable of time to have a different effect for each hospital. A *growth curve model* typically refers to statistical methods that allow, in our case, the estimation of inter-hospital variability in intra-hospital patterns of change over time [30]. Our approach to the growth-curve model fitting is within the multilevel modelling framework, with patients nested in hospitals nested in time points [30]. Different ways of specifying the growth-curve model using a multilevel modelling framework are explained in detail elsewhere [31]. Before embarking on the growth-curve modelling we examined for each hospital whether the

For peer review only - http://bmjopen.bmj.com/site/about/guidelines.xhtml

2017 strike resulted in a significant discontinuity in pulse oximetry use to check if treating time as months in the CIN, a continuous variable, instead of as calendar time was justifiable given the strike caused a 12-month data gap.

Where there was missing patient-level data, we applied fully conditional specification (FCS) multivariate imputation by chained equations (MICE) [32] to generate imputed datasets under the missing at random (MAR) assumption to allow us to analyse all eligible patients. Previous studies indicate that MAR is a reasonable approach for CIN data [33]. We contrasted findings using imputation with the findings of the complete case analysis.

Results

Figure 1 depicts the study population inclusion process. Out of the 179991 paediatric admissions to CIN hospitals, 132737 (73.7%) were eligible for analysis. Most exclusions were because an admission was randomly sampled for minimum data collection (n=33074, 70% of the exclusions) or fell in the strike year (n=8787, 19% exclusions). Of the population included in the analysis, 113196/132737 (86.1%) demonstrated at least one sign indicating increased risk of hypoxemia (respiratory distress symptoms or altered consciousness, or age below one month). Given this high proportion, we report results based on data from all eligible admissions (Figure 1).

Figure 1: Flow chart of the inclusion criteria

Descriptive findings

From February 2014 to December 2016, on average, there was a steady improvement in pulse oximetry adoption at admission in the 13 hospitals from between 10%-20% in 2014 to between 50%-70% at the end of 2016 (Figure 2), even though no pulse oximetry devices were provided through CIN. Following the strikes in 2017, from January 2018 when services resumed up until November 2018, average pulse oximetry adoption in these 13 sites seemed to stabilise at around 50%. By December

For peer review only - http://bmjopen.bmj.com/site/about/guidelines.xhtml

2018, one hospital exited and 5 hospitals joined CIN and different research studies were initiated in subsets of hospitals (Table 1, Figure 2 and Supplementary Table 2). Pulse oximetry adoption ranged from an average of 40% to almost 100% across these subsets (Figure 2).

Figure 2: Average pulse oximetry use in the CIN hospitals over time. The number of hospitals in each study covers both presently involved and those joining the CIN later: There are 13 hospitals in the period between Feb 2014 – November 2018, with the lower panel representing transition to 18 hospitals by adding 5 hospitals and research studies beginning. The 5 new hospitals' distribution among the studies in the following way: RTS,S: 2/5; RTS,S + SEARCH: 1/5; SEARCH: 1/5; None: 1/5.

Exploring each hospital's adoption trajectory illustrates in many a general improvement in adoption throughout their participation in CIN but also examples of adoption failure (Figure 3). Figure 3 tends to confirm as depicted in Figure 2, that despite a strike of almost one year there was minimal evidence of discontinuity in hospitals' pattern of adoption from 2016 to 2018. The lack of evidence for discontinuity (supported by additional analyses in Supplementary Table 3) supports our treatment of time as 'months in CIN' as a continuous variable in place of calendar time in Figure 2 and 3 and subsequent models.

From Figure 3, 8 of the 13 hospitals recruited into the CIN by February 2014 had no use of pulse oximetry at admission at all (median at baseline 0%), with the highest baseline use being 65% (H13). For the hospitals joining after the strike (i.e. from November 2018), use of pulse oximetry during admission was generally low for 4/5 hospitals (median at baseline 25%) but one hospital already used pulse oximetry on 83% admissions (H16) (Figure 3). Hospitals demonstrated various patterns of adoption: i) low adoption when joining the CIN with a sharp improvement later (e.g. Hospital H2, H12), ii) oscillation between low and high pulse oximetry use (e.g. Hospital H3, H10, H17), iii) gradual improvement that later stabilised (e.g. Hospital H1, H4, H14), and iv) failure of adoption (H7 and H5)(Figure 3). These varying patterns of adoption, especially after December 2018, do not clearly seem

For peer review only

For peer review only - http://bmjopen.bmj.com/site/about/guidelines.xhtml

to be related to study participation. Hospitals participating in both new studies, resulting in presence of two additional non-physician clinician research team members, did demonstrate a consistent improvement in pulse oximetry use at admission (Figure 2 and Figure 3). However, pulse oximetry adoption at admission was also high in hospitals not participating in any study within the CIN (Figure 2 & Figure 3).

Figure 3: Hospital specific rate of pulse oximetry use at admission over time divided into sites joining from Jan 2014 and those recruited from Dec 2018. Vertical red-line indicates national strike disruption. The title is colour-coded to reflect the research study the hospital was recruited into [Blue: None, Orange: RTS,S, Red: SEARCH, Green: RTS,S+SEARCH]. H7 exited from CIN and is therefore not included in subsequent analysis.

Individual patient data and exploratory modelling

Table 2 provides the characteristics of the children admitted to these hospitals, including the level of missingness in each variable. From the study population, 32% had at least one missing variable, with the median number of variables missing per patient of 2 of 30 (IQR: 0 - 3). None of the individual variables was missing more than 30% of values. In this paediatric admission population, the prevalence of chest indrawing, tachypnoea, and grunting is 22.4%, 33% and 10.3% respectively and inability to drink (or breastfeed), a danger sign requiring urgent attention, was present in 72.7% of the admissions; 82.9% of all the admissions had at least one of these signs. The median length of stay was 3 days (IQR: 2 - 6), with a median of one additional comorbid condition at admission (IQR: 0 – 1). Pneumonia and Malaria were the leading, often comorbid, admission diagnoses (Supplementary Figure 1).

Of the 132737 cases, 48.8% (n= 64722) had a pulse oximetry measurement taken at admission. Of the 64722 patients with pulse oximetry measured at admission, 4510 (7%) had hypoxaemia (SpO_2 < 90%). Across the same period, 11428/132737 (8.6%), were documented to have oxygen prescribed at

For peer review only

For peer review only - http://bmjopen.bmj.com/site/about/guidelines.xhtml

admission. Only 1484/11428 (12.8%) were confirmed to be hypoxaemic using pulse oximetry, while 6633/11428 (58%) had no pulse oximetry measurement and 3311/11428 (28.9%) were documented to have a pulse oximetry value of 90% or higher at admission. Thus, 87% oxygen use was not pulse oximetry guided ($SpO_2 \geq 90\%$ or unknown) and more than twice as many children prescribed oxygen had values ≥90% (3311 compared with 1484)(Figure 4).

Figure 4: Oxygen prescription and use of pulse oximetry at admission

Table 2: Descriptive summary statistics for the variables of the patient population included in the study

Indicator	Levels	Number, n (%)	Missing data, n (%)
Hospital level factors			
Malaria endemicity zone	High	53177 (40.1%)	
	Low	79560 (59.9%)	
Patient-level factors			
Age*	< 1 month	3321 (2.5%)	1221 (0.9%)
	1-11 months	38235 (28.8%)	
	12-59 months	64628 (48.7%)	
	>= 5 years	25332 (19.1%)	
Female	Present	58588 (44.1%)	1199 (0.9%)
Case is readmission	Present	13557 (10.2%)	37002 (27.9%)
Case is referral to hospital	Present	19525 (14.7%)	39879 (30%)
Presence of comorbidity	Present	82918 (62.5%)	725 (0.5%)
AVPU** = A	Present	113378 (85.4%)	10664 (8%)
Slow capillary refill	Present	2231 (1.7%)	21902 (16.5%)
Pallor	None	95612 (72%)	10799 (8.1%)
	Mild/Moderate	15596 (11.7%)	
	Severe	10730 (8.1%)	
Fever	Present	105268 (79.3%)	6952 (5.2%)
Cough	Present	64819 (48.8%)	9237 (7%)
Crackles	Present	22835 (17.2%)	11695 (8.8%)
Central cyanosis	Present	974 (0.7%)	9758 (7.4%)
Acidotic breathing	Present	3098 (2.3%)	12740 (9.6%)
Difficulty breathing	Present	38825 (29.2%)	10454 (7.9%)
Grunting	Present	13701 (10.3%)	12255 (9.2%)
Tachypnoea***	Present	43832 (33%)	31136 (23.5%)
Indrawing	Present	29698 (22.4%)	11419 (8.6%)
Wheezing	Present	7085 (5.3%)	11034 (8.3%)
Stridor	Present	2719 (2%)	14121 (10.6%)
Inability to drink	Present	96455 (72.7%)	14846 (11.2%)
Weight-for-Age Z-score	> -2SD (Normal)	74035 (55.8%)	33486 (25.2%)
	-2SD to -3SD (Low)	12377 (9.3%)	

For peer review only

For peer review only - http://bmjopen.bmj.com/site/about/guidelines.xhtml

Table 2: Descriptive summary statistics for the variables of the patient population included in the study			
**Indicator**	**Levels**	**Number, n (%)**	**Missing data, n (%)**
	< -3SD (Very low)	12839 (9.7%)	
Pulse oximetry done at admission****	Present	64722 (48.8%)	
Oxygen therapy prescribed at admission	Present	11428 (8.6%)	4275 (3.2%)
Outcome at discharge	Alive	123845 (93.3%)	499 (0.4%)
	Died	8393 (6.3%)	

Note:
* < 1 month represents neonates admitted to paediatric ward; no data from the new-born units were included.
** AVPU scale is an acronym for "Alert, Verbal, Pain, Unresponsive" and is a measure of a patient's level of consciousness ordered by severity from left to right, with "A" being the least severe.
*** Symptom is derived and calculated as present where either (1) respiratory rate > 59 and age < 1 month, (2) respiratory rate > 49 and age is 1-11 months, or (3) respiratory rate is > 39 and age is 12 months or older.
**** If missing, it was assumed that it was not done. 5525 admission cases reported having done it but did not have the SpO_2 value on the paper record.

Stridor, wheezing, acidotic breathing and cyanosis have relatively low recorded prevalence of 2%, 5.3%, 2.3% and 0.7% respectively and were not included in our previous models [10]. Whether a child is a readmission or referral from another health facility are also newly added data with higher prevalence at 10.2% and 14.7% of the patient population respectively [10] (Table 2).

Summary of the growth curve model results

Table 3 provides the results of the growth curve models, where adoption rates between hospitals were adjusted for malaria endemicity, participation in research studies, participants' demographic characteristics and clinical signs and symptoms at admission. These results are from analysis using 35 imputed datasets (32% of the cohort had at least one missing variable).

Table 3: Growth curve model of pulse oximetry adoption and orders for oxygen use at admission						
	**Pulse Oximetry Done?**			**Oxygen Prescribed?**		
**Predictors**	**OR**	**95% CI**	**p-value**	**OR**	**95% CI**	**p-value**
(Intercept)	0.03	0.01 - 0.17	<0.001	0.01	0.01 - 0.02	<0.001
Hospital factors						
Malaria Endemicity Zone: High (Ref: Low)	1.62	0.1 - 25.04	0.729	1.55	0.9 - 2.67	0.112
Time (Months in CIN)	1.11	1.05 - 1.18	<0.001	1.02	1 - 1.03	0.045

For peer review only - http://bmjopen.bmj.com/site/about/guidelines.xhtml

Table 3: Growth curve model of pulse oximetry adoption and orders for oxygen use at admission						
	Pulse Oximetry Done?			Oxygen Prescribed?		
Patient-level factors						
Ref: Patient in any study [No] ǂ						
Patient in any study [Pre-study period]ǂ ǂ	2.79	2.58 - 3.02	<0.001	1.02	0.9 - 1.15	0.800
Patient in any study [Yes]	1.46	1.35 - 1.58	<0.001	1.02	0.91 - 1.15	0.715
Referral: Yes (Ref: No)	1.05	1.01 - 1.09	0.025	1.19	1.12 - 1.26	<0.001
Readmission: Yes (Ref: No)	1.01	0.97 - 1.05	0.732	1.07	1 - 1.15	0.052
Is a weekend admission: Yes (Ref: No)	0.98	0.95 - 1.01	0.144	1.07	1.02 - 1.13	0.009
PAR used: Yes (Ref: No)	1.99	1.79 - 2.21	<0.001	1.23	1.02 - 1.48	0.031
PAR used * Time (Months in CIN)	1.01	1 - 1.01	<0.001	1	1 - 1.01	0.562
Ref: Age [> 59 months]						
Age [< 1 month]	0.46	0.41 - 0.51	<0.001	1.96	1.62 - 2.35	<0.001
Age [1-11 months]	1.08	1.03 - 1.13	<0.001	1.43	1.32 - 1.54	<0.001
Age [12-59 months]	1.09	1.05 - 1.13	<0.001	1.08	1 - 1.16	0.054
Female: Yes (Ref: No)	1	0.97 - 1.02	0.723	1.02	0.97 - 1.06	0.424
Fever: Yes (Ref: No)	1.18	1.14 - 1.23	<0.001	0.78	0.73 - 0.83	<0.001
Cough: Yes (Ref: No)	1.04	1.01 - 1.08	0.008	1.36	1.29 - 1.44	<0.001
Difficulty Breathing: Yes (Ref: No)	1.06	1.02 - 1.1	0.002	2.79	2.64 - 2.94	<0.001
Stridor: Yes (Ref: No)	1.05	0.95 - 1.16	0.325	1.14	1.02 - 1.27	0.017
Cyanosis: Yes (Ref: No)	0.77	0.65 - 0.92	0.004	1.5	1.27 - 1.77	<0.001
Indrawing: Yes (Ref: No)	1.25	1.2 - 1.31	<0.001	2.36	2.23 - 2.49	<0.001
Grunting: Yes (Ref: No)	0.88	0.84 - 0.93	<0.001	1.9	1.8 - 2	<0.001
Acidotic Breathing: Yes (Ref: No)	0.89	0.8 - 0.98	0.018	1.34	1.22 - 1.47	<0.001
Wheezing: Yes (Ref: No)	1.05	0.98 - 1.12	0.167	1.13	1.06 - 1.21	0.001
Crackles: Yes (Ref: No)	1.06	1.01 - 1.11	0.010	1.25	1.19 - 1.32	<0.001
Tachypnoea: Yes (Ref: No)	0.96	0.93 - 0.99	0.022	1.59	1.51 - 1.68	<0.001
Alert (AVPU < A): No (Ref: Yes)	0.96	0.9 - 1.03	0.238	1.95	1.81 - 2.1	<0.001
Inability to drink: Yes (Ref: No)	1.04	1 - 1.08	0.043	0.8	0.76 - 0.85	<0.001
Ref: Pallor [None]						
Pallor [Mild/Moderate]	0.93	0.89 - 0.97	0.002	1.1	1.02 - 1.17	0.008
Pallor [Severe]	0.94	0.89 - 0.99	0.024	1.3	1.19 - 1.42	<0.001
Ref: WAZ [Normal]						
WAZ [Low]	0.97	0.92 - 1.01	0.135	0.98	0.91 - 1.05	0.551

For peer review only

Table 3: Growth curve model of pulse oximetry adoption and orders for oxygen use at admission						
	Pulse Oximetry Done?			Oxygen Prescribed?		
WAZ [Very Low]	0.94	0.9 - 0.99	0.020	0.93	0.87 - 1	0.059
Slow Capillary Refill: Yes (Ref: No)	0.88	0.78 - 1	0.051	0.97	0.83 - 1.13	0.693
Ref: Hypoxaemia [Unknown]						
Hypoxaemia [No]				0.59	0.56 - 0.62	<0.001
Hypoxaemia [Yes]				1.92	1.77 - 2.08	<0.001

Note:

‡"*Patient is in any study*" point estimates and confidence intervals for the *"pulse oximetry done?"* outcome do not overlap with ones from complete case analysis (Supplementary Table 4). From the complete case sensitivity analysis, there is a material difference in the estimated association of involvement in any research study variable with the pulse oximetry use at admission outcome (Table 3, Supplementary Table 4); While the direction of association is the same (positive), the confidence intervals do not overlap. With the age variable, the direction of the association is inverted between the complete case and multiple imputation analyses, and children aged < 1 month admitted to the paediatric ward are completely missing in the complete case analysis. These material difference might be attributable to the fact that complete case analysis and our multiple imputation approach make different assumptions of missingness mechanism in the data (i.e. Missing Completely At Random (MCAR) mechanism -which is potentially more biased, and a MAR mechanism respectively).

‡‡"*Patient is in any study[Pre-study period]*" reflects the time leading up to any research study starting when no study was being conducted i.e. February 2014 – November 2018.

Associations of the hospital and clinical features with the outcomes from Table 3 are time-invariant (i.e. independent of the effect of time in months in CIN), except for the interaction term of PAR use with time. From Supplementary Table 4 and Figure 3, the models' high Intraclass Correlation Coefficient (ICC) is indicative that hospital factors play a bigger role in the adoption of pulse oximetry over time compared to individual patient-level factors. The contribution of hospital level factors is less pronounced for oxygen prescription at admission. The relatively high ICC is not unexpected in longitudinal models with measurements clustered by hospital and given the varying patterns of adoption seen in Figure 3.

For peer review only

For peer review only - http://bmjopen.bmj.com/site/about/guidelines.xhtml

Association with the use of pulse oximetry at admission

Over the entire study period and across all hospitals pulse oximetry use at admission improved as time participating in the CIN increased (month-to-month improvement OR: 1.11, 95% CI: 1.05 – 1.18). This was likely driven by noticeable improvement in 10 hospitals and in 5 of these specifically there were substantive positive changes in adoption of pulse oximetry on a month-to-month basis; (Supplementary Figure 2). Those improving most tended to have very low use of oximetry at the time they joined the CIN (Supplementary Figure 2). This improvement was seen in tandem with the improvement in PAR use which might be expected to encourage better assessment and potentially, therefore, aid clinical decision making.

From Table 3, patients admitted with signs of fever, cough, difficulty breathing, chest indrawing, crackles, and inability to drink had significantly higher odds of having pulse oximetry measurement taken at admission over time than those without these signs. With a larger dataset, the inclusion of new variables and adjusting for the time in the CIN, our findings conflict with previous findings on the significance of tachypnoea, altered consciousness (AVPU < A), and inability to drink as signs associated with the use of pulse oximetry at admission with a reversal in association (e.g. Tachypnoea, Inability to drink) or no statistically appreciable association (e.g. AVPU <A)[10]. Grunting and acidotic breathing symptoms, likely to be associated with malaria and anaemia (Supplementary Figure 1), were statistically, not significantly associated with pulse oximetry use at admission (Table 3). Cyanosis, grunting, and acidotic breathing –which were previously not reported [10]- were significantly associated with the odds of pulse oximetry done at admission being lower than one. Our findings also highlight that referral into the hospital is positively associated with the use of pulse oximeters at admission (OR: 1.05, 95% CI: 1.01 - 1.09)(Table 3) and that patients admitted to hospitals that were part of an active research study were 49% more likely to have a pulse oximetry measurement taken at admission than in hospitals not participating in any study (Table 3). Additional analyses suggest the more clinical signs a child has of respiratory distress the more likely they were to be hypoxemic (Supplementary Figure 3).

For peer review only - http://bmjopen.bmj.com/site/about/guidelines.xhtml

Associations with oxygen prescription at admission

Admission with any sign of respiratory distress where the pulse oximetry measure is unknown is associated with oxygen prescription at admission (Table 3). The specific signs that appear to prompt oxygen prescription, in reducing magnitude of association, include difficulty breathing, indrawing, AVPU <A, grunting, and tachypnoea. Admissions with altered consciousness (AVPU < A) had almost twice the odds of oxygen prescription at admission, independent of respiratory signs (Table 3). Interestingly, the direction of association with oxygen prescription is inverse to that for use of pulse oximetry for: age of the child, signs of cyanosis, grunting, acidotic breathing, AVPU <A, pallor, tachypnoea, fever and inability to drink.

With the inclusion of new variables and adjusting for the time in CIN, our findings affirm previous analyses but show the magnitude of association of cough and difficulty breathing with oxygen prescription at admission to be higher than previously reported [10]. Acidotic breathing, stridor and wheezing, clinical signs omitted from the previous study, were shown to be statistically significant and positively associated with the odds of being prescribed oxygen at admission. Grunting and acidotic breathing, likely to be associated with Malaria and Anaemia diagnoses, were not statistically associated with having pulse oximetry measurement taken at admission but were strongly associated with the prescription of oxygen at admission (Table 3).

Our findings also highlight that being a patient referred into the hospital (OR: 1.19, 95% CI: 1.12 - 1.26) or admitted during the weekend (OR: 1.07, 95% CI: 1.02 - 1.13) is positively associated with oxygen prescription at admission (Table 3). Oxygen prescription at admission increased with time as part of CIN, having a statistically significant but clinically small month-to-month change (OR: 1.02, 95% CI: 1.00 – 1.03). The improvement in the PAR use (OR: 1.23, 95% CI: 1.02 - 1.48) also appears to be aiding clinical decision making in the prescription of oxygen based on the recording of features of respiratory distress.

For peer review only

For peer review only - http://bmjopen.bmj.com/site/about/guidelines.xhtml

Discussion

Over a period of 7 years, with prolonged strikes precluding data collection in 2017, use of pulse oximetry in a population of admissions where 86% have at least one feature indicating risk of hypoxemia increased slowly with each month of participation in CIN. Most hospitals at the start of their participation in CIN used pulse oximetry in 25% or fewer children, increasing to 81.5% in all hospitals by the final quarter of 2020. Amongst the admissions prescribed oxygen in 87% this was not apparently guided by a pulse oximetry value (SpO2) of < 90%, the recommended level for intervention to address hypoxemia in current pneumonia guidelines.

The highly variable patterns of pulse oximetry adoption across hospitals (Figure 3) and high intraclass correlation coefficients in our models point to the major impact of hospital-level factors on the use of pulse oximetry. These have been previously described and include equipment supply, maintenance, local leadership and clinical efforts to train and promote oximetry use amongst others [10]. CIN provided regular feedback to hospitals including on pulse oximetry use and SpO_2 values throughout a hospital's participation in CIN and engaged subsets of hospitals in research projects that resulted in the placement of one or two additional non-physician clinicians (i.e. clinical officers, nurses) in some hospitals. In the absence of data from other settings it is hard to determine if the increase in pulse oximetry use is part of a wider secular trend (a phenomenon referred to as the "rising tide" [34]) or associated with CIN participation. Whatever the reasons, it is instructive that adoption of pulse oximetry remains incomplete after 7 years even amongst inpatient populations with severe illness in moderately sized hospitals. This slow pattern of adoption for an established technology that is widely believed to be beneficial has been observed in high-income settings. It suggests we may only realise the proposed benefits of many technologies including diagnostics much more slowly than is often claimed unless much greater attention is paid to strengthening health service delivery at scale [35, 36].

For peer review only

Overall, approximately 9% of patients were prescribed oxygen at admission, with a slight increase in patients with oxygen prescribed with each month in the CIN. Interestingly, in our models the intraclass correlation coefficients exploring oxygen use did not suggest strong hospital-level effects, placing more emphasis on patient-level factors. The number of patients with oxygen prescribed who had no SpO_2 recorded is large: it may have been that in some patients this was because of a diagnosis of shock or severe asthma where the normal SpO_2 minimum threshold is 94%, however these diagnoses are relatively uncommon in Kenyan hospitals [1, 37]. In fact, more than twice as many children receiving oxygen had pulse oximetry values 90% or more than values < 90% (Figure 4). From WHO guidelines, any child with SpO2 < 90% should receive oxygen [1].

For peer review only

We attempt to integrate our new findings of patient-level factors associated with the use of pulse oximetry and prescription of oxygen at admission with earlier findings that addressed mainly hospital-level factors in Figure 5. Lower chest-wall indrawing is strongly and positively associated with pulse oximetry being done at admission and prescription of oxygen when pulse oximetry is not done. We have previously shown an association between indrawing and mortality for pneumonia admissions and others have recently indicated indrawing may be a useful referral criterion from the community or primary care to a hospital in the absence of pulse oximetry [5, 38]. However, randomised trials, and WHO guidance, suggest pneumonia associated with indrawing can be safely treated as an outpatient [2, 37]. Our data does not directly inform the debate on the value of indrawing as screening sign of illness severity but does perhaps suggest it remains an important factor in Kenyan clinicians' decision-making.

Other signs prompting pulse oximeter use are fever, cough, difficulty breathing, crackles and inability to drink (Table 3, Figure 5). All respiratory signs are associated with oxygen prescription (Table 3, Supplementary Table 5, Figure 5) and a pulse oximetry value <90% was associated with twice the odds of oxygen prescription compared with those whose oxygen saturation was unknown. Where information from pulse oximetry was not available cyanosis, grunting, acidotic breathing, AVPU <A,

For peer review only - http://bmjopen.bmj.com/site/about/guidelines.xhtml

pallor, and tachypnoea appeared to prompt oxygen use (Table 3, Figure 5). Several of these signs were not associated with the use of pulse oximetry at admission, perhaps because healthcare providers are taught and then view these signs as indicative of very severe illness (as reported in the literature) requiring intervention with oxygen irrespective of oxygen saturation [1, 37, 38]. Interestingly, over time, the use of the standardised paediatric admission record (PAR) was associated with twice the odds of a patient having SpO_2 measurement and 23% increase in oxygen therapy prescribed at admission, where indicated (Table 3). It is possible this effect reflects its role as a prompt to conduct a complete assessment and promote reflection on the use of core interventions recommended in guidelines.

It has been well described in many settings that the logical world represented in clinical guidelines is often not replicated in the more complex world of everyday practice [39-41]. While there remain debates in the scientific world around which oxygen saturation threshold for which condition in which setting should guide the use of oxygen [5, 42, 43], our data remind us that clinicians are often influenced by many other factors in making such decisions [44]. Clinical decision making is not straightforward, and it is difficult to capture from routinely collected observational data. Reducing the decision of whether to prescribe or withhold oxygen to a pulse oximetry reading might inadequately reflect the complexities that a clinician encounters at the patient bedside. This has important implications as the presence or prevalence of hypoxemia, however defined, may be used as proxies for rational use or need for oxygen respectively. Whether clinicians should be strongly advised to adhere to pulse oximetry defined hypoxemia thresholds to determine oxygen use remains unclear as research has rarely addressed the effects this would have on resource use or patient outcomes [2].

For peer review only - http://bmjopen.bmj.com/site/about/guidelines.xhtml

Figure 5: Adapted Integrative Model of Behavioural Prediction (Enoch et al. [10]). Dashes indicate components of the model that are modified or added to reflect the findings from our new analyses. HCW: healthcare worker.

Strengths and limitations

The data we present are a rare example of long-term tracking of hospital practice in a routine LMIC setting covering common childhood illnesses (Supplementary Table 6). The longitudinal nature of our observations offers important insights but also poses challenges. Major changes may occur in the health sector over long periods, as exemplified by the prolonged strikes in Kenya. Many factors ranging from national and local procurement or equipment donations, to changes in local leadership and hospital's participation in specific studies might influence the adoption of pulse oximetry and use of oxygen we describe. Furthermore, we have no contemporaneous data from Kenyan settings that do not belong to the CIN for comparison and most other studies exploring the use of pulse oximetry are essentially cross-sectional. We do, however, feel our data are at least a useful illustration of the slow and sometimes erratic process of pulse oximetry adoption in LMIC hospitals that has broader implications for thinking on technology adoption.

Another limitation of this study is the absence of new qualitative data especially exploring why oxygen use is often not linked to pulse oximetry values. However, we have previously shown the importance of local clinical leadership in these settings [45] and the continuing challenge of promoting guideline adherence in these complex hospital settings where junior clinicians who manage patients rotate frequently and may err on the side of caution to use interventions rather than risk withholding them [46-48].

Further challenges of the large-scale, longitudinal nature of our work are data quality and analytic methods. The CIN has made efforts to capture high-quality data through continuous training of clerks linked to error checking procedures and regular engagement with hospitals that include monitoring and feedback [18, 28, 29]. Nonetheless, imputation procedures that assume a missing at random

For peer review only

For peer review only - http://bmjopen.bmj.com/site/about/guidelines.xhtml

mechanism had to be employed to enable analyses of all eligible case records [32, 49]. We also had to employ a complex modelling framework to explore patient-level associations with our outcomes. These attempted to accommodate and account for between hospital variability, within hospital non-linear trends in the outcomes over time and entry into the CIN at different time points. We tested our assumptions where possible (e.g. that the strike year was not associated with major changes in practice). However, while our large dataset enabled us to identify many credible associations specific findings should be interpreted cautiously.

Conclusions

Over time, there has been a progressive increase in the use of pulse oximeters at admission in the Kenyan hospitals we studied. After seven years, however, use in this high mortality population where almost 9 out of 10 children have least one sign indicating increased risk of hypoxemia is not universal and major differences exist between hospitals in the adoption of this technology. Guidelines suggest pulse oximetry should determine which children receive oxygen. Our data suggest this is often not the case even in late 2020 during the COVID-19 pandemic, and that many organisational and clinical factors influence whether oximetry is used at all or whether recommended hypoxemia definitions are used to guide the use of oxygen. As global efforts gain pace to provide pulse oximeters and oxygen systems our data show it is perhaps naïve to expect they will be adopted and employed in the rational ways imagined in guidelines and policies.

For peer review only

Appendix

CRediT author statement

Timothy Tuti: Conceptualization, Methodology, Software, Data curation, Visualization, Writing- Original draft, Writing - Reviewing and Editing. **Jalemba Aluvaala**: Methodology, Investigation, Writing- Reviewing and Editing. **Samuel Akech**: Methodology, Investigation, Writing- Reviewing and Editing. **Ambrose Agweyu**: Methodology, Investigation, Writing- Reviewing and Editing. **Grace Irimu**: Writing- Reviewing and Editing **Mike English**: Conceptualization, Methodology, Supervision, Investigation, Writing- Reviewing and Editing.

For peer review only - http://bmjopen.bmj.com/site/about/guidelines.xhtml

Ethics approval and consent to participate

This CIN study was approved by the Kenya Medical Research Institute's (KEMRI) Scientific and Ethics Review Unit (#SERU 3459). Additionally, it was approved by the Ministry of Health, with the Medical Superintendents of participant hospitals given consent for participation. Individual consent for access to de-identified patient data was not required.

Consent to publish

This paper is published with the permission of the Director of KEMRI.

For peer review only

Acknowledgements

The Clinical Information Network Author Group: The CIN author group who contributed to the network's development, data collection, data management, implementation of audit and feedback and who reviewed and approved this publication include: 1) Central CIN team members: Lynda Isaaka, George Mbevi, Cynthia Khaenzi, Paul Mwaniki, Mercy Chepkirui, John Wainaina, Livingstone Mumelo, Edith Gicheha, Monica Musa, Naomi Muinga and Muthoni Ogola. 2) National partners: Laura Oyiengo, Caroline Mwangi, Fred Were, and (3) Hospital teams: (**Paediatricians)** Juma Vitalis, Nyumbile Bonface, Roselyne Malangachi, Christine Manyasi, Catherine Mutinda, David Kibiwott Kimutai, Rukia Aden, Caren Emadau, Elizabeth Atieno Jowi, Cecilia Muithya, Charles Nzioki, Supa Tunje, Dr. Penina Musyoka, Wagura Mwangi, Agnes Mithamo, Magdalene Kuria, Esther Njiru, Mwangi Ngina, Penina Mwangi, Rachel Inginia, Melab Musabi, Emma Namulala, Grace Ochieng, Lydia Thuranira, Felicitas Makokha, Josephine Ojigo, Beth Maina, Catherine Mutinda, Mary Waiyego, Bernadette Lusweti, Angeline Ithondeka, Julie Barasa, Meshack Liru, Elizabeth Kibaru, Alice Nkirote Nyaribari, Joyce Akuka, Joyce Wangari;

(Nurses) Amilia Ngoda, Aggrey Nzavaye Emenwa, Dolphine Mochache, Patricia Nafula Wesakania, George Lipesa, Jane Mbungu, Marystella Mutenyo, Joyce Mbogho, Joan Baswetty, Ann Jambi, Josephine Aritho, Beatrice Njambi, Felisters Mucheke, Zainab Kioni, Jeniffer, Lucy Kinyua, Margaret Kethi, Alice Oguda, Salome Nashimiyu Situma, Nancy Gachaja, Loise N. Mwangi, Ruth Mwai, irginia Wangari Muruga, Nancy Mburu, Celestine Muteshi, Abigael Bwire, Salome Okisa Muyale,

For peer review only - http://bmjopen.bmj.com/site/about/guidelines.xhtml

Naomi Situma, Faith Mueni, Hellen Mwaura, Rosemary Mututa, Caroline Lavu, Joyce Oketch, Jane Hore Olum, Orina Nyakina, Faith Njeru, Rebecca Chelimo, Margaret Wanjiku Mwaura, Ann Wambugu, Epharus Njeri Mburu, Linda Awino Tindi, Jane Akumu, Ruth Otieno, Slessor Osok; **(HRIOs)** Seline Kulubi, Susan Wanjala, Pauline Njeru, Rebbecca Mukami Mbogo, John Ollongo, Samuel Soita, Judith Mirenja, Mary Nguri, Margaret Waweru, Mary Akoth Oruko, Jeska Kuya, Caroline Muthuri, Esther Muthiani, Esther Mwangi, Joseph Nganga, Benjamin Tanui, Alfred Wanjau, Judith Onsongo, Peter Muigai, Arnest Namayi, Elizabeth Kosiom, Dorcas Cherop, Faith Marete, Johanness Simiyu, Collince Danga, Arthur Otieno Oyugi, Fredrick Keya Okoth.

For peer review only

Data Availability

Patient-level data used in this report are under the primary jurisdiction of the Ministry of Health in Kenya. The authors had full access to all the quantitative data in the study and take responsibility for the integrity of the data and the accuracy of the data analysis. Researchers can contact the KEMRI Wellcome Trust Research Programme Data Governance Committee (dgc@kemri-wellcome.org) for advice on accessing the data including whether a new ethical review submission and approval from the Ministry of Health in Kenya would be required.

Funding

The work described was supported by multiple funders. These include the Wellcome Trust (#207522) through an award to ME as a Senior Fellowship and a core grant awarded to the KEMRI-Wellcome Trust Research Programme (#092654); by a grant from World Health Organization: An evaluation in Kenya of the cluster-randomized RTS,S/AS01 malaria vaccine implementation pilot through routine health systems in moderate to high malaria transmission settings (WHO-Project requisition number 2018/854999); by a grant to the NEST program from the John D. and Catherine T. MacArthur Foundation, the Bill & Melinda Gates Foundation, ELMA Philanthropies, and The Children's Investment Fund Foundation UK under agreements to William Marsh Rice University with a sub-agreement to University of Oxford Centre for Tropical Medicine and Global Health; and an award to AA (MR/R006083/1) from the Foreign, Commonwealth and Development Office (FCDO), the MRC, the

National Institute for Health Research (NIHR) and Wellcome Trust Joint Global Health Trials Scheme.

For the purpose of Open Access, the author has applied a CC-BY public copyright licence to any author accepted manuscript version arising from this submission.

The funders had no role in the preparation of this report or the decision to submit for publication.

Competing interests

We have read the journal's policy and the authors of this manuscript have no competing interests to declare

Abbreviations:

CI, confidence interval; CIN, Clinical Information Network; FCS, fully conditional specification; HCW, healthcare worker; HRIO, Hospital Records Information Officer; ICC, Intra-cluster Correlation Coefficient; KEMRI, Kenya Medical Research Institute; LMIC, low- and middle-income country; MAR, Missing At Random; MCAR, Missing Completely At Random; MICE, Multivariate Imputation by Chained Equations; MoH, Ministry of Health; OR, odds ratio; PAR, Paediatric Admission Record; SERU, KEMRI's Scientific and Ethics Review Unit; WHO, World Health Organization

References

1. World Health Organization, *Manual on use of oxygen therapy in children.* World Health Organization (WHO), 2016.
2. Enoch, A.J., M. English, and S. Shepperd, *Does pulse oximeter use impact health outcomes? A systematic review.* Archives of disease in childhood, 2016. **101**(8): p. 694-700.
3. Jouffroy, R., D. Jost, and B. Prunet, *Prehospital pulse oximetry: a red flag for early detection of silent hypoxemia in COVID-19 patients.* Critical Care, 2020. **24**(1): p. 1-2.
4. Starr, N., et al., *Pulse oximetry in low-resource settings during the COVID-19 pandemic.* The Lancet Global Health, 2020. **8**(9): p. e1121-e1122.
5. Colbourn, T., et al., *Predictive value of pulse oximetry for mortality in infants and children presenting to primary care with clinical pneumonia in rural Malawi: A data linkage study.* PLoS medicine, 2020. **17**(10): p. e1003300.
6. Adebola, O., O. Babatunde, and O. Bose, *Hypoxemia predicts death from severe falciparum malaria among children under 5 years of age in Nigeria: the need for*

For peer review only

pulse oximetry in case management. African health sciences, 2014. **14**(2): p. 397-407.

7. Lozano, J., *Epidemiology of hypoxaemia in children with acute lower respiratory infection [oxygen therapy in children].* The international journal of tuberculosis and lung disease, 2001. **5**(6): p. 496-504.
8. Liu, L., et al., *Global, regional, and national causes of under-5 mortality in 2000–15: an updated systematic analysis with implications for the Sustainable Development Goals.* The Lancet, 2016. **388**(10063): p. 3027-3035.
9. Gray, A.Z., et al., *Improved oxygen systems in district hospitals in Lao PDR: a prospective field trial of the impact on outcomes for childhood pneumonia and equipment sustainability.* BMJ paediatrics open, 2017. **1**(1).
10. Enoch, A.J., et al., *Variability in the use of pulse oximeters with children in Kenyan hospitals: A mixed-methods analysis.* PLoS medicine, 2019. **16**(12): p. e1002987.
11. Graham, H., et al., *Providing oxygen to children in hospitals: a realist review.* Bulletin of the World Health Organization, 2017. **95**(4): p. 288.
12. Irimu, G., et al., *Tackling health professionals' strikes: an essential part of health system strengthening in Kenya.* BMJ global health, 2018. **3**(6).
13. García-Romero, A., Á. Escribano, and J.A. Tribó, *The impact of health research on length of stay in Spanish public hospitals.* Research Policy, 2017. **46**(3): p. 591-604.
14. Lichten, C.A., et al., *Does a biomedical research centre affect patient care in local hospitals?* Health research policy and systems, 2017. **15**(1): p. 2.
15. Gathara, D., et al., *Variation in and risk factors for paediatric inpatient all-cause mortality in a low income setting: data from an emerging clinical information network.* BMC pediatrics, 2017. **17**(1): p. 99.
16. Von Elm, E., et al., *The Strengthening the Reporting of Observational Studies in Epidemiology (STROBE) statement: guidelines for reporting observational studies.* Annals of internal medicine, 2007. **147**(8): p. 573-577.
17. English, M., et al., *Building learning health systems to accelerate research and improve outcomes of clinical care in low-and middle-income countries.* PLoS medicine, 2016. **13**(4): p. e1001991.
18. Irimu, G., et al., *Approaching quality improvement at scale: a learning health system approach in Kenya.* Archives of disease in childhood, 2018. **103**(11): p. 1013-1019.
19. English, M., et al., *Employing learning health system principles to advance research on severe neonatal and paediatric illness in Kenya.* BMJ Global Health, 2021. **6**(3): p. e005300.

For peer review only

For peer review only - http://bmjopen.bmj.com/site/about/guidelines.xhtml

20. English, M., et al., *Programme theory and linked intervention strategy for large-scale change to improve hospital care in a low and middle-income country-A Study Pre-Protocol.* Wellcome Open Research, 2020. **5**.

21. Ayieko, P., et al., *Characteristics of admissions and variations in the use of basic investigations, treatments and outcomes in Kenyan hospitals within a new Clinical Information Network.* Archives of disease in childhood, 2016. **101**(3): p. 223-229.

22. Ministry of Health, *Kenya Service Availability and Readiness Assessment Mapping (SARAM)*. 2014, Government of Kenya Nairobi, Kenya.

23. Gachau, S., et al., *Does audit and feedback improve the adoption of recommended practices? Evidence from a longitudinal observational study of an emerging clinical network in Kenya.* BMJ global health, 2017. **2**(4): p. e000468.

24. Mwakyusa, S., et al., *Implementation of a structured paediatric admission record for district hospitals in Kenya–results of a pilot study.* BMC international health and human rights, 2006. **6**(1): p. 9.

25. English, M., et al., *Implementing locally appropriate guidelines and training to improve care of serious illness in Kenyan hospitals: a story of scaling-up (and down and left and right).* Archives of disease in childhood, 2011. **96**(3): p. 285-290.

26. Ministry of Health. *Basic Paediatric Protocols*. 2016 [cited 2020 11th Nov]; 4th Edition:[Available from: http://guidelines.health.go.ke/#/category/27/266/meta.

27. Health Services Unit KEMRI–Wellcome Trust Research Programme. *Paediatric Tools & Hospital forms 2016*. 2016 [cited 2020 21st August]; Available from: http://www.kenyapaediatric.org/idoc/index.php/en/etat/instructors/paeditric-tools-a-hospital-forms.html.

28. Tuti, T., et al., *Innovating to enhance clinical data management using non-commercial and open source solutions across a multi-center network supporting inpatient pediatric care and research in Kenya.* Journal of the American Medical Informatics Association, 2016. **23**(1): p. 184-192.

29. Tuti, T., et al., *Improving documentation of clinical care within a clinical information network: an essential initial step in efforts to understand and improve care in Kenyan hospitals.* BMJ global health, 2016. **1**(1): p. e000028-e000028.

30. Curran, P.J., K. Obeidat, and D. Losardo, *Twelve frequently asked questions about growth curve modeling.* Journal of cognition and development, 2010. **11**(2): p. 121-136.

31. Kristoffer, M. *Using R and lme/lmer to fit different two- and three-level longitudinal models*. 2015 [cited 2020 30th September]; Available from: https://rpsychologist.com/r-guide-longitudinal-lme-lmer.

For peer review only - http://bmjopen.bmj.com/site/about/guidelines.xhtml

32. Buuren, S.v. and K. Groothuis-Oudshoorn, *mice: Multivariate imputation by chained equations in R.* Journal of statistical software, 2010: p. 1-68.

33. Gachau, S., et al., *Handling missing data in modelling quality of clinician-prescribed routine care: Sensitivity analysis of departure from missing at random assumption.* Statistical Methods in Medical Research, 2020: p. 0962280220918279.

34. Chen, Y.-F., et al., *Secular trends and evaluation of complex interventions: the rising tide phenomenon.* BMJ quality & safety, 2016. **25**(5): p. 303-310.

35. Oliwa, J.N., et al., *Diagnostic practices and estimated burden of tuberculosis among children admitted to 13 government hospitals in Kenya: an analysis of two years' routine clinical data.* PloS one, 2019. **14**(9): p. e0221145.

36. Graham, H.R., et al., *Adoption of paediatric and neonatal pulse oximetry by 12 hospitals in Nigeria: a mixed-methods realist evaluation.* BMJ global health, 2018. **3**(3): p. e000812.

37. World Health Organization, *Pocket book of hospital care for children: guidelines for the management of common childhood illnesses*. 2013: World Health Organization.

38. Agweyu, A., et al., *Appropriateness of clinical severity classification of new WHO childhood pneumonia guidance: a multi-hospital, retrospective, cohort study.* The Lancet Global health, 2018. **6**(1): p. e74-e83.

39. Gabbay, J. and A. le May, *Mindlines: making sense of evidence in practice*. 2016, British Journal of General Practice.

40. Murray, E., et al., *Normalisation process theory: a framework for developing, evaluating and implementing complex interventions.* BMC medicine, 2010. **8**(1): p. 1-11.

41. Thompson, D.S., et al., *Scoping review of complexity theory in health services research.* BMC health services research, 2016. **16**(1): p. 1-16.

42. Serpa Neto, A., et al., *Pragmatic Recommendations for the Management of Acute Respiratory Failure and Mechanical Ventilation in Patients with COVID-19 in Low-and Middle-Income Countries.* The American Journal of Tropical Medicine and Hygiene, 2021: p. tpmd200796.

43. Maitland, K., et al., *Children's Oxygen Administration Strategies Trial (COAST): A randomised controlled trial of high flow versus oxygen versus control in African children with severe pneumonia.* Wellcome Open Research, 2017. **2**.

44. WHO;, lifebox;, and ALIMA;. *Using pulse oximetry for decision support for COVID-19.* 2020 11th April 2020 [cited 2020 30th October]; Available from: https://www.lifebox.org/wp-content/uploads/2020/04/Pulse-Oximetry-for-COVID-19-Decision-Tool-Lifebox-FINAL1.pdf.

For peer review only

45. Nzinga, J., G. McGivern, and M. English, *Examining clinical leadership in Kenyan public hospitals through the distributed leadership lens.* Health policy and planning, 2018. **33**(suppl_2): p. ii27-ii34.

46. Maina, M., et al., *Antibiotic use in Kenyan public hospitals: Prevalence, appropriateness and link to guideline availability.* International Journal of Infectious Diseases, 2020. **99**: p. 10-18.

47. McKnight, J., et al., *Evaluating hospital performance in antibiotic stewardship to guide action at national and local levels in a lower-middle income setting.* Global Health Action, 2019. **12**(sup1): p. 1761657.

48. Ogero, M., et al., *Examining which clinicians provide admission hospital care in a high mortality setting and their adherence to guidelines: an observational study in 13 hospitals.* Archives of disease in childhood, 2020. **105**(7): p. 648-654.

49. Sperrin, M. and G.P. Martin, *Multiple imputation with missing indicators as proxies for unmeasured variables: simulation study.* BMC Medical Research Methodology, 2020. **20**(1): p. 1-11.

For peer review only

For peer review only - http://bmjopen.bmj.com/site/about/guidelines.xhtml

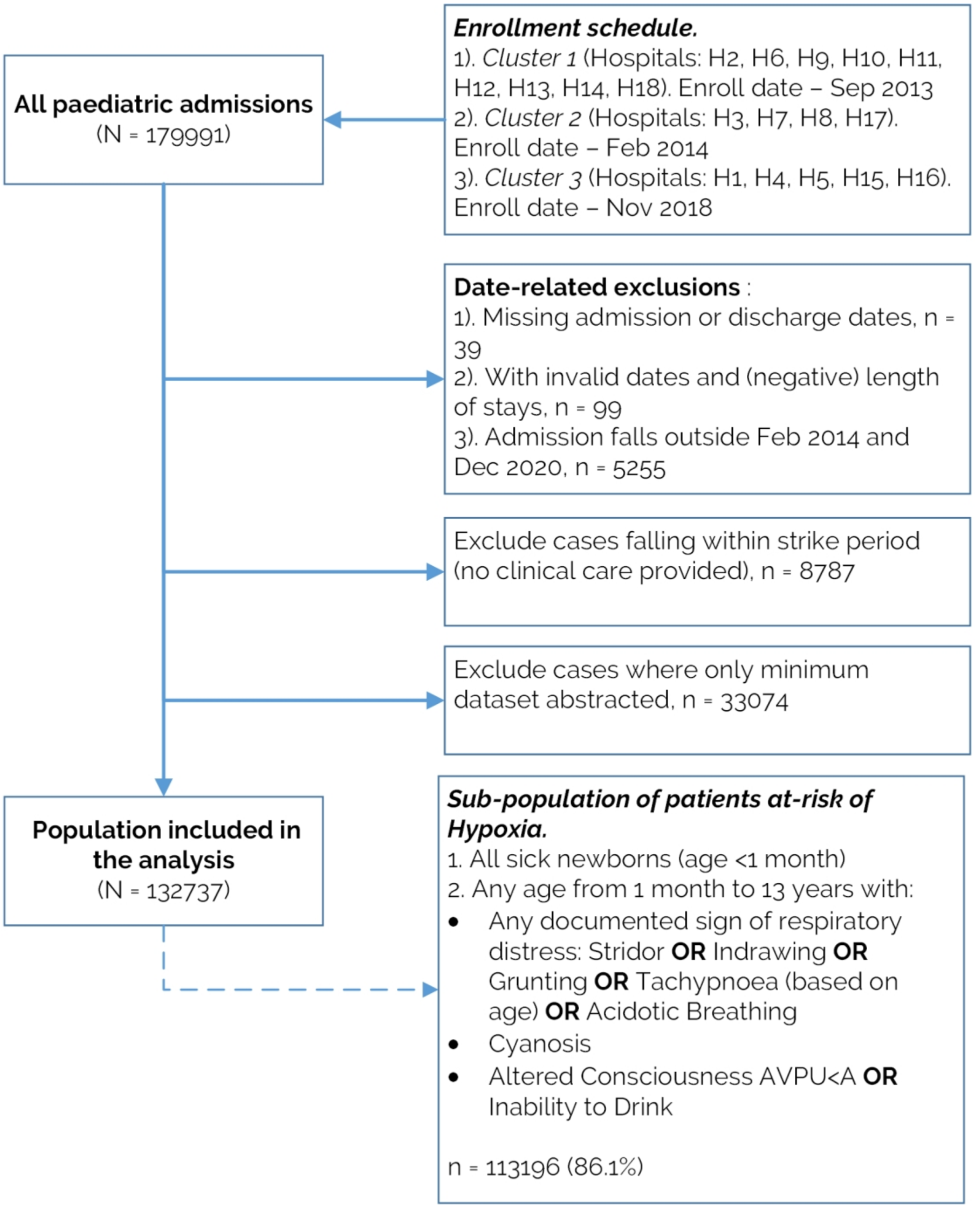

Flow chart of the inclusion criteria

146x180mm (300 x 300 DPI)

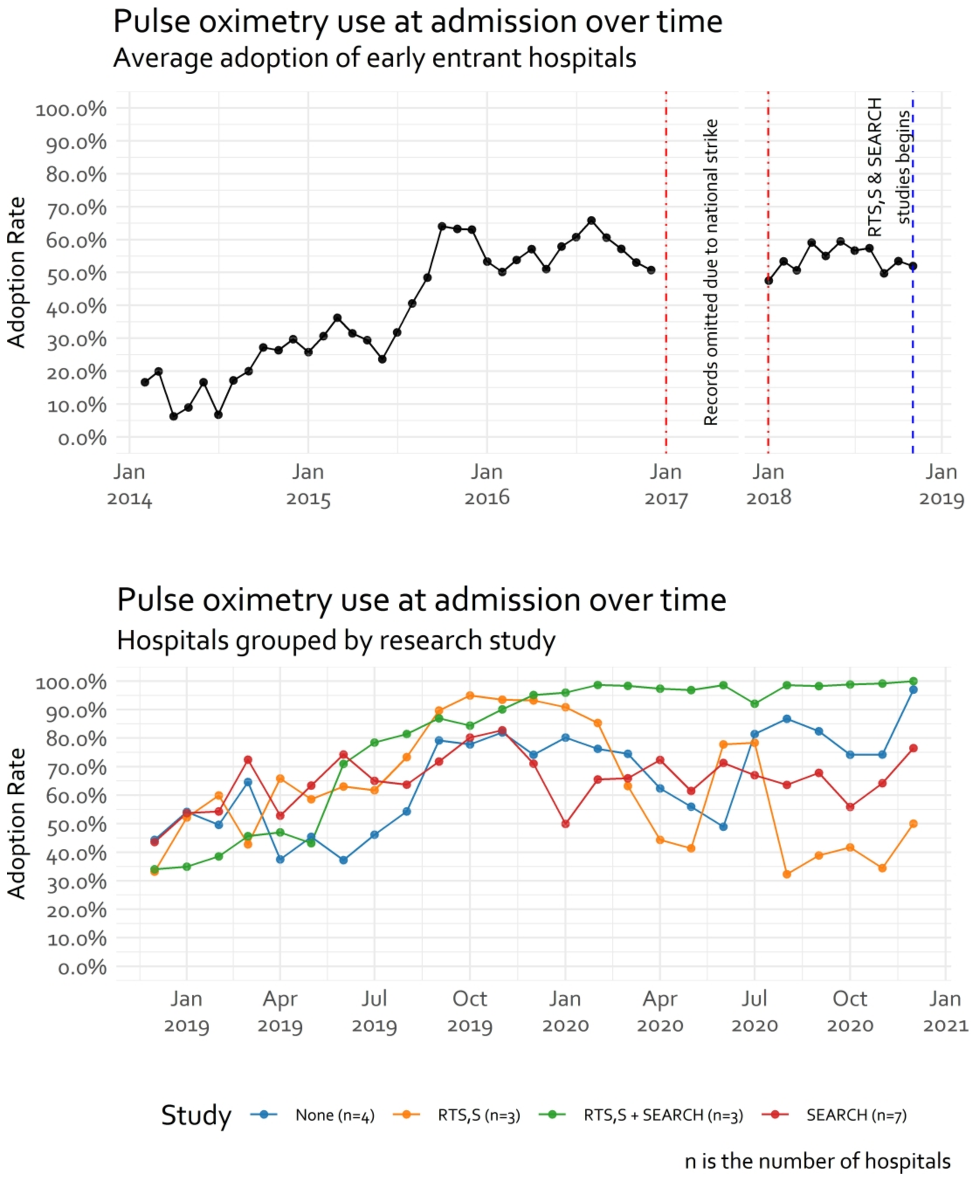

Average pulse oximetry use in the CIN hospitals over time. The number of hospitals in each study covers both presently involved and those joining the CIN later: There are 13 hospitals in the period between Feb 2014 – November 2018, with the lower panel representing transition to 18 hospitals by adding 5 hospitals and research studies beginning. The 5 new hospitals' distribution among the studies in the following way: RTS,S: 2/5; RTS,S + SEARCH: 1/5; SEARCH: 1/5; None: 1/5.

179x209mm (600 x 600 DPI)

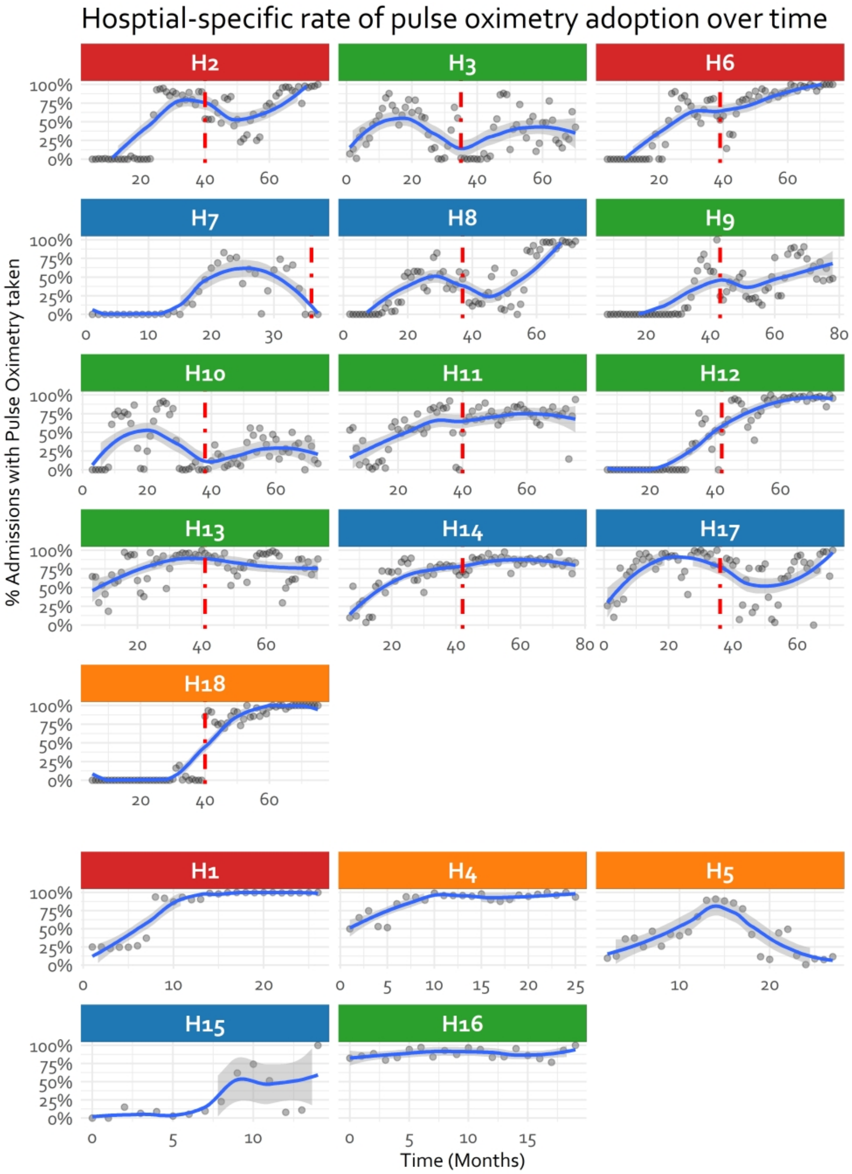

Hospital specific rate of pulse oximetry use at admission over time divided into sites joining from Jan 2014 and those recruited from Dec 2018. Vertical red-line indicates national strike disruption. The title is colour-coded to reflect the research study the hospital was recruited into [Blue: None, Orange: RTS,S, Red: SEARCH, Green: RTS,S+SEARCH]. H7 exited from CIN and is therefore not included in subsequent analysis.

179x246mm (600 x 600 DPI)

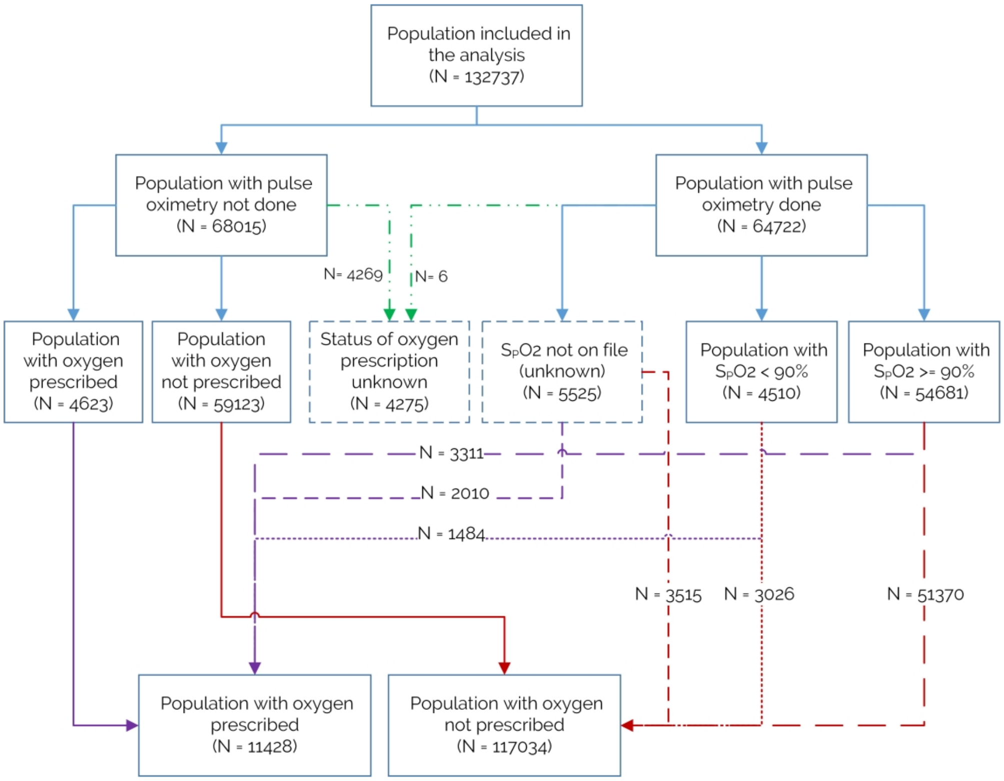

Oxygen prescription and use of pulse oximetry at admission

180x138mm (600 x 600 DPI)

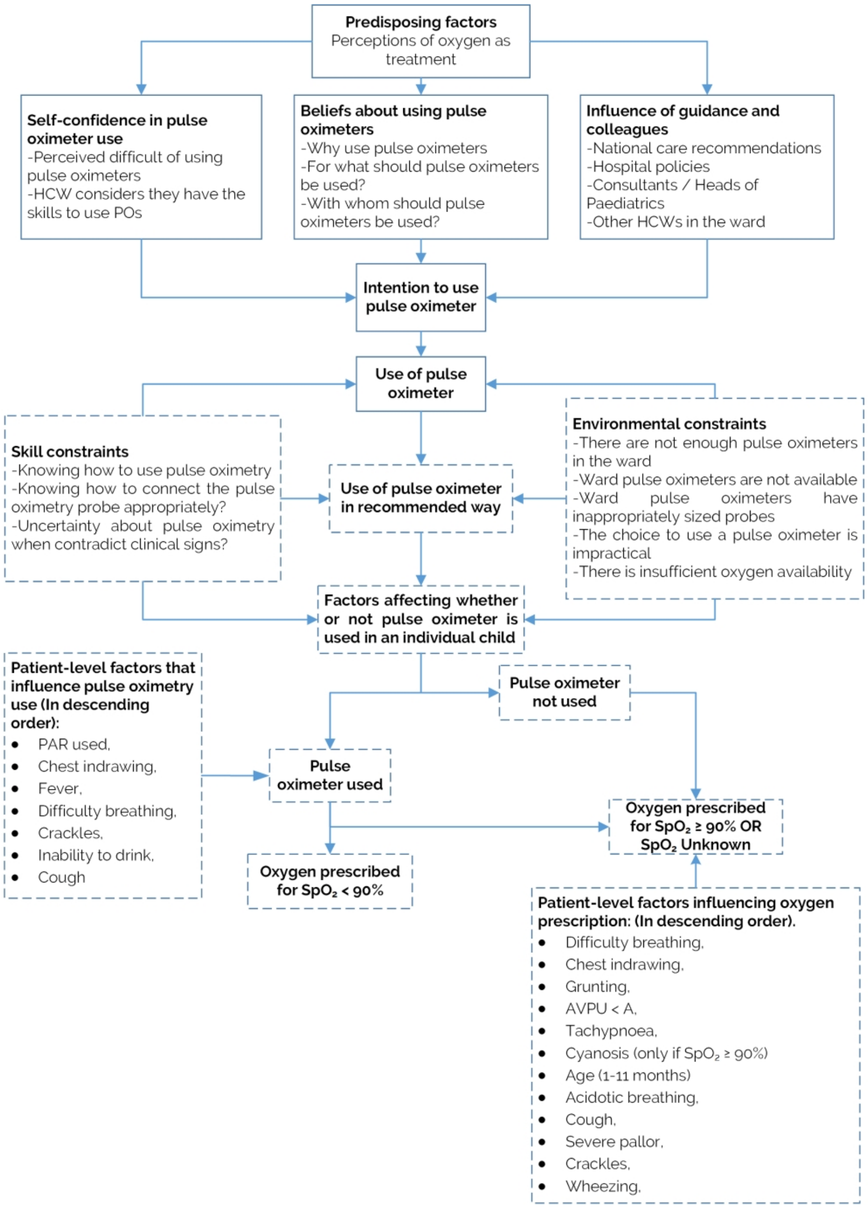

Adapted Integrative Model of Behavioural Prediction (Enoch et al. [10]). Dashes indicate components of the model that are modified or added to reflect the findings from our new analyses. HCW: healthcare worker.

180x248mm (300 x 300 DPI)

Supplementary Material

Supplementary Table 1: Number of patients included into the study per hospital

Hospital	n (%)	Pulse oximeters donated to the hospital by CIN	Current number of pulse oximeters at the paediatric ward	Current status of pulse oximeters*
**H1**	6065 (4.6%)	1	1	All functioning well
**H2**	11636 (8.8%)	1	2	All functioning well
**H3**	9584 (7.2%)	2	2	All functioning well
**H4**	1841 (1.4%)	1	1	All functioning well
**H5**	4021 (3%)	0	0	All oximeters in the neonatal ward
**H6**	13107 (9.9%)	2	2	All functioning well
**H7****	4164 (3.1%)	N/A	N/A	N/A
**H8**	8983 (6.8%)	0	2	Available intermittently. Waiting for supplier to provide longer-time solution
**H9**	9343 (7%)	2	1	One is faulty returned to supplier for repair
**H10**	8764 (6.6%)	2	2	All functioning well
**H11**	11538 (8.7%)	2	2	All functioning well
**H12**	6946 (5.2%)	2	1	One is faulty returned to supplier for repair
**H13**	10055 (7.6%)	1	3	All functioning well
**H14**	8494 (6.4%)	1	0	Stolen. Hospital following up
**H15**	919 (0.7%)	1	1	All functioning well
**H16**	1257 (0.9%)	2	1	One is faulty returned to supplier for repair
**H17**	9196 (6.9%)	0	17	All functional
**H18**	6824 (5.1%)	0	1	Functional. Shared with the Newborn ward.
**Note:**				

For peer review only

**We do not know the brands or specific types; majority of pulse oximeter machines will be portable devices based on authors' experiences of multiple visits to these facilities over this period.*

***Hospital exited from the CIN; Current status of pulse oximetry is therefore not available.*

Supplementary Table 2: Hospital participation in various studies by cluster

Group*	Study	Hospitals
**1**	None	H14
**1**	RTS, S	H18
**1**	RTS, S + SEARCH	H2, H6
**1**	SEARCH	H9, H10, H11, H12, H13
**2**	None	H7, H8, H17
**2**	SEARCH	H3
**3**	RTS, S	H4, H5
**3**	RTS, S + SEARCH	H1
**3**	SEARCH	H16
**3**	None	H15

Note: *The group represents the collection of hospitals in CIN based on joining date. The joining date for Groups 1, 2, and 3, was September 2013, February 2014, from November 2018 respectively. Group 3 hospitals were recruited to aid in increasing hospitals involved in RTS, S and SEARCH studies.

Supplementary Table 3: Discontinuity check due to strike period

(Outcome: Adoption rate per month)

Hospital*	Term**	Estimate***	std.error	statistic	p.value
**H11**	Post Strike Period:I(Time)	2.4660123	0.4525087	5.4496458	**0.012**
**H13**	Post Strike Period:I(Time)	-0.8160995	0.3053929	-2.6722938	0.076
**H10**	Post Strike Period:I(Time)	1.3733025	0.5193376	2.6443346	0.077
**H14**	Post Strike Period:I(Time)	0.2537052	0.1188514	2.1346419	0.122
**H8**	Post Strike Period:I(Time)	-0.9380901	0.4765890	-1.9683421	0.144
**H3**	Post Strike Period:I(Time)	-0.8265731	0.4394489	-1.8809311	0.157
**H18**	Post Strike Period:I(Time)	0.3832935	0.2233908	1.7157982	0.185
**H12**	Post Strike Period:I(Time)	1.2696169	1.0149519	1.2509134	0.300

For peer review only

Supplementary Table 3: Discontinuity check due to strike period (Outcome: Adoption rate per month)					
**Hospital***	**Term****	**Estimate*****	**std.error**	**statistic**	**p.value**
**H6**	Post Strike Period:I(Time)	-0.5999279	0.5146869	-1.1656172	0.328
**H2**	Post Strike Period:I(Time)	-0.3290568	0.2865754	-1.1482383	0.334
**H9**	Post Strike Period:I(Time)	-0.7842015	0.9577486	-0.8187968	0.473
**H17**	Post Strike Period:I(Time)	-0.0119919	0.7642561	-0.0156909	0.988

Note:

*Only hospitals recruited into CIN before the strike

**Time is in months, referencing 3 months before, and 3 months after the strike.

***Estimate is in logit units.

For peer review only

Supplementary Table 4: Complete Case Analysis						
	**Pulse Oximetry Done? (n = 42776)**			**Oxygen Prescribed (n = 42772)**		
**Predictors**	**OR**	**95% CI**	**p-value**	**OR**	**95% CI**	**p-value**
(Intercept)	0.33	0.05 – 2.02	0.231	0.12	0.05 – 0.28	<0.001
**Hospital-level factors**						
Malaria Endemicity Zone [High]	1.03	0.31 – 3.36	0.963	1.02	0.58 – 1.78	0.947
Time (in months)	1.06	1.00 – 1.11	0.033	0.98	0.95 – 1.00	0.076
**Patient-level factors**						
Ref: Patient in any study [No]						
Patient in any study [Pre-study period]‡	1.47	1.30 – 1.67	<0.001	0.9	0.75 – 1.07	0.229
Patient in any study [Yes]	1.15	1.00 – 1.32	0.045	0.99	0.84 – 1.17	0.949
Referral: Yes (Ref: No)	1.05	0.98 – 1.12	0.143	1.24	1.14 – 1.35	<0.001
Readmission: Yes (Ref: No)	1.05	0.97 – 1.13	0.207	0.99	0.89 – 1.11	0.93
Is a weekend admission: Yes (Ref: No)	0.99	0.94 – 1.05	0.761	1.09	1.00 – 1.17	0.04

Supplementary Table 4: Complete Case Analysis

	Pulse Oximetry Done? (n = 42776)			Oxygen Prescribed (n = 42772)		
**Predictors**	**OR**	**95% CI**	**p-value**	**OR**	**95% CI**	**p-value**
PAR used: Yes (Ref: No)	0.7	0.46 – 1.06	0.09	0.44	0.22 – 0.88	0.02
Time (in months)* PAR used: Yes (Ref: No)	1.01	1.00 – 1.03	0.051	1.03	1.01 – 1.06	0.005
Ref: Age [> 59 months]						
Age [1-11 months]	0.75	0.64 – 0.89	0.001	0.73	0.58 – 0.94	0.013
Age [12-59 months]	0.72	0.61 – 0.84	<0.001	0.56	0.44 – 0.71	<0.001
Female: Yes (Ref: No)	1.03	0.98 – 1.08	0.229	1.03	0.96 – 1.10	0.412
Fever: Yes (Ref: No)	1.03	0.97 – 1.11	0.324	0.81	0.74 – 0.89	<0.001
Cough: Yes (Ref: No)	0.99	0.94 – 1.04	0.626	1.3	1.19 – 1.41	<0.001
Difficulty Breathing: Yes (Ref: No)	1.09	1.02 – 1.15	0.007	2.54	2.34 – 2.76	<0.001
Stridor: Yes (Ref: No)	1.01	0.86 – 1.19	0.926	0.97	0.82 – 1.15	0.739
Cyanosis: Yes (Ref: No)	0.91	0.69 – 1.20	0.499	1.62	1.25 – 2.10	<0.001
Indrawing: Yes (Ref: No)	1.38	1.29 – 1.48	<0.001	2.12	1.95 – 2.31	<0.001
Grunting: Yes (Ref: No)	0.88	0.81 – 0.95	0.001	1.93	1.78 – 2.08	<0.001
Acidotic Breathing: Yes (Ref: No)	0.9	0.77 – 1.04	0.161	1.31	1.13 – 1.51	<0.001
Wheezing: Yes (Ref: No)	1.08	0.97 – 1.20	0.14	0.98	0.89 – 1.09	0.769
Crackles: Yes (Ref: No)	1.06	0.99 – 1.13	0.115	1.2	1.12 – 1.30	<0.001
Tachypnoea: Yes (Ref: No)	1.09	1.03 – 1.15	0.002	1.64	1.51 – 1.77	<0.001
Alert (AVPU < A): Yes (Ref: No)	0.89	0.79 – 1.00	0.042	1.77	1.57 – 2.01	<0.001
Inability to drink: Yes (Ref: No)	0.96	0.89 – 1.03	0.226	0.76	0.69 – 0.83	<0.001
Ref: Pallor [None]						
Pallor [Mild/Moderate]	1.02	0.94 – 1.12	0.574	1.23	1.10 – 1.37	<0.001
Pallor [Severe]	0.94	0.83 – 1.07	0.336	1.18	0.99 – 1.40	0.067
Ref: WAZ [Normal]						
WAZ [Low]	0.97	0.90 – 1.04	0.388	0.95	0.86 – 1.05	0.327
WAZ [Very Low]	1	0.93 – 1.08	0.97	0.9	0.82 – 0.99	0.033

For peer review only

Supplementary Table 4: Complete Case Analysis						
	Pulse Oximetry Done? (n = 42776)			Oxygen Prescribed (n = 42772)		
Predictors	OR	95% CI	p-value	OR	95% CI	p-value
Slow Capillary Refill: Yes (Ref: No)	0.76	0.61 – 0.94	0.013	0.8	0.60 – 1.06	0.119
Ref: Hypoxic [Unknown]						
Hypoxic [No]				0.54	0.50 – 0.59	<0.001
Hypoxic [Yes]				1.71	1.53 – 1.92	<0.001
Random effects						
**σ^2**	3.29			3.29		
**τ_{00}**	12.40 Hospital			0.23 Hospital		
**τ_{11}**	0.01 Hospital.Time			0.00 Hospital.Time		
**ρ_{01}**	-0.96 Hospital			-0.11 Hospital		
**ICC**	0.57			0.23		
**N**	17 Hospital			17 Hospital		
**Marginal R^2 / Conditional R^2**	0.144/ 0.631			0.271 / 0.442		

Supplementary Table 5: Oxygen prescription for $SpO_2 \geq 90\%$ OR SpO_2 Unknown						
	MICE model*			Complete Case Analysis**		
Predictors	OR	95% CI	p-value	OR	95% CI	p-value
(Intercept)	0.04	0.02 - 0.08	<0.001	0.08	0.03 – 0.19	<0.001
**Hospital-level factors**						
Malaria Endemicity Zone [High]	1.61	0.9 - 2.87	0.106	1.12	0.61 – 2.04	0.719
Time (in months)	1.01	1 - 1.02	0.152	0.97	0.95 – 1.00	0.032
**Patient-level factors**						
Ref: Patient in any study [No]						

For peer review only

Supplementary Table 5: Oxygen prescription for $SpO_2 \geq 90\%$ OR SpO_2 Unknown						
	MICE model*			Complete Case Analysis**		
**Predictors**	**OR**	**95% CI**	**p-value**	**OR**	**95% CI**	**p-value**
Patient in any study [Pre-study period]‡	0.99	0.86 - 1.12	0.824	0.88	0.73 – 1.06	0.168
Patient in any study [Yes]	0.94	0.83 - 1.07	0.349	0.92	0.78 – 1.10	0.369
Referral: Yes (Ref: No)	1.18	1.11 - 1.26	<0.001	1.28	1.17 – 1.40	<0.001
Readmission: Yes (Ref: No)	1.07	1 - 1.16	0.066	1.01	0.90 – 1.14	0.846
Is a weekend admission: Yes (Ref: No)	1.08	1.02 - 1.14	0.005	1.11	1.02 – 1.21	0.014
PAR used: Yes (Ref: No)	1.18	0.96 - 1.44	0.120	0.46	0.22 – 0.96	0.039
Time (in months)*PAR used: Yes (Ref: No)	1	1 - 1.01	0.560	1.03	1.01 – 1.06	0.013
Ref: Age [> 59 months]						
Age [< 1 month]	1.9	1.56 - 2.3	<0.001			
Age [1-11 months]	1.42	1.31 - 1.54	<0.001	0.79	0.61 – 1.03	0.078
Age [12-59 months]	1.06	0.98 - 1.15	0.163	0.59	0.46 – 0.76	<0.001
Female: Yes (Ref: No)	1.03	0.98 - 1.07	0.288	1.04	0.97 – 1.12	0.256
Fever: Yes (Ref: No)	0.76	0.72 - 0.81	<0.001	0.8	0.72 – 0.89	<0.001
Cough: Yes (Ref: No)	1.36	1.28 - 1.44	<0.001	1.3	1.19 – 1.42	<0.001
Difficulty Breathing: Yes (Ref: No)	2.88	2.72 - 3.05	<0.001	2.66	2.43 – 2.91	<0.001
Stridor: Yes (Ref: No)	1.12	1.01 - 1.26	0.039	0.99	0.83 – 1.17	0.865
Cyanosis: Yes (Ref: No)	1.61	1.36 - 1.91	<0.001	1.94	1.48 – 2.56	<0.001
Indrawing: Yes (Ref: No)	2.48	2.34 - 2.63	<0.001	2.27	2.07 – 2.48	<0.001
Grunting: Yes (Ref: No)	2.03	1.92 - 2.15	<0.001	2.09	1.92 – 2.27	<0.001
Acidotic Breathing: Yes (Ref: No)	1.37	1.24 - 1.51	<0.001	1.38	1.19 – 1.61	<0.001
Wheezing: Yes (Ref: No)	1.17	1.08 - 1.26	<0.001	1.04	0.93 – 1.16	0.542
Crackles: Yes (Ref: No)	1.27	1.2 - 1.34	<0.001	1.21	1.12 – 1.32	<0.001
Tachypnoea: Yes (Ref: No)	1.63	1.54 - 1.72	<0.001	1.7	1.56 – 1.85	<0.001
Alert (AVPU < A): Yes (Ref: No)	2.02	1.86 - 2.18	<0.001	1.83	1.60 – 2.08	<0.001
Inability to drink: Yes (Ref: No)	0.8	0.75 - 0.85	<0.001	0.76	0.69 – 0.84	<0.001

For peer review only

Supplementary Table 5: Oxygen prescription for $SpO_2 \geq 90\%$ OR SpO_2 Unknown						
	MICE model*			Complete Case Analysis**		
**Predictors**	**OR**	**95% CI**	**p-value**	**OR**	**95% CI**	**p-value**
Ref: Pallor [None]						
Pallor [Mild/Moderate]	1.11	1.04 - 1.2	<0.001	1.26	1.12 – 1.41	<0.001
Pallor [Severe]	1.34	1.22 - 1.46	<0.001	1.14	0.95 – 1.38	0.153
Ref: WAZ [Normal]						
WAZ [Low]	1	0.93 - 1.07	0.952	0.97	0.87 – 1.08	0.596
WAZ [Very Low]	0.96	0.89 - 1.03	0.273	0.92	0.83 – 1.02	0.122
Slow Capillary Refill: Yes (Ref: No)	1.03	0.88 - 1.2	0.708	0.87	0.65 – 1.18	0.371

Note: ******n=123,952; 4275 cases omitted from MICE analysis because of missing outcome (Figure 4)*

*******n=40,491*

Supplementary Table 6: Admission diagnoses for patients getting oxygen with pulse oximetry ≥ 90% at admission.	
**Diagnosis**	**Documented Cases, N (%)**
Pneumonia	18668 (34.14%)
Malaria	16559 (30.28%)
Dehydration	10641 (19.46%)
Diarrhoea	10592 (19.37%)
Anaemia	8018 (14.66%)
Meningitis	5875 (10.74%)
Malnutrition	5351 (9.78%)
Sickle Cell	2380 (4.35%)
Asthma	1240 (2.27%)
Rickets	1084 (1.98%)
TB	907 (1.6s6%)
Sepsis	708 (1.29%)
HIV	716 (1.31%)

For peer review only

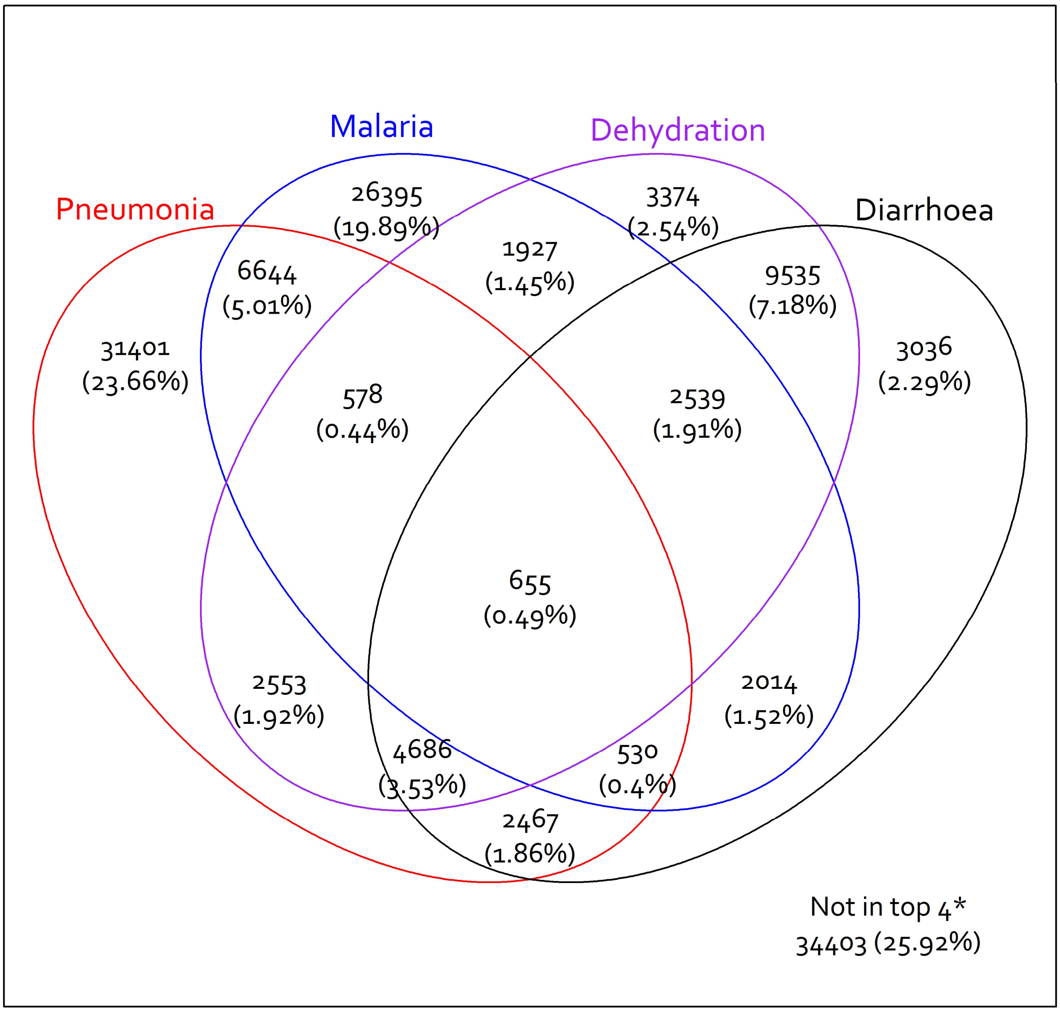

Supplementary Figure 1: Common admission diagnoses in CIN hospitals.

From the linear growth-curve model, the random intercept conveys the hospital's average pulse oximetry adoption rate over the time period in months it has participated in CIN. The random slope conveys the hospital's average monthly increase or decrease in adoption rate over the time period they have been part of the CIN. The baseline for the random intercept and slope in the growth curve model is the overall level of the response which is allowed to vary between hospitals after controlling for covariates.

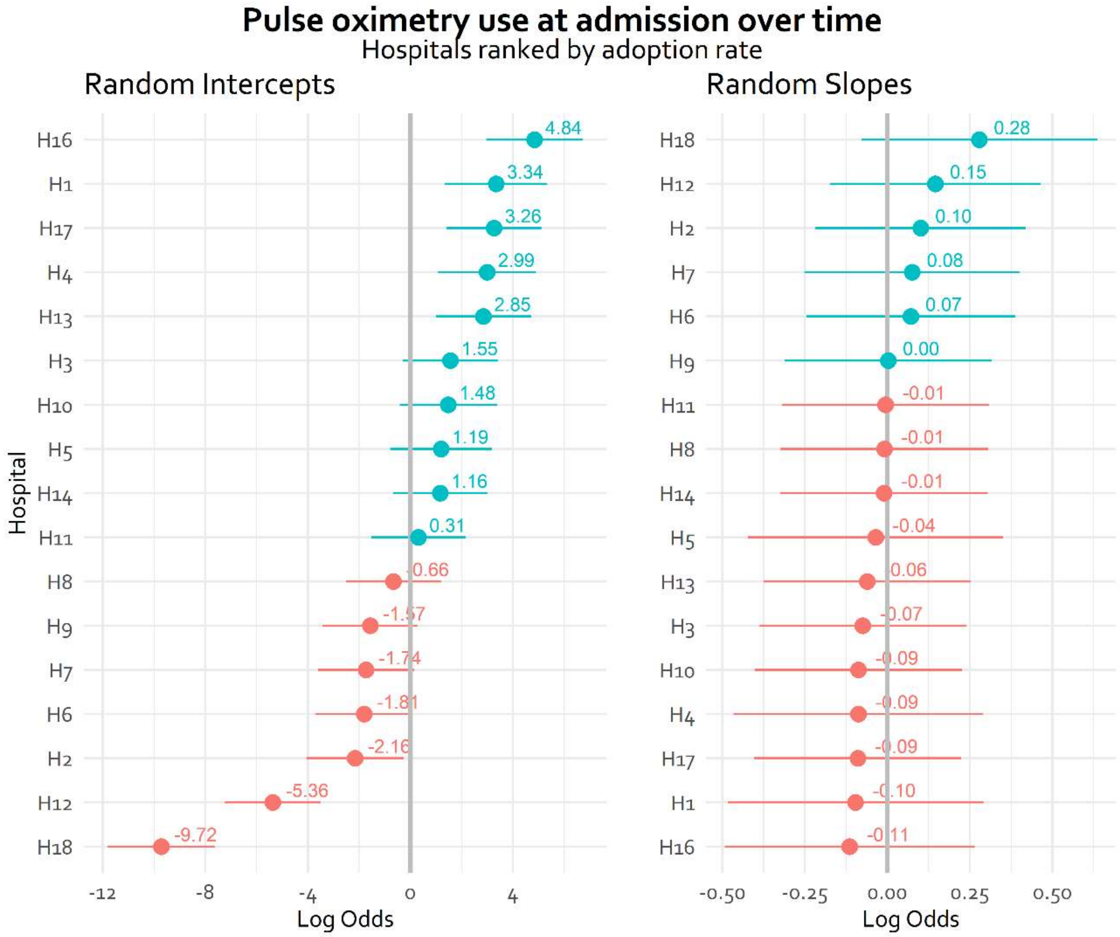

Supplementary Figure 2: Ranking of hospitals pulse oximetry use at admission

view only

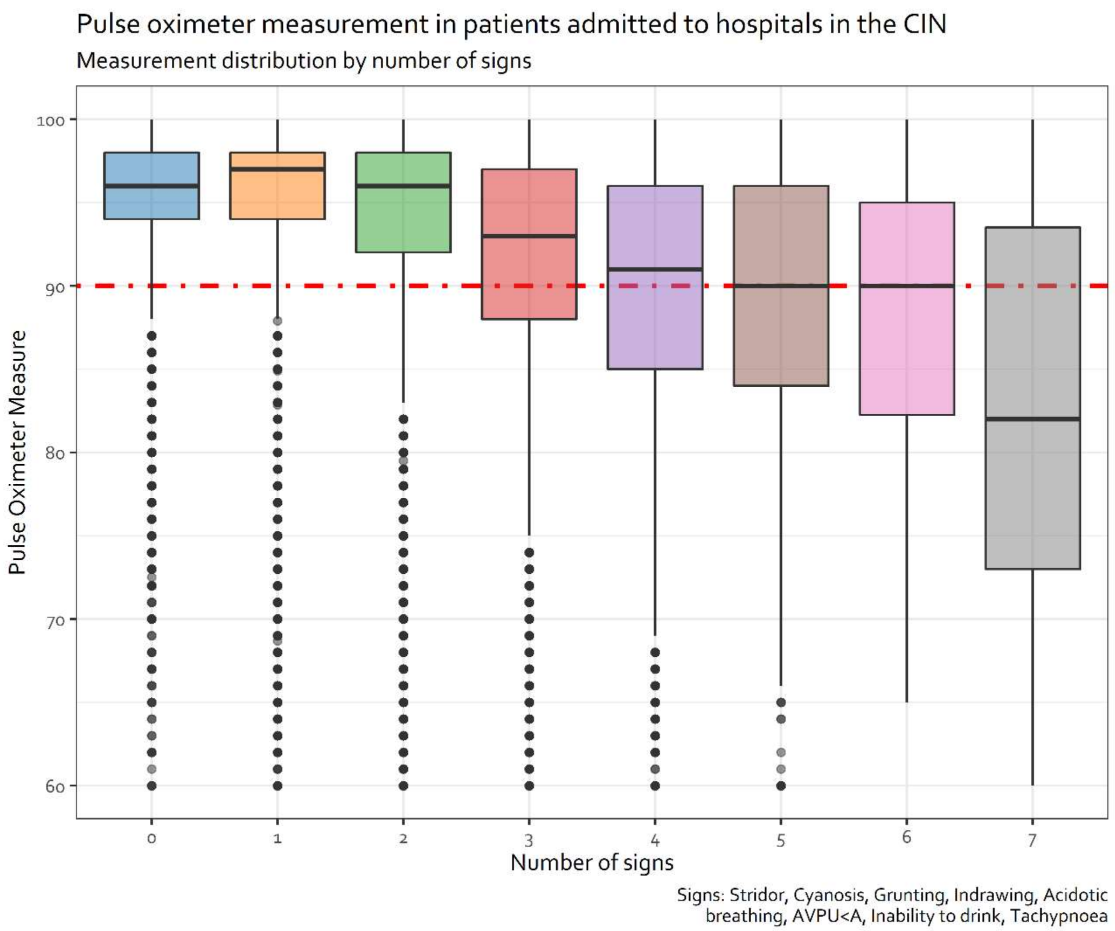

Supplementary Figure 3: Oxygen saturation measure by the number of respiratory distress symptoms

STROBE Statement—checklist of items that should be included in reports of observational studies

	Item No	Recommendation	Page No
**Title and abstract**	1	(*a*) Indicate the study's design with a commonly used term in the title or the abstract	1
		(*b*) Provide in the abstract an informative and balanced summary of what was done and what was found	1-2
**Introduction**			
Background/rationale	2	Explain the scientific background and rationale for the investigation being reported	55-76
Objectives	3	State specific objectives, including any prespecified hypotheses	76-79, 193-196
**Methods**			
Study design	4	Present key elements of study design early in the paper	92-132
Setting	5	Describe the setting, locations, and relevant dates, including periods of recruitment, exposure, follow-up, and data collection	92-132
Participants	6	(*a*) *Cohort study*—Give the eligibility criteria, and the sources and methods of selection of participants. Describe methods of follow-up<br>*Case-control study*—Give the eligibility criteria, and the sources and methods of case ascertainment and control selection. Give the rationale for the choice of cases and controls<br>*Cross-sectional study*—Give the eligibility criteria, and the sources and methods of selection of participants	133-143
		(*b*) *Cohort study*—For matched studies, give matching criteria and number of exposed and unexposed<br>*Case-control study*—For matched studies, give matching criteria and the number of controls per case	
Variables	7	Clearly define all outcomes, exposures, predictors, potential confounders, and effect modifiers. Give diagnostic criteria, if applicable	169-189
Data sources/ measurement	8*	For each variable of interest, give sources of data and details of methods of assessment (measurement). Describe comparability of assessment methods if there is more than one group	164-178
Bias	9	Describe any efforts to address potential sources of bias	205-213
Study size	10	Explain how the study size was arrived at	134-143
Quantitative variables	11	Explain how quantitative variables were handled in the analyses. If applicable, describe which groupings were chosen and why	169-189
Statistical methods	12	(*a*) Describe all statistical methods, including those used to control for confounding	178-213
		(*b*) Describe any methods used to examine subgroups and interactions	190-196
		(*c*) Explain how missing data were addressed	209-213

For peer review only - http://bmjopen.bmj.com/site/about/guidelines.xhtml

(*d*) *Cohort study*—If applicable, explain how loss to follow-up was addressed<br>*Case-control study*—If applicable, explain how matching of cases and controls was addressed<br>*Cross-sectional study*—If applicable, describe analytical methods taking account of sampling strategy	197-213
(*e*) Describe any sensitivity analyses	

Continued on next page

For peer review only

**Results**			
Participants	13*	(a) Report numbers of individuals at each stage of study—eg numbers potentially eligible, examined for eligibility, confirmed eligible, included in the study, completing follow-up, and analysed	215 - 221
		(b) Give reasons for non-participation at each stage	Figure 1
		(c) Consider use of a flow diagram	Figure 1
Descriptive data	14*	(a) Give characteristics of study participants (eg demographic, clinical, social) and information on exposures and potential confounders	226-293
		(b) Indicate number of participants with missing data for each variable of interest	Table 2
		(c) *Cohort study*—Summarise follow-up time (eg, average and total amount)	
Outcome data	15*	*Cohort study*—Report numbers of outcome events or summary measures over time	Table 2, Figures 2-3
		Case-control study—Report numbers in each exposure category, or summary measures of exposure	
		Cross-sectional study—Report numbers of outcome events or summary measures	Table 2, Figure 4, 279-286
Main results	16	(*a*) Give unadjusted estimates and, if applicable, confounder-adjusted estimates and their precision (eg, 95% confidence interval). Make clear which confounders were adjusted for and why they were included	294-357
		(*b*) Report category boundaries when continuous variables were categorized	279-280
		(*c*) If relevant, consider translating estimates of relative risk into absolute risk for a meaningful time period	
Other analyses	17	Report other analyses done—eg analyses of subgroups and interactions, and sensitivity analyses	241- 247, 331-333, Supplementary Tables 3 - 5, Table 3
**Discussion**			
Key results	18	Summarise key results with reference to study objectives	359-365
Limitations	19	Discuss limitations of the study, taking into account sources of potential bias or imprecision. Discuss both direction and magnitude of any potential bias	440-459
Interpretation	20	Give a cautious overall interpretation of results considering objectives, limitations, multiplicity of analyses, results from similar studies, and other relevant evidence	366-431
Generalisability	21	Discuss the generalisability (external validity) of the study results	433-442
**Other information**			
Funding	22	Give the source of funding and the role of the funders for the present study and, if applicable, for the original study on which the present article is based	520-534

*Give information separately for cases and controls in case-control studies and, if applicable, for exposed and unexposed groups in cohort and cross-sectional studies.

For peer review only - http://bmjopen.bmj.com/site/about/guidelines.xhtml

For peer review only

Note: An Explanation and Elaboration article discusses each checklist item and gives methodological background and published examples of transparent reporting. The STROBE checklist is best used in conjunction with this article (freely available on the Web sites of PLoS Medicine at http://www.plosmedicine.org/, Annals of Internal Medicine at http://www.annals.org/, and Epidemiology at http://www.epidem.com/). Information on the STROBE Initiative is available at www.strobe-statement.org.

For peer review only

For peer review only - http://bmjopen.bmj.com/site/about/guidelines.xhtml

BMJ Open

Pulse oximetry adoption and oxygen orders at paediatric admission over 7 years in Kenya: A multi-hospital retrospective cohort study

Journal:	*BMJ Open*
Manuscript ID	bmjopen-2021-050995.R2
Article Type:	Original research
Date Submitted by the Author:	02-Aug-2021

Complete List of Authors:	Tuti, Timothy; University of Oxford, Kellogg College; KEMRI-Wellcome Trust Research Programme, Health Services Unit<br>Aluvaala, Jalemba ; Kenya Medical Research Institute/Wellcome Trust Research Programme, Health Services Unit; University of Nairobi, Department of Paediatrics and Child Health<br>Akech, Samuel; KEMRI-Wellcome Trust Research Programme Nairobi<br>Agweyu, Ambrose; KEMRI-Wellcome Trust Research Programme Nairobi, Health Services Unit<br>Irimu, Grace ; Kenya Medical Research Institute, Health Services Unit; University of Nairobi College of Health Sciences, Department of Paediatrics and Child Health<br>English, Mike; University of Oxford, Nuffield Department of Medicine and Department of Paediatrics; KEMRI-Wellcome Trust Research Programme Nairobi, Health Services Unit

**Primary Subject Heading**:	Paediatrics
Secondary Subject Heading:	Epidemiology, Global health, Health services research, Paediatrics
Keywords:	Quality in health care < HEALTH SERVICES ADMINISTRATION & MANAGEMENT, PAEDIATRICS, AUDIT, EPIDEMIOLOGY, Protocols & guidelines < HEALTH SERVICES ADMINISTRATION & MANAGEMENT, Tropical medicine < INFECTIOUS DISEASES

SCHOLARONE™
Manuscripts

BMJ

I, the Submitting Author has the right to grant and does grant on behalf of all authors of the Work (as defined in the below author licence), an exclusive licence and/or a non-exclusive licence for contributions from authors who are: i) UK Crown employees; ii) where BMJ has agreed a CC-BY licence shall apply, and/or iii) in accordance with the terms applicable for US Federal Government officers or employees acting as part of their official duties; on a worldwide, perpetual, irrevocable, royalty-free basis to BMJ Publishing Group Ltd ("BMJ") its licensees and where the relevant Journal is co-owned by BMJ to the co-owners of the Journal, to publish the Work in this journal and any other BMJ products and to exploit all rights, as set out in our licence.

The Submitting Author accepts and understands that any supply made under these terms is made by BMJ to the Submitting Author unless you are acting as an employee on behalf of your employer or a postgraduate student of an affiliated institution which is paying any applicable article publishing charge ("APC") for Open Access articles. Where the Submitting Author wishes to make the Work available on an Open Access basis (and intends to pay the relevant APC), the terms of reuse of such Open Access shall be governed by a Creative Commons licence – details of these licences and which Creative Commons licence will apply to this Work are set out in our licence referred to above.

Other than as permitted in any relevant BMJ Author's Self Archiving Policies, I confirm this Work has not been accepted for publication elsewhere, is not being considered for publication elsewhere and does not duplicate material already published. I confirm all authors consent to publication of this Work and authorise the granting of this licence.

For peer review only

Pulse oximetry adoption and oxygen orders at paediatric admission over 7 years in Kenya: A multi-hospital retrospective cohort study

Timothy Tuti[1*], Jalemba Aluvaala[1,2], Samuel Akech[1], Ambrose Agweyu[1], Grace Irimu[1,2], Mike English[1,3] on behalf of the Clinical Information Network,

[1] Health Services Unit, KEMRI-Wellcome Trust Research Programme

[2] Department of Paediatrics and Child Health, University of Nairobi, Nairobi, Kenya

[3] Oxford Centre for Global Health Research, University of Oxford, Oxford, UK

*Corresponding Author: P.O. Box 43640, Nairobi, Kenya; Email: timothy.tuti@kellogg.ox.ac.uk

Abstract:

Objectives: To characterise adoption and explore specific clinical and patient factors that might influence pulse oximetry and oxygen use in low- and middle-income countries (LMICs) over time; To highlight useful considerations for entities working on programs to improve access to pulse oximetry and oxygen.

Design: A multi-hospital retrospective cohort study

Settings: All admissions (n=132737) to paediatric wards of 18 purposely selected public hospitals in Kenya that joined a Clinical Information Network (CIN) between March 2014 and December 2020.

Outcomes: Pulse oximetry use and oxygen prescription on admission; We performed growth-curve modelling to investigate the association of patient factors with study outcomes over time while adjusting for hospital factors.

Results: Overall, pulse oximetry was used in 48.8% (64722/132737) of all admission cases. Use rose on average with each month of participation in the CIN (OR: 1.11, 95% CI 1.05–1.18) but patterns of adoption were highly variable across hospitals suggesting important factors at hospital level influence

For peer review only

For peer review only - http://bmjopen.bmj.com/site/about/guidelines.xhtml

use of pulse oximetry. Of those with pulse oximetry measurement, 7% (4510 /64722) had hypoxaemia (SpO_2 <90%). Across the same period, 8.6% (11428/132737) had oxygen prescribed but in 87%, pulse oximetry was either not done or the hypoxemia threshold (SpO_2 <90%) was not met. Lower chest-wall indrawing and other respiratory symptoms were associated with pulse oximetry use at admission and were also associated with oxygen prescription in the absence of pulse oximetry or hypoxemia.

Conclusion: The adoption of pulse oximetry recommended in international guidelines for assessing children with severe illness has been slow and erratic, reflecting system and organisational weaknesses. Most oxygen orders at admission seem driven by clinical and situational factors other than the presence of hypoxemia. Programmes aiming to implement pulse oximetry and oxygen systems will likely need a long-term vision to promote adoption, guideline development and adherence and continuously examine impact.

Keywords: Quality Improvement, Health Systems, Paediatric Care, Diagnostics, Evaluation, Hospital

Word Count: 5,120

Strengths and limitations

- The data presented are a rare example of long-term tracking of hospital practice in a routine LMIC setting presenting a useful illustration of the process of pulse oximetry adoption in LMIC hospitals that has broader implications for thinking on technology adoption.
- We employ a complex modelling framework to explore patient- and hospital- level factors influencing outcomes of interest at admission (pulse oximetry done, and oxygen prescribed) over time that accommodate and account for between hospital variability, within hospital non-linear trends in the outcomes over time and entry into the clinical network at different time points.
- We have no contemporaneous data from Kenyan settings that do not belong to the Clinical Information Network for comparison with most other studies exploring the use of pulse oximetry essentially being cross-sectional.

For peer review only

For peer review only - http://bmjopen.bmj.com/site/about/guidelines.xhtml

Introduction

Background

Compared to clinical signs (e.g. central cyanosis, indrawing), pulse oximetry is felt to be easy to use and posited to be at least 20% more accurate in identifying children at risk of hypoxemia (defined as a blood oxygen saturation of <90%) [1, 2]. As pulse oximetry is an inexpensive tool it is now recommended for guiding the assessment of illness severity and treatment for respiratory illnesses, including increasing emphasis on its role for screening children with respiratory illness in primary care in LMIC [1, 2]. As hypoxemia may be found in all severe childhood illnesses such as sepsis, meningitis, severe acute malnutrition and malaria its use is widely recommended as a screening tool in all paediatric admissions as a fifth vital sign. The advent of COVID-19 has further invigorated programmatic efforts to scale up pulse oximetry together with improved oxygen delivery systems [3-9].

Despite being recommended for many years, there is little long-term data on the adoption of pulse oximetry in LMIC, even from hospitals. Data on use during the COVID-19 pandemic are also lacking from such settings as are large studies from routine settings on whether pulse oximeter readings influence the use of oxygen. We and others have described that adoption is undermined by system factors such as inadequate supply and repair of oximeters and if healthcare workers have insufficient training on when, how, and why to use them and interpret their results [9, 10]. This compounds wider and similar problems in managing oxygen systems themselves [11]. Our earlier work used data from 7 Kenyan hospitals between September 2013 and February 2016. Here we update and extend these analyses to include data from paediatric admissions in a growing network of Kenyan hospitals before and after prolonged national health workers strikes in 2017, including data obtained during the COVID-19 pandemic [12]. The objective of this study was to characterise adoption and explore specific patient, clinical and hospital factors that might influence pulse oximetry and oxygen use and highlight useful considerations for entities working on programs to improve access to pulse oximetry and oxygen [13-15].

For peer review only - http://bmjopen.bmj.com/site/about/guidelines.xhtml

Methods

Ethics and Reporting

The reporting of this observational study follows the Strengthening of reporting of observational studies in epidemiology (STROBE) statement [16]. The Scientific and Ethics Review Unit of the Kenya Medical Research Institute (KEMRI) approved the collection of the de-identified data that provides the basis for this study as part of the Clinical Information Network (CIN). The CIN is run in partnership with the Ministry of Health and participating hospitals with aims to improve the quality of routine paediatric hospital data for use in improvement activities, observational and interventional research [17, 18]. Individual consent for access to de-identified patient data was not required.

Patient and Public Involvement

No patients were involved in the design, conduct, reporting, or dissemination plans of our research except through the KEMRI ethical review process where they have representatives.

Study design and setting

From a broad context perspective, in many LMICs including Kenya, hospital management and monitoring systems are weak, with major human and material resource constraints. These challenges affect hospitals' delivery of inpatient maternal, surgical, and adult medical care as well as paediatric and neonatal care. Consequently, there is very limited organisational and resource slack to mobilise for any new purpose. Interventions such as oximetry and oxygen seeking to achieve large scale change must therefore either consider how to mobilise new resources or consider what is achievable with limited resources [19, 20]. We have described the broader context of the Kenyan healthcare system with reference to the paediatric burden of disease in great detail elsewhere [19, 20].

We report a retrospective cohort study of 18 public hospitals in Kenya largely providing first-referral level care predominantly admitting patients that present directly to the facility, and purposefully selected to be of at least moderate size and representative of different malaria transmission zones. Hospitals joined the CIN at different calendar time points between 2013 and 2017. Few of their patients may be formally referred from primacy care facilities but there are few functional referral

For peer review only

mechanisms such as ambulance systems [21]. Pulse oximetry has generally not been available outside hospitals in the public sector [22]. Pneumonia is the major killer of children across the country except in settings where malaria is highly endemic (9/18 hospitals in this study) [21].

The hospitals receive 3 monthly clinical audit and feedback reports on the quality of care they provide for common conditions [23]. Paediatric team leaders (paediatricians and nurses) met face to face once or twice annually until 2019 (before the pandemic) to discuss these reports and how to improve clinical care. From 2018, participation in more specific research studies was also discussed in meetings with hospitals. This resulted in two studies being initiated in subsets of the CIN hospitals which might have influenced the adoption of pulse oximetry. Hospital participation in these studies was not mutually exclusive: a hospital could be part of one, both or none of the research studies. Not all hospitals recruited in the same study were recruited at the same time. Summary details of these studies are provided in Table 1 with greater detail on which hospitals participated in the studies provided in Supplementary Tables 1 and 2. The 18 hospitals included in the study had a median of 1 pulse oximeter(s) per paediatric ward (IQR: 1 - 3) (Supplementary Table 1).

Table 1: Description of studies undertaken in CIN hospitals

Study name	Study site selection rationale and period	Goal	Extra resources provided to hospitals (H) by projects*	Extra notes
**RTS, S/AS01 trial: (ClinicalTrials.gov Identifier: NCT03806465)**	Study start: December 2018<br><br>Site Selection: By MoH/WHO, selecting high volume public hospitals in regions getting the vaccine.	The evaluation of the feasibility, safety and impact of the RTS, S/AS01 malaria vaccine in paediatric cases in Kenya	One extra healthcare worker provided per paediatric ward.<br><br>Extra pulse oximetry equipment provided per ward:<br>• One item: H4	Clinician role: Promote compliance with guidelines for meningitis and malaria including complete assessment and investigations including the performance of lumbar punctures, and consenting participants for storage of cerebrospinal fluid (CSF) storage

For peer review only - http://bmjopen.bmj.com/site/about/guidelines.xhtml

**SEARCH trial (ClinicalTrials.gov Identifier: NCT04041791)**	Started in two phases:<br>• March 2019 Hospitals: H3, H9, H12, H13, H16<br>• June 2019 Hospitals: H1, H2, H6, H10, H11<br><br>Site selection: From the expected size of pneumonia population, the geographical balance of hospitals and support from local hospital teams	Pragmatic factorial individually randomized controlled trial to compare alternative antibiotics and alternative modes of fluid therapy for children with severe pneumonia	One extra healthcare worker provided per participating hospital.<br><br>Extra pulse oximetry equipment provided to the hospital:<br>• Two items: H3, H6, H9, H10, H11, H12, H13, H16<br>• One item: H1, H2	Clinician role: Promote compliance with the study protocol, including completing patient assessment, investigations and diagnoses. Ensure appropriate administration of study interventions.<br><br>Recruitment of study participants was suspended on the 8th of April 2020 due to COVID-19 pandemic.<br><br>Adult inpatient surveillance in addition to paediatric surveillance began on the 4th of May 2020.
**Note:**<br>*H15 got one pulse oximeter in June 2020 even though it was not part of any study*<br>** We were not able to track and record introduction of pulse oximeters procured directly by hospitals or other sources, or those personally owned by healthcare workers, nor ability to repair existing pulse oximeters when required.*				

Recording of pulse oximetry values has been included as part of a structured Paediatric Admission Record (PAR) [24] used since 2013. Hospitals joining the CIN agree to provide the PAR themselves and promote its use – the purpose of the PAR is to prompt admitting clinicians to fully assess children and rapidly document their findings [24]. Emergency Triage Assessment and Treatment plus Admission Care (ETAT+) training [25] has been used in Kenya since 2008 and was adapted in 2013 so that pulse oximetry was a recommended part of the assessment in all sick children and especially those with danger signs. Many of the junior and senior medical staff in CIN hospitals would have received ETAT+ training (e.g. as an undergraduate or postgraduate course or as in-service training) [25]. Additionally, the Basic Paediatric Protocols that are widely disseminated have specifically referred to the use of pulse oximetry – if available – for all pneumonia cases since 2016 [26]. The promotion of PAR use by hospitals, adaption and scale up of ETAT+ training, and dissemination of the basic paediatric protocols

For peer review only - http://bmjopen.bmj.com/site/about/guidelines.xhtml

to clinicians nationally are posited to have a system-wide effect on the adoption of pulse oximetry use.

Study participants

All medical admissions to paediatric wards (ages 0 to 13 years), but not those admitted to specific newborn units, to the selected hospitals between February 1st 2014, or the date at which a hospital joined CIN, and December 31st 2020 were eligible for inclusion in this study. We excluded children whose admission or discharge dates were missing or improbable (e.g. discharge date is earlier than admission date), and those whose admission fell within 2017, a period of prolonged health worker strikes that resulted in major disruption to health care delivery [12]. The expectation was that all would have pulse oximetry performed at admission given they were sick enough to warrant inpatient care but amongst these we also report the proportion of children with danger signs comprising any level of altered consciousness or with recorded signs of respiratory distress especially deserving of pulse oximetry [10, 26, 27].

Data sources and management

Methods of collection and cleaning of data in the CIN are reported in detail elsewhere [28]. Clinical data for paediatric admissions to the hospitals within the CIN are captured through PAR forms [27] that are approved by the Ministry of Health. The PAR prompts the clinician with a checklist of fields including patient biodata, clinical assessment, admission and discharge diagnoses, treatments, and to record outcome (survival or death). The CIN supports one data clerk in each hospital to extract data from paper medical records, nursing charts, treatment charts, and available laboratory reports each day after children's discharge into the primary data collection tool developed in Research Electronic Data Capture (REDCap). Automated error checking happens at the point of entry by daily review, every week centrally and both are complemented by regular external data quality assurance reviews [28]. A minimal dataset – which is unsuitable for these pulse oximetry analyses - is collected for (1) all admissions with a burn or a surgical diagnosis to the paediatric ward(s), (2) admissions during major holiday breaks, (3) admissions when the data clerk was on leave, and (4) on a random selection of

For peer review only

For peer review only - http://bmjopen.bmj.com/site/about/guidelines.xhtml

records in hospitals where the workload is very high. This process is explained in detail elsewhere [28, 29].

Descriptive analysis

To characterise adoption of pulse oximetry at admission over time we use pooled data from the period February 2014 to November 2018 (excluding 2017) from 13 network hospitals. From December 2018 data were available from 17 hospitals after 5 joined the CIN linked to new studies, and one (H7) exited. To explore patterns in more detail we employed two approaches: i) for the period from December 2018 to December 2020 we plot data pooled for hospital sub-groups depending on which research studies they were taking part in as these might influence pulse oximetry use; ii) We plot adoption within each hospital for the number of months it was part of the network, again excluding 2017 where relevant. This latter approach was used to reveal the extent of variability in adoption at the hospital level.

For the individual patient population, we tabulated and summarised categorical data as proportions, with continuous variables reported as medians (IQR) if they were not normally distributed. From the included paediatric admission patient population data, we report the prevalence of important clinical features such as respiratory distress symptoms (e.g. central cyanosis, indrawing, grunting, and difficulty breathing), circulatory symptoms (e.g. slow capillary refill, less than three seconds), level of consciousness, and whether the patient was being readmitted or referred to the hospital. We also report the prevalence of summary clinical features such as having any sign of respiratory distress and any danger sign and the proportion for whom oxygen was ordered as a treatment at admission.

Statistical methods

To examine two binary outcomes, (1) pulse oximetry used and, (2) oxygen ordered at admission, we modelled individual patient data. Our key explanatory variable was time as part of (or exposure to) the CIN, computed as the number of months since joining the CIN for a specific hospital. This allowed associations with these outcomes to be explored in hospitals while accounting for clustering by both

For peer review only

For peer review only - http://bmjopen.bmj.com/site/about/guidelines.xhtml

hospital and time in months since the hospital joined the CIN; the analysis of pulse oximetry use employs a much larger dataset to extend our earlier findings [10].

Predictors included in our models were patients' age and sex, and the record of presence or absence of respiratory signs and symptoms, circulatory symptoms, level of consciousness, whether the patient was being readmitted or referred to the hospital. At the hospital level, we included terms for their specific identity and malaria endemicity zone. Hospital participation in one of the four research study sub-groups was included as a covariable at the patient level to take account of possible effects occurring only after a hospital joined specific studies.

We modelled whether oxygen was prescribed at admission with an interest in whether patients with a recorded pulse oximetry value (SpO_2) <90% at admission would be started on oxygen therapy. Where the pulse oximetry value at admission was missing, based on findings from a previous CIN study, the assumption was that the clinician did not have the information [10]. The analysis therefore explored (1) whether pulse oximetry values (SpO_2 <90%) were associated with oxygen prescription and (2) in the absence of a pulse oximetry value <90%, which clinical signs are associated with the prescription of oxygen therapy.

We included the variables of interest in hierarchical multivariate logistic regression models, with a random intercept for each hospital and a random slope for the time within hospital. This model specification commonly referred to as a growth curve model, allowed the explanatory variable of time to have a different effect for each hospital. A *growth curve model* typically refers to statistical methods that allow, in our case, the estimation of inter-hospital variability in intra-hospital patterns of change over time [30]. Our approach to the growth-curve model fitting is within the multilevel modelling framework, with patients nested in hospitals nested in time points [30]. Different ways of specifying the growth-curve model using a multilevel modelling framework are explained in detail elsewhere [31]. Before embarking on the growth-curve modelling we examined for each hospital whether the 2017 strike resulted in a significant discontinuity in pulse oximetry use to check if treating time as

For peer review only - http://bmjopen.bmj.com/site/about/guidelines.xhtml

months in the CIN, a continuous variable, instead of as calendar time was justifiable given the strike caused a 12-month data gap.

Where there was missing patient-level data, we applied fully conditional specification (FCS) multivariate imputation by chained equations (MICE) [32] to generate imputed datasets under the missing at random (MAR) assumption to allow us to analyse all eligible patients. Previous studies indicate that MAR is a reasonable approach for CIN data [33]. We contrasted findings using imputation with the findings of the complete case analysis.

Results

Descriptive findings

Figure 1 depicts the study population inclusion process. Out of the 179991 paediatric admissions to CIN hospitals, 132737 (73.7%) were eligible for analysis. Most exclusions were because an admission was randomly sampled for minimum data collection (n=33074, 70% of the exclusions) or fell in the strike year (n=8787, 19% exclusions). Of the population included in the analysis, 113196/132737 (86.1%) demonstrated at least one sign indicating increased risk of hypoxemia (respiratory distress symptoms or altered consciousness, or age below one month). Given this high proportion, we report results based on data from all eligible admissions (Figure 1).

Figure 1: Flow chart of the inclusion criteria

Of the 132737 cases, 48.8% (n= 64722) had a pulse oximetry measurement taken at admission. Of the 64722 patients with pulse oximetry measured at admission, 4510 (7%) had hypoxaemia ($SpO_2 < 90\%$). Across the same period, 11428/132737 (8.6%), were documented to have oxygen prescribed at admission. Of these, only 1484/11428 (12.8%) were confirmed to be hypoxaemic using pulse oximetry, while 6633/11428 (58%) had no pulse oximetry measurement and 3311/11428 (28.9%) were documented to have a pulse oximetry value of 90% or higher at admission. Thus, 87% oxygen use was

For peer review only - http://bmjopen.bmj.com/site/about/guidelines.xhtml

not pulse oximetry guided ($SpO_2 \geq 90\%$ or unknown) and more than twice as many children prescribed oxygen had values ≥90% (3311 compared with 1484)(Figure 2).

Figure 2: Oxygen prescription and use of pulse oximetry at admission

From February 2014 to December 2016, on average, there was a steady improvement in pulse oximetry adoption at admission in the 13 hospitals from between 10%-20% in 2014 to between 50%-70% at the end of 2016 (Figure 3), even though no pulse oximetry devices were provided through CIN. Following the strikes in 2017, from January 2018 when services resumed up until November 2018, average pulse oximetry adoption in these 13 sites seemed to stabilise at around 50%. By December 2018, one hospital exited and 5 hospitals joined CIN and different research studies were initiated in subsets of hospitals (Table 1, Figure 3 and Supplementary Table 2). Pulse oximetry adoption ranged from an average of 40% to almost 100% across these subsets (Figure 3).

Figure 3: Average pulse oximetry use in the CIN hospitals over time. The number of hospitals in each study covers both presently involved and those joining the CIN later: There are 13 hospitals in the period between Feb 2014 – November 2018, with the lower panel representing transition to 18 hospitals by adding 5 hospitals and research studies beginning. The 5 new hospitals' distribution among the studies in the following way: RTS,S: 2/5; RTS,S + SEARCH: 1/5; SEARCH: 1/5; None: 1/5.

Exploring each hospital's adoption trajectory illustrates in many a general improvement in adoption throughout their participation in CIN but also examples of adoption failure (Figure 4). Figure 4 tends to confirm as depicted in Figure 3, that despite a strike of almost one year there was minimal evidence of discontinuity in hospitals' pattern of adoption from 2016 to 2018. The lack of evidence for discontinuity (supported by additional analyses in Supplementary Table 3) supports our treatment of

For peer review only - http://bmjopen.bmj.com/site/about/guidelines.xhtml

time as ‘months in CIN’ as a continuous variable in place of calendar time in Figure 3 and 4 and subsequent models.

From Figure 4, 8 of the 13 hospitals recruited into the CIN by February 2014 had no use of pulse oximetry at admission at all (median at baseline 0%), with the highest baseline use being 65% (H13). For the hospitals joining after the strike (i.e., from November 2018), use of pulse oximetry during admission was generally low for 4/5 hospitals (median at baseline 25%) but one hospital already used pulse oximetry on 83% admissions (H16) (Figure 4). Hospitals demonstrated various patterns of adoption: i) low adoption when joining the CIN with a sharp improvement later (e.g. Hospital H2, H12), ii) oscillation between low and high pulse oximetry use (e.g. Hospital H3, H10, H17), iii) gradual improvement that later stabilised (e.g. Hospital H1, H4, H14), and iv) failure of adoption (H7 and H5)(Figure 4). These varying patterns of adoption, especially after December 2018, do not clearly seem to be related to study participation. Hospitals participating in both new studies, resulting in presence of two additional non-physician clinician research team members, did demonstrate a consistent improvement in pulse oximetry use at admission (Figure 3 and Figure 4). However, pulse oximetry adoption at admission was also high in hospitals not participating in any study within the CIN (Figure 3 and Figure 4).

Figure 4: Hospital specific rate of pulse oximetry use at admission over time divided into sites joining from Jan 2014 and those recruited from Dec 2018. Vertical red-line indicates national strike disruption. The title is colour-coded to reflect the research study the hospital was recruited into [Blue: None, Orange: RTS,S, Red: SEARCH, Green: RTS,S+SEARCH]. H7 exited from CIN and is therefore not included in subsequent analysis.

Individual patient data and exploratory modelling

Table 2 provides the characteristics of the children admitted to these hospitals, including the level of missingness in each variable. From the study population, 32% had at least one missing variable, with the median number of variables missing per patient of 2 of 30 (IQR: 0 - 3). None of the individual

For peer review only

variables was missing more than 30% of values. In this paediatric admission population, the prevalence of chest indrawing, tachypnoea, and grunting is 22.4%, 33% and 10.3% respectively and inability to drink (or breastfeed), a danger sign requiring urgent attention, was present in 72.7% of the admissions; 82.9% of all the admissions had at least one of these signs. The median length of stay was 3 days (IQR: 2 - 6), with a median of one additional comorbid condition at admission (IQR: 0 – 1). Pneumonia and Malaria were the leading, often comorbid, admission diagnoses (Supplementary Figure 1).

For peer review only

Table 2: Descriptive summary statistics for the variables of the patient population included in the study

Indicator	Levels	Number, n (%)	Missing data, n (%)
Hospital level factors			
Malaria endemicity zone	High	53177 (40.1%)	
	Low	79560 (59.9%)	
Patient-level factors			
Age*	< 1 month	3321 (2.5%)	1221 (0.9%)
	1-11 months	38235 (28.8%)	
	12-59 months	64628 (48.7%)	
	>= 5 years	25332 (19.1%)	
Female	Present	58588 (44.1%)	1199 (0.9%)
Case is readmission	Present	13557 (10.2%)	37002 (27.9%)
Case is referral to hospital	Present	19525 (14.7%)	39879 (30%)
Presence of comorbidity	Present	82918 (62.5%)	725 (0.5%)
AVPU** = A	Present	113378 (85.4%)	10664 (8%)
Slow capillary refill	Present	2231 (1.7%)	21902 (16.5%)
Pallor	None	95612 (72%)	10799 (8.1%)
	Mild/Moderate	15596 (11.7%)	
	Severe	10730 (8.1%)	
Fever	Present	105268 (79.3%)	6952 (5.2%)
Cough	Present	64819 (48.8%)	9237 (7%)
Crackles	Present	22835 (17.2%)	11695 (8.8%)
Central cyanosis	Present	974 (0.7%)	9758 (7.4%)
Acidotic breathing	Present	3098 (2.3%)	12740 (9.6%)
Difficulty breathing	Present	38825 (29.2%)	10454 (7.9%)
Grunting	Present	13701 (10.3%)	12255 (9.2%)
Tachypnoea***	Present	43832 (33%)	31136 (23.5%)
Indrawing	Present	29698 (22.4%)	11419 (8.6%)
Wheezing	Present	7085 (5.3%)	11034 (8.3%)
Stridor	Present	2719 (2%)	14121 (10.6%)
Inability to drink	Present	96455 (72.7%)	14846 (11.2%)

For peer review only - http://bmjopen.bmj.com/site/about/guidelines.xhtml

Table 2: Descriptive summary statistics for the variables of the patient population included in the study			
**Indicator**	**Levels**	**Number, n (%)**	**Missing data, n (%)**
Weight-for-Age Z-score	> -2SD (Normal)	74035 (55.8%)	33486 (25.2%)
	-2SD to -3SD (Low)	12377 (9.3%)	
	< -3SD (Very low)	12839 (9.7%)	
Pulse oximetry done at admission****	Present	64722 (48.8%)	
Oxygen therapy prescribed at admission	Present	11428 (8.6%)	4275 (3.2%)
Outcome at discharge	Alive	123845 (93.3%)	499 (0.4%)
	Died	8393 (6.3%)	

Note:
* < 1 month represents neonates admitted to paediatric ward; no data from the new-born units were included.
** AVPU scale is an acronym for "Alert, Verbal, Pain, Unresponsive" and is a measure of a patient's level of consciousness ordered by severity from left to right, with "A" being the least severe.
*** Symptom is derived and calculated as present where either (1) respiratory rate > 59 and age < 1 month, (2) respiratory rate > 49 and age is 1-11 months, or (3) respiratory rate is > 39 and age is 12 months or older.
**** If missing, it was assumed that it was not done. 5525 admission cases reported having done it but did not have the SpO_2 value on the paper record.

Stridor, wheezing, acidotic breathing and cyanosis have relatively low recorded prevalence of 2%, 5.3%, 2.3% and 0.7% respectively and were not included in our previous models [10]. Whether a child is a readmission or referral from another health facility are also newly added data with higher prevalence at 10.2% and 14.7% of the patient population respectively [10] (Table 2).

Summary of the growth curve model results

Table 3 provides the results of the growth curve models, where adoption rates between hospitals were adjusted for malaria endemicity, participation in research studies, participants' demographic characteristics and clinical signs and symptoms at admission. These results are from analysis using 35 imputed datasets (32% of the cohort had at least one missing variable).

Table 3: Growth curve model of pulse oximetry adoption and orders for oxygen use at admission						
	**Pulse Oximetry Done?**			**Oxygen Prescribed?**		
**Predictors**	**OR**	**95% CI**	**p-value**	**OR**	**95% CI**	**p-value**
(Intercept) ¥	0.03	0.01 - 0.17	<0.001	0.01	0.01 - 0.02	<0.001
Hospital factors						

For peer review only

For peer review only - http://bmjopen.bmj.com/site/about/guidelines.xhtml

Table 3: Growth curve model of pulse oximetry adoption and orders for oxygen use at admission						
	Pulse Oximetry Done?			Oxygen Prescribed?		
Malaria Endemicity Zone: High (Ref: Low)	1.62	0.1 - 25.04	0.729	1.55	0.9 - 2.67	0.112
Time (Months in CIN)	1.11	1.05 - 1.18	<0.001	1.02	1 - 1.03	0.045
Patient-level factors						
Ref: Patient in any study [No] ǂ						
Patient in any study [Pre-study period]ǂǂ	2.79	2.58 - 3.02	<0.001	1.02	0.9 - 1.15	0.800
Patient in any study [Yes]	1.46	1.35 - 1.58	<0.001	1.02	0.91 - 1.15	0.715
Referral: Yes (Ref: No)	1.05	1.01 - 1.09	0.025	1.19	1.12 - 1.26	<0.001
Readmission: Yes (Ref: No)	1.01	0.97 - 1.05	0.732	1.07	1 - 1.15	0.052
Is a weekend admission: Yes (Ref: No)	0.98	0.95 - 1.01	0.144	1.07	1.02 - 1.13	0.009
PAR used: Yes (Ref: No)	1.99	1.79 - 2.21	<0.001	1.23	1.02 - 1.48	0.031
PAR used * Time (Months in CIN)	1.01	1 - 1.01	<0.001	1	1 - 1.01	0.562
Ref: Age [> 59 months]						
Age [< 1 month]	0.46	0.41 - 0.51	<0.001	1.96	1.62 - 2.35	<0.001
Age [1-11 months]	1.08	1.03 - 1.13	<0.001	1.43	1.32 - 1.54	<0.001
Age [12-59 months]	1.09	1.05 - 1.13	<0.001	1.08	1 - 1.16	0.054
Female: Yes (Ref: No)	1	0.97 - 1.02	0.723	1.02	0.97 - 1.06	0.424
Fever: Yes (Ref: No)	1.18	1.14 - 1.23	<0.001	0.78	0.73 - 0.83	<0.001
Cough: Yes (Ref: No)	1.04	1.01 - 1.08	0.008	1.36	1.29 - 1.44	<0.001
Difficulty Breathing: Yes (Ref: No)	1.06	1.02 - 1.1	0.002	2.79	2.64 - 2.94	<0.001
Stridor: Yes (Ref: No)	1.05	0.95 - 1.16	0.325	1.14	1.02 - 1.27	0.017
Cyanosis: Yes (Ref: No)	0.77	0.65 - 0.92	0.004	1.5	1.27 - 1.77	<0.001
Indrawing: Yes (Ref: No)	1.25	1.2 - 1.31	<0.001	2.36	2.23 - 2.49	<0.001
Grunting: Yes (Ref: No)	0.88	0.84 - 0.93	<0.001	1.9	1.8 - 2	<0.001
Acidotic Breathing: Yes (Ref: No)	0.89	0.8 - 0.98	0.018	1.34	1.22 - 1.47	<0.001
Wheezing: Yes (Ref: No)	1.05	0.98 - 1.12	0.167	1.13	1.06 - 1.21	0.001
Crackles: Yes (Ref: No)	1.06	1.01 - 1.11	0.010	1.25	1.19 - 1.32	<0.001
Tachypnoea: Yes (Ref: No)	0.96	0.93 - 0.99	0.022	1.59	1.51 - 1.68	<0.001
Alert (AVPU < A): No (Ref: Yes)	0.96	0.9 - 1.03	0.238	1.95	1.81 - 2.1	<0.001
Inability to drink: Yes (Ref: No)	1.04	1 - 1.08	0.043	0.8	0.76 - 0.85	<0.001
Ref: Pallor [None]						
Pallor [Mild/Moderate]	0.93	0.89 - 0.97	0.002	1.1	1.02 - 1.17	0.008
Pallor [Severe]	0.94	0.89 - 0.99	0.024	1.3	1.19 - 1.42	<0.001

For peer review only - http://bmjopen.bmj.com/site/about/guidelines.xhtml

Table 3: Growth curve model of pulse oximetry adoption and orders for oxygen use at admission

	**Pulse Oximetry Done?**			**Oxygen Prescribed?**		
Ref: WAZ [Normal]						
WAZ [Low]	0.97	0.92 - 1.01	0.135	0.98	0.91 - 1.05	0.551
WAZ [Very Low]	0.94	0.9 - 0.99	0.020	0.93	0.87 - 1	0.059
Slow Capillary Refill: Yes (Ref: No)	0.88	0.78 - 1	0.051	0.97	0.83 - 1.13	0.693
Ref: Hypoxaemia [Unknown]						
Hypoxaemia [No]				0.59	0.56 - 0.62	<0.001
Hypoxaemia [Yes]				1.92	1.77 - 2.08	<0.001

Note:

‡"*Patient is in any study*" point estimates and confidence intervals for the *"pulse oximetry done?"* outcome do not overlap with ones from complete case analysis (Supplementary Table 4). From the complete case sensitivity analysis, there is a material difference in the estimated association of involvement in any research study variable with the pulse oximetry use at admission outcome (Table 3, Supplementary Table 4); While the direction of association is the same (positive), the confidence intervals do not overlap. With the age variable, the direction of the association is inverted between the complete case and multiple imputation analyses, and children aged < 1 month admitted to the paediatric ward are completely missing in the complete case analysis. These material difference might be attributable to the fact that complete case analysis and our multiple imputation approach make different assumptions of missingness mechanism in the data (i.e., Missing Completely At Random (MCAR) mechanism -which is potentially more biased, and a MAR mechanism respectively).

‡‡"*Patient is in any study[Pre-study period]*" reflects the time leading up to any research study starting when no study was being conducted i.e. February 2014 – November 2018.

¥Represents the average odds that a patient will have pulse oximetry is done (or oxygen therapy is prescribed) at admission in this sample when all the predictors are set to their reference levels (e.g., "No") at the first month of being a member of the CIN

For peer review only

Associations of the hospital and clinical features with the outcomes from Table 3 are time-invariant (i.e. independent of the effect of time in months in CIN), except for the interaction term of PAR use with time. From Supplementary Table 4 and Figure 4, the models' high Intraclass Correlation Coefficient (ICC) is indicative that hospital factors play a bigger role in the adoption of pulse oximetry over time compared to individual patient-level factors. The contribution of hospital level factors is less pronounced for oxygen prescription at admission. The relatively high ICC is not unexpected in

For peer review only - http://bmjopen.bmj.com/site/about/guidelines.xhtml

longitudinal models with measurements clustered by hospital and given the varying patterns of adoption seen in Figure 4.

Association with the use of pulse oximetry at admission

Over the entire study period and across all hospitals pulse oximetry use at admission improved as time participating in the CIN increased (month-to-month improvement OR: 1.11, 95% CI: 1.05 – 1.18). This was likely driven by noticeable improvement in 10 hospitals and in 5 of these specifically there were substantive positive changes in adoption of pulse oximetry on a month-to-month basis; (Supplementary Figure 2). Those improving most tended to have very low use of oximetry at the time they joined the CIN (Supplementary Figure 2). This improvement was seen in tandem with the improvement in PAR use which might be expected to encourage better assessment and potentially, therefore, aid clinical decision making.

From Table 3, patients admitted with signs of fever, cough, difficulty breathing, chest indrawing, crackles, and inability to drink had odds statistically greater than one of having pulse oximetry measurement taken at admission over time than those without these signs. With a larger dataset, the inclusion of new variables and adjusting for the time in the CIN, our findings conflict with previous findings on the significance of tachypnoea, altered consciousness (AVPU < A), and inability to drink as signs associated with the use of pulse oximetry at admission with a reversal in association (e.g. Tachypnoea, Inability to drink) or no statistically appreciable association (e.g. AVPU <A)[10]. Grunting and acidotic breathing symptoms, likely to be associated with malaria and anaemia (Supplementary Figure 1), were statistically, not significantly associated with pulse oximetry use at admission (Table 3). Cyanosis, grunting, and acidotic breathing –which were previously not reported [10]- were significantly associated with the odds of pulse oximetry done at admission being lower than one. Our findings also highlight that referral into the hospital is positively associated with the use of pulse oximeters at admission (OR: 1.05, 95% CI: 1.01 - 1.09)(Table 3) and that patients admitted to hospitals that were part of an active research study were 49% more likely to have a pulse oximetry measurement taken at admission than in hospitals not participating in any study (Table 3). Additional

For peer review only

analyses suggest the more clinical signs a child has of respiratory distress the more likely they were to be hypoxemic (Supplementary Figure 3).

Associations with oxygen prescription at admission

Admission with any sign of respiratory distress where the pulse oximetry measure is unknown is associated with oxygen prescription at admission (Table 3). The specific signs that appear to prompt oxygen prescription, in reducing magnitude of association, include difficulty breathing, indrawing, AVPU <A, grunting, and tachypnoea. Admissions with altered consciousness (AVPU < A) had almost twice the odds of oxygen prescription at admission, independent of respiratory signs (Table 3). Interestingly, the direction of association with oxygen prescription is inverse to that for use of pulse oximetry for: age of the child, signs of cyanosis, grunting, acidotic breathing, AVPU <A, pallor, tachypnoea, fever and inability to drink.

With the inclusion of new variables and adjusting for the time in CIN, our findings affirm previous analyses but show the magnitude of association of cough and difficulty breathing with oxygen prescription at admission to be higher than previously reported [10]. Acidotic breathing, stridor and wheezing, clinical signs omitted from the previous study, were shown to be statistically significant and positively associated with the odds of being prescribed oxygen at admission. Grunting and acidotic breathing, likely to be associated with Malaria and Anaemia diagnoses, were not statistically associated with having pulse oximetry measurement taken at admission but were strongly associated with the prescription of oxygen at admission (Table 3).

Our findings also highlight that being a patient referred into the hospital (OR: 1.19, 95% CI: 1.12 - 1.26) or admitted during the weekend (OR: 1.07, 95% CI: 1.02 - 1.13) is positively associated with oxygen prescription at admission (Table 3). Oxygen prescription at admission increased with time as part of CIN, having a statistically significant but clinically small month-to-month change (OR: 1.02, 95% CI: 1.00 – 1.03). The improvement in the PAR use (OR: 1.23, 95% CI: 1.02 - 1.48) also appears to be aiding

For peer review only

For peer review only - http://bmjopen.bmj.com/site/about/guidelines.xhtml

clinical decision making in the prescription of oxygen based on the recording of features of respiratory distress.

Discussion

Over a period of 7 years, with prolonged strikes precluding data collection in 2017, use of pulse oximetry in a population of admissions where 86% have at least one feature indicating risk of hypoxemia increased slowly with each month of participation in CIN. Most hospitals at the start of their participation in CIN used pulse oximetry in 25% or fewer children, increasing to 81.5% in all hospitals by the final quarter of 2020. Amongst the admissions prescribed oxygen in 87% this was not apparently guided by a pulse oximetry value (SpO2) of < 90%, the recommended level for intervention to address hypoxemia in current pneumonia guidelines.

The highly variable patterns of pulse oximetry adoption across hospitals (Figure 4) and high intraclass correlation coefficients in our models point to the major impact of hospital-level factors on the use of pulse oximetry. These have been previously described and include equipment supply, maintenance, local leadership and clinical efforts to train and promote oximetry use amongst others [10]. CIN provided regular feedback to hospitals including on pulse oximetry use and SpO_2 values throughout a hospital's participation in CIN and engaged subsets of hospitals in research projects that resulted in the placement of one or two additional non-physician clinicians (i.e., clinical officers, nurses) in some hospitals. In the absence of data from other settings it is hard to determine if the increase in pulse oximetry use is part of a wider secular trend (a phenomenon referred to as the "rising tide" [34]) or associated with CIN participation. Whatever the reasons, it is instructive that adoption of pulse oximetry remains incomplete after 7 years even amongst inpatient populations with severe illness in moderately sized hospitals. This slow pattern of adoption for an established technology that is widely believed to be beneficial has been observed in high-income settings. It suggests we may only realise the proposed benefits of many technologies including diagnostics much more slowly than is often

For peer review only - http://bmjopen.bmj.com/site/about/guidelines.xhtml

claimed unless much greater attention is paid to strengthening health service delivery at scale [35, 36].

Overall, approximately 9% of patients were prescribed oxygen at admission, with a slight increase in patients with oxygen prescribed with each month in the CIN. Interestingly, in our models the intraclass correlation coefficients exploring oxygen use did not suggest strong hospital-level effects, placing more emphasis on patient-level factors. The number of patients with oxygen prescribed who had no SpO_2 recorded is large; more than twice as many children receiving oxygen had pulse oximetry values 90% or more than values < 90% (Figure 2) although WHO and Kenyan guidelines recommend targeting oxygen use at children with SpO2 < 90% [1]. While the source of discrepancy remains largely unknown, it is unlikely that this reflects diagnoses of shock or severe asthma when oxygen use can be recommended when saturations are higher than 90% as these diagnoses are relatively uncommon in Kenyan hospitals [1, 37].

We attempt to integrate our new findings of patient-level factors associated with the use of pulse oximetry and prescription of oxygen at admission with earlier findings that addressed mainly hospital-level factors in Figure 5. Lower chest-wall indrawing is strongly and positively associated with pulse oximetry being done at admission and prescription of oxygen when pulse oximetry is not done. We have previously shown an association between indrawing and mortality for pneumonia admissions and others have recently indicated indrawing may be a useful referral criterion from the community or primary care to a hospital in the absence of pulse oximetry [5, 38]. However, randomised trials, and WHO guidance, suggest pneumonia associated with indrawing can be safely treated as an outpatient [2, 37]. Our data does not directly inform the debate on the value of indrawing as screening sign of illness severity but does perhaps suggest it remains an important factor in Kenyan clinicians' decision-making.

Other signs prompting pulse oximeter use are fever, cough, difficulty breathing, crackles and inability to drink (Table 3, Figure 5). All respiratory signs are associated with oxygen prescription (Table 3,

For peer review only

For peer review only - http://bmjopen.bmj.com/site/about/guidelines.xhtml

Supplementary Table 5, Figure 5) and a pulse oximetry value <90% was associated with twice the odds of oxygen prescription compared with those whose oxygen saturation was unknown. Where information from pulse oximetry was not available cyanosis, grunting, acidotic breathing, AVPU <A, pallor, and tachypnoea appeared to prompt oxygen use (Table 3, Figure 5). Several of these signs were not associated with the use of pulse oximetry at admission, perhaps because healthcare providers are taught and then view these signs as indicative of very severe illness (as reported in the literature) requiring intervention with oxygen irrespective of oxygen saturation [1, 37, 38]. Interestingly, over time, the use of the standardised paediatric admission record (PAR) was associated with twice the odds of a patient having SpO_2 measurement and 23% increase in oxygen therapy prescribed at admission, where indicated (Table 3). It is possible this effect reflects its role as a prompt to conduct a complete assessment and promote reflection on the use of core interventions recommended in guidelines.

Why infants less than one month old have oximetry measured so infrequently remains unknown and warrants more investigation (Table 3). It is highly likely that the paediatric wards may not have appropriately sized pulse oximetry probes for the neonates and/or that many of the neonate admissions to the paediatric ward have a diagnosis of sepsis or jaundice which have less clear guidelines on performing routine pulse oximetry on all admissions. Overall, neonates with respiratory problems who mostly tend to be aged closer to time of birth, are sent to newborn wards and thus not meant to be captured in this paediatric admissions dataset (Table 2).

It has been well described in many settings that the logical world represented in clinical guidelines is often not replicated in the more complex world of everyday practice [39-41]. While there remain debates in the scientific world around which oxygen saturation threshold for which condition in which setting should guide the use of oxygen [5, 42, 43], our data remind us that clinicians are often influenced by many other factors in making such decisions [44]. Clinical decision making is not straightforward, and it is difficult to capture from routinely collected observational data. Reducing the decision of whether to prescribe or withhold oxygen to a pulse oximetry reading might inadequately

For peer review only

For peer review only - http://bmjopen.bmj.com/site/about/guidelines.xhtml

431 reflect the complexities that a clinician encounters at the patient bedside. This has important
432 implications as the presence or prevalence of hypoxemia, however defined, may be used as proxies
433 for rational use or need for oxygen respectively. Clinicians' decision to deviate from pulse oximetry
434 defined hypoxemia thresholds in determining oxygen use, and the subsequent effects this would have
435 on resource use or patient outcomes, remains largely unaddressed in research [2].

436 **Figure 5: Adapted Integrative Model of Behavioural Prediction (Enoch et al. [10]). Dashes indicate components**
437 **of the model that are modified or added to reflect the findings from our new analyses. HCW: healthcare**
438 **worker.**

439

440 Strengths and limitations

441 The data we present are a rare example of long-term tracking of hospital practice in a routine LMIC
442 setting covering common childhood illnesses (Supplementary Table 6). The longitudinal nature of our
443 observations offers important insights but also poses challenges. Major changes may occur in the
444 health sector over long periods, as exemplified by the prolonged strikes in Kenya. Many factors ranging
445 from national and local procurement or equipment donations, to changes in local leadership and
446 hospital's participation in specific studies might influence the adoption of pulse oximetry and use of
447 oxygen we describe. Furthermore, we have no contemporaneous data from Kenyan settings that do
448 not belong to the CIN for comparison and most other studies exploring the use of pulse oximetry are
449 essentially cross-sectional. We do, however, feel our data are at least a useful illustration of the slow
450 and sometimes erratic process of pulse oximetry adoption in LMIC hospitals that has broader
451 implications for thinking on technology adoption.

452 Another limitation of this study is the absence of new qualitative data especially exploring why oxygen
453 use is often not linked to pulse oximetry values. However, we have previously shown the importance
454 of local clinical leadership in these settings [45] and the continuing challenge of promoting guideline
455 adherence in these complex hospital settings where junior clinicians who manage patients rotate

For peer review only - http://bmjopen.bmj.com/site/about/guidelines.xhtml

frequently and may err on the side of caution to use interventions rather than risk withholding them [46-48].

Further challenges of the large-scale, longitudinal nature of our work are data quality and analytic methods. The CIN has made efforts to capture high-quality data through continuous training of clerks linked to error checking procedures and regular engagement with hospitals that include monitoring and feedback [18, 28, 29]. Nonetheless, imputation procedures that assume a missing at random mechanism had to be employed to enable analyses of all eligible case records [32, 49]. We also had to employ a complex modelling framework to explore patient-level associations with our outcomes. These attempted to accommodate and account for between hospital variability, within hospital non-linear trends in the outcomes over time and entry into the CIN at different time points. We tested our assumptions where possible (e.g. that the strike year was not associated with major changes in practice). However, while our large dataset enabled us to identify many credible associations specific findings should be interpreted cautiously.

Conclusions

Over time, there has been a progressive increase in the use of pulse oximeters at admission in the Kenyan hospitals we studied. After seven years, however, use in this high mortality population where almost 9 out of 10 children have least one sign indicating increased risk of hypoxemia is not universal and major differences exist between hospitals in the adoption of this technology. Guidelines suggest pulse oximetry should determine which children receive oxygen. Our data suggest this is often not the case even in late 2020 during the COVID-19 pandemic, and that many organisational and clinical factors influence whether oximetry is used at all or whether recommended hypoxemia definitions are used to guide the use of oxygen. As global efforts gain pace to provide pulse oximeters and oxygen systems our data show it is perhaps naïve to expect they will be adopted and employed in the rational ways imagined in guidelines and policies.

For peer review only - http://bmjopen.bmj.com/site/about/guidelines.xhtml

Appendix

CRediT author statement

Timothy Tuti: Conceptualization, Methodology, Software, Data curation, Visualization, Writing- Original draft, Writing - Reviewing and Editing. **Jalemba Aluvaala**: Methodology, Investigation, Writing- Reviewing and Editing. **Samuel Akech**: Methodology, Investigation, Writing- Reviewing and Editing. **Ambrose Agweyu**: Methodology, Investigation, Writing- Reviewing and Editing. **Grace Irimu**: Writing- Reviewing and Editing **Mike English**: Conceptualization, Methodology, Supervision, Investigation, Writing- Reviewing and Editing.

Ethics approval and consent to participate

This CIN study was approved by the Kenya Medical Research Institute's (KEMRI) Scientific and Ethics Review Unit (#SERU 3459). Additionally, it was approved by the Ministry of Health, with the Medical Superintendents of participant hospitals given consent for participation. Individual consent for access to de-identified patient data was not required.

Consent to publish

This paper is published with the permission of the Director of KEMRI.

Acknowledgements

The Clinical Information Network Author Group: The CIN author group who contributed to the network's development, data collection, data management, implementation of audit and feedback and who reviewed and approved this publication include: 1) Central CIN team members: Lynda Isaaka, George Mbevi, Cynthia Khaenzi, Paul Mwaniki, Mercy Chepkirui, John Wainaina, Livingstone Mumelo, Edith Gicheha, Monica Musa, Naomi Muinga and Muthoni Ogola. 2) National partners: Laura Oyiengo, Caroline Mwangi, Fred Were, and (3) Hospital teams: **(Paediatricians)** Juma Vitalis, Nyumbile Bonface, Roselyne Malangachi, Christine Manyasi, Catherine Mutinda, David Kibiwott Kimutai, Rukia Aden, Caren Emadau, Elizabeth Atieno Jowi, Cecilia Muithya, Charles Nzioki, Supa Tunje, Dr. Penina Musyoka, Wagura Mwangi, Agnes Mithamo, Magdalene Kuria, Esther Njiru, Mwangi Ngina, Penina Mwangi, Rachel Inginia, Melab Musabi, Emma Namulala, Grace Ochieng, Lydia Thuranira, Felicitas Makokha,

For peer review only

Josephine Ojigo, Beth Maina, Catherine Mutinda, Mary Waiyego, Bernadette Lusweti, Angeline Ithondeka, Julie Barasa, Meshack Liru, Elizabeth Kibaru, Alice Nkirote Nyaribari, Joyce Akuka, Joyce Wangari;

(Nurses) Amilia Ngoda, Aggrey Nzavaye Emenwa, Dolphine Mochache, Patricia Nafula Wesakania, George Lipesa, Jane Mbungu, Marystella Mutenyo, Joyce Mbogho, Joan Baswetty, Ann Jambi, Josephine Aritho, Beatrice Njambi, Felisters Mucheke, Zainab Kioni, Jeniffer, Lucy Kinyua, Margaret Kethi, Alice Oguda, Salome Nashimiyu Situma, Nancy Gachaja, Loise N. Mwangi, Ruth Mwai, irginia Wangari Muruga, Nancy Mburu, Celestine Muteshi, Abigael Bwire, Salome Okisa Muyale, Naomi Situma, Faith Mueni, Hellen Mwaura, Rosemary Mututa, Caroline Lavu, Joyce Oketch, Jane Hore Olum, Orina Nyakina, Faith Njeru, Rebecca Chelimo, Margaret Wanjiku Mwaura, Ann Wambugu, Epharus Njeri Mburu, Linda Awino Tindi, Jane Akumu, Ruth Otieno, Slessor Osok;

(HRIOs) Seline Kulubi, Susan Wanjala, Pauline Njeru, Rebbecca Mukami Mbogo, John Ollongo, Samuel Soita, Judith Mirenja, Mary Nguri, Margaret Waweru, Mary Akoth Oruko, Jeska Kuya, Caroline Muthuri, Esther Muthiani, Esther Mwangi, Joseph Nganga, Benjamin Tanui, Alfred Wanjau, Judith Onsongo, Peter Muigai, Arnest Namayi, Elizabeth Kosiom, Dorcas Cherop, Faith Marete, Johanness Simiyu, Collince Danga, Arthur Otieno Oyugi, Fredrick Keya Okoth.

Data Availability

Patient-level data used in this report are under the primary jurisdiction of the Ministry of Health in Kenya. The authors had full access to all the quantitative data in the study and take responsibility for the integrity of the data and the accuracy of the data analysis. Researchers can contact the KEMRI Wellcome Trust Research Programme Data Governance Committee (dgc@kemri-wellcome.org) for advice on accessing the data including whether a new ethical review submission and approval from the Ministry of Health in Kenya would be required.

Funding

The work described was supported by multiple funders. These include the Wellcome Trust (#207522) through an award to ME as a Senior Fellowship and a core grant awarded to the KEMRI-Wellcome

For peer review only - http://bmjopen.bmj.com/site/about/guidelines.xhtml

Trust Research Programme (#092654); by a grant from World Health Organization: An evaluation in Kenya of the cluster-randomized RTS,S/AS01 malaria vaccine implementation pilot through routine health systems in moderate to high malaria transmission settings (WHO-Project requisition number 2018/854999); by a grant to the NEST program from the John D. and Catherine T. MacArthur Foundation, the Bill & Melinda Gates Foundation, ELMA Philanthropies, and The Children's Investment Fund Foundation UK under agreements to William Marsh Rice University with a sub-agreement to University of Oxford Centre for Tropical Medicine and Global Health; and an award to AA (MR/R006083/1) from the Foreign, Commonwealth and Development Office (FCDO), the MRC, the National Institute for Health Research (NIHR) and Wellcome Trust Joint Global Health Trials Scheme. For the purpose of Open Access, the author has applied a CC-BY public copyright licence to any author accepted manuscript version arising from this submission.

The funders had no role in the preparation of this report or the decision to submit for publication.

Competing interests

We have read the journal's policy and the authors of this manuscript have no competing interests to declare

Abbreviations:

CI, confidence interval; CIN, Clinical Information Network; FCS, fully conditional specification; HCW, healthcare worker; HRIO, Hospital Records Information Officer; ICC, Intra-cluster Correlation Coefficient; KEMRI, Kenya Medical Research Institute; LMIC, low- and middle-income country; MAR, Missing At Random; MCAR, Missing Completely At Random; MICE, Multivariate Imputation by Chained Equations; MoH, Ministry of Health; OR, odds ratio; PAR, Paediatric Admission Record; SERU, KEMRI's Scientific and Ethics Review Unit; WHO, World Health Organization

References

1. World Health Organization, *Manual on use of oxygen therapy in children.* World Health Organization (WHO), 2016.

For peer review only

2. Enoch, A.J., M. English, and S. Shepperd, *Does pulse oximeter use impact health outcomes? A systematic review.* Archives of disease in childhood, 2016. **101**(8): p. 694-700.
3. Jouffroy, R., D. Jost, and B. Prunet, *Prehospital pulse oximetry: a red flag for early detection of silent hypoxemia in COVID-19 patients.* Critical Care, 2020. **24**(1): p. 1-2.
4. Starr, N., et al., *Pulse oximetry in low-resource settings during the COVID-19 pandemic.* The Lancet Global Health, 2020. **8**(9): p. e1121-e1122.
5. Colbourn, T., et al., *Predictive value of pulse oximetry for mortality in infants and children presenting to primary care with clinical pneumonia in rural Malawi: A data linkage study.* PLoS medicine, 2020. **17**(10): p. e1003300.
6. Adebola, O., O. Babatunde, and O. Bose, *Hypoxemia predicts death from severe falciparum malaria among children under 5 years of age in Nigeria: the need for pulse oximetry in case management.* African health sciences, 2014. **14**(2): p. 397-407.
7. Lozano, J., *Epidemiology of hypoxaemia in children with acute lower respiratory infection [oxygen therapy in children].* The international journal of tuberculosis and lung disease, 2001. **5**(6): p. 496-504.
8. Liu, L., et al., *Global, regional, and national causes of under-5 mortality in 2000–15: an updated systematic analysis with implications for the Sustainable Development Goals.* The Lancet, 2016. **388**(10063): p. 3027-3035.
9. Gray, A.Z., et al., *Improved oxygen systems in district hospitals in Lao PDR: a prospective field trial of the impact on outcomes for childhood pneumonia and equipment sustainability.* BMJ paediatrics open, 2017. **1**(1).
10. Enoch, A.J., et al., *Variability in the use of pulse oximeters with children in Kenyan hospitals: A mixed-methods analysis.* PLoS medicine, 2019. **16**(12): p. e1002987.
11. Graham, H., et al., *Providing oxygen to children in hospitals: a realist review.* Bulletin of the World Health Organization, 2017. **95**(4): p. 288.
12. Irimu, G., et al., *Tackling health professionals' strikes: an essential part of health system strengthening in Kenya.* BMJ global health, 2018. **3**(6).
13. García-Romero, A., Á. Escribano, and J.A. Tribó, *The impact of health research on length of stay in Spanish public hospitals.* Research Policy, 2017. **46**(3): p. 591-604.
14. Lichten, C.A., et al., *Does a biomedical research centre affect patient care in local hospitals?* Health research policy and systems, 2017. **15**(1): p. 2.
15. Gathara, D., et al., *Variation in and risk factors for paediatric inpatient all-cause mortality in a low income setting: data from an emerging clinical information network.* BMC pediatrics, 2017. **17**(1): p. 99.

For peer review only

16. Von Elm, E., et al., *The Strengthening the Reporting of Observational Studies in Epidemiology (STROBE) statement: guidelines for reporting observational studies.* Annals of internal medicine, 2007. **147**(8): p. 573-577.

17. English, M., et al., *Building learning health systems to accelerate research and improve outcomes of clinical care in low-and middle-income countries.* PLoS medicine, 2016. **13**(4): p. e1001991.

18. Irimu, G., et al., *Approaching quality improvement at scale: a learning health system approach in Kenya.* Archives of disease in childhood, 2018. **103**(11): p. 1013-1019.

19. English, M., et al., *Employing learning health system principles to advance research on severe neonatal and paediatric illness in Kenya.* BMJ Global Health, 2021. **6**(3): p. e005300.

20. English, M., et al., *Programme theory and linked intervention strategy for large-scale change to improve hospital care in a low and middle-income country-A Study Pre-Protocol.* Wellcome Open Research, 2020. **5**.

21. Ayieko, P., et al., *Characteristics of admissions and variations in the use of basic investigations, treatments and outcomes in Kenyan hospitals within a new Clinical Information Network.* Archives of disease in childhood, 2016. **101**(3): p. 223-229.

22. Ministry of Health, *Kenya Service Availability and Readiness Assessment Mapping (SARAM).* 2014, Government of Kenya Nairobi, Kenya.

23. Gachau, S., et al., *Does audit and feedback improve the adoption of recommended practices? Evidence from a longitudinal observational study of an emerging clinical network in Kenya.* BMJ global health, 2017. **2**(4): p. e000468.

24. Mwakyusa, S., et al., *Implementation of a structured paediatric admission record for district hospitals in Kenya–results of a pilot study.* BMC international health and human rights, 2006. **6**(1): p. 9.

25. English, M., et al., *Implementing locally appropriate guidelines and training to improve care of serious illness in Kenyan hospitals: a story of scaling-up (and down and left and right).* Archives of disease in childhood, 2011. **96**(3): p. 285-290.

26. Ministry of Health. *Basic Paediatric Protocols*. 2016 [cited 2020 11th Nov]; 4th Edition:[Available from: http://guidelines.health.go.ke/#/category/27/266/meta.

27. Health Services Unit KEMRI–Wellcome Trust Research Programme. *Paediatric Tools & Hospital forms 2016*. 2016 [cited 2020 21st August]; Available from: http://www.kenyapaediatric.org/idoc/index.php/en/etat/instructors/paeditric-tools-a-hospital-forms.html.

For peer review only

28. Tuti, T., et al., *Innovating to enhance clinical data management using non-commercial and open source solutions across a multi-center network supporting inpatient pediatric care and research in Kenya.* Journal of the American Medical Informatics Association, 2016. **23**(1): p. 184-192.

29. Tuti, T., et al., *Improving documentation of clinical care within a clinical information network: an essential initial step in efforts to understand and improve care in Kenyan hospitals.* BMJ global health, 2016. **1**(1): p. e000028-e000028.

30. Curran, P.J., K. Obeidat, and D. Losardo, *Twelve frequently asked questions about growth curve modeling.* Journal of cognition and development, 2010. **11**(2): p. 121-136.

31. Kristoffer, M. *Using R and lme/lmer to fit different two- and three-level longitudinal models*. 2015 [cited 2020 30th September]; Available from: https://rpsychologist.com/r-guide-longitudinal-lme-lmer.

32. Buuren, S.v. and K. Groothuis-Oudshoorn, *mice: Multivariate imputation by chained equations in R.* Journal of statistical software, 2010: p. 1-68.

33. Gachau, S., et al., *Handling missing data in modelling quality of clinician-prescribed routine care: Sensitivity analysis of departure from missing at random assumption.* Statistical Methods in Medical Research, 2020: p. 0962280220918279.

34. Chen, Y.-F., et al., *Secular trends and evaluation of complex interventions: the rising tide phenomenon.* BMJ quality & safety, 2016. **25**(5): p. 303-310.

35. Oliwa, J.N., et al., *Diagnostic practices and estimated burden of tuberculosis among children admitted to 13 government hospitals in Kenya: an analysis of two years' routine clinical data.* PloS one, 2019. **14**(9): p. e0221145.

36. Graham, H.R., et al., *Adoption of paediatric and neonatal pulse oximetry by 12 hospitals in Nigeria: a mixed-methods realist evaluation.* BMJ global health, 2018. **3**(3): p. e000812.

37. World Health Organization, *Pocket book of hospital care for children: guidelines for the management of common childhood illnesses*. 2013: World Health Organization.

38. Agweyu, A., et al., *Appropriateness of clinical severity classification of new WHO childhood pneumonia guidance: a multi-hospital, retrospective, cohort study.* The Lancet Global health, 2018. **6**(1): p. e74-e83.

39. Gabbay, J. and A. le May, *Mindlines: making sense of evidence in practice*. 2016, British Journal of General Practice.

40. Murray, E., et al., *Normalisation process theory: a framework for developing, evaluating and implementing complex interventions.* BMC medicine, 2010. **8**(1): p. 1-11.

For peer review only

For peer review only - http://bmjopen.bmj.com/site/about/guidelines.xhtml

41. Thompson, D.S., et al., *Scoping review of complexity theory in health services research.* BMC health services research, 2016. **16**(1): p. 1-16.

42. Serpa Neto, A., et al., *Pragmatic Recommendations for the Management of Acute Respiratory Failure and Mechanical Ventilation in Patients with COVID-19 in Low-and Middle-Income Countries.* The American Journal of Tropical Medicine and Hygiene, 2021: p. tpmd200796.

43. Maitland, K., et al., *Children's Oxygen Administration Strategies Trial (COAST): A randomised controlled trial of high flow versus oxygen versus control in African children with severe pneumonia.* Wellcome Open Research, 2017. **2**.

44. WHO;, lifebox;, and ALIMA;. *Using pulse oximetry for decision support for COVID-19*. 2020 11th April 2020 [cited 2020 30th October]; Available from: https://www.lifebox.org/wp-content/uploads/2020/04/Pulse-Oximetry-for-COVID-19-Decision-Tool-Lifebox-FINAL1.pdf.

45. Nzinga, J., G. McGivern, and M. English, *Examining clinical leadership in Kenyan public hospitals through the distributed leadership lens.* Health policy and planning, 2018. **33**(suppl_2): p. ii27-ii34.

46. Maina, M., et al., *Antibiotic use in Kenyan public hospitals: Prevalence, appropriateness and link to guideline availability.* International Journal of Infectious Diseases, 2020. **99**: p. 10-18.

47. McKnight, J., et al., *Evaluating hospital performance in antibiotic stewardship to guide action at national and local levels in a lower-middle income setting.* Global Health Action, 2019. **12**(sup1): p. 1761657.

48. Ogero, M., et al., *Examining which clinicians provide admission hospital care in a high mortality setting and their adherence to guidelines: an observational study in 13 hospitals.* Archives of disease in childhood, 2020. **105**(7): p. 648-654.

49. Sperrin, M. and G.P. Martin, *Multiple imputation with missing indicators as proxies for unmeasured variables: simulation study.* BMC Medical Research Methodology, 2020. **20**(1): p. 1-11.

For peer review only

For peer review only - http://bmjopen.bmj.com/site/about/guidelines.xhtml

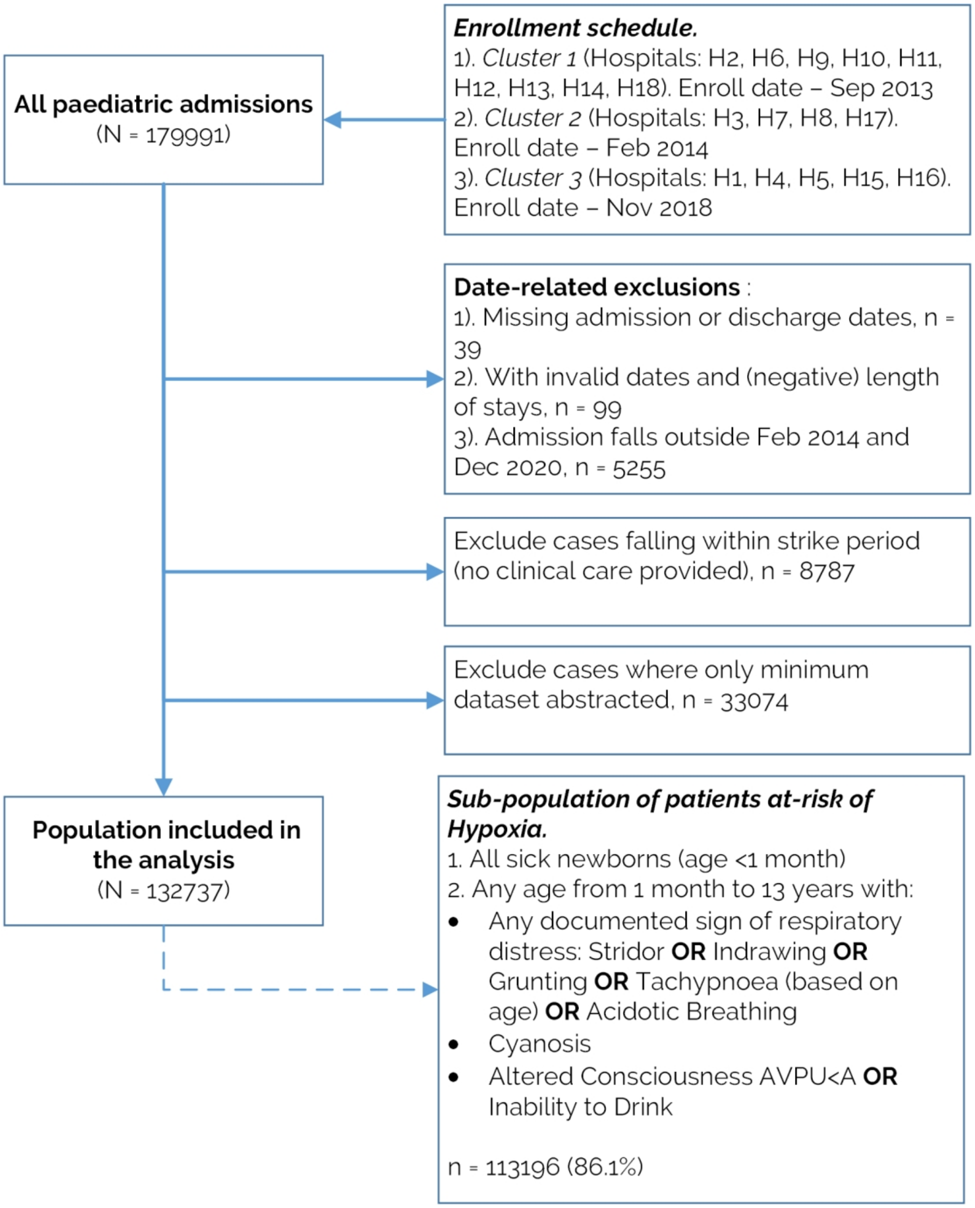

Flow chart of the inclusion criteria

146x180mm (300 x 300 DPI)

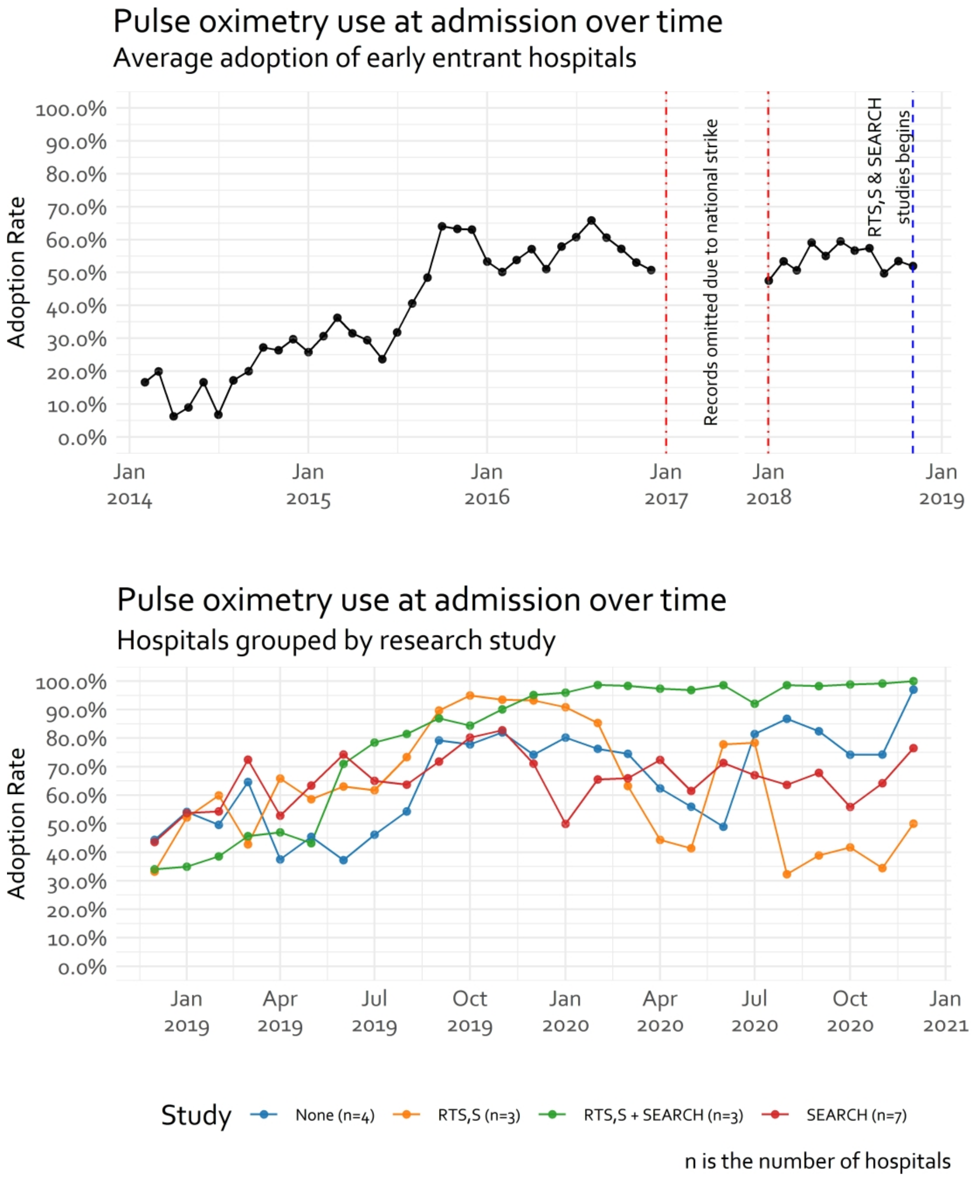

Average pulse oximetry use in the CIN hospitals over time. The number of hospitals in each study covers both presently involved and those joining the CIN later: There are 13 hospitals in the period between Feb 2014 – November 2018, with the lower panel representing transition to 18 hospitals by adding 5 hospitals and research studies beginning. The 5 new hospitals' distribution among the studies in the following way: RTS,S: 2/5; RTS,S + SEARCH: 1/5; SEARCH: 1/5; None: 1/5.

179x209mm (600 x 600 DPI)

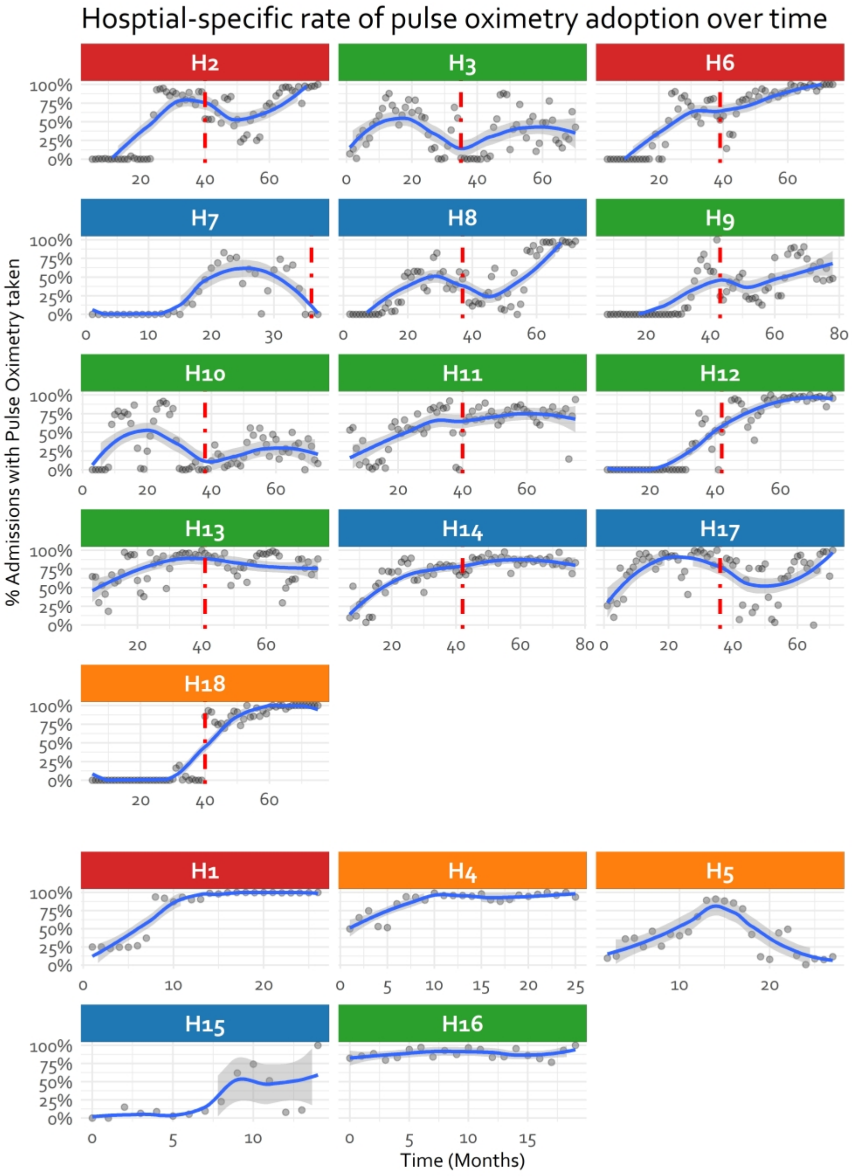

Hospital specific rate of pulse oximetry use at admission over time divided into sites joining from Jan 2014 and those recruited from Dec 2018. Vertical red-line indicates national strike disruption. The title is colour-coded to reflect the research study the hospital was recruited into [Blue: None, Orange: RTS,S, Red: SEARCH, Green: RTS,S+SEARCH]. H7 exited from CIN and is therefore not included in subsequent analysis.

179x246mm (600 x 600 DPI)

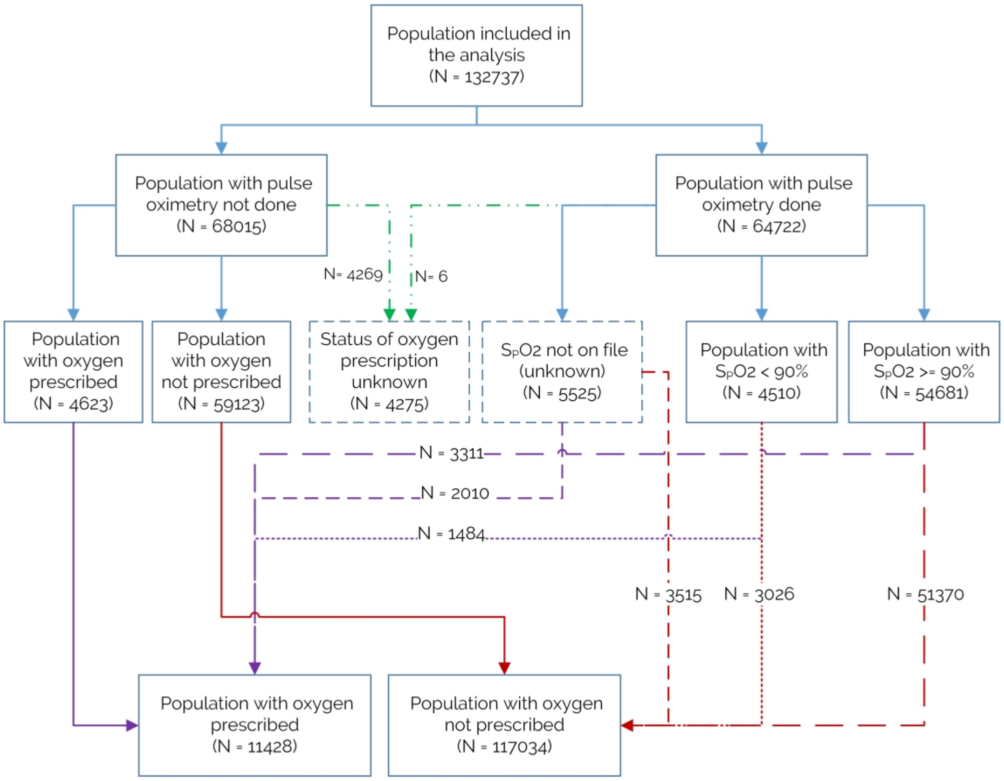

Oxygen prescription and use of pulse oximetry at admission

180x138mm (600 x 600 DPI)

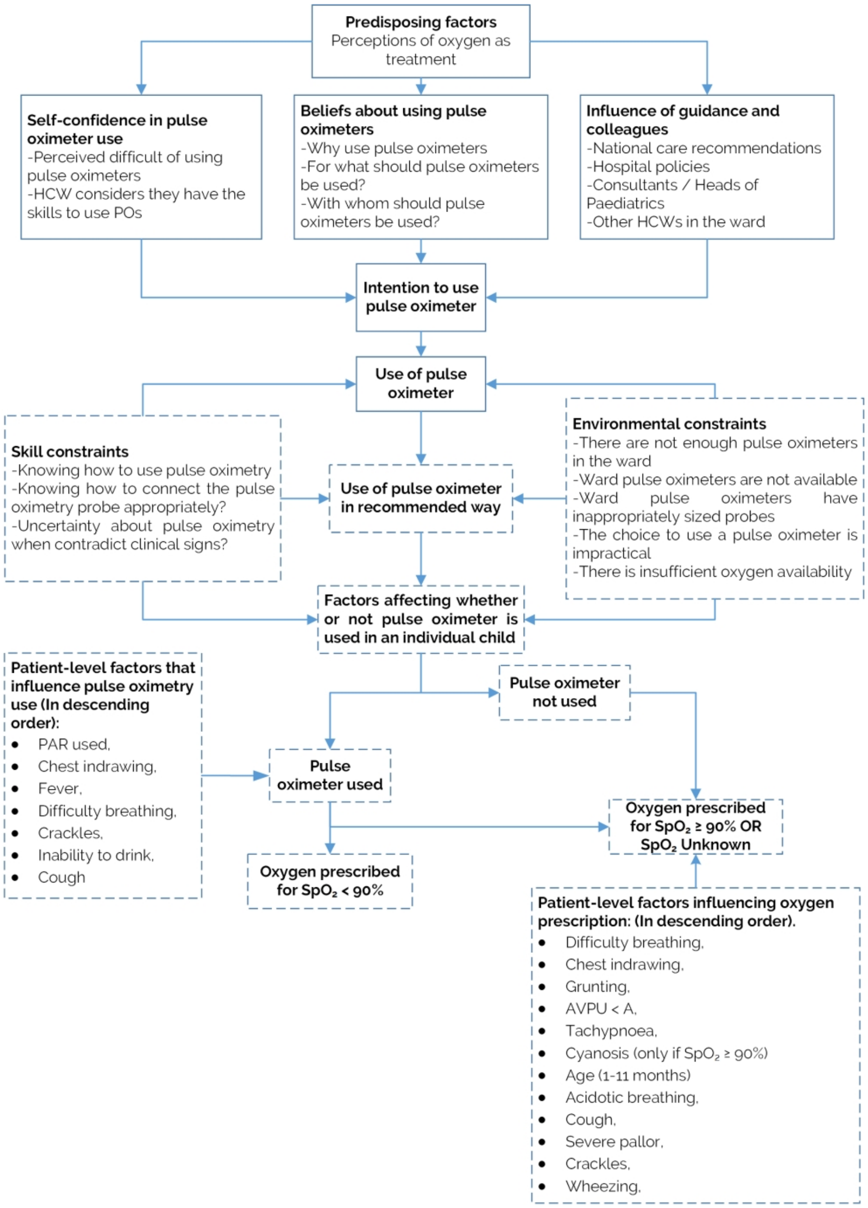

Adapted Integrative Model of Behavioural Prediction (Enoch et al. [10]). Dashes indicate components of the model that are modified or added to reflect the findings from our new analyses. HCW: healthcare worker.

180x248mm (300 x 300 DPI)

Supplementary Material

Supplementary Table 1: Number of patients included into the study per hospital

Hospital	n (%)	Pulse oximeters donated to the hospital by CIN	Current number of pulse oximeters at the paediatric ward	Current status of pulse oximeters*
**H1**	6065 (4.6%)	1	1	All functioning well
**H2**	11636 (8.8%)	1	2	All functioning well
**H3**	9584 (7.2%)	2	2	All functioning well
**H4**	1841 (1.4%)	1	1	All functioning well
**H5**	4021 (3%)	0	0	All oximeters in the neonatal ward
**H6**	13107 (9.9%)	2	2	All functioning well
**H7****	4164 (3.1%)	N/A	N/A	N/A
**H8**	8983 (6.8%)	0	2	Available intermittently. Waiting for supplier to provide longer-time solution
**H9**	9343 (7%)	2	1	One is faulty returned to supplier for repair
**H10**	8764 (6.6%)	2	2	All functioning well
**H11**	11538 (8.7%)	2	2	All functioning well
**H12**	6946 (5.2%)	2	1	One is faulty returned to supplier for repair
**H13**	10055 (7.6%)	1	3	All functioning well
**H14**	8494 (6.4%)	1	0	Stolen. Hospital following up
**H15**	919 (0.7%)	1	1	All functioning well
**H16**	1257 (0.9%)	2	1	One is faulty returned to supplier for repair
**H17**	9196 (6.9%)	0	17	All functional
**H18**	6824 (5.1%)	0	1	Functional. Shared with the Newborn ward.
**Note:**				

For peer review only

**We do not know the brands or specific types; majority of pulse oximeter machines will be portable devices based on authors' experiences of multiple visits to these facilities over this period.*

***Hospital exited from the CIN; Current status of pulse oximetry is therefore not available.*

Supplementary Table 2: Hospital participation in various studies by cluster

Group*	Study	Hospitals
**1**	None	H14
**1**	RTS, S	H18
**1**	RTS, S + SEARCH	H2, H6
**1**	SEARCH	H9, H10, H11, H12, H13
**2**	None	H7, H8, H17
**2**	SEARCH	H3
**3**	RTS, S	H4, H5
**3**	RTS, S + SEARCH	H1
**3**	SEARCH	H16
**3**	None	H15

Note: *The group represents the collection of hospitals in CIN based on joining date. The joining date for Groups 1, 2, and 3, was September 2013, February 2014, from November 2018 respectively. Group 3 hospitals were recruited to aid in increasing hospitals involved in RTS, S and SEARCH studies.

Supplementary Table 3: Discontinuity check due to strike period

(Outcome: Adoption rate per month)

Hospital*	Term**	Estimate***	std.error	statistic	p.value
**H11**	Post Strike Period:I(Time)	2.4660123	0.4525087	5.4496458	**0.012**
**H13**	Post Strike Period:I(Time)	-0.8160995	0.3053929	-2.6722938	0.076
**H10**	Post Strike Period:I(Time)	1.3733025	0.5193376	2.6443346	0.077
**H14**	Post Strike Period:I(Time)	0.2537052	0.1188514	2.1346419	0.122
**H8**	Post Strike Period:I(Time)	-0.9380901	0.4765890	-1.9683421	0.144
**H3**	Post Strike Period:I(Time)	-0.8265731	0.4394489	-1.8809311	0.157
**H18**	Post Strike Period:I(Time)	0.3832935	0.2233908	1.7157982	0.185
**H12**	Post Strike Period:I(Time)	1.2696169	1.0149519	1.2509134	0.300

For peer review only

Supplementary Table 3: Discontinuity check due to strike period (Outcome: Adoption rate per month)					
**Hospital***	**Term****	**Estimate*****	**std.error**	**statistic**	**p.value**
**H6**	Post Strike Period:I(Time)	-0.5999279	0.5146869	-1.1656172	0.328
**H2**	Post Strike Period:I(Time)	-0.3290568	0.2865754	-1.1482383	0.334
**H9**	Post Strike Period:I(Time)	-0.7842015	0.9577486	-0.8187968	0.473
**H17**	Post Strike Period:I(Time)	-0.0119919	0.7642561	-0.0156909	0.988

Note:

***Only hospitals recruited into CIN before the strike**

Time is in months, referencing 3 months before, and 3 months after the strike.**

***Estimate is in logit units.

Supplementary Table 4: Complete Case Analysis						
	**Pulse Oximetry Done? (n = 42776)**			**Oxygen Prescribed (n = 42772)**		
**Predictors**	**OR**	**95% CI**	**p-value**	**OR**	**95% CI**	**p-value**
(Intercept)	0.33	0.05 – 2.02	0.231	0.12	0.05 – 0.28	<0.001
**Hospital-level factors**						
Malaria Endemicity Zone [High]	1.03	0.31 – 3.36	0.963	1.02	0.58 – 1.78	0.947
Time (in months)	1.06	1.00 – 1.11	0.033	0.98	0.95 – 1.00	0.076
**Patient-level factors**						
Ref: Patient in any study [No]						
Patient in any study [Pre-study period]‡	1.47	1.30 – 1.67	<0.001	0.9	0.75 – 1.07	0.229
Patient in any study [Yes]	1.15	1.00 – 1.32	0.045	0.99	0.84 – 1.17	0.949
Referral: Yes (Ref: No)	1.05	0.98 – 1.12	0.143	1.24	1.14 – 1.35	<0.001
Readmission: Yes (Ref: No)	1.05	0.97 – 1.13	0.207	0.99	0.89 – 1.11	0.93
Is a weekend admission: Yes (Ref: No)	0.99	0.94 – 1.05	0.761	1.09	1.00 – 1.17	0.04

For peer review only

Supplementary Table 4: Complete Case Analysis						
	Pulse Oximetry Done? (n = 42776)			Oxygen Prescribed (n = 42772)		
**Predictors**	**OR**	**95% CI**	**p-value**	**OR**	**95% CI**	**p-value**
PAR used: Yes (Ref: No)	0.7	0.46 – 1.06	0.09	0.44	0.22 – 0.88	0.02
Time (in months)* PAR used: Yes (Ref: No)	1.01	1.00 – 1.03	0.051	1.03	1.01 – 1.06	0.005
Ref: Age [> 59 months]						
Age [1-11 months]	0.75	0.64 – 0.89	0.001	0.73	0.58 – 0.94	0.013
Age [12-59 months]	0.72	0.61 – 0.84	<0.001	0.56	0.44 – 0.71	<0.001
Female: Yes (Ref: No)	1.03	0.98 – 1.08	0.229	1.03	0.96 – 1.10	0.412
Fever: Yes (Ref: No)	1.03	0.97 – 1.11	0.324	0.81	0.74 – 0.89	<0.001
Cough: Yes (Ref: No)	0.99	0.94 – 1.04	0.626	1.3	1.19 – 1.41	<0.001
Difficulty Breathing: Yes (Ref: No)	1.09	1.02 – 1.15	0.007	2.54	2.34 – 2.76	<0.001
Stridor: Yes (Ref: No)	1.01	0.86 – 1.19	0.926	0.97	0.82 – 1.15	0.739
Cyanosis: Yes (Ref: No)	0.91	0.69 – 1.20	0.499	1.62	1.25 – 2.10	<0.001
Indrawing: Yes (Ref: No)	1.38	1.29 – 1.48	<0.001	2.12	1.95 – 2.31	<0.001
Grunting: Yes (Ref: No)	0.88	0.81 – 0.95	0.001	1.93	1.78 – 2.08	<0.001
Acidotic Breathing: Yes (Ref: No)	0.9	0.77 – 1.04	0.161	1.31	1.13 – 1.51	<0.001
Wheezing: Yes (Ref: No)	1.08	0.97 – 1.20	0.14	0.98	0.89 – 1.09	0.769
Crackles: Yes (Ref: No)	1.06	0.99 – 1.13	0.115	1.2	1.12 – 1.30	<0.001
Tachypnoea: Yes (Ref: No)	1.09	1.03 – 1.15	0.002	1.64	1.51 – 1.77	<0.001
Alert (AVPU < A): Yes (Ref: No)	0.89	0.79 – 1.00	0.042	1.77	1.57 – 2.01	<0.001
Inability to drink: Yes (Ref: No)	0.96	0.89 – 1.03	0.226	0.76	0.69 – 0.83	<0.001
Ref: Pallor [None]						
Pallor [Mild/Moderate]	1.02	0.94 – 1.12	0.574	1.23	1.10 – 1.37	<0.001
Pallor [Severe]	0.94	0.83 – 1.07	0.336	1.18	0.99 – 1.40	0.067
Ref: WAZ [Normal]						
WAZ [Low]	0.97	0.90 – 1.04	0.388	0.95	0.86 – 1.05	0.327
WAZ [Very Low]	1	0.93 – 1.08	0.97	0.9	0.82 – 0.99	0.033

For peer review only

Supplementary Table 4: Complete Case Analysis						
	Pulse Oximetry Done? (n = 42776)			Oxygen Prescribed (n = 42772)		
**Predictors**	**OR**	**95% CI**	**p-value**	**OR**	**95% CI**	**p-value**
Slow Capillary Refill: Yes (Ref: No)	0.76	0.61 – 0.94	0.013	0.8	0.60 – 1.06	0.119
Ref: Hypoxic [Unknown]						
Hypoxic [No]				0.54	0.50 – 0.59	<0.001
Hypoxic [Yes]				1.71	1.53 – 1.92	<0.001
Random effects						
σ^2	3.29			3.29		
τ_{00}	12.40 Hospital			0.23 Hospital		
τ_{11}	0.01 Hospital.Time			0.00 Hospital.Time		
ρ_{01}	-0.96 Hospital			-0.11 Hospital		
**ICC**	0.57			0.23		
**N**	17 Hospital			17 Hospital		
**Marginal R^2 / Conditional R^2**	0.144/ 0.631			0.271 / 0.442		

Supplementary Table 5: Oxygen prescription for $SpO_2 \geq 90\%$ OR SpO_2 Unknown						
	MICE model*			Complete Case Analysis**		
**Predictors**	**OR**	**95% CI**	**p-value**	**OR**	**95% CI**	**p-value**
(Intercept)	0.04	0.02 - 0.08	<0.001	0.08	0.03 – 0.19	<0.001
**Hospital-level factors**						
Malaria Endemicity Zone [High]	1.61	0.9 - 2.87	0.106	1.12	0.61 – 2.04	0.719
Time (in months)	1.01	1 - 1.02	0.152	0.97	0.95 – 1.00	0.032
**Patient-level factors**						
Ref: Patient in any study [No]						

For peer review only

Supplementary Table 5: Oxygen prescription for $SpO_2 \geq 90\%$ OR SpO_2 Unknown						
	MICE model*			Complete Case Analysis**		
**Predictors**	**OR**	**95% CI**	**p-value**	**OR**	**95% CI**	**p-value**
Patient in any study [Pre-study period]ǂ	0.99	0.86 - 1.12	0.824	0.88	0.73 – 1.06	0.168
Patient in any study [Yes]	0.94	0.83 - 1.07	0.349	0.92	0.78 – 1.10	0.369
Referral: Yes (Ref: No)	1.18	1.11 - 1.26	<0.001	1.28	1.17 – 1.40	<0.001
Readmission: Yes (Ref: No)	1.07	1 - 1.16	0.066	1.01	0.90 – 1.14	0.846
Is a weekend admission: Yes (Ref: No)	1.08	1.02 - 1.14	0.005	1.11	1.02 – 1.21	0.014
PAR used: Yes (Ref: No)	1.18	0.96 - 1.44	0.120	0.46	0.22 – 0.96	0.039
Time (in months)*PAR used: Yes (Ref: No)	1	1 - 1.01	0.560	1.03	1.01 – 1.06	0.013
Ref: Age [> 59 months]						
Age [< 1 month]	1.9	1.56 - 2.3	<0.001			
Age [1-11 months]	1.42	1.31 - 1.54	<0.001	0.79	0.61 – 1.03	0.078
Age [12-59 months]	1.06	0.98 - 1.15	0.163	0.59	0.46 – 0.76	<0.001
Female: Yes (Ref: No)	1.03	0.98 - 1.07	0.288	1.04	0.97 – 1.12	0.256
Fever: Yes (Ref: No)	0.76	0.72 - 0.81	<0.001	0.8	0.72 – 0.89	<0.001
Cough: Yes (Ref: No)	1.36	1.28 - 1.44	<0.001	1.3	1.19 – 1.42	<0.001
Difficulty Breathing: Yes (Ref: No)	2.88	2.72 - 3.05	<0.001	2.66	2.43 – 2.91	<0.001
Stridor: Yes (Ref: No)	1.12	1.01 - 1.26	0.039	0.99	0.83 – 1.17	0.865
Cyanosis: Yes (Ref: No)	1.61	1.36 - 1.91	<0.001	1.94	1.48 – 2.56	<0.001
Indrawing: Yes (Ref: No)	2.48	2.34 - 2.63	<0.001	2.27	2.07 – 2.48	<0.001
Grunting: Yes (Ref: No)	2.03	1.92 - 2.15	<0.001	2.09	1.92 – 2.27	<0.001
Acidotic Breathing: Yes (Ref: No)	1.37	1.24 - 1.51	<0.001	1.38	1.19 – 1.61	<0.001
Wheezing: Yes (Ref: No)	1.17	1.08 - 1.26	<0.001	1.04	0.93 – 1.16	0.542
Crackles: Yes (Ref: No)	1.27	1.2 - 1.34	<0.001	1.21	1.12 – 1.32	<0.001
Tachypnoea: Yes (Ref: No)	1.63	1.54 - 1.72	<0.001	1.7	1.56 – 1.85	<0.001
Alert (AVPU < A): Yes (Ref: No)	2.02	1.86 - 2.18	<0.001	1.83	1.60 – 2.08	<0.001
Inability to drink: Yes (Ref: No)	0.8	0.75 - 0.85	<0.001	0.76	0.69 – 0.84	<0.001

For peer review only

Supplementary Table 5: Oxygen prescription for $SpO_2 \geq 90\%$ OR SpO_2 Unknown						
	MICE model*			Complete Case Analysis**		
**Predictors**	**OR**	**95% CI**	**p-value**	**OR**	**95% CI**	**p-value**
Ref: Pallor [None]						
Pallor [Mild/Moderate]	1.11	1.04 - 1.2	<0.001	1.26	1.12 – 1.41	<0.001
Pallor [Severe]	1.34	1.22 - 1.46	<0.001	1.14	0.95 – 1.38	0.153
Ref: WAZ [Normal]						
WAZ [Low]	1	0.93 - 1.07	0.952	0.97	0.87 – 1.08	0.596
WAZ [Very Low]	0.96	0.89 - 1.03	0.273	0.92	0.83 – 1.02	0.122
Slow Capillary Refill: Yes (Ref: No)	1.03	0.88 - 1.2	0.708	0.87	0.65 – 1.18	0.371

Note: ******n=123,952; 4275 cases omitted from MICE analysis because of missing outcome (Figure 4)*

*******n=40,491*

Supplementary Table 6: Admission diagnoses for patients getting oxygen with pulse oximetry ≥ 90% at admission.	
**Diagnosis**	**Documented Cases, N (%)**
Pneumonia	18668 (34.14%)
Malaria	16559 (30.28%)
Dehydration	10641 (19.46%)
Diarrhoea	10592 (19.37%)
Anaemia	8018 (14.66%)
Meningitis	5875 (10.74%)
Malnutrition	5351 (9.78%)
Sickle Cell	2380 (4.35%)
Asthma	1240 (2.27%)
Rickets	1084 (1.98%)
TB	907 (1.6s6%)
Sepsis	708 (1.29%)
HIV	716 (1.31%)

For peer review only

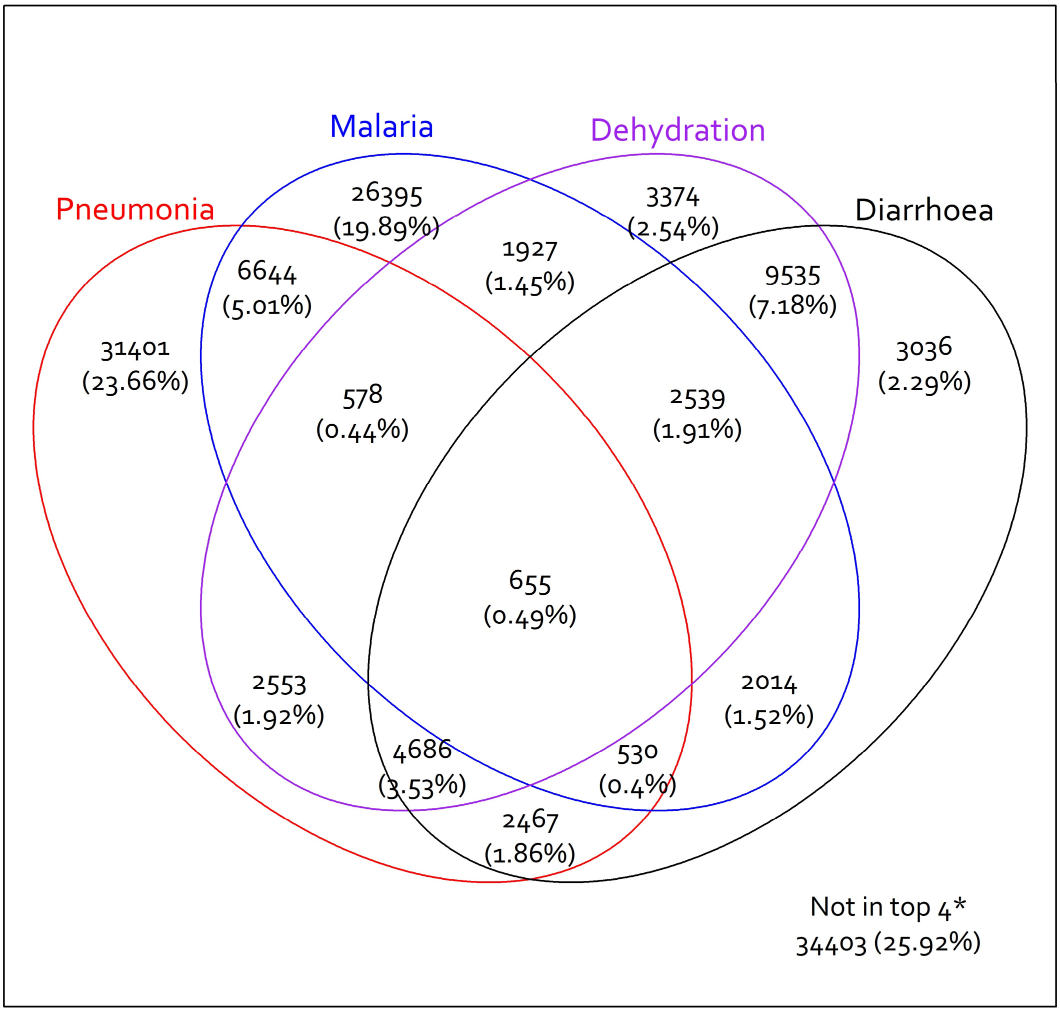

Supplementary Figure 1: Common admission diagnoses in CIN hospitals.

From the linear growth-curve model, the random intercept conveys the hospital's average pulse oximetry adoption rate over the time period in months it has participated in CIN. The random slope conveys the hospital's average monthly increase or decrease in adoption rate over the time period they have been part of the CIN. The baseline for the random intercept and slope in the growth curve model is the overall level of the response which is allowed to vary between hospitals after controlling for covariates.

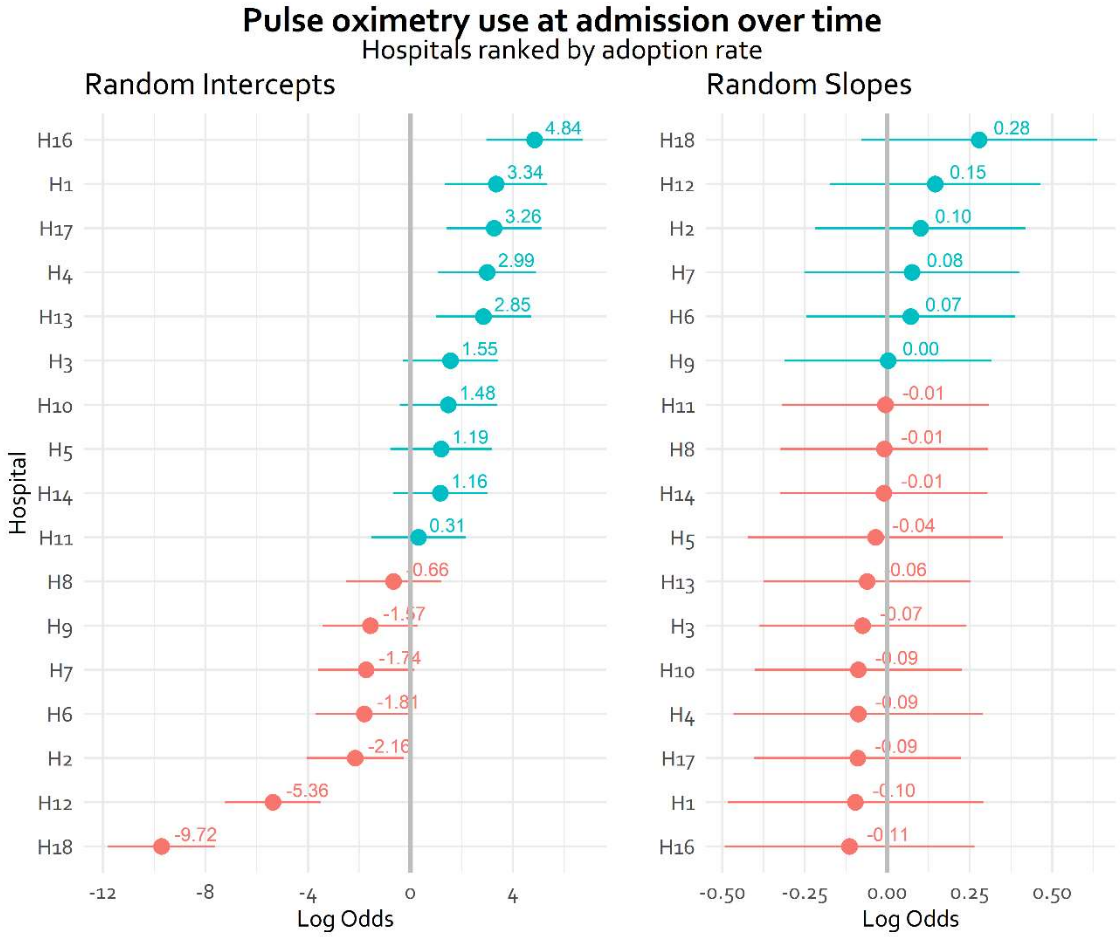

Supplementary Figure 2: Ranking of hospitals pulse oximetry use at admission

view only

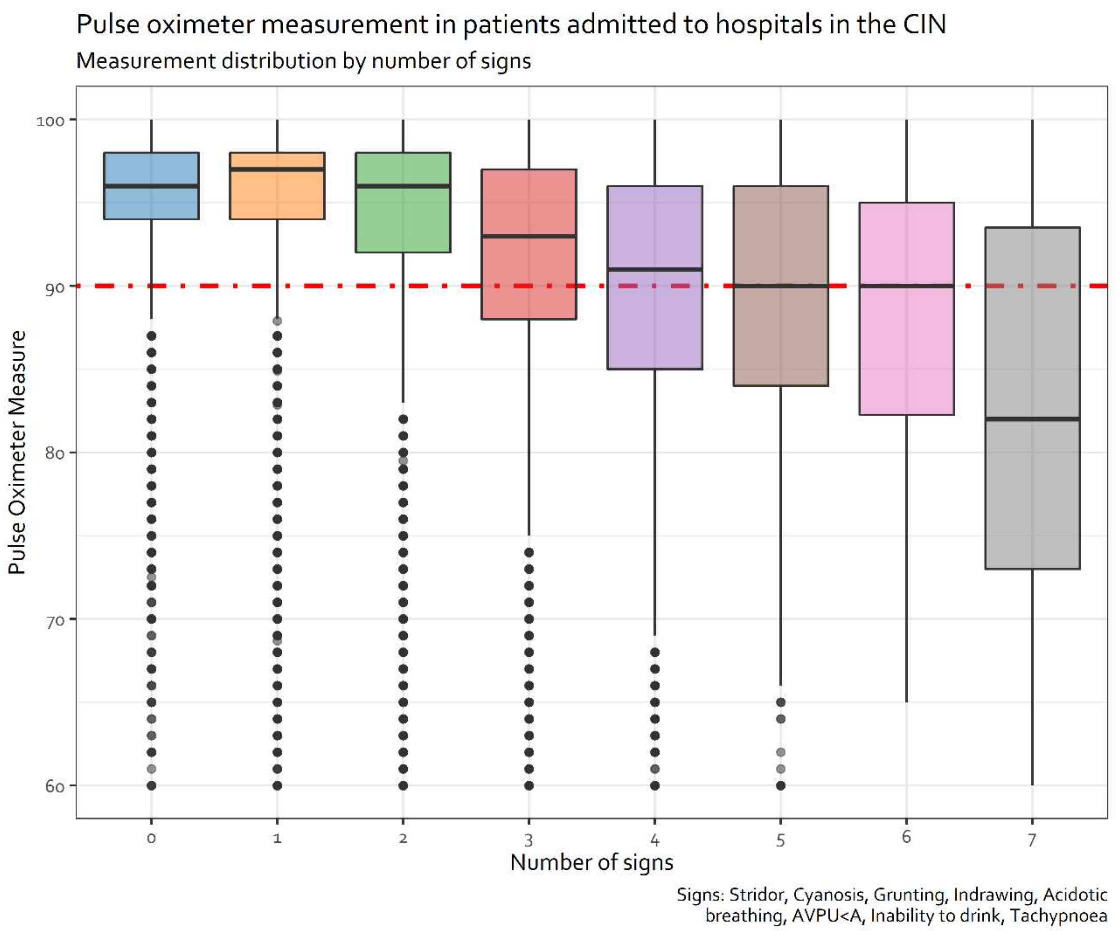

Supplementary Figure 3: Oxygen saturation measure by the number of respiratory distress symptoms

STROBE Statement—checklist of items that should be included in reports of observational studies

	Item No	Recommendation	Page No
**Title and abstract**	1	(*a*) Indicate the study's design with a commonly used term in the title or the abstract	1
		(*b*) Provide in the abstract an informative and balanced summary of what was done and what was found	1-2
**Introduction**			
Background/rationale	2	Explain the scientific background and rationale for the investigation being reported	55-76
Objectives	3	State specific objectives, including any prespecified hypotheses	76-79, 193-196
**Methods**			
Study design	4	Present key elements of study design early in the paper	92-132
Setting	5	Describe the setting, locations, and relevant dates, including periods of recruitment, exposure, follow-up, and data collection	92-132
Participants	6	(*a*) *Cohort study*—Give the eligibility criteria, and the sources and methods of selection of participants. Describe methods of follow-up<br>*Case-control study*—Give the eligibility criteria, and the sources and methods of case ascertainment and control selection. Give the rationale for the choice of cases and controls<br>*Cross-sectional study*—Give the eligibility criteria, and the sources and methods of selection of participants	133-143
		(*b*) *Cohort study*—For matched studies, give matching criteria and number of exposed and unexposed<br>*Case-control study*—For matched studies, give matching criteria and the number of controls per case	
Variables	7	Clearly define all outcomes, exposures, predictors, potential confounders, and effect modifiers. Give diagnostic criteria, if applicable	169-189
Data sources/ measurement	8*	For each variable of interest, give sources of data and details of methods of assessment (measurement). Describe comparability of assessment methods if there is more than one group	164-178
Bias	9	Describe any efforts to address potential sources of bias	205-213
Study size	10	Explain how the study size was arrived at	134-143
Quantitative variables	11	Explain how quantitative variables were handled in the analyses. If applicable, describe which groupings were chosen and why	169-189
Statistical methods	12	(*a*) Describe all statistical methods, including those used to control for confounding	178-213
		(*b*) Describe any methods used to examine subgroups and interactions	190-196
		(*c*) Explain how missing data were addressed	209-213

For peer review only - http://bmjopen.bmj.com/site/about/guidelines.xhtml

(*d*) *Cohort study*—If applicable, explain how loss to follow-up was addressed<br>*Case-control study*—If applicable, explain how matching of cases and controls was addressed<br>*Cross-sectional study*—If applicable, describe analytical methods taking account of sampling strategy	197-213
(*e*) Describe any sensitivity analyses	

Continued on next page

For peer review only

**Results**			
Participants	13*	(a) Report numbers of individuals at each stage of study—eg numbers potentially eligible, examined for eligibility, confirmed eligible, included in the study, completing follow-up, and analysed	215 - 221
		(b) Give reasons for non-participation at each stage	Figure 1
		(c) Consider use of a flow diagram	Figure 1
Descriptive data	14*	(a) Give characteristics of study participants (eg demographic, clinical, social) and information on exposures and potential confounders	226-293
		(b) Indicate number of participants with missing data for each variable of interest	Table 2
		(c) *Cohort study*—Summarise follow-up time (eg, average and total amount)	
Outcome data	15*	*Cohort study*—Report numbers of outcome events or summary measures over time	Table 2, Figures 2-3
		Case-control study—Report numbers in each exposure category, or summary measures of exposure	
		Cross-sectional study—Report numbers of outcome events or summary measures	Table 2, Figure 4, 279-286
Main results	16	(*a*) Give unadjusted estimates and, if applicable, confounder-adjusted estimates and their precision (eg, 95% confidence interval). Make clear which confounders were adjusted for and why they were included	294-357
		(*b*) Report category boundaries when continuous variables were categorized	279-280
		(*c*) If relevant, consider translating estimates of relative risk into absolute risk for a meaningful time period	
Other analyses	17	Report other analyses done—eg analyses of subgroups and interactions, and sensitivity analyses	241- 247, 331-333, Supplementary Tables 3 - 5, Table 3
**Discussion**			
Key results	18	Summarise key results with reference to study objectives	359-365
Limitations	19	Discuss limitations of the study, taking into account sources of potential bias or imprecision. Discuss both direction and magnitude of any potential bias	440-459
Interpretation	20	Give a cautious overall interpretation of results considering objectives, limitations, multiplicity of analyses, results from similar studies, and other relevant evidence	366-431
Generalisability	21	Discuss the generalisability (external validity) of the study results	433-442
**Other information**			
Funding	22	Give the source of funding and the role of the funders for the present study and, if applicable, for the original study on which the present article is based	520-534

*Give information separately for cases and controls in case-control studies and, if applicable, for exposed and unexposed groups in cohort and cross-sectional studies.

For peer review only - http://bmjopen.bmj.com/site/about/guidelines.xhtml

For peer review only

Note: An Explanation and Elaboration article discusses each checklist item and gives methodological background and published examples of transparent reporting. The STROBE checklist is best used in conjunction with this article (freely available on the Web sites of PLoS Medicine at http://www.plosmedicine.org/, Annals of Internal Medicine at http://www.annals.org/, and Epidemiology at http://www.epidem.com/). Information on the STROBE Initiative is available at www.strobe-statement.org.

For peer review only

For peer review only - http://bmjopen.bmj.com/site/about/guidelines.xhtml
